# Element-Specific Donor Numbers for Anions

**DOI:** 10.1021/jacs.6c06567

**Published:** 2026-08-13

**Authors:** Lewis G. Parker, Frances K. Towers Tompkins, Jake M. Seymour, Ekaterina Gousseva, Christopher D. Smith, Roger A. Bennett, Pilar Ferrer, David C. Grinter, Claudia Kolbeck, Dennis Hein, Garlef Wartner, Robert Seidel, Denis Céolin, Robert Temperton, Robert G. Palgrave, Richard P. Matthews, Kevin R. J. Lovelock

**Affiliations:** 1 Department of Chemistry, 6816 University of Reading, Reading RG6 6DX, U.K.; 2 Department of Chemistry, 1555 University of Bath, Bath BA2 7AY, U.K.; 3 120796 Diamond Light Source, Didcot, Oxfordshire OX11 0DE, U.K.; 4 28259 Fritz-Haber-Institut der Max-Planck-Gesellschaft, 14195 Berlin, Germany; 5 28340 Helmholtz-Zentrum Berlin für Materialien und Energie GmbH, 14109 Berlin, Germany; 6 Synchrotron SOLEIL, 91192 Gif sur Yvette, France; 7 MAX IV Laboratory, 5193 Lund University, 221 00 Lund, Sweden; 8 Department of Chemistry, 4919 University College London, London WC1H 0AJ, U.K.; 9 School of Health, Sport and Bioscience, 4917 University of East London, London E15 4LZ, U.K.

## Abstract

Anion interactions with cations/molecules are crucial across chemistry and biology: in battery electrolytes, synthesis, atmospheric processes, and protein denaturation. Selecting the optimum anion represents an immense challenge, as anion interactions are diverse and complex. Descriptors are needed that quantitatively capture the ability of anions to interact with cations and molecules. However, only a small number of experimental anion interaction descriptors exist, all with similar limitations. Here we show that core-level anion electron binding energies, *E*_B_(core,anion), can be used to produce a new atomic-level descriptor for each of the key donor elements for anion interactions (O, N, F, Cl, Br, I, S), the element-specific donor number (DN_E‑XPS_). DN_E‑XPS_ descriptors are intrinsic, capturing the anion interaction abilities independent of any probe, countercation, or solvent. DN_E‑XPS_ descriptors are also interpretable, as *E*_B_(core,anion) and therefore DN_E‑XPS_ are proportional to the electrostatic potential at the specific donor atom nucleus. Experimental core-level X-ray photoelectron spectroscopy (XPS) is used to measure *E*_B_(core,anion) and therefore DN_E‑XPS_. Furthermore, DN_E‑XPS_ descriptors are produced easily and at low cost using lone-anion-SMD (solvation model density) calculations, a significant advance on the current anion descriptors, greatly reducing and potentially removing the need for experimental anion characterization. This work will greatly facilitate anion selection, especially for complex anions capable of forming interactions through multiple different atoms. We envisage our calculation method enabling the production of a very large database of DN_E‑XPS_ for each key element, ideal for use as machine learning training data sets.

## Graphical abstract

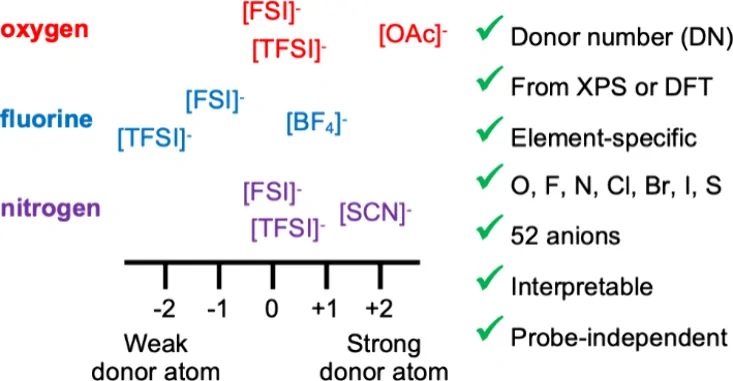

## 1 Introduction

Interactions of anions with cations/molecules/solids play a key role in a vast number of chemical, biological, and industrial processes. For energy storage devices,^1,2^ anion–metal cation interactions in battery electrolytes can both ease metal cation desolvation before deposition/intercalation^3–5^ and facilitate superior solid-electrolyte interphase (SEI) formation.^1,6–9^ Furthermore, strong anion–solvent interactions in the battery electrolyte can enhance solvent reductive stability, reducing unwanted electrochemical decomposition.^10–12^ In the realm of chemical synthesis,^13–20^ the strength of anion–hydroxyl group interactions is an important factor for deconstruction of lignocellulosic biomass into biofuels.^13^ Moreover, weakly coordinating anions are important for both (stereoselective) cationic catalysts^17,18^ and stabilization of highly electrophilic/oxidizing cations.^16^ Lastly, in the electrochemical synthesis of CO_2_ for fuel production, anion–metal surface interactions significantly impact both kinetics and selectivity.^19,20^ For biological processes,^21,22^ protein denaturation can be directed by the strength of anion–protein interactions.^21^

Selecting the optimum anion for an application represents an immense challenge. The number of anions to choose from is large and growing.^23–29^ Anion structures are diverse: from monatomic to large polyatomic; from highly symmetric to relatively asymmetric (examples in Figure 1). Anion interaction/binding modes to cations/molecules are complex. Most anions have multiple potential donor atoms and so can bind mono-/bi-/tridentate, depending on the solution composition, e.g., [PF_6_]^−^ with Li^+^ can be mono-/bi-/tridentate.^30–34^ A further complication can be anions that contain more than one type of donor atom (Figure 1 and Supporting Information Table S2); the primary (1°) donor atom will dominate anion interactions (e.g., O donor atoms for [(CF_3_SO_2_)_2_N]^−^), but strong interactions of secondary/tertiary (2°/3°) donor atoms are also influential, e.g., F donor atoms of [(CF_3_SO_2_)_2_N]^−^ for LiF formation in SEIs.^35,36^

**Figure 1:**
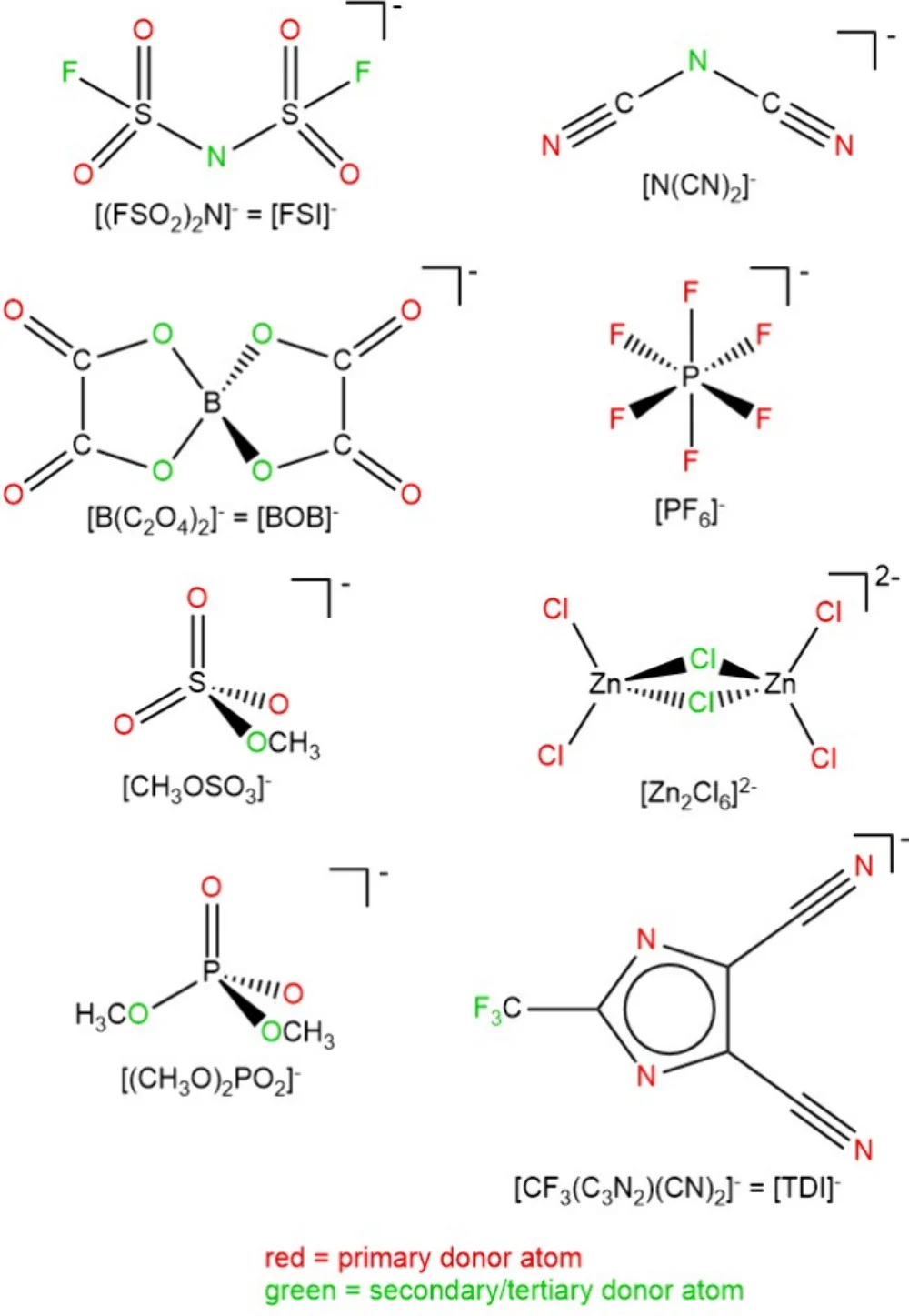
Selected anions with primary/secondary/tertiary (1°/2°/3°) donor atoms labeled.

Descriptors that quantify anion interactions are needed to rationalize and optimize anion selection. Historically, trial-and-error has been used to some extent to select good candidate anions.^37–41^ More recently, data-driven methods have come to the fore.^37,42,43^ Experimental anion descriptors based on the spectroscopy of anion-probe interactions, summarized in Table 1,^37,43–53^ are now routinely used to aid anion selection, especially in the ionic liquid^13^ and battery communities.^54–59^ Probes used are either a cation (Na^+^,^44^ Li^+^,^45^ dialkylimidazolium cation,^46–49^ Cu^+^ complex^46^) to produce donor number (DN) descriptors or heterocyclic dye molecules to produce hydrogen bond acceptor (β) descriptors.^50–53^ The most prominent of these anion descriptors were originally developed as solvent descriptors in the 1970s (Kamlet–Taft β_KT_, and DN from ^23^Na NMR, DN_Na‑NMR_),^60–62^ highlighting that new interaction strength descriptors are rarely discovered. Furthermore, the current experimental anion descriptors have several critical limitations, as discussed in the next four paragraphs.

**Table 1:** Strengths and Weaknesses of Experimental Anion Descriptors That Capture Intermolecular Anion–Cation/Anion–Molecule Interaction Strengths

descriptor	ref	spectroscopic method	probe	atomic-level?	probe-independent?	readily calculated?	interpretability
β_KT_	^50^,^51^	UV–vis	heterocyclic dye molecules	X	X	X	Reasonable
β_NMR_	^52^	^19^F NMR	fluorinated heterocyclic dye molecules	X	X	X	Reasonable
β_titr_	^53^	UV–vis	heterocyclic dye molecules	X	X	X	Reasonable
DN_Na‑NMR_	^44^	^23^Na NMR	Na^+^	X	X	X	Challenging
DN_Li‑NMR_	^45^	^7^Li NMR	Li^+^	X	X	X	Very challenging
DN_Im‑NMR_	^46^,^47^	^1^H NMR	[C_4_C_1_Im]^+^	X	X	X	Challenging
DN_Im‑XPS_	^48^,^49^	XPS	[C_8_C_1_Im]^+^	X	X	X	Excellent
DN_Cu‑UV_	^46^	UV–vis	cationic Cu complex	X	X	X	Reasonable
DN_E‑XPS_	here	XPS	no probe	√	√	√	Excellent

Anion descriptors are needed that provide atomic-level insights into interactions. For solvents, there has been a recent realization that single-value interaction strength descriptors do not provide atomic-level insight and are insufficient to capture interaction complexity.^63–68^ For example, calculated electrostatic potential (ESP) descriptors are a recent and growing area in the battery community to identify which part of the solvent/additive molecule has good electron donor ability,^63,64^ including the oxygen atom coordinated to Li^+^.^68^ Given that anion interactions are more complex than solvent interactions, the single value to describe the whole anion provided by the existing anion descriptors leaves significant room for improvement.

There is strong uncertainty over the general applicability of the current probe-dependent experimental anion descriptors. The anion-substrate interaction relevant to an application will often be very different to the anion-probe interaction captured by the anion descriptor.^66^ Moreover, anion descriptors are probe-dependent (Table 1); probe-cation/probe-solvent interactions contribute (see Supporting Information section 1 for more details).^44,50–52^ Thus, the current anion descriptors are not intrinsic measures of anion properties.^37,43–53^ Intrinsic anion descriptors do not exist due to the limitations of the current spectroscopic toolbox; NMR, IR and Raman spectroscopies are all limited to certain elements/functional groups in anions, and the 1° donor atom for anions varies greatly, e.g., O in [(CF_3_S**O**_2_)_2_N]^−^, F in [P**F**_6_]^−^, N in [B­(C**N**)_4_]^−^, Cl in [Zn**Cl**_4_]^2–^ (examples in Figure 1).

Expensive anion synthesis combined with descriptor measurement is currently essential to understand and predict anion interaction strengths. The existing experimental anion descriptors have not been calculated using high accuracy and low-cost methods. One attempt for anion β_KT_ concluded that their method, calculation of the spectroscopic signature of relatively large anion-probe structures, was not suitable for routine screening.^69^ Furthermore, the accuracy of anion descriptors from low-cost calculations, e.g., Fukui functions,^70–72^ is under question as they have not been validated against experimental anion descriptors; gas-phase lone-anion calculations generally do not capture liquid-phase anion properties.^73^ Machine learning (ML) has been used to predict interaction strength descriptors for solvents/molecules^74–77^ but not for anions to date.

For existing experimental anion descriptors, interpretability remains an issue; correlations between common/easily understood anion physicochemical properties and the experimental anion descriptors are sorely lacking (Table 1). This is caused by the same problems that limit calculations: lack of understanding of both the specific anion-probe interactions and the spectroscopies (especially NMR^78–80^ ). Interpretable experimental anion descriptors can provide a route to design rules for anion selection.

Here we use the element specificity of core-level X-ray photoelectron spectroscopy (XPS),^81^ along with easy and low-cost DFT calculations, to obtain core-level anion electron binding energies, *E*_B_(core,anion), for key elements for anion electron donation (O, N, F, Cl, Br, I, S). Both liquid-jet XPS apparatus (for anions in molecular solvents) and liquid drop apparatus (for anions in ionic liquids) are used to experimentally measure *E*_B_(core,anion). Lone-anion SMD (solvation model density) calculations are used to calculate *E*_B_(core,anion). We then convert these *E*_B_(core,anion) into new element-specific donor number (DN_E‑XPS_) descriptors for 52 anions in total across the seven elements (Supporting Information Table S2). For *E*_B_(core,anion) in ionic liquids, we use a combination of data measured here and literature data;^48,73,82–95^ the focus of literature studies involving *E*_B_(core,anion) has previously been anion speciation^86,92^ and not anion descriptor generation. Here we provide the first XPS measurements for the same anions in different molecular solvents and make crucial comparisons to XPS for anions in ionic liquids for the first time. There is little *E*_B_(core,anion) data for anions solvated in molecular solvents;^96,97^ the focus has been on *E*_B_(valence,anion) for anions in water.^98–100^ *E*_B_(core,anion) and therefore DN_E‑XPS_ have the potential to capture anion interaction strength.^101^ The 52 anions (Supporting Information Table S2) are chosen based on a combination of relevance to specific applications (batteries, synthesis and catalysis, atmospheric chemistry, gas capture, and protein denaturation) and covering the range of possible donor atom types. The solvents studied here cover an excellent range of solvation environments; water is a good electron acceptor solvent, whereas triethyl phosphate (TEP), propylene carbonate (PC), and dialkylimidazolium (the countercation used here for ionic liquids) are relatively poor electron acceptors.^102,103^

## 2 Results and Discussion

### 2.1 Element-Specific Anion Donor Number Values: Atomic-Level Descriptors

We have measured XPS-derived element-specific donor numbers, DN_E‑XPS_, for the first time. DN_E‑XPS_ provide experimental atomic-level descriptors for primary/secondary/tertiary (1°/2°/3°) donor atoms for anions. Small *E*_B_(core,anion) → small *V*_n_ (*V*_n_ is the electrostatic potential at the nucleus, where n is the nucleus of a specific atom) → large DN_E‑XPS_ → strong donor atom (Figure 2, top). In contrast, large *E*_B_(core,anion) → large *V*_n_ → small DN_E‑XPS_ → weak donor atom (Figure 2, bottom).

**Figure 2:**
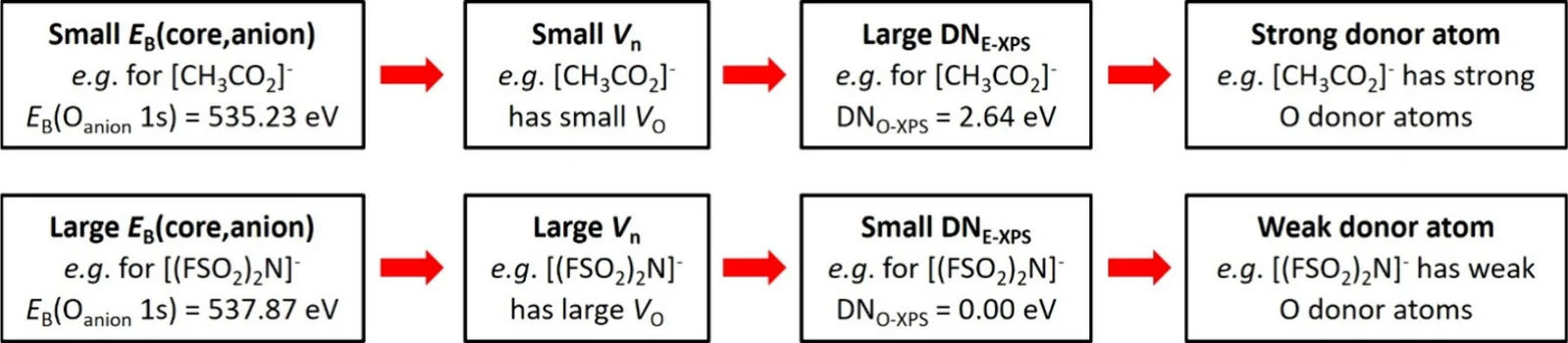
Relationship between XPS electron binding energies, *E*_B_(core,anion), e.g., *E*_B_(O_anion_ 1s), electrostatic potential at the nucleus (as defined in ref 101), *V*_n_, e.g., *V*_O_, element-specific donor number, DN_E‑XPS_, e.g., DN_O‑XPS_, and donor atom strength/ability.

As this publication represents the first time that XPS has been used to obtain DN_E‑XPS_, here we show exemplar O_anion_ 1s XP spectra used to obtain DN_O‑XPS_ for seven anions in ionic liquids (Figure 3, Supporting Information Figure S56 for O_anion_ 1s XP spectra of all 15 anions studied here). In core-level XPS, different covalent bonding environments can be distinguished for the same element in a single anion given sufficiently different *E*_B_(core,anion); peak area ratios for the same element match the stoichiometry of the anion, which allows the contributions from anion structural moieties to be identified in the experimental XP spectra. The two different O atom bonding environments in [ROSO_3_]^−^ (R = H, CH_3_, C_8_H_17_), 1° donor SO_3_ and 2° donor RO (structure for R = CH_3_ in Figure 1), are well-separated in *E*_B_(O_anion_ 1s) and in a 3:1 area ratio (Figure 3 and Supporting Information Figure S56); *E*_B_(O_anion_ 1s) and therefore DN_O‑XPS_ can be measured for both SO_3_ and RO. Different covalent bonding environments can be distinguished for other anions: O_PO_ and O_OC_ in [(CH_3_O)_2_PO_2_]^−^ (structure in Supporting Information Table S2, XP spectrum in Supporting Information Figure S56), N_terminal_ and N_bridging_ in [N­(CN)_2_]^−^ (structure in Figure 1, XP spectrum in Supporting Information Figure S57), F_PF_ and F_CF_ in [(C_2_F_5_)_3_PF_3_]^−^ (structure in Supporting Information Table S2, XP spectrum in Supporting Information Figure S58), terminal and bridge halides in halozincates (example structure in Figure 1, XP spectra in Supporting Information Figures S59 and S60). Furthermore, anions that are too challenging to measure for other experimental descriptors (e.g., I^–^ or open-shell metal complexes for β based on UV–vis spectroscopy) can be measured using core-level XPS. Calculations can facilitate identification of atoms of the same element with different covalent bonding with similar donor strength in the same anion, which is very challenging for experimental XPS, e.g., for oxygen in [B­(C_2_O_4_)_2_]^−^ (structure in Figure 1), O_BO_ and O_CO_ bonding environments can be readily distinguished in the calculated O 1s XP spectrum (Figure 3, structure in Figure 1).

**Figure 3:**
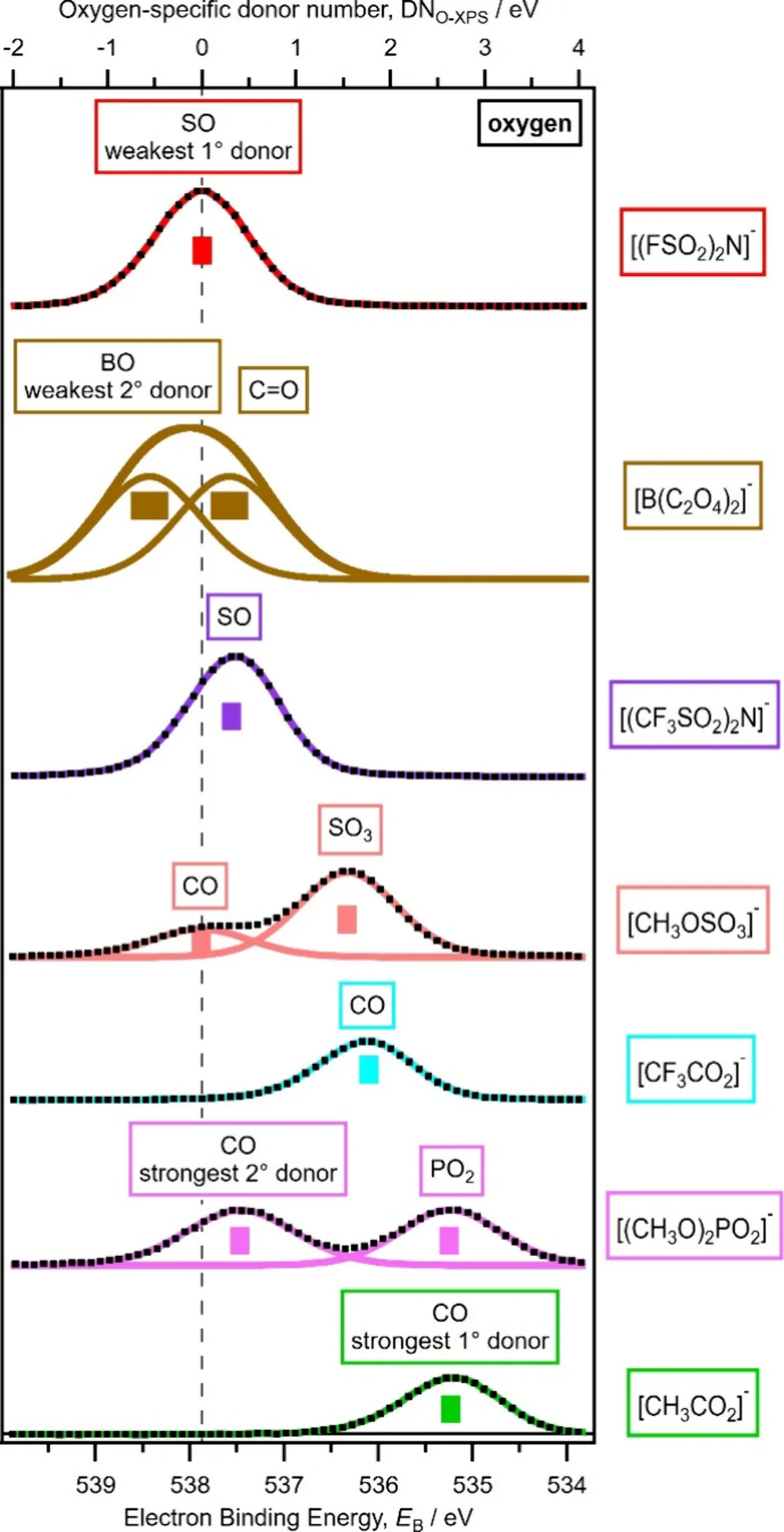
O 1s XP spectra (dots are XP spectra, colored traces are fits) and values of *E*_B_(O_anion_ 1s) and DN_O‑XPS_ (bottom and top axes, respectively) of oxygen-containing anions in ionic liquid: [C_8_C_1_Im]­[(FSO_2_)_2_N]; [B­(C_2_O_4_)_2_]^−^ (total and also calculated C=O and BO contributions); [C_8_C_1_Im]­[(CF_3_SO_2_)_2_N]; [C_4_C_1_Im]­[CH_3_OSO_3_] (total and also fitted CO and SO_3_ components); [C_8_C_1_Im]­[CF_3_CO_2_]; [C_4_C_1_Im]­[(CH_3_O)_2_PO_2_] (total and also fitted CO and PO_2_ components); [C_4_C_1_Im]­[CH_3_CO_2_]. Traces are vertically offset for clarity. Area normalization is to the number of atoms in each anion and explained further in Supporting Information section 8.3. Charge referencing is given in Supporting Information section 8.2. For *E*_B_(O_anion_ 1s) (and therefore DN_O‑XPS_), the experimental uncertainty is ±0.10 eV (Supporting Information section 14.2) and the calculated uncertainty is ±0.20 eV (Supporting Information section 14.4); these uncertainty values are represented by the width of each block.

For four of the 52 anions studied here ([(CF_3_SO_2_)_2_N]^−^ (also known as [NTf_2_]^−^ or [TFSI]^−^), [(C_2_F_5_SO_2_)_2_N]^−^ (also known as [NPf_2_]^−^ or [BETI]^−^), [(FSO_2_)_2_N]^−^ (also known as [FSI]^−^), and [SCN]^−^), the identity of the primary donor atom depends on the cation/molecule the anion is interacting with, e.g., O or N for [(CF_3_SO_2_)_2_N]^−^. We have identified the primary donor atom for these four anions as the atom type that most commonly interacts with cations/molecules using a combination of experiments^104–106^ and calculations:^35^ O for [(CF_3_SO_2_)_2_N]^−^, [(C_2_F_5_SO_2_)_2_N]^−^, and [(FSO_2_)_2_N]^−^; N for [SCN]^−^.

DN_E‑XPS_ values for seven elements are each produced using the same method (see Figure S9 and Figure S10 for two flowcharts explaining the method in detail). Values of DN_E‑XPS_ for each element are obtained using a combination of *E*_B_(core,anion) (Supporting Information Figures S56–S62) and the appropriate equation (Table 2, column 2); DN_E‑XPS_ values are given in Table 3 to Table 6, Supporting Information Tables S52–S54). The equations for DN_E‑XPS_ (Table 2, column 2) are produced using *E*_B_(core,anion) for the weakest 1° donor atom for each element (anion identified in Table 2, column 3, *E*_B_(core,anion) value given in Table 2, column 4). For example, [(FSO_2_)_2_N]^−^ is the weakest 1° oxygen donor atom presented here, giving the largest *E*_B_(O_anion_ 1s) for a 1° oxygen donor atom, *E*_B_(O_anion_ 1s) = 537.87 eV. Therefore, [(FSO_2_)_2_N]^−^ is set to DN_O‑XPS_ = 0 eV to produce the equation for oxygen in Table 2, row 2 (see top axis on Figure 3, dashed vertical line represents DN_O‑XPS_ = 0 eV and Figure 2), We chose not to scale DN_E‑XPS_ between 0 (weakest donor) and 1 (strongest donor), e.g., as is done for β_KT_, because ΔDN_E‑XPS_ are directly related to Δ*V*_n_ (see section 2.4).

**Table 2:** Summary of Equations and Data for Element-Specific Donor Numbers from XPS, DN_E‑XPS_

element	equation for DN_E‑XPS_	anion with largest *E*_B_(core,anion) for 1° donor atoms	largest *E*_B_(core,anion) for 1° donor atoms/eV
O	DN_O‑XPS_ = 537.87 – *E*_B_(O_anion_ 1s)	[(FSO_2_)_2_N]^−^	537.87
N	DN_N‑XPS_ = 404.60 – *E*_B_(N_anion_ 1s)	[B(CN)_4_]^−^	404.60
F	DN_F‑XPS_ = 691.59 – *E*_B_(F_anion_ 1s)	[PF_6_]^−^	691.59
Cl	DN_Cl‑XPS_ = 204.39 – *E*_B_(Cl_anion_ 2p_3/2_)	[GaCl_4_]^−^	204.39
Br	DN_Br‑XPS_ = 74.42 – *E*_B_(Br_anion_ 3d_5/2_)	[InBr_4_]^−^	74.42
I	DN_I‑XPS_ = 624.15 – *E*_B_(I_anion_ 3d_5/2_)	[I_3_]^−^	624.15
S	DN_S‑XPS_ = 167.93 – *E*_B_(S_anion_ 2p_3/2_)	[Co(NCS)_4_]^2–^	167.93

**Table 3:** Experimental (Nonitalicized entries) And Calculated (Italicized Entries, Taken from Table S35) *E*_B_(O_anion_ 1s) and Oxygen-Specific Donor Numbers (DN_O‑XPS_) for Ionic Liquid Samples^a^

anion	moiety	1°/2°/3°	*E*_B_(O_anion_ 1s)/eV	DN_O‑XPS_/eV
[CH_3_CO_2_]^−^	O	Strongest 1°	535.23 ± 0.10	2.64 ± 0.10
[(CH_3_O)_2_PO_2_]^−^	O_PO_	1°	535.25 ± 0.10	2.62 ± 0.10
[CH_3_SO_3_]^−^	O	1°	536.05 ± 0.10	1.82 ± 0.10
*[CH*_*3*_ *C*_*6*_ *H*_*4*_ *SO*_*3*_ *]* ^*–*^	*O*	*1°*	*536.06 ± 0.20*	*1.81 ± 0.20*
[CF_3_CO_2_]^−^	O	1°	536.10 ± 0.10	1.77 ± 0.10
[C_8_H_17_OSO_3_]^−^	O_SO_	1°	536.28 ± 0.10	1.59 ± 0.10
[CH_3_OSO_3_]^−^	O_SO_	1°	536.33 ± 0.10	1.54 ± 0.10
[HOSO_3_]^−^	O_SO_	1°	536.48 ± 0.10	1.39 ± 0.10
[CF_3_SO_3_]^−^	O	1°	536.74 ± 0.10	1.13 ± 0.10
[NO_3_]^−^	O	1°	536.95 ± 0.10	0.92 ± 0.10
[(CF_3_SO_2_)_2_N]^−^	O	1°	537.56 ± 0.10	0.31 ± 0.10
[(C_2_F_5_SO_2_)_2_N]^−^	O	1°	537.56 ± 0.10	0.31 ± 0.10
*[ClO*_*4*_ *]* ^*‑*^	*O*	*1°*	*537.57 ± 0.20*	*0.30 ± 0.20*
*[B(C*_*2*_ *O*_*4*_ *)*_*2*_ *]* ^*–*^	*O*_*CO*_	*1°*	*537.58 ± 0.20*	*0.29 ± 0.20*
[(FSO_2_)_2_N]^−^	O	Weakest 1°	537.87 ± 0.10	0.00 ± 0.10
[(CH_3_O)_2_PO_2_]^−^	O_OC_	Strongest 2°	537.47 ± 0.10	0.40 ± 0.10
[C_8_H_17_OSO_3_]^−^	O_OC_	2°	537.71 ± 0.10	0.16 ± 0.10
[HOSO_3_]^−^	O_OH_	2°	537.88 ± 0.10	–0.01 ± 0.10
[CH_3_OSO_3_]^−^	O_OC_	2°	537.88 ± 0.10	–0.01 ± 0.10
*[B(C*_*2*_ *O*_*4*_ *)*_*2*_ *]* ^*–*^	*O*_*BO*_	*Weakest 2°*	*538.43 ± 0.20*	*–0.56 ± 0.20*

a Experimental uncertainties are ±0.10 eV (Supporting Information section 14.2), and calculated uncertainties are ±0.20 eV (Supporting Information section 14.4).

The strongest donor atoms have the largest positive DN_E‑XPS_ value for that element (Figure 2, Tables 3–6, Supporting Information Tables S52–S54). For example, the strongest O_anion_ donor atoms are in [(CH_3_O)_2_PO_2_]^−^ and [CH_3_CO_2_]^−^, with DN_O‑XPS_ of 2.63 and 2.64 eV respectively (Figure 2, Figure 3 top axis, Table 3 and Supporting Information Figure S56). Given our choice here of setting DN_E‑XPS_ = 0 eV for the weakest 1° donor atom, negative values of DN_E‑XPS_ only occur for 2°/3° donor atoms; the weakest donor atoms have the largest negative DN_E‑XPS_ value for that element. In the future, negative values of DN_E‑XPS_ are likely to be found for very weakly interacting 1° donor atoms once more anions are studied using this method. For example, DN_O‑XPS_ = −0.56 eV is the smallest value for oxygen, for the BO oxygen 2° donor atoms in [B­(C_2_O_4_)_2_]^−^ (structure in Figure 1, DN_O‑XPS_ in Table 3, top axis on Figure 3 and Supporting Information Figure S56).

**Table 4:** Experimental (Nonitalicized Entries) And Calculated (Italicized Entries, Taken from Table S36) *E*_B_(N_anion_ 1s) and Nitrogen-Specific Donor Numbers (DN_N‑XPS_) for Ionic Liquid Samples^a^

anion	moiety	1°/2°/3°	*E*_B_(N_anion_ 1s)/eV	DN_N‑XPS_/eV
*[(CH)*_*2*_ *N*_*2*_ *CH]* ^*–*^	*N*	*Strongest 1°*	*402.49 ± 0.20*	*2.11 ± 0.20*
*[C*_*6*_ *H*_*4*_ *N*_*2*_ *CH]* ^*–*^	*N*	*1°*	*402.64 ± 0.20*	*1.96 ± 0.20*
[SCN]^−^	N	1°	402.66 ± 0.10	1.94 ± 0.10
[N(CN)_2_]^−^	N_terminal_	1°	403.21 ± 0.10	1.39 ± 0.10
[C(CN)_3_]^−^	N	1°	403.69 ± 0.10	0.91 ± 0.10
*[CF*_*3*_ *(C*_*3*_ *N*_*2*_ *)(CN)*_*2*_ *]* ^*–*^	*N*_*ring*_	*1°*	*404.18 ± 0.20*	*0.42 ± 0.20*
*[CF*_*3*_ *(C*_*3*_ *N*_*2*_ *)(CN)*_*2*_ *]* ^*–*^	*N*_*CN*_	*1°*	*404.24 ± 0.20*	*0.36 ± 0.20*
[B(CN)_4_]^−^	N	Weakest 1°	404.60 ± 0.10	0.00 ± 0.10
[(CF_3_SO_2_)_2_N]^−^	N	Strongest 2°	404.32 ± 0.10	0.28 ± 0.10
[(C_2_F_5_SO_2_)_2_N]^−^	N	2°	404.32 ± 0.10	0.28 ± 0.10
[N(CN)_2_]^−^	N_bridge_	2°	404.50 ± 0.10	0.10 ± 0.10
[(FSO_2_)_2_N]^−^	N	Weakest 2°	404.64 ± 0.10	–0.04 ± 0.10

a Experimental uncertainties are ±0.10 eV (Supporting Information section 14.2), and calculated uncertainties are ±0.20 eV (Supporting Information section 14.4).

**Table 5:** Experimental (Nonitalicized Entries) and Calculated (Italicized Entries, Taken from Table S37) *E*_B_(F_anion_ 1s) and Fluorine-Specific Donor Numbers (DN_F‑XPS_) for Ionic Liquid Samples^a^

anion	moiety	1°/2°/3°	*E*_B_(F_anion_ 1s)/eV	DN_F‑XPS_/eV
[BF_4_]^−^	F	Strongest 1°	690.76 ± 0.10	0.83 ± 0.10
[SbF_6_]^−^	F	1°	691.05 ± 0.15	0.54 ± 0.15
[(C_2_F_5_)_3_PF_3_]^−^	F_PF_	1°	691.58 ± 0.10	0.01 ± 0.10
[PF_6_]^−^	F	Weakest 1°	691.59 ± 0.10	0.00 ± 0.10
[(FSO_2_)_2_N]^−^	F	Strongest 2°/3°	692.66 ± 0.10	–1.07 ± 0.10
[CF_3_CO_2_]^−^	F	2°	693.03 ± 0.10	–1.44 ± 0.10
*[CF*_*3*_ *(C*_*3*_ *N*_*2*_ *)(CN)*_*2*_ *]* ^*–*^	*F*	*2°/3°*	*693.14 ± 0.20*	*–1.55 ± 0.20*
[CF_3_SO_3_]^−^	F	2°	693.23 ± 0.10	–1.64 ± 0.10
[(C_2_F_5_)_3_PF_3_]^−^	F_CF_	2°	693.23 ± 0.10	–1.64 ± 0.10
[(CF_3_SO_2_)_2_N]^−^	F	2°/3°	693.77 ± 0.10	–2.18 ± 0.10
[(C_2_F_5_SO_2_)_2_N]^−^	F	Weakest 2°/3°	693.86 ± 0.10	–2.27 ± 0.10

a Experimental uncertainties are ±0.10 eV (apart from the [SbF_6_]^−^ anion with ±0.15 eV, Supporting Information section 14.2), and calculated uncertainties are ±0.20 eV (Supporting Information section 14.4).

**Table 6:** Experimental (Nonitalicized Entries) And Calculated (Italicized Entries, Taken from Table S38) *E*_B_(Cl_anion_ 2p_3/2_) and Chlorine-Specific Donor Numbers (DN_Cl‑XPS_) for Ionic Liquid Samples^a^

anion	moiety	1°/2°/3°	*E*_B_(Cl_anion_ 2p_3/2_)/eV	DN_Cl‑XPS_/eV
Cl^–^	Cl	Strongest 1°	201.84 ± 0.10	2.55 ± 0.10
[LiCl_3_]^2–^	Cl	1°	202.42 ± 0.20	1.97 ± 0.20
[ZnCl_4_]^2–^	Cl	1°	203.01 ± 0.10	1.38 ± 0.10
[NiCl_4_]^2–^	Cl	1°	203.01 ± 0.10	1.38 ± 0.10
[CoCl_4_]^2–^	Cl	1°	203.03 ± 0.10	1.36 ± 0.10
[FeCl_4_]^2–^	Cl	1°	203.05 ± 0.10	1.34 ± 0.10
[Bi_2_Cl_8_]^2–^	Cl	1°	203.32 ± 0.10	1.07 ± 0.10
[SnCl_3_]^−^	Cl	1°	203.43 ± 0.10	0.96 ± 0.10
[Zn_2_Cl_6_]^2–^	Cl_terminal_	1°	203.50 ± 0.15	0.89 ± 0.15
[Zn_3_Cl_8_]^2–^	Cl_terminal_	1°	203.65 ± 0.15	0.74 ± 0.15
[Zn_4_Cl_10_]^2–^	Cl_terminal_	1°	203.72 ± 0.15	0.67 ± 0.15
[InCl_4_]^−^	Cl	1°	204.23 ± 0.10	0.16 ± 0.10
[FeCl_4_]^−^	Cl	1°	204.25 ± 0.15	0.14 ± 0.15
*[AlCl*_4_ *]* ^*–*^	*Cl*	*1°*	*204.27 ± 0.20*	*0.12 ± 0.20*
*[GaCl*_4_ *]* ^*–*^	*Cl*	*Weakest 1°*	*204.39 ± 0.20*	*0.00 ± 0.20*
[Zn_2_Cl_6_]^2–^	Cl_bridge_	Strongest 2°	204.20 ± 0.15	0.19 ± 0.15
[Zn_3_Cl_8_]^2–^	Cl_bridge_	2°	204.51 ± 0.15	–0.12 ± 0.15
[Zn_4_Cl_10_]^2–^	Cl_bridge_	Weakest 2°	204.66 ± 0.15	–0.27 ± 0.15

a Experimental uncertainties are ±0.10 eV (apart from the oligomeric chlorozincate and [FeCl_4_]^−^ anions with ±0.15 eV, [LiCl_3_]^−^ anion with ±0.20 eV, Supporting Information section 14.2), and calculated uncertainties are ±0.20 eV (Supporting Information section 14.4).

We chose to use ionic liquids here as the principal solvent environment for producing DN_E‑XPS_ (rather than molecular solvents, e.g., see section 2.2) for three major reasons. First, *E*_B_(O_anion_ 1s) can be measured for all O-containing anions in ionic liquids. For many molecular solvents (e.g., water, PC, TEP), the O_solvent_ 1s contribution obscures the O_anion_ 1s contribution (e.g., O 1s graphs for 18 samples in Supporting Information sections 11–13), meaning measurement of *E*_B_(O_anion_ 1s) in most molecular solvents is very challenging and potentially impossible, certainly at low anion concentration. Second, the existing *E*_B_(core,anion) literature data sets for ionic liquids can be combined with new data measured here;^48,73,82–95^ ionic liquids can be readily studied using standard XPS apparatus due to their very low vapor pressures.^107–109^ Third, the anion makes up half the solution (or one-third for 2– anions), so solubility/contaminant issues that occur for the current probe-dependent anion descriptors (e.g., any colored contaminant for UV–vis spectroscopy) are far less of a problem for DN_E‑XPS_ from ionic liquids.

### 2.2 Anion Element-Specific Donor Numbers: Intrinsic Measures of Anion Donor Abilities

Intrinsic anion donor abilities are captured by *E*_B_(core,anion) and therefore DN_E‑XPS_, with excellent independence from the solvation environment and spectroscopic contributions. Intra-anion covalent bonding contributions dominate over noncovalent solvation effects (anion-molecule or anion-countercation) and different anion solvation at the liquid–gas surface (see Supporting Information section 32). The intrinsic nature of DN_E‑XPS_ is reinforced by the calculation results presented in section 2.3. In the field of Lewis acids and Lewis bases, it has been assumed that intrinsic Lewis acidity or intrinsic Lewis basicity can only be captured using calculations;^110,111^ we show that intrinsic anion donor ability (very closely related to intrinsic anion Lewis basicity) can be measured using experiments.

There are excellent visual matches of both the *E*_B_(core,anion) order and Δ*E*_B_(core,anion) for anions in water, ionic liquid, PC and TEP (Figure 4 for F_anion_ 1s and Figure 5 for N_anion_ 1s). For example, the *E*_B_(N_anion_ 1s) order in all four solvents is [(FSO_2_)_2_ **N**]^−^ > [(CF_3_SO_2_)_2_ **N**]^−^ > [SC**N**]^−^ (Figure 5); *E*_B_(N_anion_ 1s) for both types of donor nitrogen atom in [N­(CN)_2_]^−^ also appear in the same order for water and ionic liquid (Figure 5). Furthermore, there are excellent linear correlations (*R* ^2^ very near 1 and gradients close to 1) for *E*_B_(F_anion_ 1s) in different solvents, e.g., ionic liquid versus water (Figure 6a), and also *E*_B_(N_anion_ 1s) in different solvents, e.g., ionic liquid versus water (Figure 6c).

**Figure 4:**
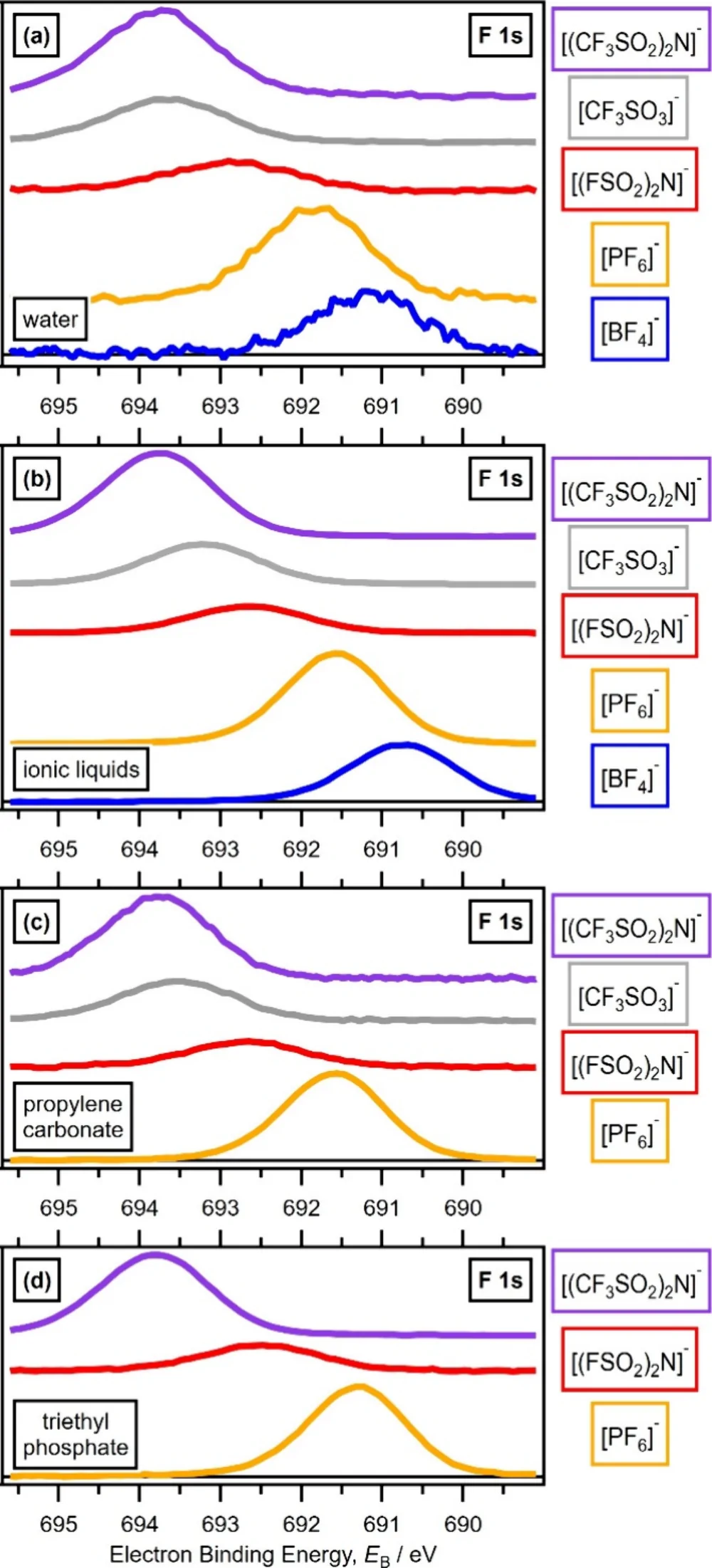
Comparison of fluorine-containing anions in different solvation environments. (a) F 1s XPS of fluorine-containing anions in water: 0.50 M Li­[(CF_3_SO_2_)_2_N], 0.40 M Na­[CF_3_SO_3_], 0.40 M Na­[(FSO_2_)_2_N], 0.50 M Li­[PF_6_], 0.50 M [C_4_C_1_Im]­[BF_4_]. (b) F 1s XPS of fluorine-containing anions in ionic liquid: [C_8_C_1_Im]­[(CF_3_SO_2_)_2_N], [C_8_C_1_Im]­[CF_3_SO_3_], [C_8_C_1_Im]­[(FSO_2_)_2_N], [C_8_C_1_Im]­[PF_6_], [C_8_C_1_Im]­[BF_4_]. (c) F 1s XPS of fluorine-containing anions in propylene carbonate: 0.14 M Li­[(CF_3_SO_2_)_2_N], 0.15 M Na­[CF_3_SO_3_], 0.30 M Na­[(FSO_2_)_2_N], 0.50 M Li­[PF_6_]. (d) F 1s XPS of fluorine-containing anions in triethyl phosphate: 0.48 M Li­[(CF_3_SO_2_)_2_N], 0.50 M Na­[(FSO_2_)_2_N], 0.50 M Li­[PF_6_]. Traces are vertically offset for clarity. Area normalization is to the number of atoms in each anion and explained further in Supporting Information section 8.3. Charge referencing: water *E*_B_(O_liquid_ 1s) = 538.13 eV; ionic liquid *E*_B_(C_alkyl_ 1s) = 289.85 eV; propylene carbonate *E*_B_(C_carbonyl_ 1s) = 295.80 eV; triethyl phosphate *E*_B_(C_alkyl_ 1s) = 290.32 eV (see Supporting Information section 8.1 for more details).

**Figure 5:**
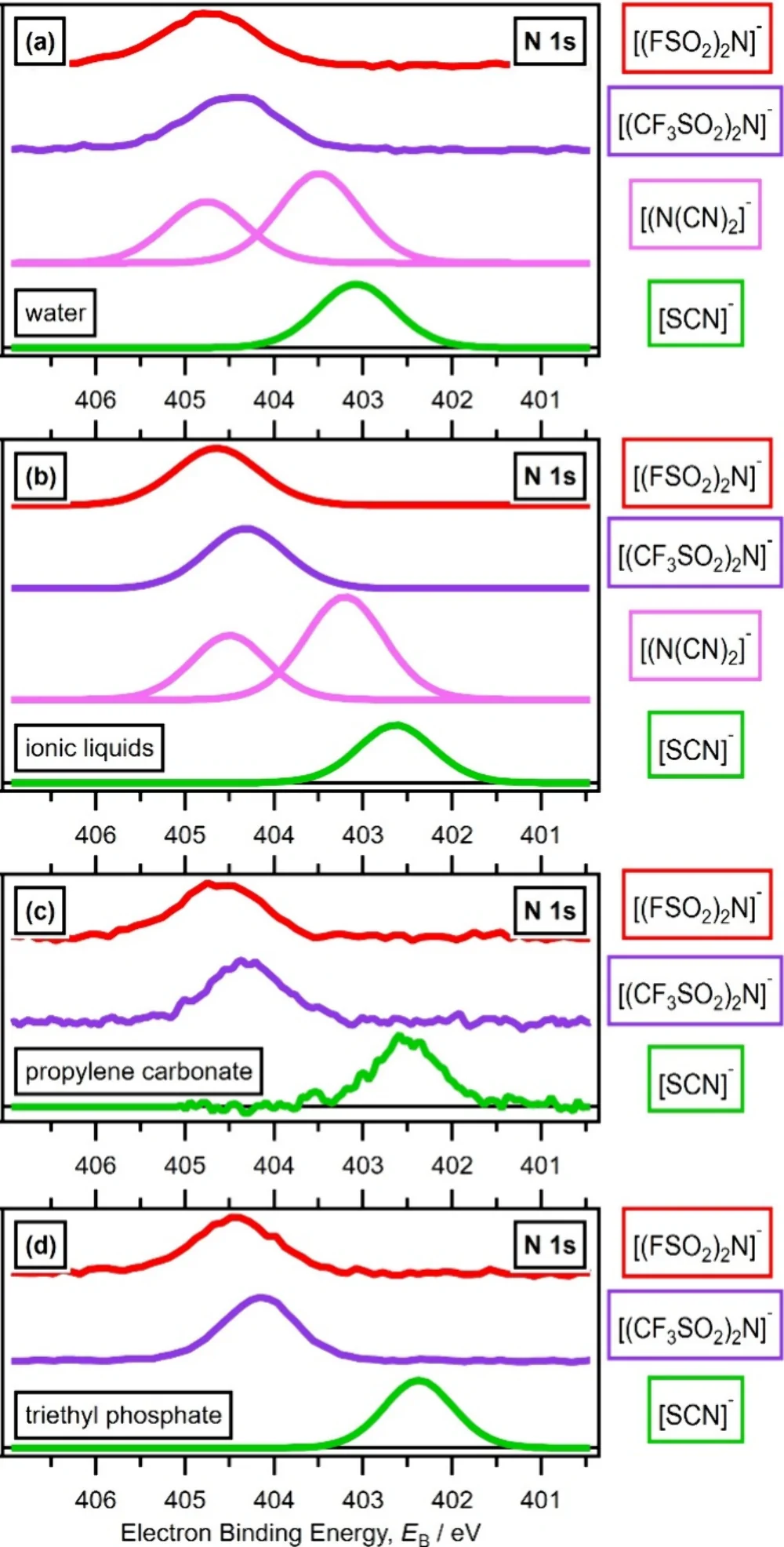
Comparison of nitrogen-containing anions in different solvation environments. (a) N 1s XPS of nitrogen-containing anions in water: 0.40 M Na­[(FSO_2_)_2_N], 0.50 M Li­[(CF_3_SO_2_)_2_N], 0.50 M [C_4_C_1_Im]­[N­(CN)_2_], 0.50 M [C_4_C_1_Im]­[SCN] (for the last two samples, only the anion fitted components are included; the cation fitted components are not included for clarity). (b) N 1s XPS of nitrogen-containing anions in ionic liquid: [C_8_C_1_Im]­[(FSO_2_)_2_N], [C_8_C_1_Im]­[(CF_3_SO_2_)_2_N], [C_4_C_1_Im]­[N­(CN)_2_], [C_8_C_1_Im]­[SCN] (for all four samples, only the anion fitted components are included; the cation fitted components are not included for clarity). (c) N 1s XPS of nitrogen-containing anions in propylene carbonate: 0.30 M Na­[(FSO_2_)_2_N], 0.14 M Li­[(CF_3_SO_2_)_2_N], 0.50 M K­[SCN]. (d) N 1s XPS of nitrogen-containing anions in triethyl phosphate: 0.50 M Na­[(FSO_2_)_2_N], 0.48 M Li­[(CF_3_SO_2_)_2_N], 0.18 M [C_4_C_1_Im]­[SCN] (for the last sample, only the anion fitted component is included; the cation fitted component is not included for clarity). Traces are vertically offset for clarity. Area normalization is to the number of atoms in each anion and explained further in Supporting Information section 8.3. Charge referencing: water *E*_B_(O_liquid_ 1s) = 538.13 eV; ionic liquid *E*_B_(C_alkyl_ 1s) = 289.85 eV (and *E*_B_(N_cation_ 1s) = 406.85 eV for [C_4_C_1_Im]­[N­(CN)_2_]); propylene carbonate *E*_B_(C_carbonyl_ 1s) = 295.80 eV; triethyl phosphate *E*_B_(C_alkyl_ 1s) = 290.32 eV (see Supporting Information section 8.1 and section 8.2 for more details).

**Figure 6:**
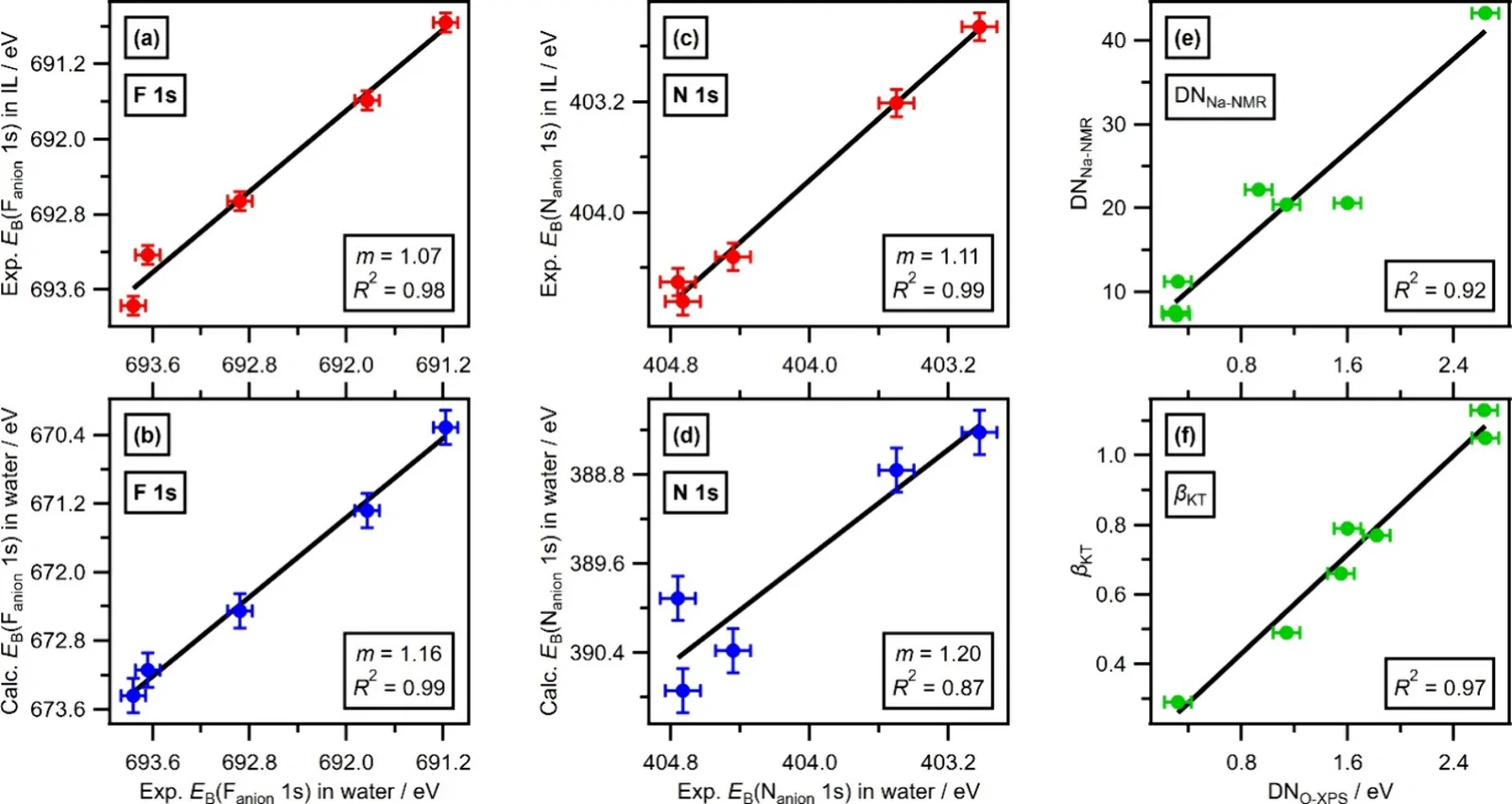
Linear correlations. (a) Experimental *E*_B_(F_anion_ 1s) for ionic liquid versus experimental *E*_B_(F_anion_ 1s) for water. (b) Calculated *E*_B_(F_anion_ 1s) (using lone-anion-SMD for water) versus experimental *E*_B_(F_anion_ 1s) in water. (c) Experimental *E*_B_(N_anion_ 1s) for ionic liquid versus experimental *E*_B_(N_anion_ 1s) for water. (d) Calculated *E*_B_(N_anion_ 1s) (using lone-anion-SMD for water) versus experimental *E*_B_(N_anion_ 1s) in water. Values used for the linear correlations are given in Table 4, Table 5, Supporting Information Table S39, and Supporting Information Table S40; linear fitting data are given in Supporting Information Table S62. Measures of anion donor ability plotted against DN_O‑XPS_ of [C_*n*_C_1_Im]­[A] neat ionic liquids: (e) anion donor numbers measured from the chemical shift δ­(Na^+^) by ^23^Na NMR spectroscopy of Na­[ClO_4_] dissolved in [C_4_C_1_Im]­[A] neat ionic liquids, DN_Na‑NMR_;^44^ (f) Kamlet–Taft hydrogen-bond acceptor numbers using UV–vis spectroscopy comparisons of two different neutral dye molecules in [C_4_C_1_Im]­[A] neat ionic liquids, β_KT_.^51^ Six other linear correlations between anion donor ability plotted and DN_O‑XPS_ of [C_*n*_C_1_Im]­[A] neat ionic liquids are given in Supporting Information Figure S71. Values used for the linear correlations are given in Supporting Information Table S63; linear fitting data are given in Supporting Information Table S64. The experimental uncertainty in DN_O‑XPS_ is ±0.10 eV (unless stated otherwise in Supporting Information section 14.2) and calculated uncertainty in DN_O‑XPS_ is ±0.20 eV (Supporting Information section 14.4).

We have high confidence that any solvent, beyond those studied here, will not have a strong effect on Δ*E*_B_(core,anion) and therefore ΔDN_E‑XPS_. The four different solvents studied here give an excellent range of solvent acceptor numbers, i.e., the abilities of the solvents studied here to interact with the anion are not the same. Water is a strong electron acceptor; PC, TEP and most ionic liquids are midrange to weak electron acceptors.^102^

Countercation effects on *E*_B_(core,anion) and therefore DN_E‑XPS_ in molecular solvents, i.e., changing the cation and keeping the anion [A]^−^ the same, are within the experimental uncertainty at relatively low salt concentration (≤1 M). We have substantial data for same anion-different countercations samples for all three molecular solvents studied here (Supporting Information Figures S63–S67). The countercations studied have very different interaction properties/strengths: large, organic [C_4_C_1_Im]^+^ versus small inorganic M^+^ (Li^+^, Na^+^, K^+^) versus small inorganic M^2+^ (Mg^2+^, Ca^2+^, Zn^2+^). For example, [cation]­[CF_3_SO_3_] in water was studied with six different countercations (Na^+^, K^+^, Mg^2+^, Ca^2+^, Zn^2+^, [C_4_C_1_Im]^+^, Supporting Information Figure S64); [cation]­[SCN] in PC with two different countercations (K^+^, [C_4_C_1_Im]^+^, Supporting Information Figure S65); [cation]­[(CF_3_SO_2_)_2_N] in TEP with two different countercations (Li^+^, Na^+^, Supporting Information Figure S67). At higher salt concentrations, e.g., 8 M, countercation effects are likely to become important.

For ionic liquids, the effect of the organic dialkylimidazolium countercation, [C_*n*_C_1_Im]^+^, with different *n* values (i.e., different alkyl chain substituent lengths) on *E*_B_(core,anion) and therefore DN_E‑XPS_ is within the experimental uncertainty.^73,84^ In contrast, the alkyl chain substituent length *n* had an impact on anion DN_Na‑NMR_ measured using ^23^Na NMR spectroscopy in different [C_*n*_C_1_Im]­[A] ionic liquids. This literature finding is perhaps surprising given that the [C_*n*_C_1_Im]^+^ cations are not directly solvating the Na^+^ cation, i.e., Na^+^ will have anions in the first solvation shell and [C_*n*_C_1_Im]^+^ cations in the second solvation shell. The differing countercation dependencies for DN_E‑XPS_ versus DN_Na‑NMR_ may be due to the spectroscopic time scales of core-level XPS and NMR spectroscopy; nuclear motion could be important for NMR spectroscopy, whereas photoemission in XPS is sufficiently fast (∼10^–15^ s) that nuclear motion during the spectroscopic event will be negligible.

Our intrinsic DN_O‑XPS_ for 1° oxygen atoms show excellent matches to (single-value) literature anion-level descriptors, demonstrating that, despite being measured using probes, the anion-level descriptors do a good job of capturing intrinsic anion donor ability. DN_O‑XPS_ give excellent/good linear correlations with eight different anion-level DN/β descriptors (range of *R* ^2^ 0.97 to 0.70, Figure 6e and Figure 6f for two comparisons, Supporting Information Figure S71 for all eight comparisons).^44,46–51,53,112^ For example, excellent linear correlations are found between DN_O‑XPS_ and both anion DNs (e.g., DN_Na‑NMR_,^44^ Figure 6e) and anion β (e.g., β_KT_,^51^ Figure 6f). These eight anion-level descriptors include a range of probes (metal cations, organic cations and molecules), giving high confidence that our 1° DN_O‑XPS_ and anion-level descriptors both capture donor ability. There is clearly some influence of the countercation on probe-dependent, anion-level DNs and β (see section 2.2), but when the same cation is used anion-level probe-dependent descriptors are primarily driven by intrinsic anion donor properties. For both *E*_B_(N_anion_ 1s) and *E*_B_(F_anion_ 1s), there is insufficient data for meaningful linear correlations to be obtained (see Supporting Information section 33 for more comment on this area).

By comparing probe-independent DN_E‑XPS_ against probe-dependent descriptors, there is an opportunity to learn more about probe-specific interactions. Differences in experimental DN_E‑XPS_ are driven by anion electronic properties, with minimal contributions from specific anion-probe binding or steric hindrance. In contrast, for the eight different anion-level DN/β descriptors specific anion-probe binding interactions provide an unknown contribution that is potentially probe-dependent and therefore anion-dependent. For a linear correlation between our DN_E‑XPS_ and an anion-level descriptor, any anion data point outside the experimental uncertainty could indicate a different anion-probe binding mechanism for that anion compared to the other anions studied (e.g., nitrogen dominated binding for [(CF_3_SO_2_)_2_N]^−^) or highlight the importance of steric hindrance. However, the lack of experimental uncertainty provision for most existing anion descriptors makes this approach challenging, and we will not pursue any such analysis here.

### 2.3 Lone-Ion-SMD Calculations Can Accurately and Efficiently Predict Anion Element-Specific Donor Numbers

The goal of *E*_B_(core,anion) and therefore DN_E‑XPS_ prediction with high accuracy and low computational cost is achieved. Calculated DN_E‑XPS_ can be used with high confidence in different solvent environments (ionic liquids, water, PC, and TEP), e.g., DN_O‑XPS_ calculated in an ionic liquid SMD for [B­(C_2_O_4_)_2_]^−^ can be directly compared to DN_O‑XPS_ measured using ionic liquid experimental XPS (Figure 3 and Table 3). This greatly reduces the need to produce DN_E‑XPS_ from expensive synthesis followed by challenging liquid-phase XPS experiments; the high-vacuum environment required for XPS of ionic liquids and the instability of certain anions under X-ray irradiation, and particularly the need for very challenging and rare synchrotron-based liquid-jet XPS apparatus to study anions in molecular solutions.

For ionic liquids, a high-accuracy and low-cost computational method to predict *E*_B_(core,anion) is already available: lone-anion calculations with a solvation model based on density (SMD), called lone-anion-SMD.^49,73,92,101,113,114^ The core-levels included were O 1s, N 1s, F 1s, Cl 2p, and S 2p (and also C 1s and P 2p). To predict *E*_B_(core,anion) and DN_E‑XPS_ in ionic liquids that are comparable to experimental DN_E‑XPS_, a single *E*_B_(correction) is required for the core-level of interest, as long as the same basis set, functional, and SMD parameters are used. The *E*_B_ correction for each core-level for ionic liquids is given in Supporting Information Table S11, e.g., +20.69 eV for calculations of *E*_B_(F_anion_ 1s); furthermore, eqs S2–S5 can be used to convert uncorrected calculated *E*_B_(core,anion) into DN_E‑XPS_.

*E*_B_(core,anion) and therefore DN_E‑XPS_ can be predicted with high accuracy and low computational cost in molecular solvents for different anions. There are excellent visual matches of experiments and lone-anion-SMD calculations for common anions in water, PC and TEP; both the *E*_B_(core,anion) order and Δ*E*_B_(core,anion) (Figure 7 for water, Supporting Information Figure S68 for PC and TEP). Furthermore, there are excellent linear correlations for calculated *E*_B_(core,anion) against experimental *E*_B_(core,anion); Figure 6b for *E*_B_(F_anion_ 1s) and Figure 6d for *E*_B_(N_anion_ 1s) for water, Supporting Information Figure S69b,d for PC, Supporting Information Figure S70b,d for TEP. These linear correlations give R^2^ near 1 and gradients close to 1. As for calculations of anions in ionic liquid SMD, calculations of anions in water SMD, PC SMD or TEP SMD can accurately and efficiently predict *E*_B_(core,anion) and therefore DN_E‑XPS_ using a single *E*_B_(correction) for each core-level; the *E*_B_ corrections for water are given in Supporting Information Table S51, e.g., +20.53 eV for calculations of *E*_B_(F_anion_ 1s).

**Figure 7:**
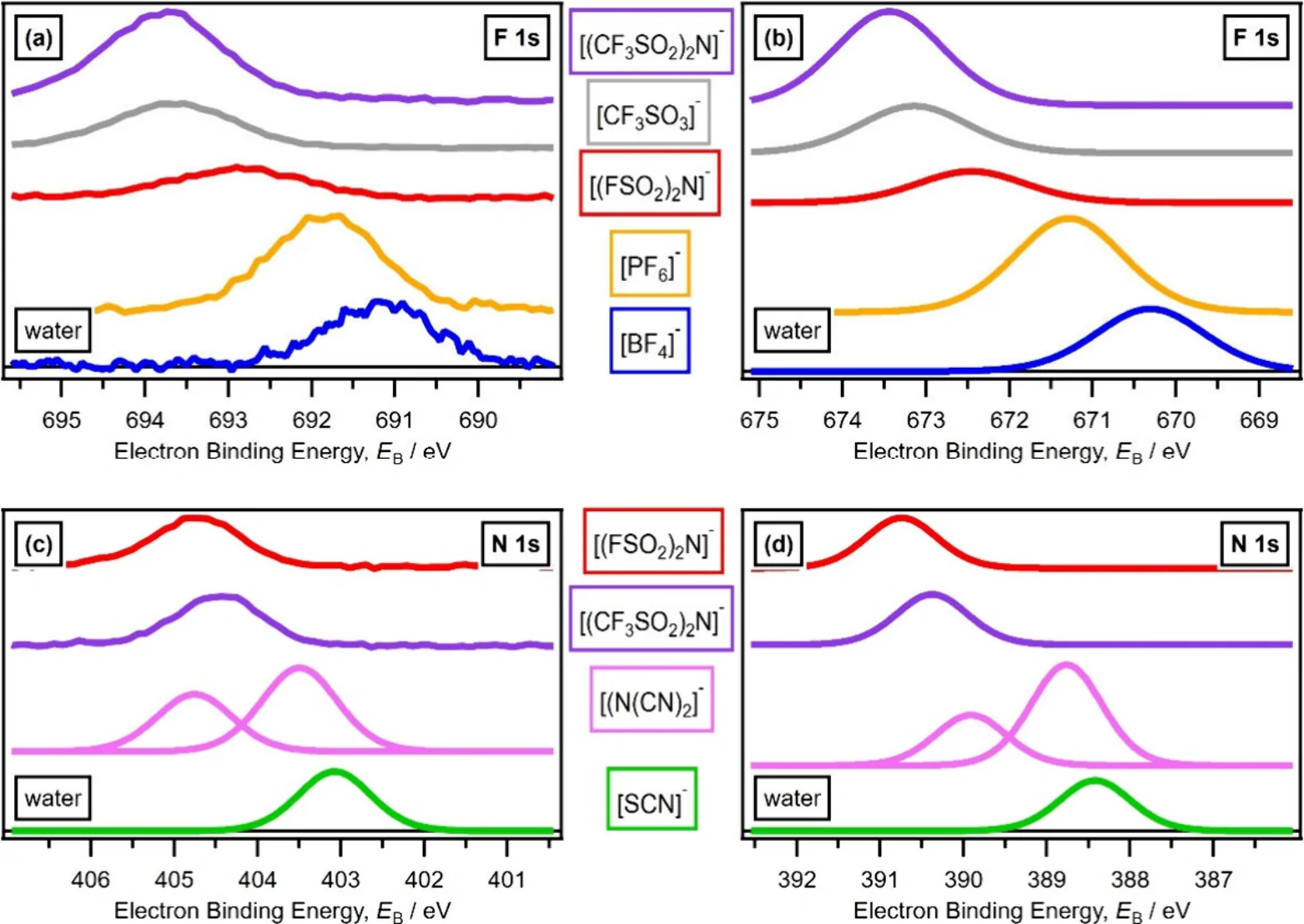
Experimental and calculated XPS spectra for anions. F 1s XP spectra for five different anions: (a) experimental in water (same samples as in Figure 4a,b calculated using lone-anion-SMD for water). N 1s XP spectra for four different anions: (c) experimental in water (same samples as in Figure 5a,d calculated using lone-anion-SMD for water). Traces are vertically offset for clarity. Experimental XPS area normalization is to the number of atoms in each anion and explained further in Supporting Information section 8.1. Calculated XP spectra are produced as explained in Supporting Information section 6. Charge referencing for experimental XP spectra in water *E*_B_(O_liquid_ 1s) = 538.13 eV.

There is great potential for DN_E‑XPS_ as reliable anion descriptors for data-driven discovery, with major advantages over current anion descriptors. First, our DN_E‑XPS_ can be calculated for far more anions than the current experimentally measured (and not cheaply calculated) anion descriptors. Second, calculated DN_E‑XPS_ can be used for fine-tuning anion donor properties (e.g., controlling anion local donor ability by varying R groups on anion families such as [(RSO_2_)_2_N]^−^,^115^ oxyborate,^27^ dicyanoimidazolide^116^). Third, our DN_E‑XPS_ descriptors have the chance to capture the complexity of interactions for anions in the liquid-phase, especially when solvation will be strongly directional/inhomogeneous, e.g., large and complex anions solvating metal cations; our DN_E‑XPS_ descriptors have the potential to give insight into different anion denticities, unlike current anion-level DN/β descriptors. Fourth, our DN_E‑XPS_ descriptors have great potential as ML training data sets, given that DN_E‑XPS_ can be calculated with high accuracy and low computational cost. Electronic descriptors for molecular solvents have been used as training data for ML,^63^ so there is potential for anion descriptors in ML.

The ability of calculated DN_E‑XPS_ to capture the relative donor strength of atoms with different covalent bonding in the same anion is further validated by comparison to interactions in crystal structures. For [CF_3_(C_3_N_2_)­(CN)_2_]^−^ (also known as [TDI]^−^, structure in Figure 1) N_CN_ and N_ring_ have the same DN_N‑XPS_ within calculated uncertainty (Table 4, Supporting Information Figure S57) – we have labeled both N_CN_ and N_ring_ as 1° in Figure 1 and Table 4. This finding matches to crystal structures of [CF_3_(C_3_N_2_)­(CN)_2_]^−^ with Li^+^, where the shortest Li^+^–N_CN_ and Li^+^–N_ring_ distances are all within 0.1 Å.^117^ For oxygen in [B­(C_2_O_4_)_2_]^−^ (structure in Figure 1), O_BO_ and O_CO_ bonding environments can be readily distinguished in the calculated O 1s XP spectrum (Figure 3, Table 3, structure in Figure 1), with DN_O‑XPS_ larger for O_CO_ than O_BO_. This finding matches to crystal structures of [B­(C_2_O_4_)_2_]^−^ with Li^+^, where the shortest Li^+^-O_CO_ distances are ∼1.1 Å shorter than the shortest Li^+^-O_BO_ distances.^118^

The intrinsic nature of DN_E‑XPS_ demonstrated in section 2.2 is reinforced by the calculation results presented here in section 2.3. There are no explicit countercations or solvent molecules in lone-anion-SMD calculations, demonstrating that our *E*_B_(core,anion) captures intrinsic differences in anion properties.

### 2.4 Anion Element-Specific Donor Numbers: Excellent Interpretability

Ground state bonding effects (called initial-state effects in the XPS community) dominate Δ*E*_B_(core,anion), and therefore DN_E‑XPS_, measured in the same solvent, as conclusively demonstrated by the linear correlations between experimental and calculated *E*_B_(core,anion) for all solvents studied here (see section 2.3 and references 49,73,92,101,113,114 ). The influence of the core-hole created during photoemission (final-state effects) on Δ*E*_B_(core,anion) is minimal.

*E*_B_(core,anion), and therefore DN_E‑XPS_, is a measure of donor ability, based on a number of excellent correlations presented here between: (i) experimental *E*_B_(core,anion) for ionic liquids and experimental *E*_B_(core,anion) for molecular solvents (section 2.2); (ii) experimental *E*_B_(core,anion) and calculated *E*_B_(core,anion) for ionic liquids (section 2.3 and references 73,92,101 ); (iii) experimental *E*_B_(core,anion) and calculated *E*_B_(core,anion) for water, PC and TEP (section 2.3); (iv) experimental and calculated *E*_B_(core,anion) and calculated *V*_n_ for ionic liquids;^49,101,113^ (v) *V*_n_ and donor ability.^119–121^ We can also conclude with high confidence that ΔDN_E‑XPS_ will capture Δ*V*_n_(anion) and therefore anion donor ability for anions solvated by other solvents used for battery electrolytes (e.g., carbonate mixtures, acetonitrile), as the solvents we have studied cover an excellent range of solvent properties.

Our results are excellent experimental evidence that the calculated ESP, closely related to *V*_n_, can be used to judge the local donor ability. We are unaware of any study using calculated ESP to help select anions for batteries. However, calculated ESP descriptors are a recent and growing area in the battery community, to identify which part of the molecule has good electron donor ability,^63,64^ and capturing oxygen atom donor strength.^68^

We make a recommendation here with regard to placing the location of the negative charge on a chemical structure of an anion. Many researchers may have given this point little thought. However, such placement can be misleading, e.g., placing the negative charge near the central N_CNC_ in [N­(CN)_2_]^−^, near N_ring_ in [CF_3_(C_3_N_2_)­(CN)_2_]^−^ (structure in Figure 1). Hence, in this article, all negative charge symbols are represented over the whole anion in each case (e.g., Figure 1), and we recommend this as a general approach for all anion chemical structures to avoid misinterpretation.

There is a trade-off when using DN_E‑XPS_ compared to existing experimental (single value and probe-dependent) anion descriptors; significant atomic-level insight is gained, but overall comparability across all anions is decreased. DN_E‑XPS_ provide more than one descriptor for many anions and can be applied to all anions; one just needs two (or more) anions containing a particular element to construct a DN_E‑XPS_ descriptor. However, a single DN_E‑XPS_ descriptor is not presented here that covers all anions; not all anions contain, for example, oxygen. In the future, we will attempt to produce a single descriptor that brings together DN_E‑XPS_ for the different elements. We know that Δ*E*_B_(O_anion_ 1s) = 1 eV and Δ*E*_B_(F_anion_ 1s) = 1 eV correspond to Δ*V*_O_ ≈ 1 eV and Δ*V*_F_ ≈ 1 eV respectively. The question then arises: Does Δ*V*_O_ = 1 eV represent the same change in donor ability as Δ*V*_F_ = 1 eV? From our literature searches it appears that *V*_n_ values are only ever compared for the same element,^119–121^ so this question has not yet been rigorously answered, suggesting that obtaining a single anion descriptor from *E*_B_(core,anion) is still some way off.

### 2.5 Fine-Tuning DN_E‑XPS_ for [(RSO_2_)_2_N]^−^: Implications for Battery Electrolyte Selection

The effect on [(RSO_2_)_2_N]^−^ donor ability of decreased fluorination from R = CF_3_ to R = F is relatively large; this can potentially impact battery electrolyte performance, e.g., in lithium-ion batteries. First, changing from R = CF_3_ to R = F can potentially improve battery stability due to increased chances of LiF formation at the SEI,^35,36^ caused by (a) much larger DN_F‑XPS_ (Figure 8a) and therefore stronger 3° Li^+^–F interactions; (b) smaller DN_N‑XPS_ (Figure 8b) and therefore weaker 2° Li^+^-N interactions. Second, changing from R = CF_3_ to R = F likely reduces the Li^+^-anion desolvation energy at the SEI through an improvement in this potentially rate-determining step in battery charging, caused by smaller DN_O‑XPS_ (Figure 8c) and therefore weaker 1° Li^+^–O interactions. Lastly, changing from R = CF_3_ to R = F will lead to weaker transition metal-N interactions from smaller DN_N‑XPS_ (Figure 8b) when metal dissolution from the cathode occurs,^122^ as N can become the 1° donor atom for [(RSO_2_)_2_N]^−^.^105^

**Figure 8:**
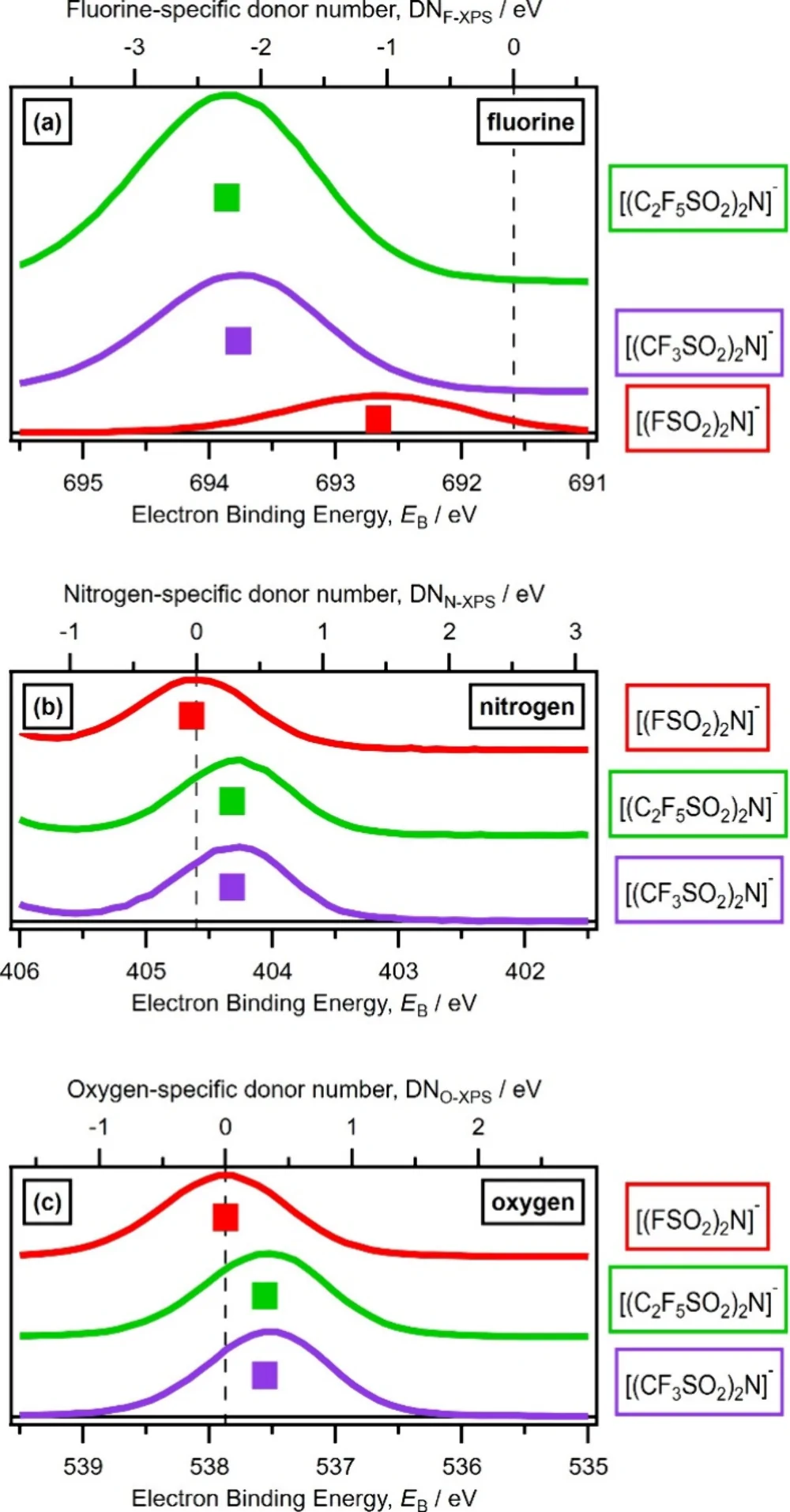
Values of *E*_B_(core,anion) and DN_E‑XPS_ (bottom and top axes, respectively) for the three ionic liquids [C_8_C_1_Im]­[(FSO_2_)_2_N], [C_4_C_1_Im]­[(C_2_F_5_SO_2_)_2_N], and [C_8_C_1_Im]­[(CF_3_SO_2_)_2_N]: (a) O 1s, (b) N 1s, (c) F 1s. Values are vertically offset for clarity. Charge referencing is given in Supporting Information section 8.2. For *E*_B_(core,anion) (and therefore DN_E‑XPS_), the experimental uncertainty is ±0.10 eV (Supporting Information section 14.2); these uncertainty values are represented by the width of each block.

The effect on the [(RSO_2_)_2_N]^−^ donor ability of increased fluorination from R = CF_3_ to R = C_2_F_5_ is minimal. Altering R = CF_3_ to R = C_2_F_5_ leads to no change within experimental uncertainty of DN_N‑XPS_ (Figure 8b) or DN_O‑XPS_ (Figure 8c). Therefore, changing from R = CF_3_ to C_2_F_5_ will provide little difference in Li^+^–O or Li^+^–N interaction strengths. There is a suggestion from DN_F‑XPS_ that increasing fluorination leads to slightly smaller DN_F‑XPS_ and therefore slightly weaker Li^+^-F interactions (experimental data in Figure 8a and calculated *E*_B_(F_anion_ 1s) in ref^73^); of course, increasing fluorination may also lead to an increase in the number of Li^+^–F interactions given the greater number of F atoms in the anion, so overall any effect is likely to be minimal.

## 3 Conclusions and Future Work

Robust and interpretable new element-specific donor number descriptors, DN_E‑XPS_, are defined and measured using a combination of experimental XPS and low-cost, simple calculations. New methods for producing DNs are rarely discovered; DN_E‑XPS_ greatly expands and enhances the small toolbox of existing DN descriptors.

DN_E‑XPS_ descriptors capture local (i.e., atomic level) atom donor abilities. We provide separate anion DN_E‑XPS_ for seven different elements (O, N, F, Cl, Br, I, S); each anion 1°, 2°, and 3° donor atom has its own DN_E‑XPS_ value. DN_E‑XPS_ can be used to evaluate anion-substrate interactions by 2° and 3° anion donor atoms, unlike anion-level descriptors, providing insight into rare reactivity events. For example, for battery electrolyte selection DN_O‑XPS_, DN_N‑XPS_ and DN_F‑XPS_ can be used to predict that [(FSO_2_)_2_N]^−^ will provide superior electrolyte performance over [(CF_3_SO_2_)_2_N]^−^ and [(C_2_F_5_SO_2_)_2_N]^−^ with regard to both anion–metal cation desolvation and LiF formation at the SEI.

DN_E‑XPS_ can be applied with confidence to a wide range of anion–substrate interactions, as DN_E‑XPS_ captures intrinsic anion donor ability, independent of specific probe cation/molecule interactions. Furthermore, there is the potential to gain molecular-level insight into anion interactions by comparing DN_E‑XPS_ against other descriptors; however, more experimental data are required for existing anion descriptors to make the most of this opportunity.

DN_E‑XPS_ can be used for data-driven discovery. The cheap and low-cost calculation method for DN_E‑XPS_ presented here, in conjunction with our substantial existing DN_E‑XPS_ database for anions, opens up the possibility of using DN_E‑XPS_ for both fine-tuning anion donor properties (e.g., controlling anion local donor ability by varying R groups on anion families such as [(RSO_2_)_2_N]^−^,^115^ oxyborate,^27^ dicyanoimidazolide^116^) and as anion descriptors for machine learning (ML) training data sets.

The development of a full picture of liquid-phase donor ability for both anions and molecules, which would fast-forward data-driven discovery, is a long-term goal. DN_E‑XPS_ scales including both anions and molecules (i.e., solvents/additives) for each element are needed, extending beyond the DN_E‑XPS_ scales for anions published here.

## 4 Methods

### 4.1 Samples Studied and Preparation

44 anions are studied (44 [C_*n*_C_1_Im]­[anion] ionic liquids in total) using a combination of literature and new data (Supporting Information Table S5); seven ionic liquids were measured on multiple apparatuses to demonstrate the small experimental uncertainty (Supporting Information section 14.2). All XPS data for anions in molecular solvents are new here: seven anions in water (15 samples, Supporting Information Table S6); five anions in PC (nine samples, Supporting Information Table S7); and four anions in TEP (five samples, Supporting Information Table S8).

Lone-anion SMD (ionic liquid) calculations were carried out for four new anions; results from these calculations are used in conjunction with previously published calculations for three anions from ref 123 (Table S9). Lone-anion SMD (water) calculations were carried out for nine new anions (Table S9).

### 4.2 Experimental Apparatus

#### 4.2.1 Ionic Liquid Drop XPS Apparatus

Laboratory-based XPS measurements for two ionic liquid samples were performed at the University of Reading on a Thermo Scientific ESCALAB 250 monochromated Al Kα source (*hν* = 1486.6 eV) spectrometer and for one ionic liquid sample was performed at University College London on a Thermo Scientific Theta Probe monochromated Al Kα source (*hν* = 1486.6 eV) spectrometer. A drop of ionic liquid was placed directly onto a stainless steel sample plate. XPS apparatus used for literature *E*_B_(core,anion) has already been described elsewhere.^48,49,73,86,91–95,124,125^

Synchrotron-based XPS measurements for nine ionic liquid samples were performed at beamline B07-B XPS.^114,126^ A drop of ionic liquid was placed directly onto a custom-made tantalum sample plate. Rastering of the sample was performed to minimize sample charging/damage.

#### 4.2.2 Liquid-Jet XPS Apparatus

Solutes were weighed and mixed with a corresponding mass of the solvent to achieve the desired concentration. A liquid-jet apparatus was used. The measurement for eight solutions was performed on the U49/2-PGM 1 beamline with SOL^3^PES end-station^127^ at the BESSY II electron storage ring (Germany). XP spectra were acquired using a Scienta Omicron R4000 HIPP-2 hemispherical electron analyzer. The measurement for 16 solutions was performed on the HIPPIE beamline^128^ at the MAX IV II electron storage ring (Sweden). XP spectra were acquired using the Solid–Liquid Endstation with a SPECS Phoibos 150 hemispherical electron analyzer. The measurement for five solutions was performed on the GALAXIES beamline^129^ at the Soleil electron storage ring (France). XP spectra were acquired by using an EW4000 hemispherical electron analyzer.

### 4.3 Calculations

All DFT calculations were carried out using Gaussian 16 (version C.01).^130^ Each of the anions was optimized under no symmetry constraints and confirmed as a minimum using vibrational analysis. DFT calculations were carried out at the B3LYP-D3­(BJ)/6-311+G­(d,p) level. The SMD (solvation model based on density) was employed to account for solvation effects. Specifically, the [C_4_C_1_Im]­[PF_6_] parameters (Supporting Information Table S10) were selected to be consistent with previous ionic liquid work.^92^ The SMD solvent continua for water, PC and TEP (Supporting Information Table S10) were also employed. Optimization convergence criteria were set to 10^–11^ on the density matrix and 10^–9^ on the energy matrix. Moreover, the numerical grid was improved from the default using the “int=ultrafine” keyword. Vibrational frequencies and zero-point vibrational energy corrections (ZPE) were attained using the harmonic approximation.

### 4.4 Data Analysis

#### 4.4.1 Component Fitting for Core-Level XPS

All experimental XP spectra were fitted using CASAXPS software.^131^ Fitting was carried out using a Shirley/linear background and GL30 line shapes (70% Gaussian, 30% Lorentzian). Peak constraints used are outlined in Supporting Information Tables S13–S16. Relative sensitivity factors (RSFs) from ref 132 were used for XPS recorded at *hν* = 1486.6 eV to ensure the experimental stoichiometries matched the nominal stoichiometries (Supporting Information Table S21, Supporting Information section 10).

#### 4.4.2 Charge Referencing

Each sample was internally charge referenced, effectively to the vacuum level. Each of the three molecular solvent systems studied here (water, PC, TEP) were charge referenced to a single solvent-specific core-level (Supporting Information section 8.1): water *E*_B_(O_liquid_ 1s) = 538.13 eV; propylene carbonate *E*_B_(C_carbonyl_ 1s) = 295.80 eV; triethyl phosphate *E*_B_(C_alkyl_ 1s) = 290.32 eV (Supporting Information Tables S17 to S19). For ionic liquids, we used an adaptation of the very well-established method for charge referencing ionic liquid XPS (Supporting Information Table S20).^49,73,84^ It is common practice for ionic liquid XPS to charge reference *E*_B_(core) effectively to the Fermi level using either *E*_B_(C_alkyl_ 1s) = 285.00 eV for ionic liquids with long alkyl chains or an anion-specific *E*_B_(N_cation_ 1s) value for ionic liquids with shorter alkyl chains.^125^ Here we charge reference ionic liquid XPS to the vacuum level to match the charge referencing level used for anions in molecular solvents by adding 4.85 eV to *E*_B_(core) values charge-referenced to the Fermi level (the 4.85 eV value can be thought of as a work function value). *E*_B_(core) values used for charge referencing ionic liquid XPS are given in Supporting Information section 8.2, e.g., *E*_B_(C_alkyl_ 1s) = 289.85 eV for [C_8_C_1_Im]­[anion] ionic liquids (Supporting Information Table S20).

## Supplementary Material



## Data Availability

The data underlying this study are openly available in the University of Bath Research Data Archive at https://doi.org/10.15125/BATH-01706.

## Notes

Methods (samples studied, calculation method, how to produce calculated and “experimental” XP spectra); data analysis (peak fitting, charge referencing, producing DN_E‑XPS_); results (demonstrating purity, uncertainty, *E*_B_(core,anion) values, effect of countercations on DN_E‑XPS_, linear correlations and bulk versus surface contributions) (PDF)

## References

[1] Huang Z., Li X., Chen Z., Li P., Ji X., Zhi C. (2023). Anion chemistry
in energy storage devices. Nat. Rev. Chem..

[2] MacFarlane D. R., Forsyth M., Howlett P. C., Kar M., Passerini S., Pringle J. M., Ohno H., Watanabe M., Yan F., Zheng W. J., Zhang S. G., Zhang J. (2016). Ionic liquids and their
solid-state analogues as materials for energy generation and storage. Nat. Rev. Mater..

[3] Wang Z. X., Sun Z. H., Li J., Shi Y., Sun C. G., An B. G., Cheng H. M., Li F. (2021). Insights into
the deposition
chemistry of Li ions in nonaqueous electrolyte for stable Li anodes. Chem. Soc. Rev..

[4] Holoubek J., Liu H. D., Wu Z. H., Yin Y. J., Xing X., Cai G. R., Yu S. C., Zhou H. Y., Pascal T. A., Chen Z., Liu P. (2021). Tailoring electrolyte solvation for
Li metal batteries cycled at ultra-low temperature. Nat. Energy.

[5] Lu Z., Geng C., Yang H., He P., Wu S., Yang Q., Zhou H. (2022). Step-by-step desolvation enables
high-rate and ultra-stable sodium storage in hard carbon anodes. Proc. Natl. Acad. Sci. U. S. A..

[6] Han J., Mariani A., Passerini S., Varzi A. (2023). A perspective on the
role of anions in highly concentrated aqueous electrolytes. Energy Environ. Sci..

[7] Xiao Y., Xu R., Xu L., Ding J. F., Huang J. Q. (2022). Recent advances
in anion-derived SEIs for fast- charging and stable lithium batteries. Energy Mater..

[8] Li T., Zhang X. Q., Yao N., Yao Y. X., Hou L. P., Chen X., Zhou M. Y., Huang J. Q., Zhang Q. (2021). Stable Anion-Derived
Solid Electrolyte Interphase in Lithium Metal Batteries. Angew. Chem., Int. Ed..

[9] Park G., Lee K., Yoo D. J., Choi J. W. (2022). Strategy for Stable Interface in
Lithium Metal Batteries: Free Solvent Derived vs Anion Derived. ACS Energy Lett..

[10] Schroder K. W., Dylla A. G., Bishop L. D. C., Kamilar E. R., Saunders J., Webb L. J., Stevenson K. J. (2015). Effects
of Solute-Solvent Hydrogen
Bonding on Nonaqueous Electrolyte Structure. J. Phys. Chem. Lett..

[11] Yamada Y., Usui K., Chiang C. H., Kikuchi K., Furukawa K., Yamada A. (2014). General Observation of Lithium Intercalation
into Graphite
in Ethylene-Carbonate-Free Superconcentrated Electrolytes. ACS Appl. Mater. Interfaces.

[12] Chen X., Zhang Q. (2020). Atomic Insights into
the Fundamental Interactions in Lithium Battery
Electrolytes. Acc. Chem. Res..

[13] Brandt A., Gräsvik J., Hallett J. P., Welton T. (2013). Deconstruction of lignocellulosic
biomass with ionic liquids. Green Chem..

[14] Du H., Chatti M., Hodgetts R., Cherepanov P., Nguyen C., Matuszek K., MacFarlane D., Simonov A. (2022). Electroreduction of nitrogen with almost 100% current-to-ammonia
efficiency. Nature.

[15] Estager J., Holbrey J. D., Swadźba-Kwaśny M. (2014). Halometallate
ionic liquids - revisited. Chem. Soc. Rev..

[16] Riddlestone I. M., Kraft A., Schaefer J., Krossing I. (2018). Taming the Cationic
Beast: Novel Developments in the Synthesis and Application of Weakly
Coordinating Anions. Angew. Chem., Int. Ed..

[17] Brak K., Jacobsen E. (2013). Asymmetric Ion-Pairing
Catalysis. Angew. Chem., Int. Ed..

[18] Ovian J., Vojácková P., Jacobsen E. (2023). Enantioselective transition-metal
catalysis via an anion-binding approach. Nature.

[19] Noh J., Kim H., Park H., Chung D. (2025). The Roles of Ions in Electrochemical
Interface for Electrocatalysis. ACS Catal..

[20] Yoo J., Ingenmey J., Salanne M., Lukatskaya M. (2024). Anion Effect
in Electrochemical CO2 Reduction: From Spectators to Orchestrators. J. Am. Chem. Soc..

[21] Jungwirth P., Cremer P. S. (2014). Beyond Hofmeister. Nat. Chem..

[22] Okur H., Hladílková J., Rembert K., Cho Y., Heyda J., Dzubiella J., Cremer P., Jungwirth P. (2017). Beyond the
Hofmeister Series: Ion-Specific Effects on Proteins and Their Biological
Functions. J. Phys. Chem. B.

[23] Xia Y., Zhou P., Kong X., Tian J., Zhang W., Yan S., Hou W., Zhou H., Dong H., Chen X., Wang P., Xu Z., Wan L., Wang B., Liu K. (2023). Designing an asymmetric
ether-like lithium salt to enable fast-cycling
high-energy lithium metal batteries. Nat. Energy.

[24] Min X., Han C., Zhang S., Ma J., Hu N., Li J., Du X., Xie B., Lin H., Kuo C., Chen C., Hu Z., Qiao L., Cui Z., Xu G., Cui G. (2023). Highly Oxidative-Resistant
Cyano-Functionalized Lithium Borate Salt for Enhanced Cycling Performance
of Practical Lithium-Ion Batteries. Angew. Chem.,
Int. Ed..

[25] Song Z. Y., Wang X. X., Feng W. F., Armand M., Zhou Z. B., Zhang H. (2024). Designer Anions for
Better Rechargeable Lithium Batteries and Beyond. Adv. Mater..

[26] Lu Y., Cao Q., Zhang W., Zeng T., Ou Y., Yan S., Liu H., Song X., Zhou H., Hou W., Zhou P., Hu N., Feng Q., Li Y., Liu K. (2024). Breaking the molecular
symmetricity of sulfonimide anions for high-performance lithium metal
batteries under extreme cycling conditions. Nat. Energy.

[27] Ould D., Ruff Z., Penrod M., Didwal P., Weiss T., Mylrea K., Smith H., Bond A., Weatherup R., Grey C., Wright D. (2025). Synthesis and Electrolyte Study of
Lithium Bis­(perfluorinated pinacolato)­Borate for Lithium-Ion Batteries. Chem.—Eur. J..

[28] Wu Y., Wang H., Goh S., Chen J., Ma X., Lu Y., Zhou P., Yan S., Xia Y., Liu Z., Hou W., Ou Y., Zhang Y., Li C., Song X., Wei L., Li K., Liu K. (2026). Rational Design of Asymmetric Lithium
Salts with Multi-Functional Capabilities for Stable Lithium Metal
Batteries. Angew. Chem., Int. Ed..

[29] Yanai H. (2026). Stable and
isolable carbanions: synthesis, chemical bonding, and unique applications. Coord. Chem. Rev..

[30] Han S. D., Yun S. H., Borodin O., Seo D. M., Sommer R. D., Young V. G., Henderson W. A. (2015). Solvate Structures and Computational/Spectroscopic
Characterization of LiPF_6_ Electrolytes. J. Phys. Chem. C.

[31] Chapman N., Borodin O., Yoon T., Nguyen C. C., Lucht B. L. (2017). Spectroscopic
and Density Functional Theory Characterization of Common Lithium Salt
Solvates in Carbonate Electrolytes for Lithium Batteries. J. Phys. Chem. C.

[32] Takeuchi M., Kameda Y., Umebayashi Y., Ogawa S., Sonoda T., Ishiguro S. I., Fujita M., Sano M. (2009). Ion-ion interactions
of LiPF_6_ and LiBF_4_ in propylene carbonate solutions. J. Mol. Liq..

[33] Takeuchi M., Matubayasi N., Kameda Y., Minofar B., Ishiguro S., Umebayashi Y. (2012). Free-Energy and Structural Analysis
of Ion Solvation
and Contact Ion-Pair Formation of Li<SUP>+</SUP> with
BF_4_ <SUP>-</SUP> and PF_6_ <SUP>-</SUP>
in Water and Carbonate Solvents. J. Phys. Chem.
B.

[34] Mennucci B., Cances E., Tomasi J. (1997). Evaluation
of solvent effects in
isotropic and anisotropic dielectrics and in ionic solutions with
a unified integral equation method: Theoretical bases, computational
implementation, and numerical applications. J. Phys. Chem. B.

[35] Borodin O., Giffin G. A., Moretti A., Haskins J. B., Lawson J. W., Henderson W. A., Passerini S. (2018). Insights into the Structure and Transport
of the Lithium, Sodium, Magnesium, and Zinc Bis­(trifluoromethansulfonyl)­imide
Salts in Ionic Liquids. J. Phys. Chem. C.

[36] Zhang X. Q., Chen X., Hou L. P., Li B. Q., Cheng X. B., Huang J. Q., Zhang Q. (2019). Regulating
Anions in the Solvation
Sheath of Lithium Ions for Stable Lithium Metal Batteries. ACS Energy Lett..

[37] Cui B. C., Xu J. J. (2025). Enabling rational electrolyte design
for lithium batteries through
precise descriptors: progress and future perspectives. J. Mater. Chem. A.

[38] Gao Y. C., Yao N., Chen X., Yu L. G., Zhang R., Zhang Q. (2023). Data-Driven
Insight into the Reductive Stability of Ion-Solvent Complexes in Lithium
Battery Electrolytes. J. Am. Chem. Soc..

[39] Fichtner M., Edström K., Ayerbe E., Berecibar M., Bhowmik A., Castelli I. E., Clark S., Dominko R., Erakca M., Franco A. A., Grimaud A., Horstmann B., Latz A., Lorrmann H., Meeus M., Narayan R., Pammer F., Ruhland J., Stein H., Vegge T., Weil M. (2022). Rechargeable Batteries
of the Future-The State of the Art from a
BATTERY 2030+Perspective. Adv. Energy Mater..

[40] Lv C. D., Zhou X., Zhong L. X., Yan C. S., Srinivasan M., Seh Z. W., Liu C. T., Pan H. G., Li S. Z., Wen Y. G., Yan Q. Y. (2022). Machine
Learning: An Advanced Platform
for Materials Development and State Prediction in Lithium-Ion Batteries. Adv. Mater..

[41] Nair M. R., Roy T. (2025). Role of artificial intelligence in the design and discovery of next-generation
battery electrolytes. Chem. Phys. Rev..

[42] Deng J., Chen X., Bai Y., Mo J., Yu X. (2026). Data-Driven
Electrolyte Design for Advanced Rechargeable Lithium Batteries. Chin. J. Chem..

[43] Zhou P., Xiang Y., Liu K. (2024). Understanding
and applying the donor
number of electrolytes in lithium metal batteries. Energy Environ. Sci..

[44] Schmeisser M., Illner P., Puchta R., Zahl A., van Eldik R. (2012). Gutmann Donor
and Acceptor Numbers for Ionic Liquids. Chem.—Eur.
J..

[45] Kulkarni U., Cho W. J., Lee S. H. Y., Hwang D. S., Im T., Jeong Y. K., Ahn K., Yi G. R. (2025). Donor Numbers for
Ionic Liquids and Carbonate Solvents Using <SUP>7</SUP>Li
and
<SUP>23</SUP>Na as Probes. Korean
J. Chem.
Eng..

[46] Holzweber M., Lungwitz R., Doerfler D., Spange S., Koel M., Hutter H., Linert W. (2013). Mutual Lewis Acid-Base Interactions
of Cations and Anions in Ionic Liquids. Chem.—Eur.
J..

[47] Lungwitz R., Spange S. (2008). A hydrogen bond accepting
(HBA) scale for anions, including
room temperature ionic liquids. New J. Chem..

[48] Cremer T., Kolbeck C., Lovelock K. R. J., Paape N., Wölfel R., Schulz P. S., Wasserscheid P., Weber H., Thar J., Kirchner B., Maier F., Steinrück H. P. (2010). Towards
a Molecular Understanding of Cation-Anion Interactions-Probing the
Electronic Structure of Imidazolium Ionic Liquids by NMR Spectroscopy,
X-ray Photoelectron Spectroscopy and Theoretical Calculations. Chem.—Eur. J..

[49] Gousseva E., Towers Tompkins F. K., Seymour J. M., Parker L. G., Clarke C. J., Palgrave R. G., Bennett R. A., Grau-Crespo R., Lovelock K. R. J. (2024). Anion-Dependent Strength Scale of Interactions in Ionic
Liquids from X-ray Photoelectron Spectroscopy, Ab Initio Molecular
Dynamics, and Density Functional Theory. J.
Phys. Chem. B.

[50] Spange S., Lungwitz R., Schade A. (2014). Correlation of molecular
structure
and polarity of ionic liquids. J. Mol. Liq..

[51] Ab
Rani M. A., Brant A., Crowhurst L., Dolan A., Hassan N. H., Hallett J. P., Hunt P. A., Lui M., Niedermeyer H., Perez-Arlandis J. M., Schrems M., To T. Q., Welton T., Wilding R. (2011). Understanding the polarity of ionic
liquids. Phys. Chem. Chem. Phys..

[52] Laurence C., Mansour S., Vuluga D., Legros J. (2021). Measurement of the
hydrogen bond acceptance of ionic liquids and green solvents by the
F-19 solvatomagnetic comparison method. Green
Chem..

[53] Pike S. J., Hutchinson J. J., Hunter C. A. (2017). H-Bond Acceptor Parameters for Anions. J. Am. Chem. Soc..

[54] Burke C. M., Pande V., Khetan A., Viswanathan V., McCloskey B. D. (2015). Enhancing electrochemical intermediate solvation through
electrolyte anion selection to increase nonaqueous Li-O-2 battery
capacity. Proc. Natl. Acad. Sci. U. S. A..

[55] Chu H., Jung J., Noh H., Yuk S., Lee J., Lee J. H., Baek J., Roh Y., Kwon H., Choi D., Sohn K., Kim Y., Kim H. T. (2020). Unraveling
the Dual Functionality of High-Donor-Number Anion in Lean-Electrolyte
Lithium-Sulfur Batteries. Adv. Energy Mater..

[56] Chai D. D., Yan H. T., Wang X., Li X., Fu Y. Z. (2024). Retuning
Solvating Ability of Ether Solvent by Anion Chemistry toward 4.5 V
Class Li Metal Battery. Adv. Funct. Mater..

[57] Yu Y., Li D., Guo Q., Wang M., Zhang H., An Q., Cao L. (2025). Anion-induced
optimization of non-aqueous zinc-air battery performance:
Insights into OTF-selection. J. Power Sources.

[58] González-Aguilera L., Vicent-Luna J., García-Balaguer P., Calero S., Madero-Castro R., Raymundo-Piñero E., Lu X., Gutiérrez M., Ferrer M., del Monte F. (2025). Anion-rich
solvation structures in high entropy aqueous electrolytes for supercapacitors
with enlarged potential windows and superior rate capabilities. J. Mater. Chem. A.

[59] Zhou P., Ou Y., Feng Q., Xia Y., Zhou H., Hou W., Song X., Lu Y., Yan S., Zhang W., He Y., Liu K. (2025). Tuning the Nucleophilicity of Anion in Lithium Salt
to Enable an Anion-Rich Solvation Sheath for Stable Lithium Metal
Batteries. Adv. Funct. Mater..

[60] Erlich R. H., Popov A. I. (1971). SPECTROSCOPIC STUDIES
OF IONIC SOLVATION 0.10. STUDY
OF SOLVATION OF SODIUM IONS IN NONAQUEOUS SOLVENTS BY NA-23 NUCLEAR
MAGNETIC RESONANCE. J. Am. Chem. Soc..

[61] Kamlet M. J., Taft R. W. (1976). SOLVATOCHROMIC COMPARISON
METHOD 0.1. BETA-SCALE OF
SOLVENT HYDROGEN-BOND ACCEPTOR (HBA) BASICITIES. J. Am. Chem. Soc..

[62] Reichardt, C. ; Welton, T. Solvents and Solvent Effects in Organic Chemistry, 4th ed.; Wiley: Weinheim, Germany, 2011.

[63] Wu Y. Z., Hu Q., Liang H. M., Wang A. P., Xu H., Wang L., He X. M. (2023). Electrostatic
Potential as Solvent Descriptor to Enable Rational
Electrolyte Design for Lithium Batteries. Adv.
Energy Mater..

[64] Li Z., Li W., Li Z., Fu J., Chen Q., Yang H., Guo X. (2025). Fluorine-oxygen co-coordination
of lithium in fluorinated polymers
for broad temperature quasi-solid-state batteries. Nat. Commun..

[65] Chen K., Chen H., Zheng Z., Liu Y., Fang Y., Chen Z., Cao Y. (2025). Identifying the Role
of Solvation
Entropy for the Solvation Chemistry in Nonaqueous Electrolytes. Angew. Chem., Int. Ed..

[66] Zhao F., Zhang J., Chen J., Gu Z., Fan X., Zhu L., Na H., Dong M., Guan C., Kong L. (2025). Lithium Salt
Association-Mediated Interfacial Charge Exchange for Low-Temperature
Lithium-Metal Batteries: Beyond Lithium De-Solvation Manner. Research.

[67] Qi H., Liv P. (2025). Solvation engineering
in lithium-ion batteries: from fundamental
mechanisms to electrolyte design. Ionics.

[68] Peng Z., Ding K., Lai M., Qiu R., Xiao Y., Shi J., Guan X., Cai Y., Xu C., Wang F., Zheng Q. (2026). Rational electrolyte solvent screening
for high-energy lithium metal
batteries at low temperatures. Nat. Commun..

[69] Niedermeyer H., Ashworth C., Brandt A., Welton T., Hunt P. A. (2013). A step
towards the a priori design of ionic liquids. Phys. Chem. Chem. Phys..

[70] Cerda-Monje A., Ormazabal-Toledo R., Cardenas C., Fuentealba P., Contreras R. (2014). Regional Electrophilic and Nucleophilic Fukui Functions
Efficiently Highlight the Lewis Acidic/Basic Regions in Ionic Liquids. J. Phys. Chem. B.

[71] Wu W. H., Lu Y. X., Ding H. R., Peng C. J., Liu H. L. (2015). The acidity/basicity
of metal-containing ionic liquids: insights from surface analysis
and the Fukui function. Phys. Chem. Chem. Phys..

[72] Liu Y., Wang J. F. (2018). Lewis Acidity and
Basicity of Mixed Chlorometallate
Ionic Liquids: Investigations from Surface Analysis and Fukui Function. Molecules.

[73] Towers
Tompkins F. K., Parker L. G., Fogarty R. M., Seymour J. M., Rowe R., Palgrave R. G., Matthews R. P., Bennett R. A., Hunt P. A., Lovelock K. R. J. (2025). Efficient prediction of the local
electronic structure of ionic liquids from low-cost calculations. Phys. Chem. Chem. Phys..

[74] Hu H. P., Shan Y. Q., Zhao Q. M., Wang J. L., Wu L. J., Liu W. Q. (2024). The prediction of donor number and acceptor number
of electrolyte solvent molecules based on machine learning. J. Energy Chem..

[75] Luo H. R., Gou Q. Z., Zheng Y. J., Wang K. X., Yuan R. D., Zhang S. D., Fang W., Luogu Z., Hu Y. Z., Mei H. P., Song B. Y., Sun K., Wang J. H., Li M. (2025). Machine Learning-Assisted High-Donor-Number
Electrolyte Additive
Screening toward Construction of Dendrite-Free Aqueous Zinc-Ion Batteries. ACS Nano.

[76] Zhan Y. X., Ren X. J., Zhao S., Guo Z. L. (2025). Enhancing prediction
of electron affinity and ionization energy in liquid organic electrolytes
for lithium-ion batteries using machine learning. J. Power Sources.

[77] Gao Y., Qi S., Li M., Ma T., Song H., Cui Z., Liang Z., Du L. (2025). Machine Learning
Assisted Prediction
of Donor Numbers: Guiding Rational Fabrication of Polymer Electrolytes
for Lithium-Ion Batteries. Angew. Chem., Int.
Ed..

[78] CAHEN Y., HANDY P., ROACH E., POPOV A. (1975). SPECTROSCOPIC STUDIES
OF IONIC SOLVATION. 16. LITHIUM-7 AND CHLORINE-35 NUCLEAR MAGNETIC-RESONANCE
STUDIES IN VARIOUS SOLVENTS. J. Phys. Chem..

[79] Holoubek J., Yu K., Wu J., Wang S., Li M., Gao H., Hui Z., Hyun G., Yin Y., Mu A., Kim K., Liu A., Yu S., Pascal T., Liu P., Chen Z. (2023). Toward a quantitative
interfacial description of solvation for Li metal battery operation
under extreme conditions. Proc. Natl. Acad.
Sci. U. S. A..

[80] Maciel G. E., Hancock J. K., Lafferty L. F., Mueller P. A., Musker W. K. (1966). Lithium-7
Chemical Shifts of Lithium Perchlorate and Bromide in Various Solvents. Inorg. Chem..

[81] Briggs, D. , Grant, J. T. , Eds. Surface Analysis by Auger and X-ray Photoelectron Spectroscopy; IM Publications: Manchester, 2003.

[82] Lovelock K.
R. J., Kolbeck C., Cremer T., Paape N., Schulz P. S., Wasserscheid P., Maier F., Steinrück H. P. (2009). Influence
of Different Substituents on the Surface Composition of Ionic Liquids
Studied Using ARXPS. J. Phys. Chem. B.

[83] Apperley D. C., Hardacre C., Licence P., Murphy R. W., Plechkova N. V., Seddon K. R., Srinivasan G., Swadźba-Kwaśny M., Villar-Garcia I. J. (2010). Speciation
of chloroindate­(III) ionic liquids. Dalton Trans..

[84] Villar-Garcia I. J., Smith E. F., Taylor A. W., Qiu F. L., Lovelock K. R. J., Jones R. G., Licence P. (2011). Charging of
ionic liquid surfaces
under X-ray irradiation: the measurement of absolute binding energies
by XPS. Phys. Chem. Chem. Phys..

[85] Hurisso B. B., Lovelock K. R. J., Licence P. (2011). Amino acid-based
ionic liquids: using
XPS to probe the electronic environment via binding energies. Phys. Chem. Chem. Phys..

[86] Taylor A. W., Men S., Clarke C. J., Licence P. (2013). Acidity and basicity of halometallate-based
ionic liquids from X-ray photoelectron spectroscopy. RSC Adv..

[87] Currie M., Estager J., Licence P., Men S., Nockemann P., Seddon K. R., Swadźba-Kwaśny M., Terrade C. (2013). Chlorostannate­(II) Ionic Liquids: Speciation, Lewis
Acidity, and Oxidative Stability. Inorg. Chem..

[88] Villar-Garcia I. J., Lovelock K. R. J., Men S., Licence P. (2014). Tuning the electronic
environment of cations and anions using ionic liquid mixtures. Chem. Sci..

[89] Men S. A., Lovelock K. R. J., Licence P. (2017). X-ray photoelectron spectroscopy
of trihalide ionic liquids: Comparison to halide-based analogues,
anion basicity and beam damage. Chem. Phys.
Lett..

[90] Fogarty R. M., Matthews R. P., Ashworth C. R., Brandt-Talbot A., Palgrave R. G., Bourne R. A., Vander Hoogerstraete T., Hunt P. A., Lovelock K. R. J. (2018). Experimental validation of calculated
atomic charges in ionic liquids. J. Chem. Phys..

[91] Fogarty R. M., Rowe R., Matthews R. P., Clough M. T., Ashworth C. R., Brandt A., Corbett P. J., Palgrave R. G., Smith E. F., Bourne R. A., Chamberlain T. W., Thompson P. B. J., Hunt P. A., Lovelock K. R. J. (2018). Atomic charges
of sulfur in ionic liquids: experiments
and calculations. Faraday Discuss..

[92] Seymour J. M., Gousseva E., Towers Tompkins F. K., Parker L. G., Alblewi N. O., Clarke C. J., Hayama S., Palgrave R. G., Bennett R. A., Matthews R. P., Lovelock K. R. J. (2024). Unravelling
the complex speciation
of halozincate ionic liquids using X-ray spectroscopies and calculations. Faraday Discuss..

[93] Longo L. S., Smith E. F., Licence P. (2016). Study of the Stability of 1-Alkyl-3-methylimidazolium
Hexafluoroantimonate­(V) Based Ionic Liquids Using X-ray Photoelectron
Spectroscopy. ACS Sustain. Chem. Eng..

[94] May B., Honle M., Heller B., Greco F., Bhuin R., Steinrück H. P., Maier F. (2017). Surface-Induced Changes in the Thermochromic
Transformation of an Ionic Liquid Cobalt Thiocyanate Complex. J. Phys. Chem. Lett..

[95] Seymour J. M., Gousseva E., Parker L. G., Towers
Tompkins F. K., Fogarty R. M., Frankemoelle L., Rowe R., Clarke C. J., Duncan D. A., Palgrave R. G., Bennett R. A., Hunt P. A., Lovelock K. R. J. (2025). d10s2 Post-Transition
Metal Anions: Identifying and
Analyzing Their Dual-Mode Lewis Basicity. J.
Phys. Chem. Lett..

[96] Winter B., Faubel M. (2006). Photoemission from liquid aqueous
solutions. Chem. Rev..

[97] Ottosson N., Vacha R., Aziz E. F., Pokapanich W., Eberhardt W., Svensson S., Öhrwall G., Jungwirth P., Björneholm O., Winter B. (2009). Large variations in
the propensity of aqueous oxychlorine anions for the solution/vapor
interface. J. Chem. Phys..

[98] Seidel R., Winter B., Bradforth S. E. (2016). Valence
Electronic Structure of Aqueous
Solutions: Insights from Photoelectron Spectroscopy. Annu. Rev. Phys. Chem..

[99] Gaiduk A. P., Govoni M., Seidel R., Skone J. H., Winter B., Galli G. (2016). Photoelectron Spectra
of Aqueous Solutions from First Principles. J. Am. Chem. Soc..

[100] Pham T. A., Govoni M., Seidel R., Bradforth S. E., Schwegler E., Galli G. (2017). Electronic structure of aqueous solutions:
Bridging the gap between theory and experiments. Sci. Adv..

[101] Towers Tompkins F. K., Gousseva E., Bennett R. A., Grau-Crespo R., Lovelock K. R. J. (2025). Core-level binding energies describe
electrostatic
potentials at nuclei for ionic liquids. Phys.
Chem. Chem. Phys..

[102] Marcus Y. (1993). THE PROPERTIES
OF ORGANIC LIQUIDS THAT ARE RELEVANT
TO THEIR USE AS SOLVATING SOLVENTS. Chem. Soc.
Rev..

[103] Pike S. J., Lavagnini E., Varley L. M., Cook J. L., Hunter C. A. (2019). H-Bond
donor parameters for cations. Chem. Sci..

[104] McGrogan A., Lafferty J., O’Neill L., Brown L., Young J. M., Goodrich P., Muldoon M. J., Moura L., Youngs S., Hughes T. L., Gärtner S., Youngs T. G. A., Holbrey J. D., Swadzba-Kwasny M. (2024). Liquid Structure
of Ionic Liquids with NTf_2_ <SUP>-</SUP> Anions,
Derived
from Neutron Scattering. J. Phys. Chem. B.

[105] Gumaste T., Cooper F., Blakemore J. (2026). Analysis of
Bis­(trifluoromethylsulfonyl)­imide Interactions with Metal Cations
Through a Chemical Informatics Approach. Molecules.

[106] Best S. A., Walton R. A. (1976). X-RAY PHOTOELECTRON-SPECTRA
OF INORGANIC
MOLECULES. 19. THIOCYANATO AND ISOTHIOCYANATO COMPLEXES OF TRANSITION-METALS
- CARBON-1S, NITROGEN-1S AND SULFUR-2P BINDING-ENERGIES AND THEIR
POSSIBLE CORRELATION WITH MODE OF THIOCYANATE BONDING. Isr. J. Chem..

[107] Lovelock K. R. J., Villar-Garcia I. J., Maier F., Steinrück H. P., Licence P. (2010). Photoelectron Spectroscopy of Ionic Liquid-Based Interfaces. Chem. Rev..

[108] Lovelock, K. R. J. ; Licence, P. In Ionic Liquids UnCOILed: Critical Expert Overviews; Seddon, K. R. , Plechkova, N. V. , Eds.; Wiley: Oxford, 2012; Chapter 8, pp 251–282.

[109] Steinrück H. P. (2012). Recent developments in the study of ionic liquid interfaces
using X-ray photoelectron spectroscopy and potential future directions. Phys. Chem. Chem. Phys..

[110] Erdmann P., Greb L. (2022). What Distinguishes the Strength and
the Effect of a Lewis Acid: Analysis of the Gutmann-Beckett Method. Angew. Chem., Int. Ed..

[111] Stoess L., Greb L. (2025). What distinguishes the strength and
the effect of a Lewis base: insights with a strong chromogenic silicon
Lewis acid. Chem. Sci..

[112] Cláudio A. F.
M., Swift L., Hallett J. P., Welton T., Coutinho J. A. P., Freire M. G. (2014). Extended
scale for the hydrogen-bond
basicity of ionic liquids. Phys. Chem. Chem.
Phys..

[113] Gousseva E., Midgley S. D., Seymour J. M., Seidel R., Grau-Crespo R., Lovelock K. R. J. (2022). Understanding X-ray Photoelectron
Spectra of Ionic Liquids: Experiments and Simulations of 1-Butyl-3-methylimidazolium
Thiocyanate. J. Phys. Chem. B.

[114] Towers Tompkins F. K., Parker L. G., Fogarty R. M., Seymour J. M., Gousseva E., Grinter D. C., Palgrave R. G., Smith C. D., Bennett R. A., Matthews R. P., Lovelock K. R. J. (2024). Controlling
and
predicting alkyl-onium electronic structure. Chem. Commun..

[115] Philippi F., Middendorf M., Shigenobu K., Matsuyama Y., Palumbo O., Pugh D., Sudoh T., Dokko K., Watanabe M., Schoenhoff M., Shinoda W., Ueno K. (2024). Evolving better
solvate electrolytes
for lithium secondary batteries. Chem. Sci..

[116] Pokorny D., Syrovy T., Klikar M., Parík P., Buresová Z., Rehácková L., Bures F. (2026). Tailoring
lithium dicyanoimidazolide: structure-property relationships. Mater. Adv..

[117] Niedzicki L., Karpierz E., Zawadzki M., Dranka M., Kasprzyk M., Zalewska A., Marcinek M., Zachara J., Domanska U., Wieczorek W. (2014). Lithium cation conducting TDI anion-based
ionic liquids. Phys. Chem. Chem. Phys..

[118] Zavalij P., Yang S., Whittingham M. (2003). Structures
of potassium, sodium and lithium bis­(oxalato)­borate salts from powder
diffraction data. Acta Crystallogr. Sect. B-Struct.
Commun..

[119] Galabov B., Ilieva S., Koleva G., Allen W. D., Schaefer H. F., von R. Schleyer P. (2013). Structure-reactivity relationships
for aromatic molecules: electrostatic potentials at nuclei and electrophile
affinity indices. Wiley Interdiscip. Rev.-Comput.
Mol. Sci..

[120] Suresh C.
H., Remya G. S., Anjalikrishna P. K. (2022). Molecular
electrostatic potential analysis: A powerful tool to interpret and
predict chemical reactivity. Wiley Interdiscip.
Rev.-Comput. Mol. Sci..

[121] Suresh C. H., Anila S. (2023). Molecular Electrostatic Potential
Topology Analysis of Noncovalent Interactions. Acc. Chem. Res..

[122] Asl H., Manthiram A. (2020). Reining in dissolved transition-metal
ions. Science.

[123] Fogarty R. M., Matthews R. P., Hunt P. A., Lovelock K. R. J. (2025). Accurate
prediction of ionic liquid density-of-states from low-cost calculations. Phys. Chem. Chem. Phys..

[124] Fogarty R. M., Palgrave R. G., Bourne R. A., Handrup K., Villar-Garcia I. J., Payne D. J., Hunt P. A., Lovelock K. R. J. (2019). Electron
spectroscopy of ionic liquids: experimental identification of atomic
orbital contributions to valence electronic structure. Phys. Chem. Chem. Phys..

[125] Seymour J. M., Gousseva E., Large A. I., Clarke C. J., Licence P., Fogarty R. M., Duncan D. A., Ferrer P., Venturini F., Bennett R. A., Palgrave R. G., Lovelock K. R. J. (2021). Experimental
measurement and prediction of ionic liquid ionisation energies. Phys. Chem. Chem. Phys..

[126] Grinter D., Ferrer P., Venturini F., van Spronsen M., Large A., Kumar S., Jaugstetter M., Iordachescu A., Watts A., Schroeder S., Kroner A., Grillo F., Francis S., Webb P., Hand M., Walters A., Hillman M., Held G. (2024). VerSoX B07-B:
a high-throughput XPS and ambient pressure NEXAFS beamline at Diamond
Light Source. J. Synchrot. Radiat..

[127] Seidel R., Pohl M. N., Ali H., Winter B., Aziz E. F. (2017). Advances in liquid phase soft-x-ray
photoemission spectroscopy:
A new experimental setup at BESSY II. Rev. Sci.
Instrum..

[128] Zhu S., Scardamaglia M., Kundsen J., Sankari R., Tarawneh H., Temperton R., Pickworth L., Cavalca F., Wang C., Tissot H., Weissenrieder J., Hagman B., Gustafson J., Kaya S., Lindgren F., Källquist I., Maibach J., Hahlin M., Boix V., Gallo T., Rehman F., D’Acunto G., Schnadt J., Shavorskiy A. (2021). HIPPIE: a
new platform for ambient-pressure X-ray photoelectron spectroscopy
at the MAX IV Laboratory. J. Synchrot. Radiat..

[129] Rueff J., Ablett J., Céolin D., Prieur D., Moreno T., Balédent V., Lassalle-Kaiser B., Rault J., Simon M., Shukla A. (2015). The GALAXIES
beamline at the SOLEIL synchrotron: inelastic X-ray scattering and
photoelectron spectroscopy in the hard X-ray range. J. Synchrot. Radiat..

[130] Frisch, M. J. ; Trucks, G. W. ; Schlegel, H. B. ; Scuseria, G. E. ; Robb, M. A. ; Cheeseman, J. R. ; Scalmani, G. ; Barone, V. ; Petersson, G. A. ; Nakatsuji, H. ; Li, X. ; Caricato, M. ; Marenich, A. V. ; Bloino, J. ; Janesko, B. G. ; Gomperts, R. ; Mennucci, B. ; Hratchian, H. P. ; Ortiz, J. V. ; Izmaylov, A. F. ; Sonnenberg, J. L. ; Williams-Young, D. ; Ding, F. ; Lipparini, F. ; Egidi, F. ; Goings, J. ; Peng, B. ; Petrone, A. ; Henderson, T. ; Ranasinghe, D. ; Zakrzewski, V. G. ; Gao, J. ; Rega, N. ; Zheng, G. ; Liang, W. ; Hada, M. ; Ehara, M. ; Toyota, K. ; Fukuda, R. ; Hasegawa, J. ; Ishida, M. ; Nakajima, T. ; Honda, Y. ; Kitao, O. ; Nakai, H. ; Vreven, T. ; Throssell, K. ; Montgomery, J. J. A. ; Peralta, J. E. ; Ogliaro, F. ; Bearpark, M. J. ; Heyd, J. J. ; Brothers, E. N. ; Kudin, K. N. ; Staroverov, V. N. ; Keith, T. A. ; Kobayashi, R. ; Normand, J. ; Raghavachari, K. ; Rendell, A. P. ; Burant, J. C. ; Iyengar, S. S. ; Tomasi, J. ; Cossi, M. ; Millam, J. M. ; Klene, M. ; Adamo, C. ; Cammi, R. ; Ochterski, J. W. ; Martin, R. L. ; Morokuma, K. ; Farkas, O. ; Foresman, J. B. ; Fox, D. J. Gaussian 16, revision C.01; Gaussian, Inc., 2016.

[131] Fairley N., Fernandez V., Richard-Plouet M., Guillot-Deudon C., Walton J., Smith E., Flahaut D., Greiner M., Biesinger M., Tougaard S., Morgan D., Baltrusaitis J. (2021). Systematic
and collaborative approach to problem solving
using X-ray photoelectron spectroscopy. Appl.
Surf. Sci. Adv..

[132] Kolbeck C., Killian M., Maier F., Paape N., Wasserscheid P., Steinrück H. P. (2008). Surface characterization of functionalized
imidazolium-based ionic liquids. Langmuir.

