# Supplementary material for: Element-Specific Donor Numbers for Anions
Source: J Am Chem Soc. 2026 Aug 13;148(33):35498–514. doi: 10.1021/jacs.6c06567 (PMC13523738; doi:10.1021/jacs.6c06567)
Supplement: Supplementary file 1 [file ja6c06567_si_001.pdf]

Element-Specific Donor Numbers for Anions

Lewis G. Parker,[1,2] Frances K. Towers Tompkins,[1] Jake M. Seymour,[1] Ekaterina Gousseva,[1] Christopher D. Smith,[1] Roger A. Bennett,[1] Pilar Ferrer,[3] David C. Grinter,[3] Claudia Kolbeck,[4] Dennis Hein,[5] Garlef Wartner,[5] Robert Seidel,[5] Denis Céolin,[6] Robert Temperton,[7] Robert G. Palgrave,[8] Richard P. Matthews,[9] Kevin R. J. Lovelock[1,2]

[1] Department of Chemistry, University of Reading, Reading, UK
[2] Department of Chemistry, University of Bath, Bath, UK
[3] Diamond Light Source, Didcot, Oxfordshire, UK
[4] Fritz-Haber-Institut der Max-Planck-Gesellschaft, Berlin, Germany
[5] Helmholtz-Zentrum Berlin für Materialien und Energie (HZB), Berlin, Germany
[6] Synchrotron SOLEIL, Gif sur Yvette, France
[7] MAX IV Laboratory, Lund University, Lund, Sweden
[8] Department of Chemistry, University College London, London, UK
[9] School of Health, Sport and Bioscience, University of East London, London, UK
Contact E-mail: krjl20@bath.ac.uk

1. Introduction S2
2. Methods. Anions, cations and solvents studied S3-S14
3. Methods. Anion-focused samples studied using XPS experiments S15-S18
4. Methods. Samples studied using lone-anion SMD calculations S19
5. Methods. Lone-anion-SMD DFT calculations S20
6. Methods. Producing calculated and "experimental" XP spectra S21-S22
7. Data analysis. Peak fitting core level XP spectra S23-S26
8. Data analysis. Charge referencing and area normalization S27-S32
9. Results. XPS: demonstrating purity using XPS for new ionic liquid samples S33-S41
10. Results. XPS: demonstrating purity using XPS for new salt in water samples S42-S55
11. Results. XPS: demonstrating purity using XPS for new salt in PC samples S56-S64
12. Results. XPS: demonstrating purity using XPS for new salt in TEP samples S65-S69
13. Results. Anion element-specific donor numbers: experimental uncertainty S70-S75
14. Results. XP spectra used to obtain $DN_{E\text{-}XPS}$ S76-S81
15. Results. E_B(core,anion) for ionic liquids with more than one sample measured S82-S83
16. Results. E_B(core,anion) for water with more than one sample measured S84
17. Results. E_B(core,anion) for PC with more than one sample measured S85
18. Results. E_B(core,anion) for TEP with more than one sample measured S86
19. Results. Calculated E_B(core,anion) using ionic liquid SMD parameters S87-S88
20. Results. Calculated E_B(core,anion) using water SMD parameters S89
21. Results. Calculated E_B(core,anion) using PC SMD parameters S90
22. Results. Calculated E_B(core,anion) using TEP SMD parameters S91
23. Results. E_B(core,anion) correction for lone-anion molecular solvent SMD calculations S92
24. Results. E_B(core,anion) for ionic liquids used to obtain $DN_{E\text{-}XPS}$ S93
25. Results. E_B(core,anion) for water S94
26. Results. E_B(core,anion) for PC S95
27. Results. E_B(core,anion) for TEP S96
28. Results. Effect of countercations on $DN_{E\text{-}XPS}$ S97-S101
29. Results. Experimental and calculated XP spectra for anions S102
30. Results. Linear correlations of E_B(core,anion) for PC and TEP S103-S104
31. Results. Bulk versus surface contributions to E_B(core,anion) S105
32. Results. Comparisons of $DN_{E\text{-}XPS}$ to literature DN/β S106-S109
33. References S110

1. Introduction

1.1. Influence of the probe on DN/β

Different heterocyclic dye molecule probes give significantly different β for the same ionic liquid.[1-3] For $[CH_3CO_2]^-$ (with a $[C_4C_1Im]^+$ countercation) β has been found as 0.85,[1] 1,20,[2] and 1.30,[3] very significant differences given β for anions only varies between ~0.15 and ~1.3.[1-3]

Different organic countercations give significantly different DN for the same anion. For organic countercations, $[C_nC_1Im]^+$, $DN_{Na\text{-}NMR}$ = 11.2 for n = 2 and $DN_{Na\text{-}NMR}$ = 6.7 for n = 12 (*i.e.* different alkyl chain substituent lengths).[4]

1.2. Calculated anion descriptors

Table S1. Strengths and weaknesses of calculated anion descriptors that capture intermolecular anion-cation/anion-molecule interaction strengths

Descriptor	Ref.	Probe	Atomic-level descriptor?	Intrinsic anion property?	Interpretability	Validated?
$-E_{HB}$ (very closely related to β)	5	$[C_4C_1Im]^+$	✘	✘	Excellent	✘
Frontier orbital energies	6	no probe	✘	✓	Excellent	✘
$DN_{E\text{-}XPS}$	Here	no probe	✓	✓	Excellent	✓

2. Methods. Anions, cations and solvents studied

For each anion the atom type most likely to interact with a cation or molecule, known here as the anion primary donor atom, can be readily identified from a combination of structural studies and chemical intuition, *e.g.* O in $[(CF_3SO_2)_2N]^-$.[7] Donor atoms for other anions are listed in Table S2.

Table S2. Structures, names and abbreviations of anions studied. Red = primary (1°) donor atom. Green = secondary (2°) or tertiary (3°) donor atom

Anion no.	Abbreviation	Name	Structure	1° donor atom	2°/3° donor atom(s)
1	$[NO_3]^-$	nitrate	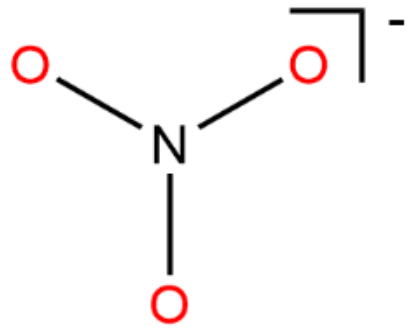	O	
2	$[CH_3CO_2]^-$ (also known as $[OAc]^-$)	acetate	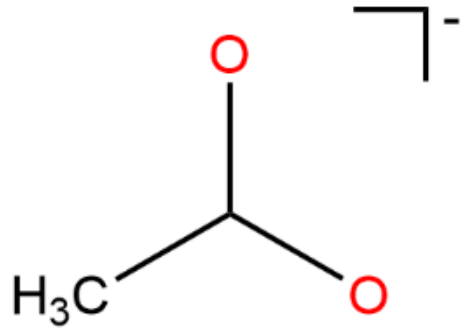	O	
3	$[CH_3SO_3]^-$ (also known as $[MeSO_3]^-$)	methanesulfonate	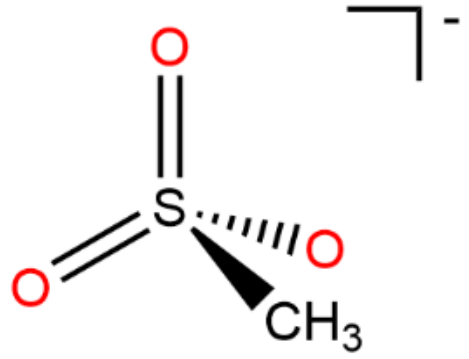	O	

4	$[CH_3C_6H_4SO_3]^-$ (also known as $[Tos]^-$)	toluenesulfonate (tosylate)	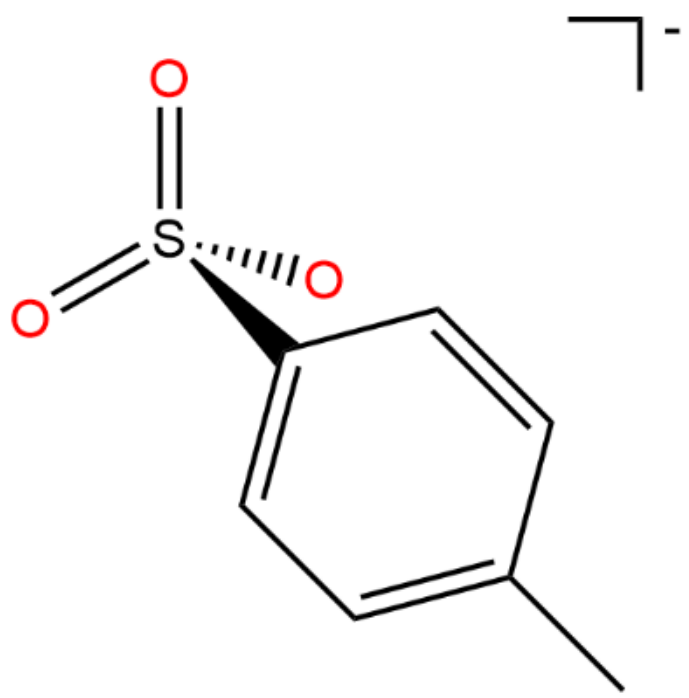	O	
5	$[ClO_4]^-$	perchlorate	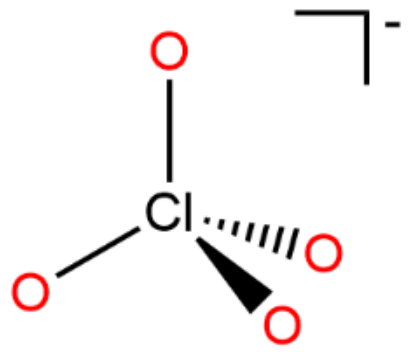	O	
6	$[HOSO_3]^-$ (also known as $[HSO_4]^-$)	hydrogensulfate	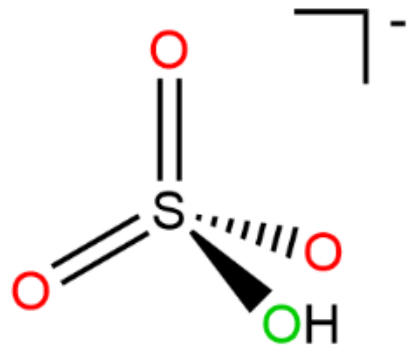	O_{SO}	O_{OH}
7	$[CH_3OSO_3]^-$ (also known as $[MeSO_4]^-$)	methylsulfate	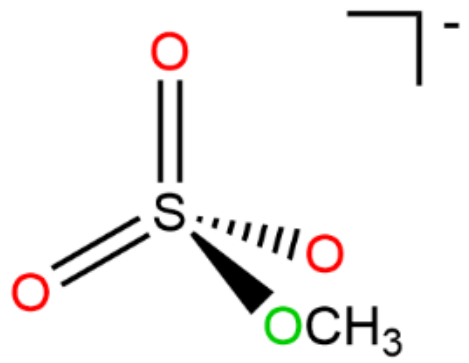	O_{SO}	O_{OC}
8	$[C_8H_{17}OSO_3]^-$ (also known as $[OcSO_4]^-$)	octylsulfate	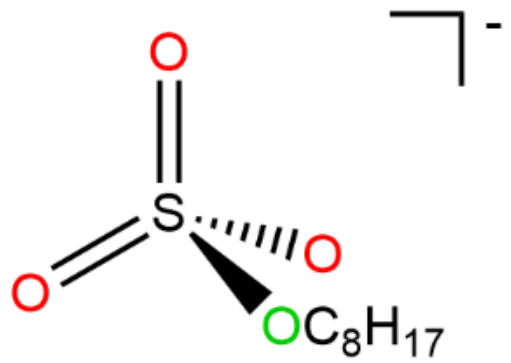	O_{SO}	O_{OC}

9	$[(CH_3O)_2PO_2]^-$	dimethylphosphate	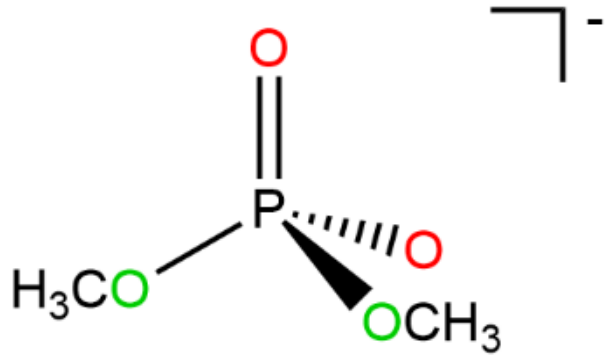	O_{PO}	O_{OC}
10	$[B(C_2O_4)_2]^-$ (commonly known as $[BOB]^-$)	bis(oxalato)borate	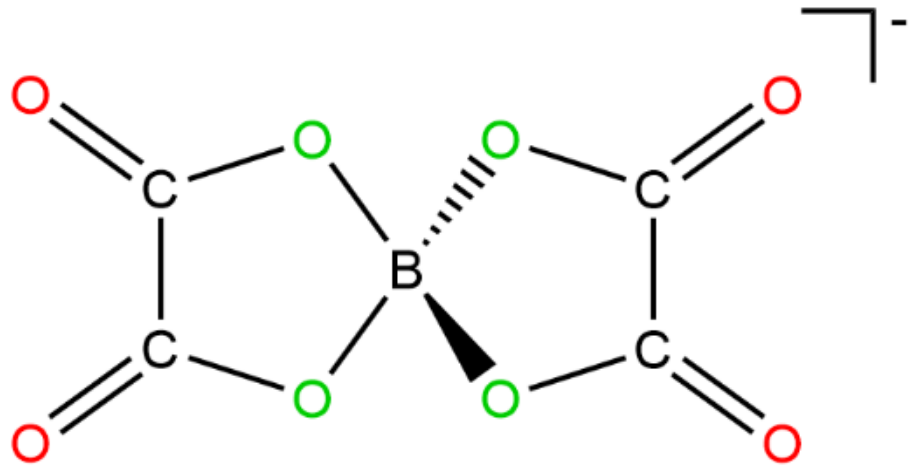	O_{CO}	O_{BO}
11	$[CF_3CO_2]^-$ (commonly known as $[TFA]^-$)	trifluoroacetate	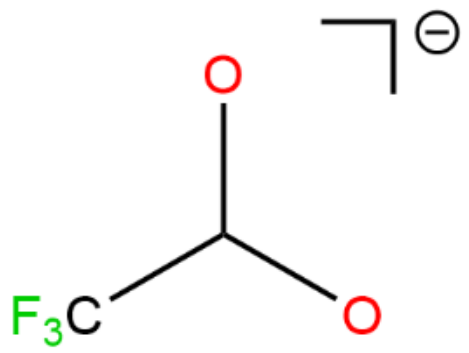	O	F
12	$[CF_3SO_3]^-$ (commonly known as $[TfO]^-$)	trifluoromethylsulfonate	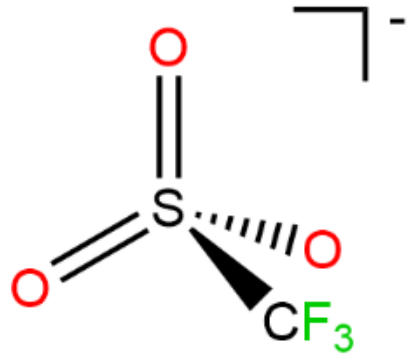	O	F
13	$[(CF_3SO_2)_2N]^-$ (commonly known as $[NTf_2]^-$ or $[TFSI]^-$)	bis[(trifluoromethane)sulfonyl]imide	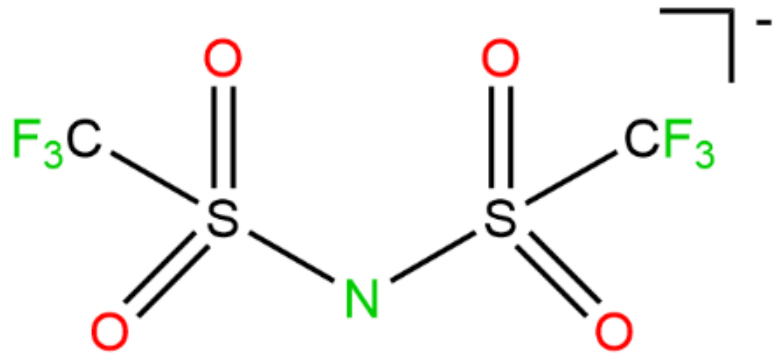	O	N, F
14	$[(FSO_2)_2N]^-$ (commonly known as $[FSI]^-$)	bis(fluorosulfonyl)imide	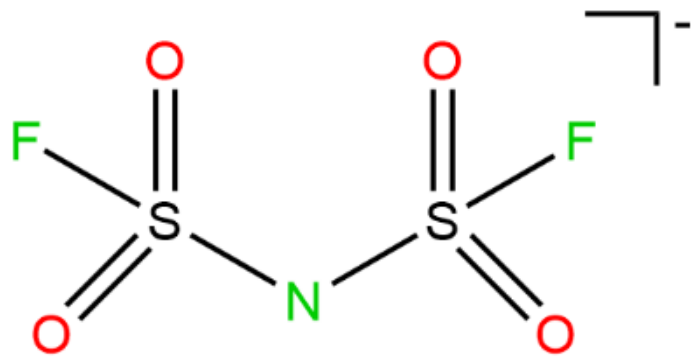	O	N, F

15	$[(C_2F_5SO_2)_2N]^-$ (commonly known as $[NPf_2]^-$ or $[BETI]^-$)	bis(pentafluoroethylsulfonyl)imide	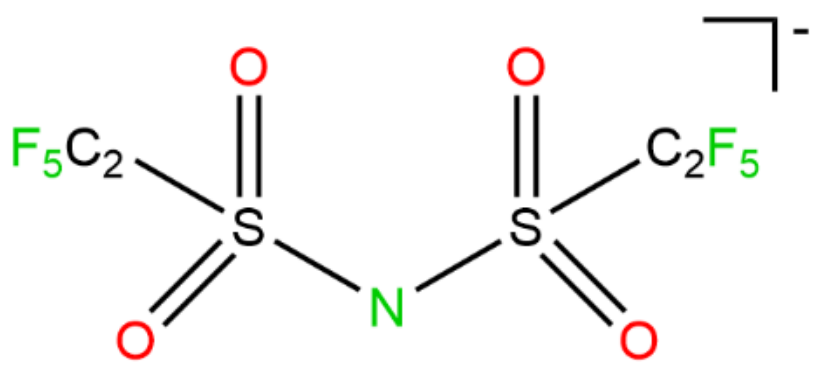	O	N, F
16	$[C(CN)_3]^-$	tricyanomethanide	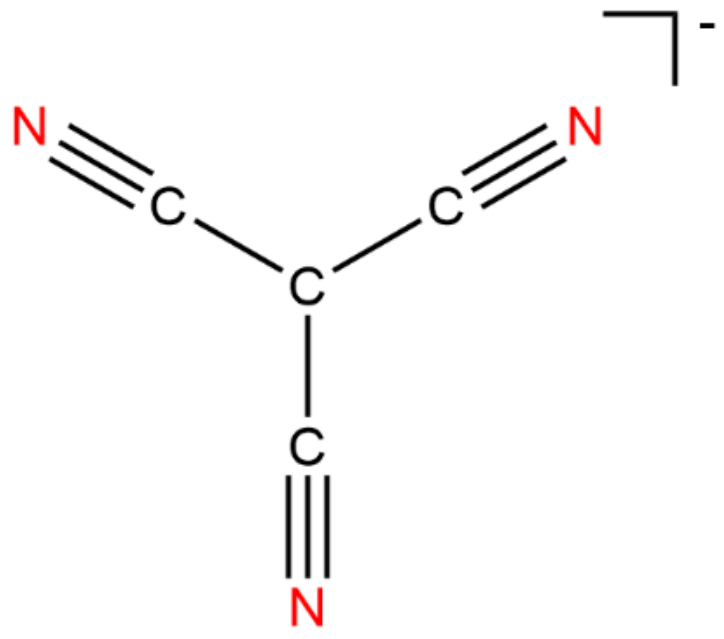	N	
17	$[B(CN)_4]^-$	tetracyanoborate	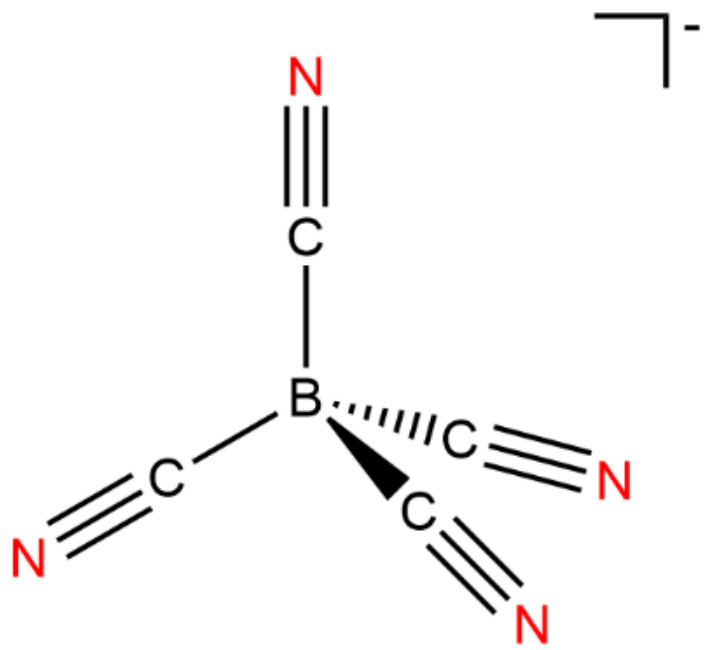	N	
18	$[(CH)_2N_2CH]^-$	imidazolate	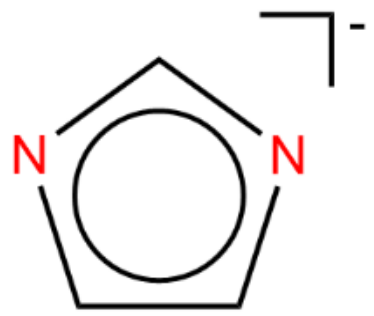	N	

19	$[C_6H_4N_2CH]^-$	benzimidazolate	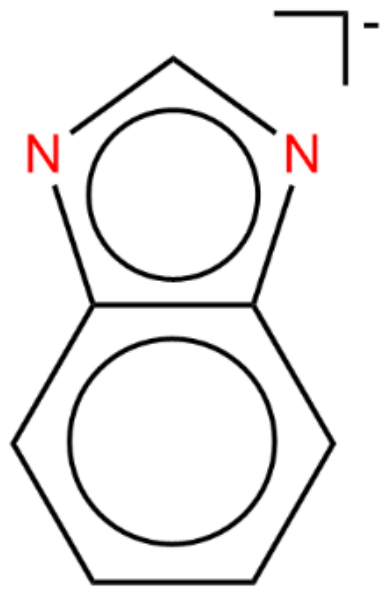	N	
20	$[N(CN)_2]^-$	dicyanamide	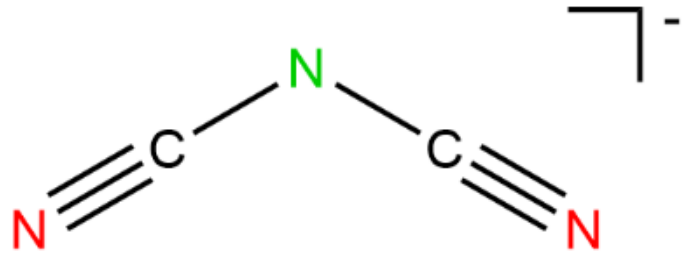	$N_{terminal}$	N_{bridge}
21	$[SCN]^-$	thiocyanate	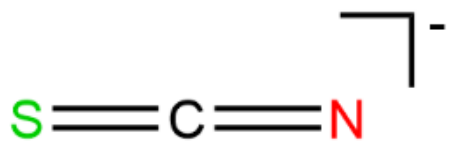	N	S
22	$[CF_3(C_3N_2)(CN)_2]^-$ (commonly known as $[TDI]^-$)	4,5-dicyano-2-(trifluoromethyl)imidazol-1-ide	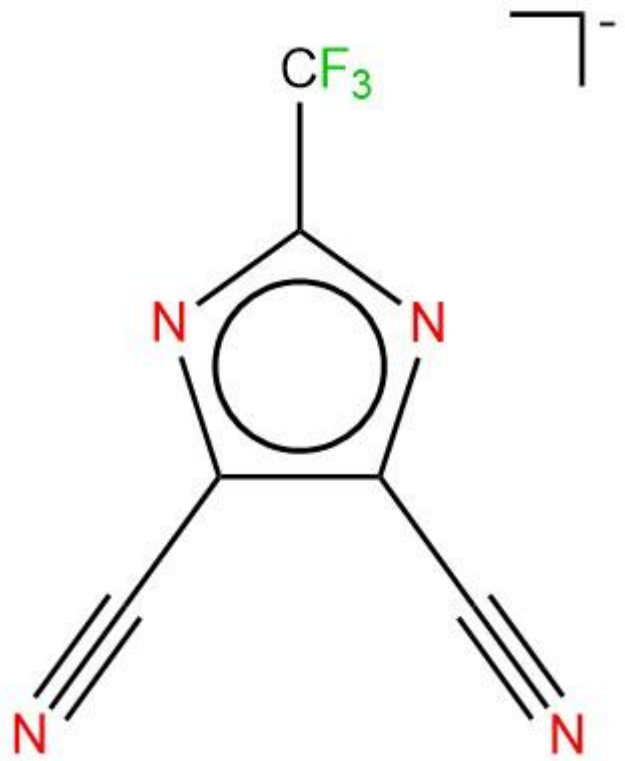	N_{ring} N_{CN}	F
23	$[BF_4]^-$	tetrafluoroborate	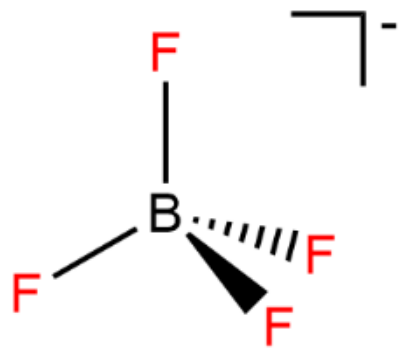	F	

24	$[PF_6]^-$	hexafluorophosphate	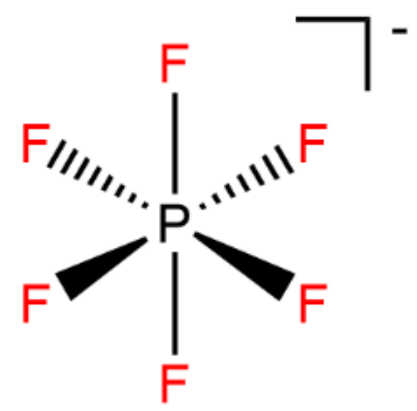	F	
25	$[SbF_6]^-$	hexafluoroantimonate	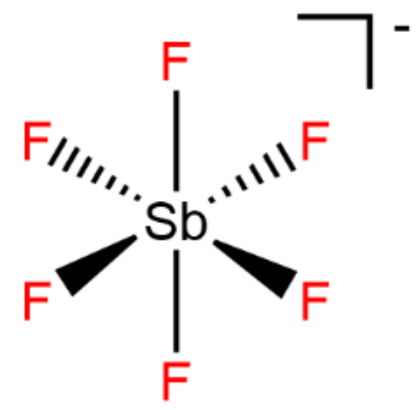	F	
26	$[(C_2F_5)_3PF_3]^-$ (commonly known as $[FAP]^-$)	tris(pentafluoroethyl)trifluorophosphate	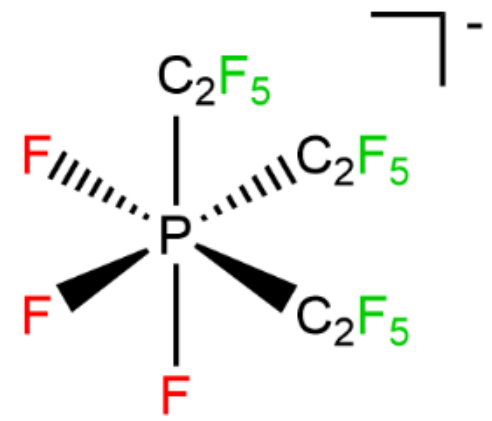	F_{PF}	F_{CF}
27	Cl^-	chloride	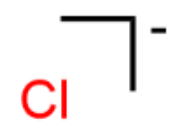	Cl	
28	$[ZnCl_4]^{2-}$	tetrachlorozincate	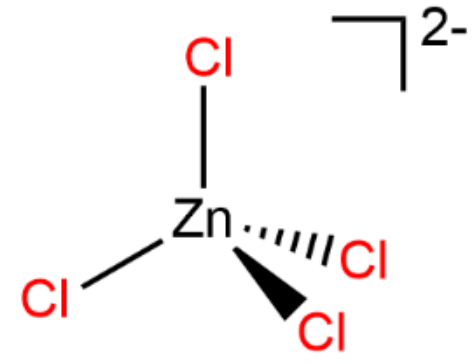	Cl	
29	$[FeCl_4]^{2-}$	tetrachloroferrate(II)	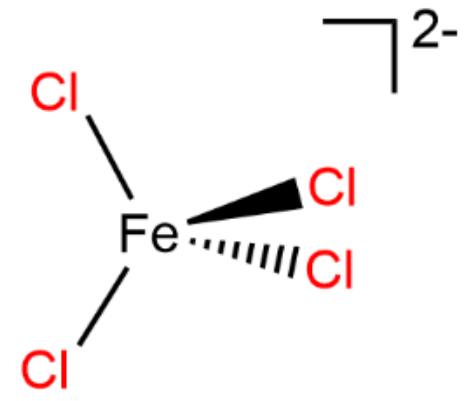	Cl	

30	$[CoCl_4]^{2-}$	tetrachlorocobaltate	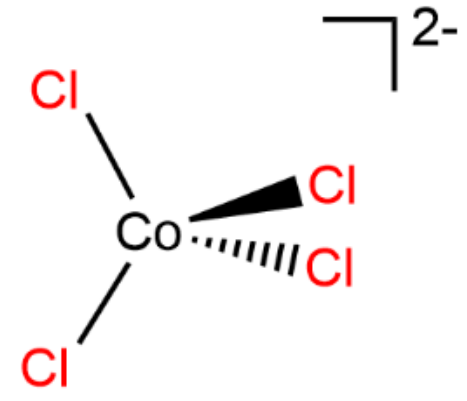	Cl
31	$[NiCl_4]^{2-}$	tetrachloronickelate	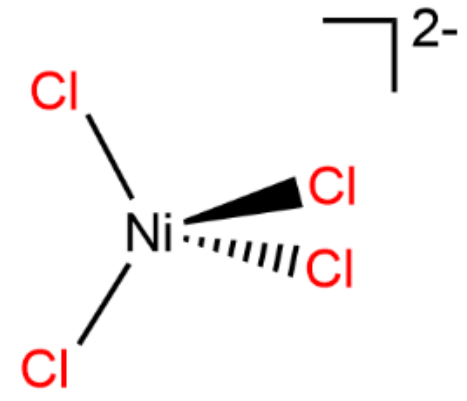	Cl
32	$[FeCl_4]^{-}$	tetrachloroferrate(III)	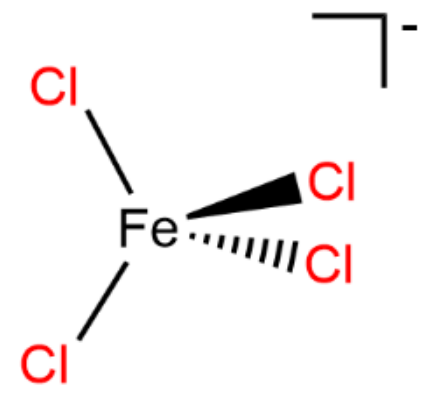	Cl
33	$[InCl_4]^{-}$	tetrachloroindate	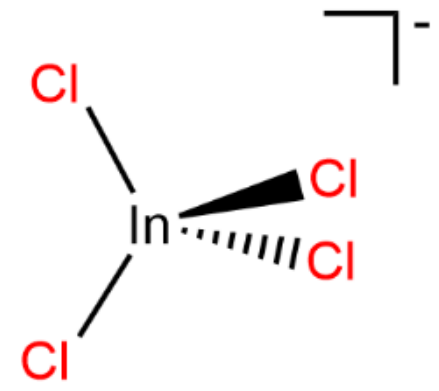	Cl
34	$[AlCl_4]^{-}$	tetrachloroaluminate	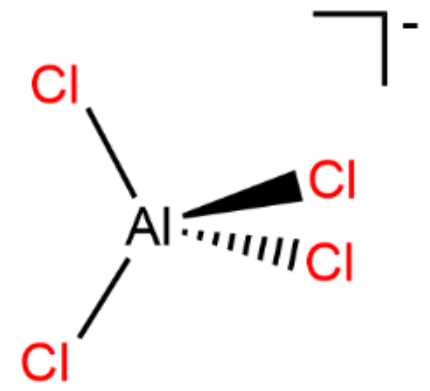	Cl

35	$[GaCl_4]^-$	tetrachlorogallate	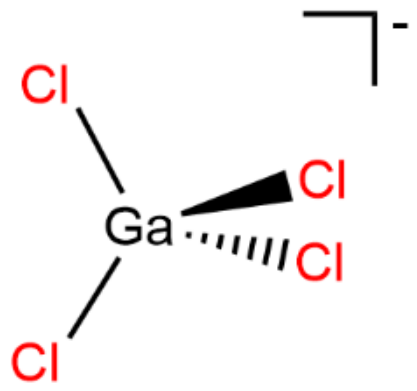	Cl	
36	$[SnCl_3]^-$	trichlorostannate	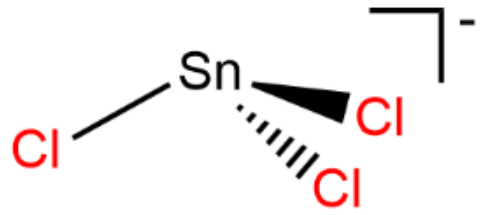	Cl	
37	$[LiCl_3]^{2-}$	trichlorolithate	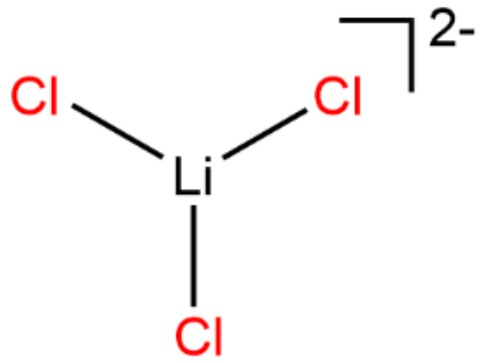	Cl	
38	$[Bi_2Cl_8]^{2-}$	octachlorodibismuthate	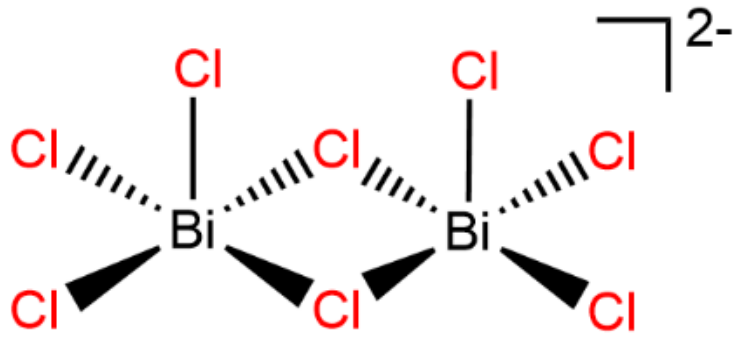	Cl	
39	$[Zn_2Cl_6]^{2-}$	hexachlorodizincate	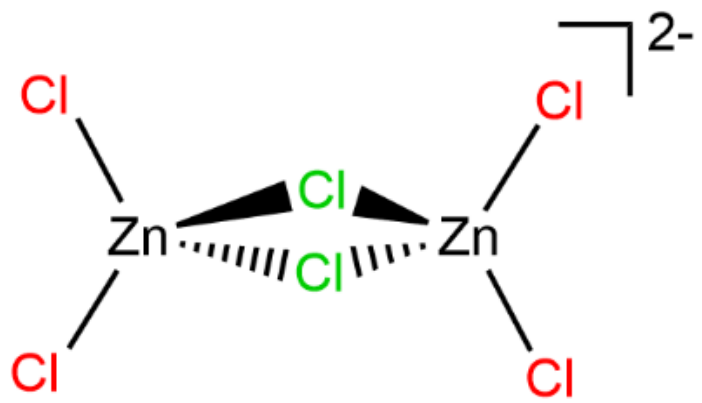	$Cl_{terminal}$	Cl_{bridge}
40	$[Zn_3Cl_8]^{2-}$	octachlorotrizincate	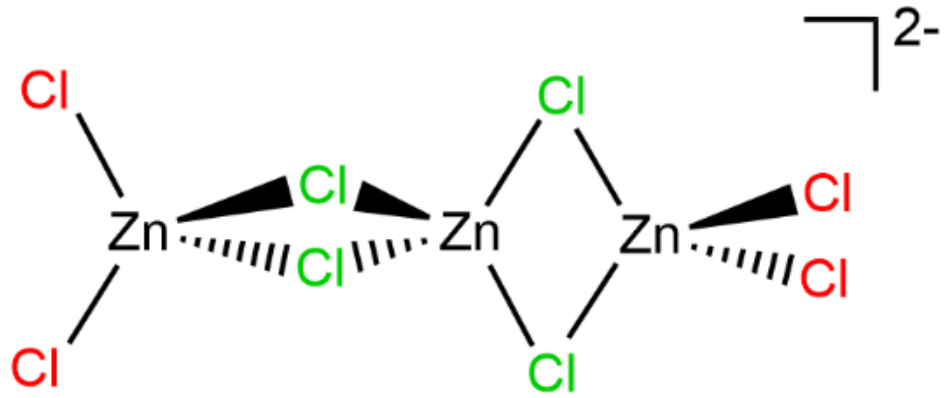	$Cl_{terminal}$	Cl_{bridge}

41	$[Zn_4Cl_{10}]^{2-}$	decachlorotetrazincate	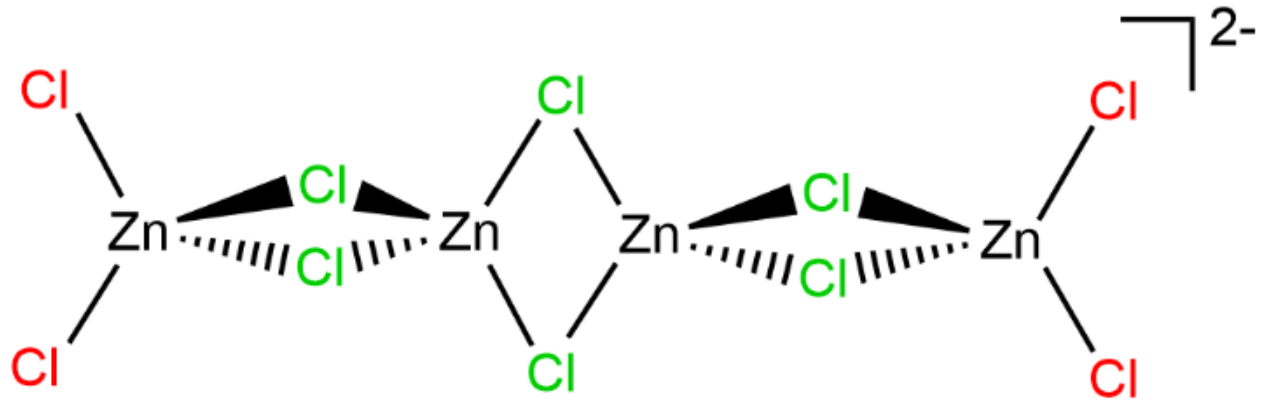 and 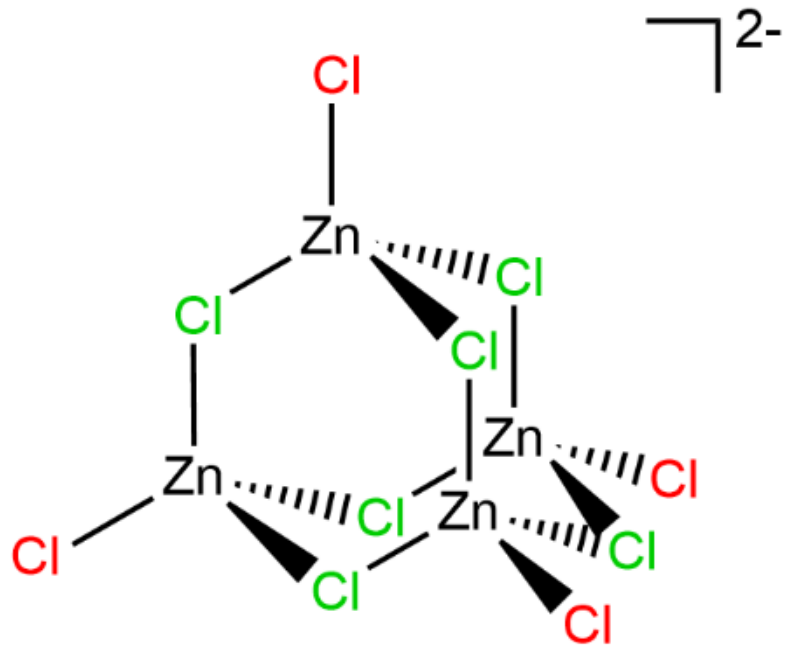	$Cl_{terminal}$ Cl_{bridge}
42	Br^-	bromide	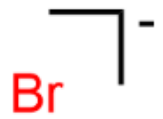	Br
43	$[ZnBr_4]^{2-}$	tetrabromozincate	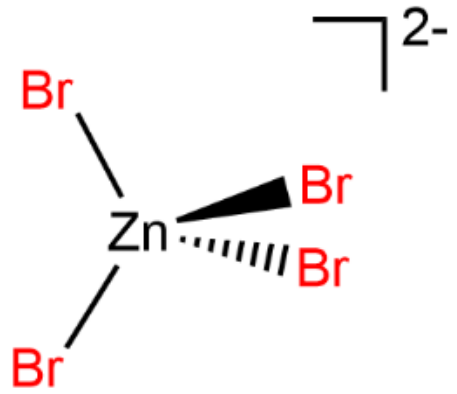	Br
44	$[CoBr_4]^{2-}$	tetrabromocobaltate	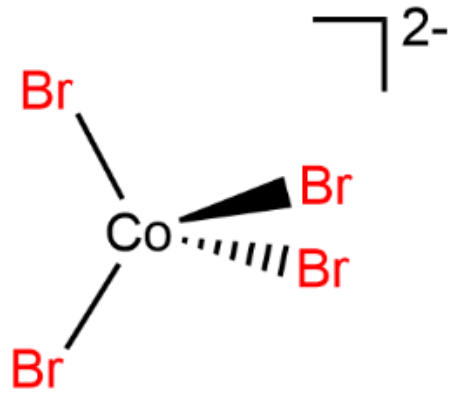	Br

45	$[InBr_4]^-$	tetrabromoindate	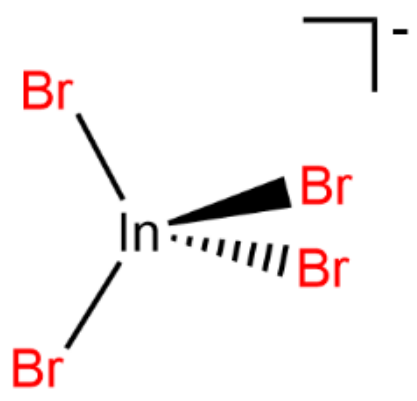	Br	
46	$[SnBr_3]^-$	tribromostannate	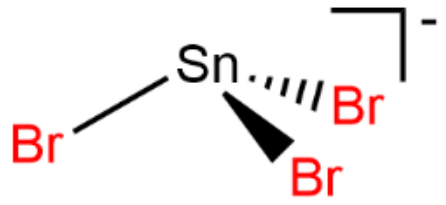	Br	
47	$[Zn_2Br_6]^{2-}$	hexabromodizincate	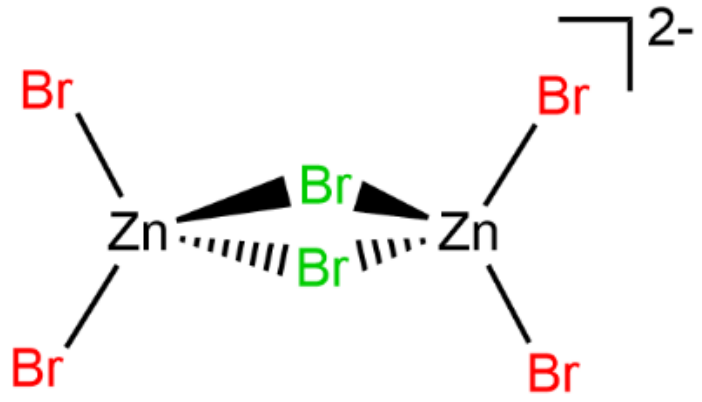	$Br_{terminal}$	Br_{bridge}
48	$[Zn_3Br_8]^{2-}$	octabromotrizincate	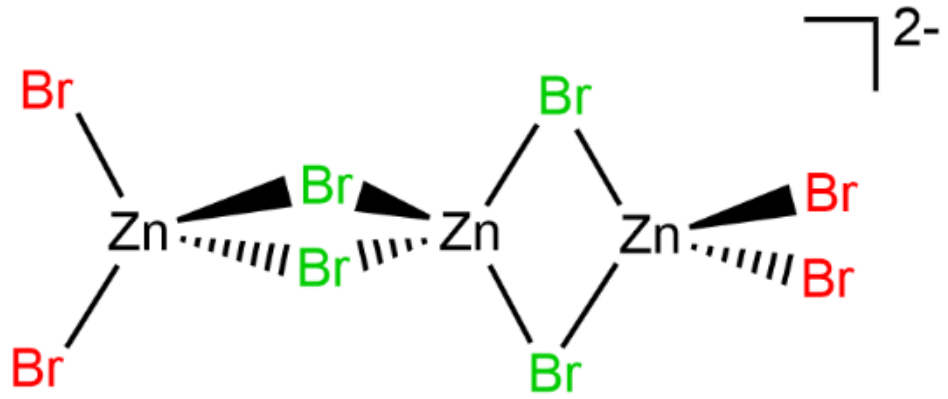	$Br_{terminal}$	Br_{bridge}
49	$[Zn_4Br_{10}]^{2-}$	decabromotetrazincate	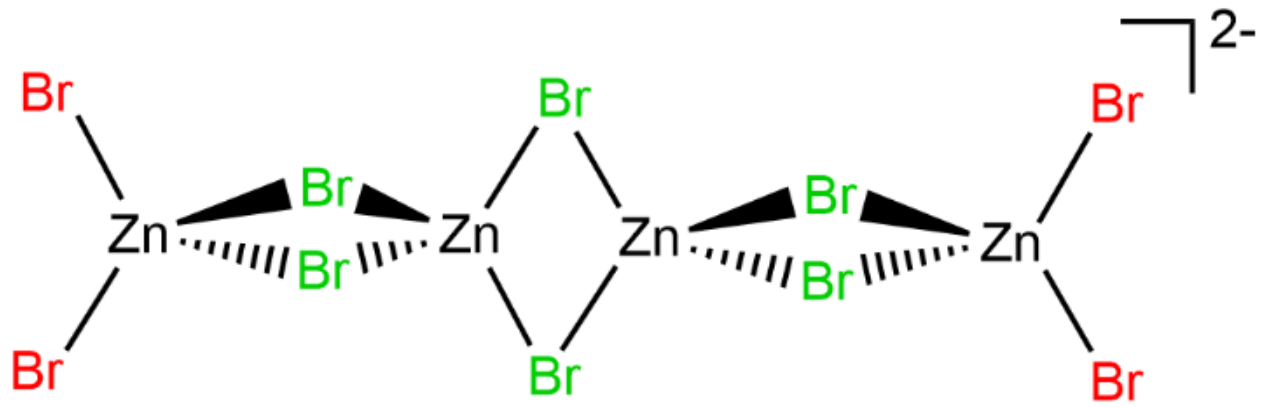 and	$Br_{terminal}$	Br_{bridge}

			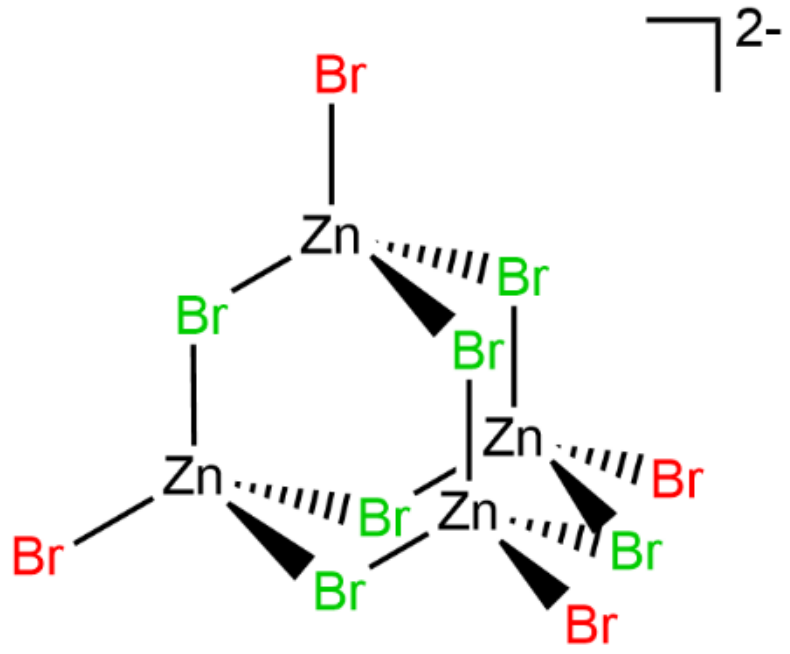		
50	I^-	iodide	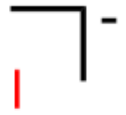	I	
51	$[I_3]^-$	triiodide	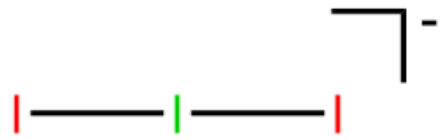	$I_{terminal}$	I_{bridge}
52	$[Co(NCS)_4]^{2-}$	tetrathiocyanatocobaltate	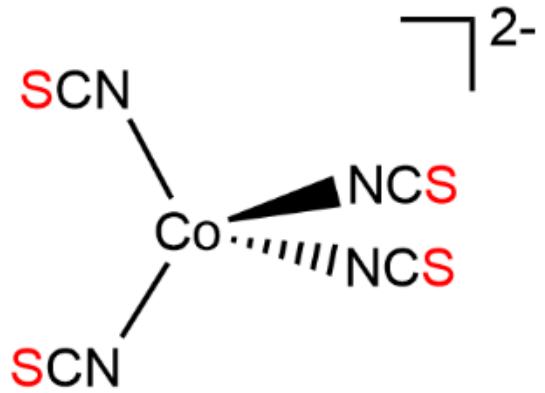	S	

Table S3. Structures, names and abbreviations of cations studied.

Cation no.	Abbreviation	Name	Structure
1	Li^+	lithium	
2	Na^+	sodium	
3	K^+	potassium	
4	Mg^{2+}	magnesium	
5	Ca^{2+}	calcium	
6	Zn^{2+}	zinc	
7	Al^{3+}	aluminium	
8	$[C_2C_1Im]^+$	1-ethyl-3-methylimidazolium	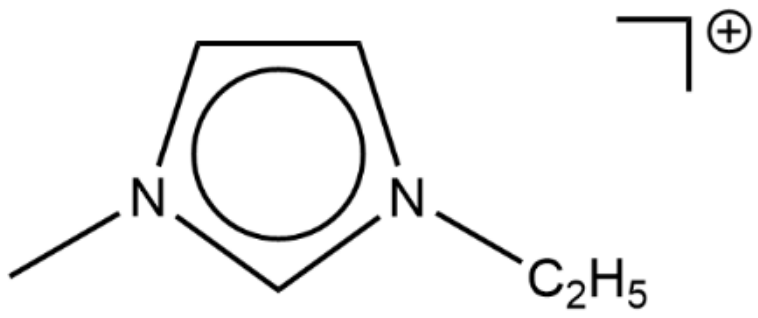
9	$[C_4C_1Im]^+$	1-*n*-butyl-3-methylimidazolium	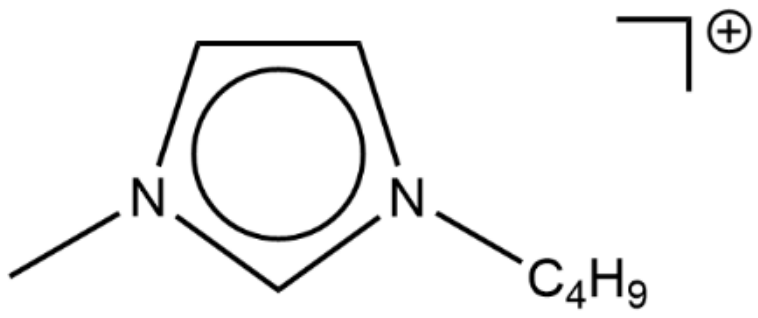
10	$[C_6C_1Im]^+$	1-*n*-hexyl-3-methylimidazolium	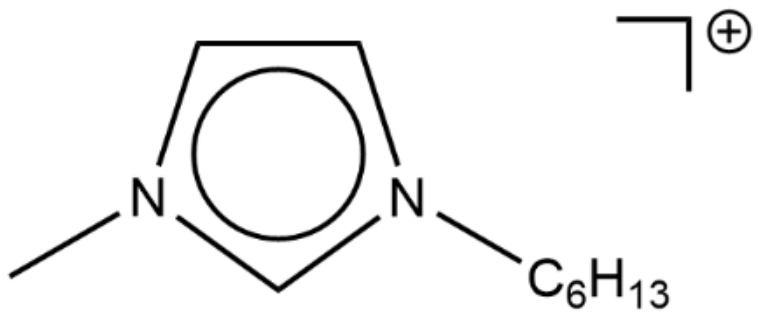
11	$[C_8C_1Im]^+$	1-*n*-octyl-3-methylimidazolium	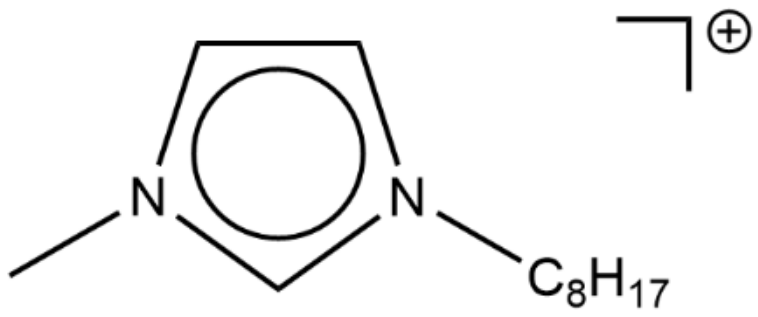

Table S4. Structures, names and abbreviations of molecular solvents studied.

Solvent no.	Abbreviation	Name	Structure
1	H_2O	water	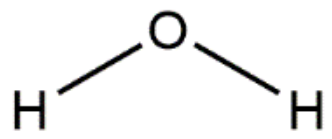
2	PC	propylene carbonate	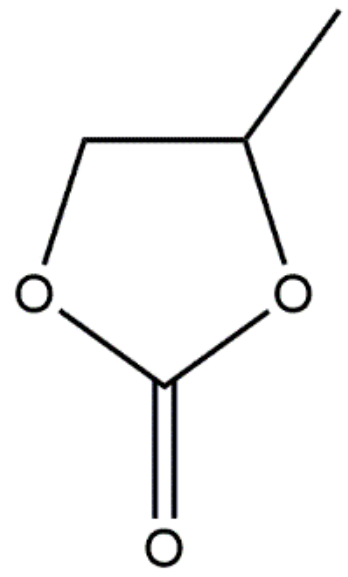
3	TEP	triethyl phosphate	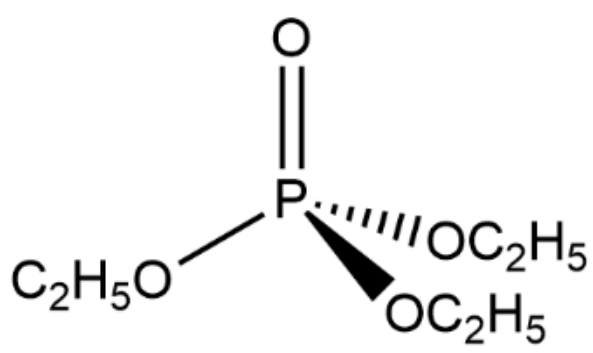

3. Methods. Anion-focused samples studied using XPS experiments

3.1. Methods. Samples studied using XPS experiments: anions in ionic liquid-based solutions

Table S5. Ionic liquids investigated in this work.

Ionic liquid no.	Abbreviation	Name	UCL Al source (Kα)	UCL Al source (Theta Probe)	Reading Al source	B07-B-XPS	Erlangen	Nottingham Kratos Al source	Nottingham Kratos Ag source
1	$[C_8C_1Im][NO_3]$	1-*n*-octyl-3-methylimidazolium nitrate					[8]		
2	$[C_4C_1Im][CH_3CO_2]$	1-*n*-butyl-3-methylimidazolium acetate			[9]				
3	$[C_2C_1Im][CH_3SO_3]$	1-ethyl-3-methylimidazolium methanesulfonate	[10]						
4	$[C_8C_1Im][HOSO_3]$	1-*n*-octyl-3-methylimidazolium hydrogensulfate	[10]						
5	$[C_4C_1Im][CH_3OSO_3]$	1-*n*-butyl-3-methylimidazolium methylsulfate	[10]						
6	$[C_4C_1Im][C_8H_{17}OSO_3]$	1-*n*-butyl-3-methylimidazolium octylsulfate	[10]						
7	$[C_4C_1Im][(CH_3O)_2PO_2]$	1-*n*-butyl-3-methylimidazolium dimethylphosphate	[10]						
8	$[C_8C_1Im][CF_3CO_2]$	1-*n*-octyl-3-methylimidazolium trifluoroacetate			[11]	Iolitec			
9	$[C_8C_1Im][CF_3SO_3]$	1-*n*-octyl-3-methylimidazolium trifluoromethylsulfonate	[10]						
10	$[C_8C_1Im][(CF_3SO_2)_2N]$	1-*n*-octyl-3-methylimidazolium bis[(trifluoromethane)sulfonyl]imide	[10]	Iolitec	Iolitec	Iolitec			[12]
11	$[C_8C_1Im][(FSO_2)_2N]$	1-*n*-octyl-3-methylimidazolium bis(fluorosulfonyl)imide			[11]				
12	$[C_4C_1Im][(C_2F_5SO_2)_2N]$	1-*n*-butyl-3-methylimidazolium bis(pentafluoroethylsulfonyl)imide			[9]	Iolitec			
13	$[C_8C_1Im][C(CN)_3]$	1-*n*-octyl-3-methylimidazolium tricyanomethanide	[10]						
14	$[C_6C_1Im][B(CN)_4]$	1-*n*-hexyl-3-methylimidazolium tetracyanoborate	[10]						
15	$[C_4C_1Im][N(CN)_2]$	1-*n*-butyl-3-methylimidazolium dicyanamide	[10]						

16	$[C_8C_1Im][SCN]$	1-*n*-octyl-3-methylimidazolium thiocyanate	[10]		Iolitec		
17	$[C_8C_1Im][BF_4]$	1-*n*-octyl-3-methylimidazolium tetrafluoroborate	[10]				
18	$[C_8C_1Im][PF_6]$	1-*n*-octyl-3-methylimidazolium hexafluorophosphate				Iolitec	
19	$[C_8C_1Im][SbF_6]$	1-*n*-octyl-3-methylimidazolium hexafluoroantimonate					[13, 14]
20	$[C_2C_1Im][(C_2F_5)_3PF_3]$	1-ethyl-3-methylimidazolium tris(pentafluoroethyl)-trifluorophosphate			[9]	Sigma-Aldrich	
21	$[C_8C_1Im]Cl$	1-*n*-octyl-3-methylimidazolium chloride		[14]		Iolitec	
22	$[C_8C_1Im]_2[ZnCl_4]$	1-*n*-octyl-3-methylimidazolium tetrachlorozincate		[9, 15]			
23	$[C_8C_1Im]_2[FeCl_4]$	1-*n*-octyl-3-methylimidazolium tetrachloroferrate(II)		[9]			
24	$[C_8C_1Im]_2[CoCl_4]$	1-*n*-octyl-3-methylimidazolium tetrachlorocobaltate		[9]			
25	$[C_8C_1Im]_2[NiCl_4]$	1-*n*-octyl-3-methylimidazolium tetrachloronickelate		[9]			
26	$[C_8C_1Im][FeCl_4]$	1-*n*-octyl-3-methylimidazolium tetrachloroferrate(III)					[16]
27	$[C_8C_1Im][InCl_4]$	1-*n*-octyl-3-methylimidazolium tetrachloroindate		[9]			
28	$[C_8C_1Im][SnCl_3]$	1-*n*-octyl-3-methylimidazolium trichlorostannate		[9]			
29	$[C_8C_1Im]_{11}[LiCl_3]Cl[(CF_3SO_2)_2N]_8$	1-*n*-octyl-3-methylimidazolium trichlorolithate / chloride / bis[(trifluoromethane)sulfonyl]imide				$Li[(CF_3SO_2)_2N]$ (Solvionic), $[C_8C_1Im]Cl$ and $[C_8C_1Im][(CF_3SO_2)_2N]$ (Iolitec)	
30	$[C_8C_1Im]_2[Bi_2Cl_8]$	1-*n*-octyl-3-methylimidazolium octachlorodibismuthate		[9]			
31	$[C_8C_1Im]_2[Zn_2Cl_6]$	1-*n*-octyl-3-methylimidazolium hexachlorodizincate		[9, 15]			
32	$[C_8C_1Im]_2[Zn_3Cl_8]$	1-*n*-octyl-3-methylimidazolium octachlorotrizincate			[15]		

33	$[C_8C_1Im]_2[Zn_4Cl_{10}]$	1-*n*-octyl-3-methylimidazolium decachlorotetrazincate		[9, 15]				
34	$[C_8C_1Im]Br$	1-*n*-octyl-3-methylimidazolium bromide		[14]		Iolitec		
35	$[C_8C_1Im]_2[ZnBr_4]$	1-*n*-octyl-3-methylimidazolium tetrabromozincate		[9, 15]				
36	$[C_8C_1Im]_2[CoBr_4]$	1-*n*-octyl-3-methylimidazolium tetrabromocobaltate		[9, 15]				
37	$[C_8C_1Im][InBr_4]$	1-*n*-octyl-3-methylimidazolium tetrabromoindate			[11, 17]			
38	$[C_8C_1Im][SnBr_3]$	1-*n*-octyl-3-methylimidazolium tribromostannate			[11, 17]			
39	$[C_8C_1Im]_2[Zn_2Br_6]$	1-*n*-octyl-3-methylimidazolium hexabromodizincate		[9, 15]				
40	$[C_8C_1Im]_2[Zn_3Br_8]$	1-*n*-octyl-3-methylimidazolium octabromotrizincate		[9, 15]				
41	$[C_8C_1Im]_2[Zn_4Br_{10}]$	1-*n*-octyl-3-methylimidazolium decabromotetrazincate		[9, 15]				
42	$[C_6C_1Im]I$	1-*n*-hexyl-3-methylimidazolium iodide	[10]					
43	$[C_8C_1Im][I_3]$	1-*n*-octyl-3-methylimidazolium triiodide						[14]
44	$[C_4C_1Im]_2[Co(NCS)_4]$	1-*n*-butyl-3-methylimidazolium tetrathiocyanatocobaltate					[18]	

3.2. Methods. Samples studied using XPS experiments: anions in water-based solutions

Table S6. Water-based solutions investigated in this work. SL = HIPPIE Solid-Liquid Endstation

Sample no.	Salt abbreviation	Salt name	Salt purchase	Salt conc. / M	Beamline
1	$[SCN][C_4C_1Im]$	1-*n*-butyl-3-methylimidazolium thiocyanate	Iolitec	0.50	SOL³PES
2	$[SCN]K$	potassium thiocyanate	Sigma-Aldrich	0.50	SOL³PES
3	$[N(CN)_2][C_4C_1Im]$	1-*n*-butyl-3-methylimidazolium dicyanamide	Iolitec	0.50	SOL³PES
4	$[CF_3SO_3]Na$	sodium trifluoromethylsulfonate	Sigma-Aldrich	0.40	HIPPIE (SL)
5	$[CF_3SO_3]_2Zn$	zinc trifluoromethylsulfonate	Sigma-Aldrich	0.40	HIPPIE (SL)
6	$[CF_3SO_3]_2Mg$	magnesium trifluoromethylsulfonate	Sigma-Aldrich	0.40	HIPPIE (SL)
7	$[CF_3SO_3]_2Ca$	calcium trifluoromethylsulfonate	Sigma-Aldrich	0.40	HIPPIE (SL)
8	$[CF_3SO_3]K$	potassium trifluoromethylsulfonate	Solvionic	0.15	GALAXIES
9	$[CF_3SO_3][C_4C_1Im]$	1-*n*-butyl-3-methylimidazolium trifluoromethylsulfonate	Iolitec	0.50	GALAXIES
10	$[BF_4][C_4C_1Im]$	1-*n*-butyl-3-methylimidazolium tetrafluoroborate	Iolitec	0.50	SOL³PES
11	$[PF_6]Li$	lithium hexafluorophosphate	Sigma-Aldrich	0.50	HIPPIE (SL)
12	$[(CF_3SO_2)_2N]Li$	lithium bis[(trifluoromethane)sulfonyl]imide	Sigma-Aldrich	0.50	HIPPIE (SL)
13	$[(CF_3SO_2)_2N]Li$	lithium bis[(trifluoromethane)sulfonyl]imide	Iolitec	0.50	SOL³PES
14	$[(CF_3SO_2)_2N]Li$	lithium bis[(trifluoromethane)sulfonyl]imide	Sigma-Aldrich	0.14	GALAXIES
15	$[(FSO_2)_2N]Na$	sodium bis(fluorosulfonyl)imide	Sigma-Aldrich	0.40	HIPPIE (SL)

3.3. Methods. Samples studied using XPS experiments: anions in PC-based solutions

Table S7. PC-based solutions investigated in this work. SL = HIPPIE Solid-Liquid Endstation

Sample no.	Salt abbreviation	Salt name	Salt purchase	Salt conc. / M	Beamline
1	$[SCN]K$	potassium thiocyanate	Sigma-Aldrich	0.50	SOL³PES
2	$[SCN][C_4C_1Im]$	1-*n*-butyl-3-methylimidazolium thiocyanate	Iolitec	0.50	SOL³PES
3	$[SCN][C_4C_1Im]$	1-*n*-butyl-3-methylimidazolium thiocyanate	Iolitec	0.14	GALAXIES
4	$[CF_3SO_3]Na$	sodium trifluoromethylsulfonate	Sigma-Aldrich	0.15	HIPPIE (SL)
5	$[PF_6]Li$	lithium hexafluorophosphate	Sigma-Aldrich	0.50	HIPPIE (SL)
6	$[(CF_3SO_2)_2N]Na$	sodium bis[(trifluoromethane)sulfonyl]imide	Sigma-Aldrich	0.30	HIPPIE (SL)
7	$[(CF_3SO_2)_2N]Li$	lithium bis[(trifluoromethane)sulfonyl]imide	Sigma-Aldrich	0.46	SOL³PES
8	$[(CF_3SO_2)_2N]Li$	lithium bis[(trifluoromethane)sulfonyl]imide	Sigma-Aldrich	0.14	GALAXIES
9	$[(FSO_2)_2N]Na$	sodium bis(fluorosulfonyl)imide	Sigma-Aldrich	0.30	HIPPIE (SL)

3.4. Methods. Samples studied using XPS experiments: anions in TEP-based solutions

Table S8. TEP-based solutions investigated in this work. SL = HIPPIE Solid-Liquid Endstation

Sample no.	Salt abbreviation	Salt name	Salt purchase	Salt conc. / M	Beamline
1	$[SCN][C_4C_1Im]$	1-*n*-butyl-3-methylimidazolium thiocyanate	Iolitec	0.18	HIPPIE (SL)
2	$[PF_6]Li$	lithium hexafluorophosphate	Sigma-Aldrich	0.50	HIPPIE (SL)
3	$[(CF_3SO_2)_2N]Na$	sodium bis[(trifluoromethane)sulfonyl]imide	Sigma-Aldrich	0.50	HIPPIE (SL)
4	$[(CF_3SO_2)_2N]Li$	lithium bis[(trifluoromethane)sulfonyl]imide	Sigma-Aldrich	0.48	HIPPIE (SL)
5	$[(FSO_2)_2N]Na$	sodium bis(fluorosulfonyl)imide	Sigma-Aldrich	0.50	HIPPIE (SL)

4. Methods. Samples studied using lone-anion SMD calculations

Table S9. Lone-anion-SMD calculations in this work. ✓ means published here for the first time, reference [19] where the calculations were first published for three of the anions included here.

Anion no.	Abbreviation	Ionic liquid SMD	Water SMD	PC SMD	TEP SMD
5	$[ClO_4]^-$	[19]			
10	$[B(C_2O_4)_2]^-$	✓			
18	$[(CH)_2N_2CH]^-$	✓			
19	$[C_6H_4N_2CH]^-$	✓			
22	$[CF_3(C_3N_2)(CN)_2]^-$	✓			
37	$[AlCl_4]^-$	[19]			
35	$[GaCl_4]^-$	[19]			
2	$[CH_3CO_2]^-$		✓		
11	$[CF_3CO_2]^-$		✓		
12	$[CF_3SO_3]^-$		✓	✓	✓
13	$[(CF_3SO_2)_2N]^-$		✓	✓	✓
14	$[(FSO_2)_2N]^-$		✓	✓	✓
20	$[N(CN)_2]^-$		✓		
21	$[SCN]^-$		✓	✓	✓
23	$[BF_4]^-$		✓		
24	$[PF_6]^-$		✓	✓	✓

5. Methods. Lone-anion-SMD DFT calculations

5.1. Structure optimization

Structures were optimized as individual, isolated anions (referred to as lone-anions). Structures optimized using SMD are referred to as SMD structures.

Table S10. Parameters used in the SMD model

Solvent	Relative permittivity, ε_r	Refractive index, *n*	Surface tension, γ / cal mol^{-1} Å^2)	Abraham acidity, *α*	Abraham basicity, *β*
$[C_4C_1Im][PF_6]$	11.40	1.4090	70.24	0.266	0.216
Water	78.36	N/A	N/A	N/A	N/A
PC	64.90	1.421	60.33727	0.00	0.40
TEP	13.01	1.405	43.48658	0.00	0.77

5.2. E_B(core,anion) correction for lone-anion ionic liquid SMD calculations

Table S11. Average E_B(correction) for each core-level for lone-anion ionic liquid SMD calculations

Core-level	Average E_B(correction) from lone-anion-SMD effectively charge referenced to Fermi level (taken from reference [9]) / eV	Standard error of the sample mean / eV	Average E_B(correction) from lone-anion-SMD effectively charge referenced to vacuum level / eV
F 1s	15.84	0.04	20.69
O 1s	12.12	0.04	16.97
N 1s	9.76	0.08	14.61
Cl $2p_{3/2}$	5.18	0.09	10.03
S $2p_{3/2}$	3.80	0.08	8.65

6. Methods. Producing calculated and “experimental” XP spectra

To produce calculated and “experimental” XP spectra for core-levels that have spin-orbit coupling (S 2p, Cl 2p), each E_B(S 2p) value was adjusted to produce E_B(S $2p_{3/2}$) and E_B(S $2p_{1/2}$) of the appropriate relative intensity (using E_B and area factors given in Table S12). Once E_B(S $2p_{3/2}$) and E_B(S $2p_{1/2}$) were obtained, a Gaussian-Lorentzian Product (GLP) function was applied to each E_B(S $2p_{3/2}$) and E_B(S $2p_{1/2}$) data point for each core-state using Equation S1 and then summed to produce calculated/”experimental” XP spectra. The mixing parameter, *m,* and function width, *F*, were set to the values given in Table S12. The spin-orbit coupling ΔE_B values for S 2p and Cl 2p come from fitting experimental core-level XP spectra for many samples and represent an average value of the ΔE_B values needed to obtain satisfactory fits when one electronic environment is present for the relevant element.

To produce calculated and “experimental” XP spectra for orbitals that do not have spin-orbit coupling (F 1s, O 1s, N 1s), a Gaussian-Lorentzian Product (GLP) function was applied to each E_B(core) data point for each valence-state using Equation S1 and then summed to produce calculated XP spectra. E_B represents E_B for each core-level and *x* is E_B at which the intensity is calculated. The mixing parameter, *m,* and function width, *F*, were set to the values given in Table S12.

$$\mathrm{GLP}\,(x; F, E_\mathrm{B}, m) = \frac{\exp\left[-4\ln 2(1-m)\frac{(x-E_\mathrm{B})^2}{F^2}\right]}{\left[1+4m\frac{(x-E_\mathrm{B})^2}{F^2}\right]} \qquad \text{Equation S1}$$

Table S12. Values used to produce calculated XP spectra and XP spectra from literature E_B(core,anion) values

**Orbital**	***F* (FWHM) / eV**	***m* (Gaussian-Lorentzian (GL) Product function)**	**Spin-orbit coupling ΔE_B / eV**	**E_B(calc.) with spin-orbit coupling correction**	**Spin-orbit coupling peak area ratio**	**Peak area ratio**
F 1s	1.60	GL(30)	N/A	N/A	N/A	N/A
O 1s	1.35	GL(30)	N/A	N/A	N/A	N/A
N 1s	1.05	GL(30)	N/A	N/A	N/A	N/A
S 2p	1.05	GL(30)	ΔE_B(S $2p_{3/2}$ - S $2p_{3/2}$) = 1.20 eV	E_B(S $2p_{3/2}$,calc.) = E_B(S 2p,calc.) – (1.20 × 2/3)<br><br>E_B(S $2p_{1/2}$,calc.) = E_B(S 2p,calc.) + (1.20 × 1/3)	1:2 for $2p_{1/2}$:$2p_{3/2}$	Area(S $2p_{3/2}$,calc.) = Area(S 2p,calc.) × 2/3<br><br>Area(S $2p_{1/2}$,calc.) = Area(S 2p,calc.) × 1/3
Cl 2p	0.90	GL(30)	ΔE_B(Cl $2p_{3/2}$ - Cl $2p_{3/2}$) = 1.60 eV	E_B(Cl $2p_{3/2}$,calc.) = E_B(Cl 2p,calc.) – (1.60 × 2/3)<br><br>E_B(Cl $2p_{1/2}$,calc.) = E_B(Cl 2p,calc.) + (1.60 × 1/3)	1:2 for $2p_{1/2}$:$2p_{3/2}$	Area(Cl $2p_{3/2}$,calc.) = Area(Cl 2p,calc.) × 2/3<br><br>Area(Cl $2p_{1/2}$,calc.) = Area(Cl 2p,calc.) × 1/3

7. Data analysis. Peak fitting core level XP spectra

Peak fitting core level XP spectra is important for charge referencing to obtain E_B(core,anion) values (ESI Sections 16-29) and demonstrating purity (ESI Sections 10-13). All core-level XP spectra were fitted using CASAXPS™ software. Spectra were fitted with a GL30 lineshape (70% Gaussian, 30% Lorentzian) and a Shirley/linear background. How the core level XP spectra were fitted is given in Table S13, along with any constraints used. The constraints used were mostly explained in the ESI of references [10, 15].

7.1. Peak fitting core level XP spectra: ionic liquids

Table S13. Fitting constraints used for core level X-ray photoelectron spectroscopy (XPS) for each ionic liquid

Ionic liquid no.	Abbreviation	Core-level	Fitting constraints used
1	$[C_8C_1Im][NO_3]$	C 1s	Peak area ratio 5:7 for C_{hetero}:C_{alkyl} [8]
2	$[C_4C_1Im][CH_3CO_2]$	C 1s	Peak area ratio 2:4:4 for C^2:C_{hetero}:C_{alkyl}
3	$[C_2C_1Im][CH_3SO_3]$	C 1s<br>S 2p	Peak area ratio 1:4:2 for C^2:C_{hetero}:C_{alkyl}<br>Peak area ratio 1:2 for $2p_{1/2}$:$2p_{3/2}$
4	$[C_8C_1Im][HOSO_3]$	C 1s<br>S 2p	Peak area ratio 1:4:7 for C^2:C_{hetero}:C_{alkyl}<br>Peak area ratio 1:2 for $2p_{1/2}$:$2p_{3/2}$
5	$[C_4C_1Im][CH_3OSO_3]$	C 1s<br>S 2p	Peak area ratio 1:5:3 for C^2:C_{hetero}:C_{alkyl}<br>Peak area ratio 1:2 for $2p_{1/2}$:$2p_{3/2}$
6	$[C_4C_1Im][C_8H_{17}OSO_3]$	C 1s<br>S 2p	Peak area ratio 1:5:10 for C^2:C_{hetero}:C_{alkyl}<br>Peak area ratio 1:2 for $2p_{1/2}$:$2p_{3/2}$
7	$[C_4C_1Im][(CH_3O)_2PO_2]$	C 1s<br>P 2p	Peak area ratio 1:6:3 for C^2:C_{hetero}:C_{alkyl}<br>Peak area ratio 1:2 for $2p_{1/2}$:$2p_{3/2}$
8	$[C_8C_1Im][CF_3CO_2]$	C 1s	Peak area ratio 1:4:7 for C^2:C_{hetero}:C_{alkyl}
9	$[C_8C_1Im][CF_3SO_3]$	C 1s<br>S 2p	Peak area ratio 1:4:7 for C^2:C_{hetero}:C_{alkyl}<br>Peak area ratio 1:2 for $2p_{1/2}$:$2p_{3/2}$
10	$[C_8C_1Im][(CF_3SO_2)_2N]$	C 1s<br>S 2p	Peak area ratio 1:4:7 for C^2:C_{hetero}:C_{alkyl}<br>Peak area ratio 1:2 for $2p_{1/2}$:$2p_{3/2}$
11	$[C_8C_1Im][(FSO_2)_2N]$	C 1s<br>S 2p	Peak area ratio 1:4:7 for C^2:C_{hetero}:C_{alkyl}<br>Peak area ratio 1:2 for $2p_{1/2}$:$2p_{3/2}$
12	$[C_4C_1Im][(C_2F_5SO_2)_2N]$	C 1s<br>S 2p	Peak area ratio 1:4:3 for C^2:C_{hetero}:C_{alkyl}<br>Peak area ratio 1:2 for $2p_{1/2}$:$2p_{3/2}$
13	$[C_8C_1Im][C(CN)_3]$	C 1s	Peak area ratio 1:8:7 for C^2:C_{hetero}:C_{alkyl}
14	$[C_6C_1Im][B(CN)_4]$	C 1s	Peak area ratio 1:8:5 for C^2:C_{hetero}:C_{alkyl}
15	$[C_4C_1Im][N(CN)_2]$	C 1s	Peak area ratio 1:6:3 for C^2:C_{hetero}:C_{alkyl}<br>FWHM(C^2) = FWHM(C_{hetero})
16	$[C_8C_1Im][SCN]$	C 1s<br>S 2p	Peak area ratio 1:5:7 for C^2:C_{hetero}:C_{alkyl}<br>Peak area ratio 1:2 for $2p_{1/2}$:$2p_{3/2}$
17	$[C_8C_1Im][BF_4]$	C 1s	Peak area ratio 1:4:7 for C^2:C_{hetero}:C_{alkyl}
18	$[C_8C_1Im][PF_6]$	C 1s<br>P 2p	Peak area ratio 1:4:7 for C^2:C_{hetero}:C_{alkyl}<br>Peak area ratio 1:2 for $2p_{1/2}$:$2p_{3/2}$
19	$[C_8C_1Im][SbF_6]$	C 1s	Peak area ratio 1:4:7 for C^2:C_{hetero}:C_{alkyl}
20	$[C_2C_1Im][(C_2F_5)_3PF_3]$	C 1s	Peak area ratio 1:4:1 for C^2:C_{hetero}:C_{alkyl}
21	$[C_8C_1Im]Cl$	C 1s<br>Cl 2p	Peak area ratio 1:4:7 for C^2:C_{hetero}:C_{alkyl}<br>Peak area ratio 1:2 for $2p_{1/2}$:$2p_{3/2}$
22	$[C_8C_1Im]_2[ZnCl_4]$	C 1s<br>Cl 2p	Peak area ratio 1:4:7 for C^2:C_{hetero}:C_{alkyl}<br>Peak area ratio 1:2 for $2p_{1/2}$:$2p_{3/2}$<br>ΔE_B(Cl $2p_{3/2}$ - Cl $2p_{3/2}$) = 1.60 eV
23	$[C_8C_1Im]_2[FeCl_4]$	C 1s<br>Cl 2p	Peak area ratio 1:4:7 for C^2:C_{hetero}:C_{alkyl}<br>Peak area ratio 1:2 for $2p_{1/2}$:$2p_{3/2}$<br>ΔE_B(Cl $2p_{3/2}$ - Cl $2p_{3/2}$) = 1.60 eV
24	$[C_8C_1Im]_2[CoCl_4]$	C 1s<br>Cl 2p	Peak area ratio 1:4:7 for C^2:C_{hetero}:C_{alkyl}<br>Peak area ratio 1:2 for $2p_{1/2}$:$2p_{3/2}$<br>ΔE_B(Cl $2p_{3/2}$ - Cl $2p_{3/2}$) = 1.60 eV
25	$[C_8C_1Im]_2[NiCl_4]$	C 1s<br>Cl 2p	Peak area ratio 1:4:7 for C^2:C_{hetero}:C_{alkyl}<br>Peak area ratio 1:2 for $2p_{1/2}$:$2p_{3/2}$<br>ΔE_B(Cl $2p_{3/2}$ - Cl $2p_{3/2}$) = 1.60 eV
26	$[C_8C_1Im][FeCl_4]$	C 1s	Peak area ratio 1:4:7 for C^2:C_{hetero}:C_{alkyl}

		Cl 2p	Peak area ratio 1:2 for $2p_{1/2}:2p_{3/2}$<br>ΔE_B(Cl $2p_{3/2}$ - Cl $2p_{3/2}$) = 1.60 eV
27	$[C_8C_1Im][InCl_4]$	C 1s<br>Cl 2p	Peak area ratio 1:4:7 for $C^2:C_{hetero}:C_{alkyl}$<br>Peak area ratio 1:2 for $2p_{1/2}:2p_{3/2}$<br>ΔE_B(Cl $2p_{3/2}$ - Cl $2p_{3/2}$) = 1.60 eV
28	$[C_8C_1Im][SnCl_3]$	C 1s<br>Cl 2p	Peak area ratio 1:4:7 for $C^2:C_{hetero}:C_{alkyl}$<br>Peak area ratio 1:2 for $2p_{1/2}:2p_{3/2}$<br>ΔE_B(Cl $2p_{3/2}$ - Cl $2p_{3/2}$) = 1.60 eV
29	$[C_8C_1Im]_{11}[LiCl_3]Cl[(CF_3SO_2)_2N]_8$	C 1s<br>S 2p<br>Cl 2p	Peak area ratio 1:4:7 for $C^2:C_{hetero}:C_{alkyl}$<br>Peak area ratio 1:2 for $2p_{1/2}:2p_{3/2}$<br>Peak area ratio 1:2 for $2p_{1/2}:2p_{3/2}$
30	$[C_8C_1Im]_2[Bi_2Cl_8]$	C 1s<br>Cl 2p	Peak area ratio 1:4:7 for $C^2:C_{hetero}:C_{alkyl}$<br>Peak area ratio 1:2 for $2p_{1/2}:2p_{3/2}$
31	$[C_8C_1Im]_2[Zn_2Cl_6]$	C 1s<br>Cl 2p	Peak area ratio 1:4:7 for $C^2:C_{hetero}:C_{alkyl}$<br>Peak area ratio 1:2 for $2p_{1/2}:2p_{3/2}$<br>Peak area ratio 1:2 for $Cl_{bridging}:Cl_{terminal}$<br>$\Delta E_B$(Cl $2p_{3/2}$ - Cl $2p_{3/2}$) = 1.60 eV<br>FWHM($Cl_{terminal}$ $2p_{3/2}$) = FWHM($Cl_{terminal}$ $2p_{1/2}$)<br>FWHM($Cl_{bridging}$ $2p_{3/2}$) = FWHM($Cl_{bridging}$ $2p_{1/2}$)
32	$[C_8C_1Im]_2[Zn_3Cl_8]$	C 1s<br>Cl 2p	Peak area ratio 1:4:7 for $C^2:C_{hetero}:C_{alkyl}$<br>Peak area ratio 1:2 for $2p_{1/2}:2p_{3/2}$<br>Peak area ratio 1:1 for $Cl_{bridging}:Cl_{terminal}$<br>$\Delta E_B$(Cl $2p_{3/2}$ - Cl $2p_{3/2}$) = 1.60 eV<br>FWHM($Cl_{terminal}$ $2p_{3/2}$) = FWHM($Cl_{terminal}$ $2p_{1/2}$)<br>FWHM($Cl_{bridging}$ $2p_{3/2}$) = FWHM($Cl_{bridging}$ $2p_{1/2}$)
33	$[C_8C_1Im]_2[Zn_4Cl_{10}]$	C 1s<br>Cl 2p	Peak area ratio 1:4:7 for $C^2:C_{hetero}:C_{alkyl}$<br>Peak area ratio 1:2 for $2p_{1/2}:2p_{3/2}$<br>Peak area ratio 3:2 for $Cl_{bridging}:Cl_{terminal}$<br>$\Delta E_B$(Cl $2p_{3/2}$ - Cl $2p_{3/2}$) = 1.60 eV<br>FWHM($Cl_{terminal}$ $2p_{3/2}$) = FWHM($Cl_{terminal}$ $2p_{1/2}$)<br>FWHM($Cl_{bridging}$ $2p_{3/2}$) = FWHM($Cl_{bridging}$ $2p_{1/2}$)
34	$[C_8C_1Im]Br$	C 1s<br>Br 3d	Peak area ratio 1:4:7 for $C^2:C_{hetero}:C_{alkyl}$<br>Peak area ratio 2:3 for $3d_{3/2}:3d_{5/2}$
35	$[C_8C_1Im]_2[ZnBr_4]$	C 1s<br>Br 3d	Peak area ratio 1:4:7 for $C^2:C_{hetero}:C_{alkyl}$<br>Peak area ratio 2:3 for $3d_{3/2}:3d_{5/2}$<br>ΔE_B(Br $3d_{5/2}$ - Br $3d_{3/2}$) = 1.04 eV
36	$[C_8C_1Im]_2[CoBr_4]$	C 1s<br>Br 3d	Peak area ratio 1:4:7 for $C^2:C_{hetero}:C_{alkyl}$<br>Peak area ratio 2:3 for $3d_{3/2}:3d_{5/2}$<br>ΔE_B(Br $3d_{5/2}$ - Br $3d_{3/2}$) = 1.04 eV
37	$[C_8C_1Im][InBr_4]$	C 1s<br>Br 3d	Peak area ratio 1:4:7 for $C^2:C_{hetero}:C_{alkyl}$<br>Peak area ratio 2:3 for $3d_{3/2}:3d_{5/2}$<br>ΔE_B(Br $3d_{5/2}$ - Br $3d_{3/2}$) = 1.04 eV
38	$[C_8C_1Im][SnBr_3]$	C 1s<br>Br 3d	Peak area ratio 1:4:7 for $C^2:C_{hetero}:C_{alkyl}$<br>Peak area ratio 2:3 for $3d_{3/2}:3d_{5/2}$<br>ΔE_B(Br $3d_{5/2}$ - Br $3d_{3/2}$) = 1.04 eV
39	$[C_8C_1Im]_2[Zn_2Br_6]$	C 1s<br>Br 3d	Peak area ratio 1:4:7 for $C^2:C_{hetero}:C_{alkyl}$<br>Peak area ratio 2:3 for $3d_{3/2}:3d_{5/2}$<br>Peak area ratio 1:2 for $Br_{bridging}:Br_{terminal}$<br>$\Delta E_B$(Br $3d_{5/2}$ - Br $3d_{3/2}$) = 1.04 eV<br>FWHM($Br_{terminal}$ $3d_{5/2}$) = FWHM($Br_{terminal}$ $3d_{3/2}$)<br>FWHM($Br_{bridging}$ $3d_{5/2}$) = FWHM($Br_{bridging}$ $3d_{3/2}$)
40	$[C_8C_1Im]_2[Zn_3Br_8]$	C 1s<br>Br 3d	Peak area ratio 1:4:7 for $C^2:C_{hetero}:C_{alkyl}$<br>Peak area ratio 2:3 for $3d_{3/2}:3d_{5/2}$<br>Peak area ratio 1:1 for $Br_{bridging}:Br_{terminal}$<br>$\Delta E_B$(Br $3d_{5/2}$ - Br $3d_{3/2}$) = 1.04 eV<br>FWHM($Br_{terminal}$ $3d_{5/2}$) = FWHM($Br_{terminal}$ $3d_{3/2}$)<br>FWHM($Br_{bridging}$ $3d_{5/2}$) = FWHM($Br_{bridging}$ $3d_{3/2}$)
41	$[C_8C_1Im]_2[Zn_4Br_{10}]$	C 1s<br>Br 3d	Peak area ratio 1:4:7 for $C^2:C_{hetero}:C_{alkyl}$<br>Peak area ratio 2:3 for $3d_{3/2}:3d_{5/2}$<br>Peak area ratio 3:2 for $Br_{bridging}:Br_{terminal}$<br>$\Delta E_B$(Br $3d_{5/2}$ - Br $3d_{3/2}$) = 1.04 eV<br>FWHM($Br_{terminal}$ $3d_{5/2}$) = FWHM($Br_{terminal}$ $3d_{3/2}$)

			FWHM($Br_{bridging}$ $3d_{5/2}$) = FWHM($Br_{bridging}$ $3d_{3/2}$)
42	$[C_6C_1Im]I$	C 1s	1:4:5 for C^2:C_{hetero}:C_{alkyl}
43	$[C_8C_1Im][I_3]$	C 1s	1:4:7 for C^2:C_{hetero}:C_{alkyl}
44	$[C_4C_1Im]_2[Co(NCS)_4]$	C 1s	None provided in reference [18]

7.2. Peak fitting core level XP spectra: water

Table S14. Water-based solutions investigated in this work.

Sample no.	Salt abbreviation	Core-level	Fitting constraints used
1	$[SCN][C_4C_1Im]$	C 1s<br>S 2p	Peak area ratio 1:5:3 for C^2:C_{hetero}:C_{alkyl}<br>Peak area ratio 1:2 for $2p_{1/2}$:$2p_{3/2}$
2	[SCN]K	S 2p	Peak area ratio 1:2 for $2p_{1/2}$:$2p_{3/2}$
3	$[N(CN)_2][C_4C_1Im]$	C 1s	Peak area ratio 1:6:3 for C^2:C_{hetero}:C_{alkyl}
4	$[CF_3SO_3]Na$	S 2p	Peak area ratio 1:2 for $2p_{1/2}$:$2p_{3/2}$
5	$[CF_3SO_3]_2Zn$	S 2p	Peak area ratio 1:2 for $2p_{1/2}$:$2p_{3/2}$
6	$[CF_3SO_3]_2Mg$	S 2p	Peak area ratio 1:2 for $2p_{1/2}$:$2p_{3/2}$
7	$[CF_3SO_3]_2Ca$	S 2p	Peak area ratio 1:2 for $2p_{1/2}$:$2p_{3/2}$
8	$[CF_3SO_3]K$	S 2p	Peak area ratio 1:2 for $2p_{1/2}$:$2p_{3/2}$
9	$[CF_3SO_3][C_4C_1Im]$	C 1s<br>S 2p	Peak area ratio 1:4:3 for C^2:C_{hetero}:C_{alkyl}<br>Peak area ratio 1:2 for $2p_{1/2}$:$2p_{3/2}$
10	$[BF_4][C_4C_1Im]$	C 1s	Peak area ratio 1:4:3 for C^2:C_{hetero}:C_{alkyl}
11	$[PF_6]Li$	P 2p	Peak area ratio 1:2 for $2p_{1/2}$:$2p_{3/2}$
12	$[(CF_3SO_2)_2N]Li$	S 2p	Peak area ratio 1:2 for $2p_{1/2}$:$2p_{3/2}$
13	$[(CF_3SO_2)_2N]Li$	S 2p	Peak area ratio 1:2 for $2p_{1/2}$:$2p_{3/2}$
14	$[(CF_3SO_2)_2N]Li$	S 2p	Peak area ratio 1:2 for $2p_{1/2}$:$2p_{3/2}$
15	$[(FSO_2)_2N]Na$	S 2p	Peak area ratio 1:2 for $2p_{1/2}$:$2p_{3/2}$

7.3. Peak fitting core level XP spectra: PC

Table S15. PC-based solutions investigated in this work.

Sample no.	Salt abbreviation	Core-level	Fitting constraints used
1	[SCN]K	S 2p	Peak area ratio 1:2 for $2p_{1/2}$:$2p_{3/2}$
2	$[SCN][C_4C_1Im]$	S 2p	Peak area ratio 1:2 for $2p_{1/2}$:$2p_{3/2}$
3	$[SCN][C_4C_1Im]$	S 2p	Peak area ratio 1:2 for $2p_{1/2}$:$2p_{3/2}$
4	$[CF_3SO_3]Na$	S 2p	Peak area ratio 1:2 for $2p_{1/2}$:$2p_{3/2}$
5	$[PF_6]Li$	P 2p	Peak area ratio 1:2 for $2p_{1/2}$:$2p_{3/2}$
6	$[(CF_3SO_2)_2N]Na$	S 2p	Peak area ratio 1:2 for $2p_{1/2}$:$2p_{3/2}$
7	$[(CF_3SO_2)_2N]Li$	S 2p	Peak area ratio 1:2 for $2p_{1/2}$:$2p_{3/2}$
8	$[(CF_3SO_2)_2N]Li$	S 2p	Peak area ratio 1:2 for $2p_{1/2}$:$2p_{3/2}$
9	$[(FSO_2)_2N]Na$	S 2p	Peak area ratio 1:2 for $2p_{1/2}$:$2p_{3/2}$

7.4. Peak fitting core level XP spectra: TEP

Table S16. TEP-based solutions investigated in this work.

Sample no.	Salt abbreviation	Core-level	Fitting constraints used
1	$[SCN][C_4C_1Im]$	O 1s	Peak area ratio 3:1 for $O_{POC,liquid}$:$O_{P=O,liquid}$
			Peak area ratio 3:1 for $O_{POC,gas}$:$O_{P=O,gas}$
			FWHM($O_{POC,gas}$) = FWHM($O_{P=O,gas}$) = 0.80 eV
		C 1s	Peak area ratio 1:1 for $C_{alkyl,liquid}$:$C_{CO,liquid}$
			Peak area ratio 1:1 for $C_{alkyl,gas}$:$C_{CO,gas}$
			FWHM($C_{alkyl,liquid}$) = FWHM($C_{CO,liquid}$)
			FWHM($C_{alkyl,gas}$) = FWHM($C_{CO,gas}$) = 0.71 eV
		S 2p	Peak area ratio 1:2 for $2p_{1/2}$:$2p_{3/2}$
		P 2p	Peak area ratio 1:2 for $2p_{1/2}$:$2p_{3/2}$ (liquid)
			Peak area ratio 1:2 for $2p_{1/2}$:$2p_{3/2}$ (gas)
			FWHM(P $2p_{3/2,gas}$) = FWHM(P $2p_{1/2,gas}$) = 0.71 eV
2	$[PF_6]Li$	O 1s	Peak area ratio 3:1 for $O_{POC,liquid}$:$O_{P=O,liquid}$
			Peak area ratio 3:1 for $O_{POC,gas}$:$O_{P=O,gas}$
			FWHM($O_{POC,gas}$) = FWHM($O_{P=O,gas}$)
		C 1s	Peak area ratio 1:1 for $C_{alkyl,liquid}$:$C_{CO,liquid}$
			Peak area ratio 1:1 for $C_{alkyl,gas}$:$C_{CO,gas}$
			FWHM($C_{alkyl,liquid}$) = FWHM($C_{CO,liquid}$)
			FWHM($C_{alkyl,gas}$) = FWHM($C_{CO,gas}$) = 0.71 eV
		P 2p	Peak area ratio 1:2 for $2p_{1/2,liquid}$:$2p_{3/2,liquid}$
			Peak area ratio 1:2 for $2p_{1/2,gas}$:$2p_{3/2,gas}$
			FWHM(P $2p_{3/2,liquid}$) = FWHM(P $2p_{1/2,liquid}$)
			FWHM(P $2p_{3/2,gas/PF6}$) = FWHM(P $2p_{1/2,gas/PF6}$)
3	$[(CF_3SO_2)_2N]Na$	O 1s	Peak area ratio 3:1 for $O_{POC,liquid}$:$O_{P=O,liquid}$
			Peak area ratio 3:1 for $O_{POC,gas}$:$O_{P=O,gas}$
			FWHM($O_{POC,gas}$) = FWHM($O_{P=O,gas}$)
		C 1s	Peak area ratio 1:1 for $C_{alkyl,liquid}$:$C_{CO,liquid}$
			Peak area ratio 1:1 for $C_{alkyl,gas}$:$C_{CO,gas}$
			FWHM($C_{alkyl,liquid}$) = FWHM($C_{CO,liquid}$)
			FWHM($C_{alkyl,gas}$) = FWHM($C_{CO,gas}$) = 0.71 eV
		S 2p	Peak area ratio 1:2 for $2p_{1/2}$:$2p_{3/2}$
		P 2p	Peak area ratio 1:2 for $2p_{1/2,liquid}$:$2p_{3/2,liquid}$
			No peaks due to gas-phase TEP were fitted
4	$[(CF_3SO_2)_2N]Li$	O 1s	Peak area ratio 3:1 for $O_{POC,liquid}$:$O_{P=O,liquid}$
			Peak area ratio 3:1 for $O_{POC,gas}$:$O_{P=O,gas}$
		C 1s	Peak area ratio 1:1 for $C_{alkyl,liquid}$:$C_{CO,liquid}$
			Peak area ratio 1:1 for $C_{alkyl,gas}$:$C_{CO,gas}$
			FWHM($C_{alkyl,liquid}$) = FWHM($C_{CO,liquid}$)
			FWHM($C_{alkyl,gas}$) = FWHM($C_{CO,gas}$) = 0.71 eV
		S 2p	Peak area ratio 1:2 for $2p_{1/2}$:$2p_{3/2}$
		P 2p	Peak area ratio 1:2 for $2p_{1/2,liquid}$:$2p_{3/2,liquid}$
			No peaks due to gas-phase TEP were fitted
5	$[(FSO_2)_2N]Na$	O 1s	Peak area ratio 3:1 for $O_{POC,liquid}$:$O_{P=O,liquid}$
			Peak area ratio 3:1 for $O_{POC,gas}$:$O_{P=O,gas}$
			FWHM($O_{POC,liquid}$) = FWHM($O_{P=O,liquid}$)
			FWHM($O_{POC,gas}$) = FWHM($O_{P=O,gas}$)
		C 1s	Peak area ratio 1:1 for $C_{alkyl,liquid}$:$C_{CO,liquid}$
			Peak area ratio 1:1 for $C_{alkyl,gas}$:$C_{CO,gas}$
			FWHM($C_{alkyl,liquid}$) = FWHM($C_{CO,liquid}$)
			FWHM($C_{alkyl,gas}$) = FWHM($C_{CO,gas}$) = 0.71 eV
		S 2p	Peak area ratio 1:2 for $2p_{1/2}$:$2p_{3/2}$
		P 2p	Peak area ratio 1:2 for $2p_{1/2,liquid}$:$2p_{3/2,liquid}$
			No peaks due to gas-phase TEP were fitted

8. Data analysis. Charge referencing and area normalization

8.1. Charge referencing molecular solvent XPS

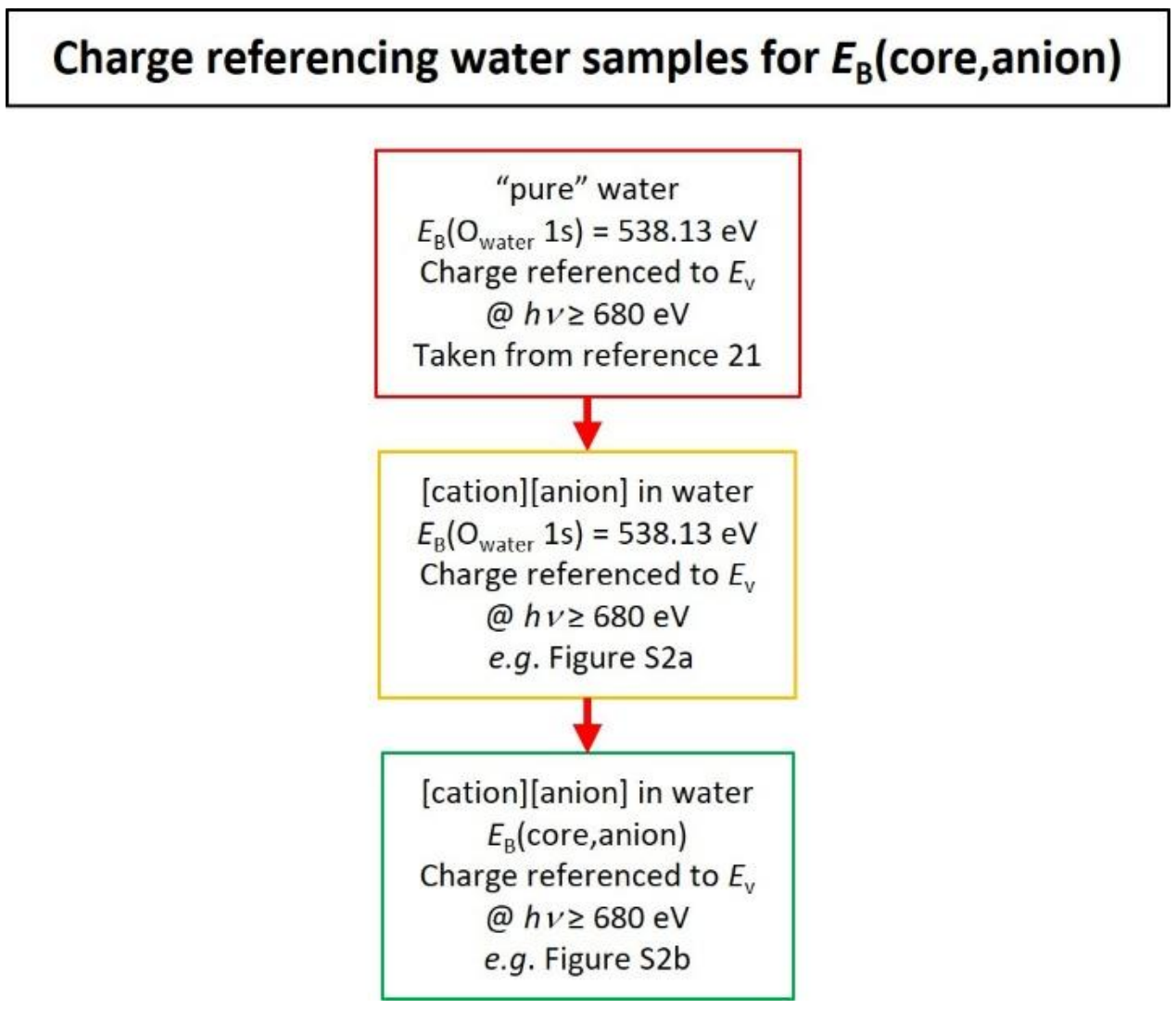

Figure S1. Flow-chart for charge referencing anions solvated in water-based samples.

Table S17. Charge referencing core level X-ray photoelectron spectroscopy (XPS) for water samples

Sample no.	Salt abbreviation	Core-level used for charge referencing	E_B(core) used to effectively charge reference to vacuum level / eV	Rationale for choosing core-level used for charge referencing
1	[SCN][C_4C_1Im]	O_{liquid} 1s	538.13	Taken from reference [20]
2	[SCN]K	O_{liquid} 1s	538.13	Taken from reference [20]
3	[$N(CN)_2$][C_4C_1Im]	O_{liquid} 1s	538.13	Taken from reference [20]
4	[CF_3SO_3]Na	O_{liquid} 1s	538.13	Taken from reference [20]
5	[CF_3SO_3]$_2$Zn	O_{liquid} 1s	538.13	Taken from reference [20]
6	[CF_3SO_3]$_2$Mg	O_{liquid} 1s	538.13	Taken from reference [20]
7	[CF_3SO_3]$_2$Ca	O_{liquid} 1s	538.13	Taken from reference [20]
8	[CF_3SO_3]K	O_{liquid} 1s	538.13	Taken from reference [20]
9	[CF_3SO_3][C_4C_1Im]	O_{liquid} 1s	538.13	Taken from reference [20]
10	[BF_4][C_4C_1Im]	O_{liquid} 1s	538.13	Taken from reference [20]
11	[PF_6]Li	O_{liquid} 1s	538.13	Taken from reference [20]
12	[$(CF_3SO_2)_2N$]Li	O_{liquid} 1s	538.13	Taken from reference [20]
13	[$(CF_3SO_2)_2N$]Li	O_{liquid} 1s	538.13	Taken from reference [20]
14	[$(CF_3SO_2)_2N$]Li	O_{liquid} 1s	538.13	Taken from reference [20]
15	[$(FSO_2)_2N$]Na	O_{liquid} 1s	538.13	Taken from reference [20]

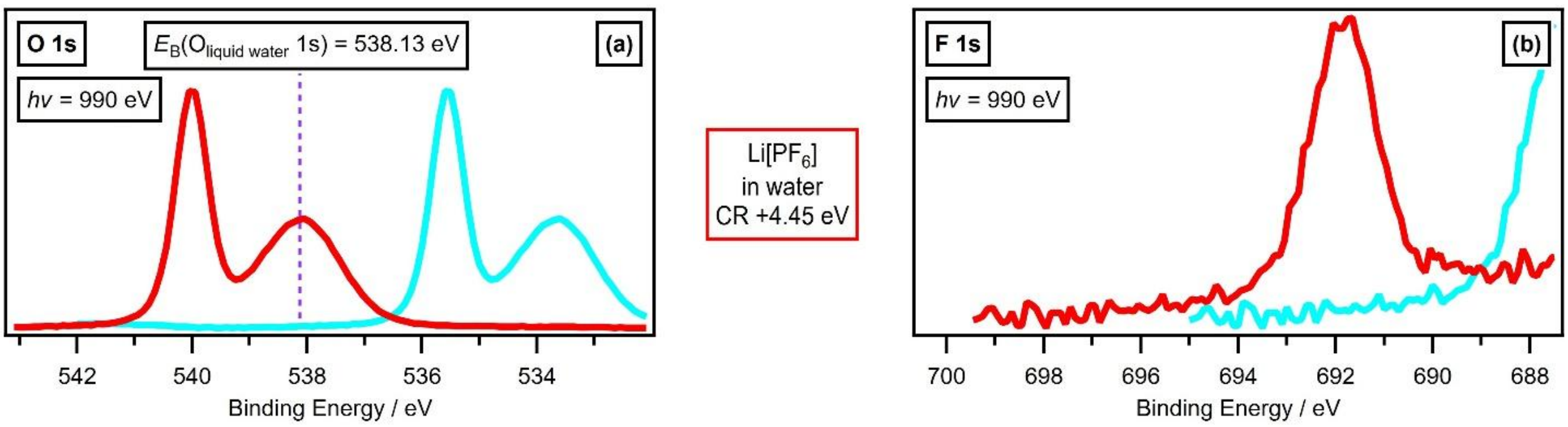

Figure S2. Example of charge referencing anions solvated in water-based samples using 0.50 M Li[PF_6] in water: (a) O 1s, with E_B($O_{liquid,water}$ 1s) = 538.13 eV, (b) F 1s, shifted by +4.45 eV from the raw data.

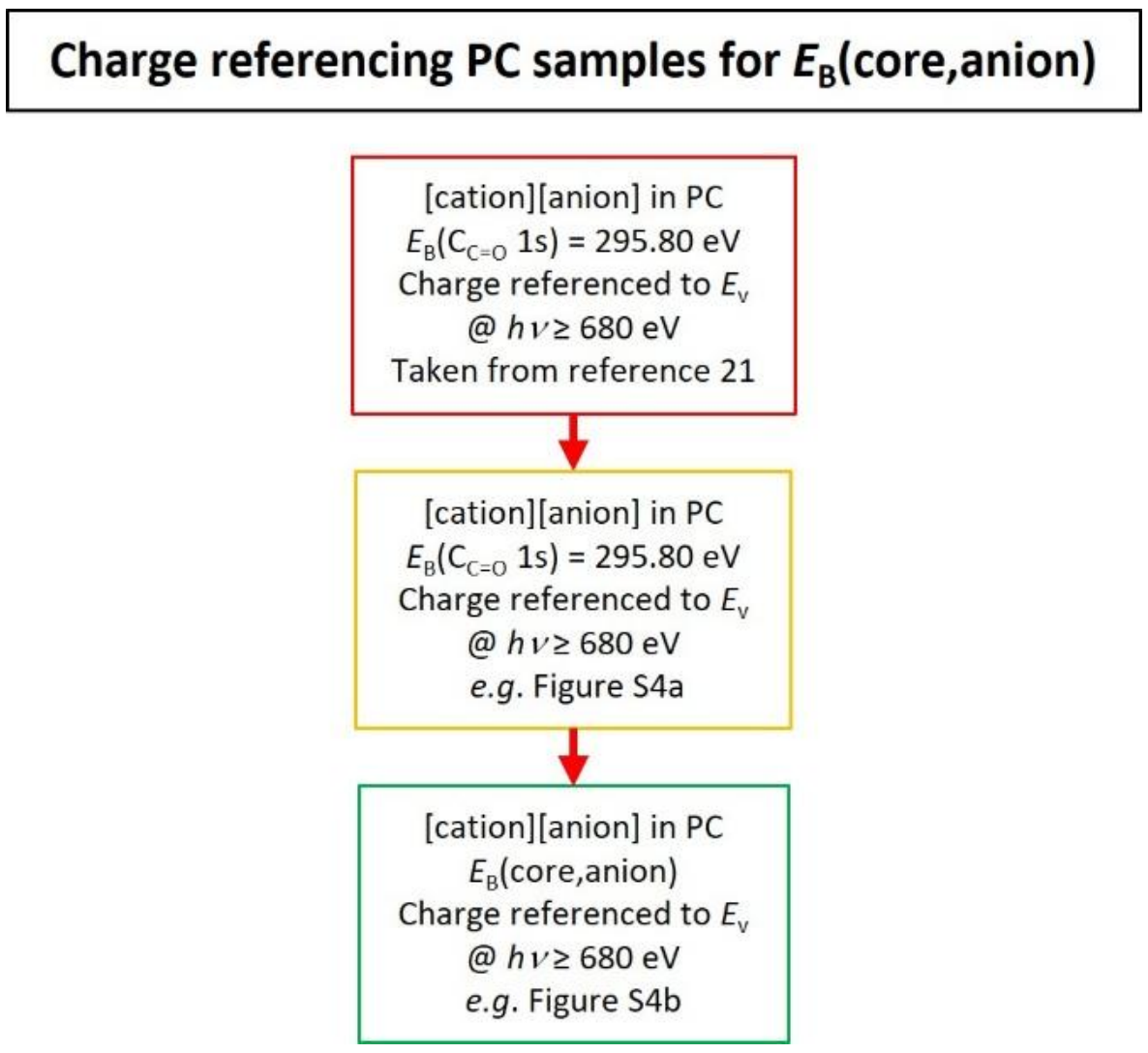

Figure S3. Flow-chart for charge referencing anions solvated in PC-based samples.

Table S18. Charge referencing core level X-ray photoelectron spectroscopy (XPS) for PC samples

Sample no.	Salt abbreviation	Core-level used for charge referencing	E_B(core) used to effectively charge reference to vacuum level / eV	Rationale for choosing core-level used for charge referencing
1	[SCN]K	$C_{C=O}$ 1s	295.80	Taken from reference [20]
2	$[SCN][C_4C_1Im]$	$C_{C=O}$ 1s	295.80	Taken from reference [20]
3	$[SCN][C_4C_1Im]$	$C_{C=O}$ 1s	295.80	Taken from reference [20]
4	$[CF_3SO_3]Na$	$C_{C=O}$ 1s	295.80	Taken from reference [20]
5	$[PF_6]Li$	$C_{C=O}$ 1s	295.80	Taken from reference [20]
6	$[(CF_3SO_2)_2N]Na$	$C_{C=O}$ 1s	295.80	Taken from reference [20]
7	$[(CF_3SO_2)_2N]Li$	$C_{C=O}$ 1s	295.80	Taken from reference [20]
8	$[(CF_3SO_2)_2N]Li$	$C_{C=O}$ 1s	295.80	Taken from reference [20]
9	$[(FSO_2)_2N]Na$	$C_{C=O}$ 1s	295.80	Taken from reference [20]

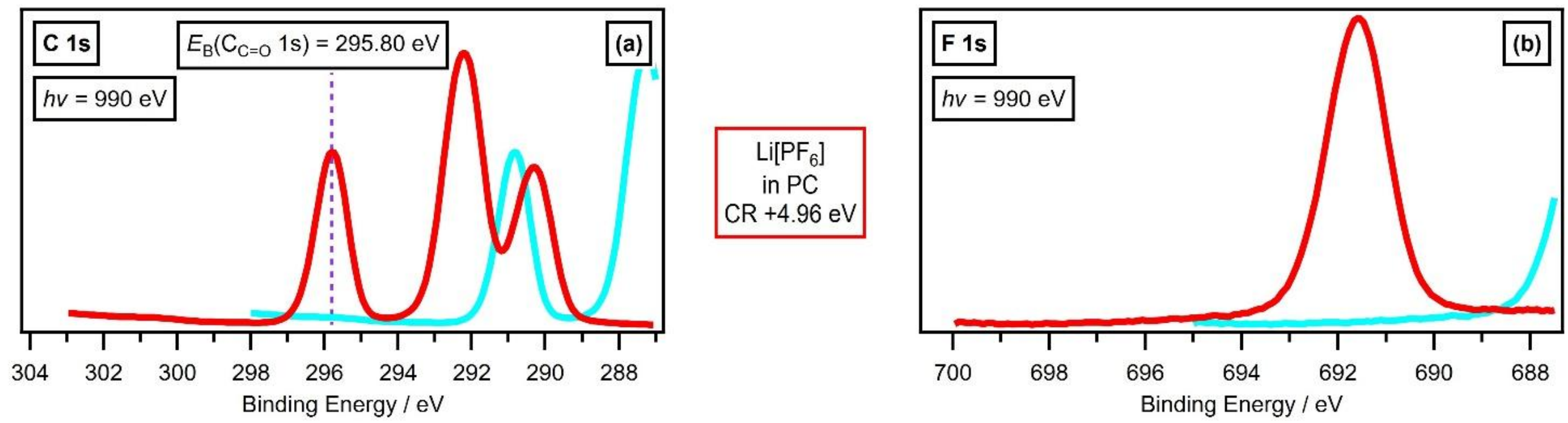

Figure S4. Example of charge referencing anions solvated in water-based samples using 0.50 M $Li[PF_6]$ in water: (a) C 1s, with $E_B(C_{C=O}$ 1s) = 295.80 eV, (b) F 1s, shifted by +4.96 eV from the raw data.

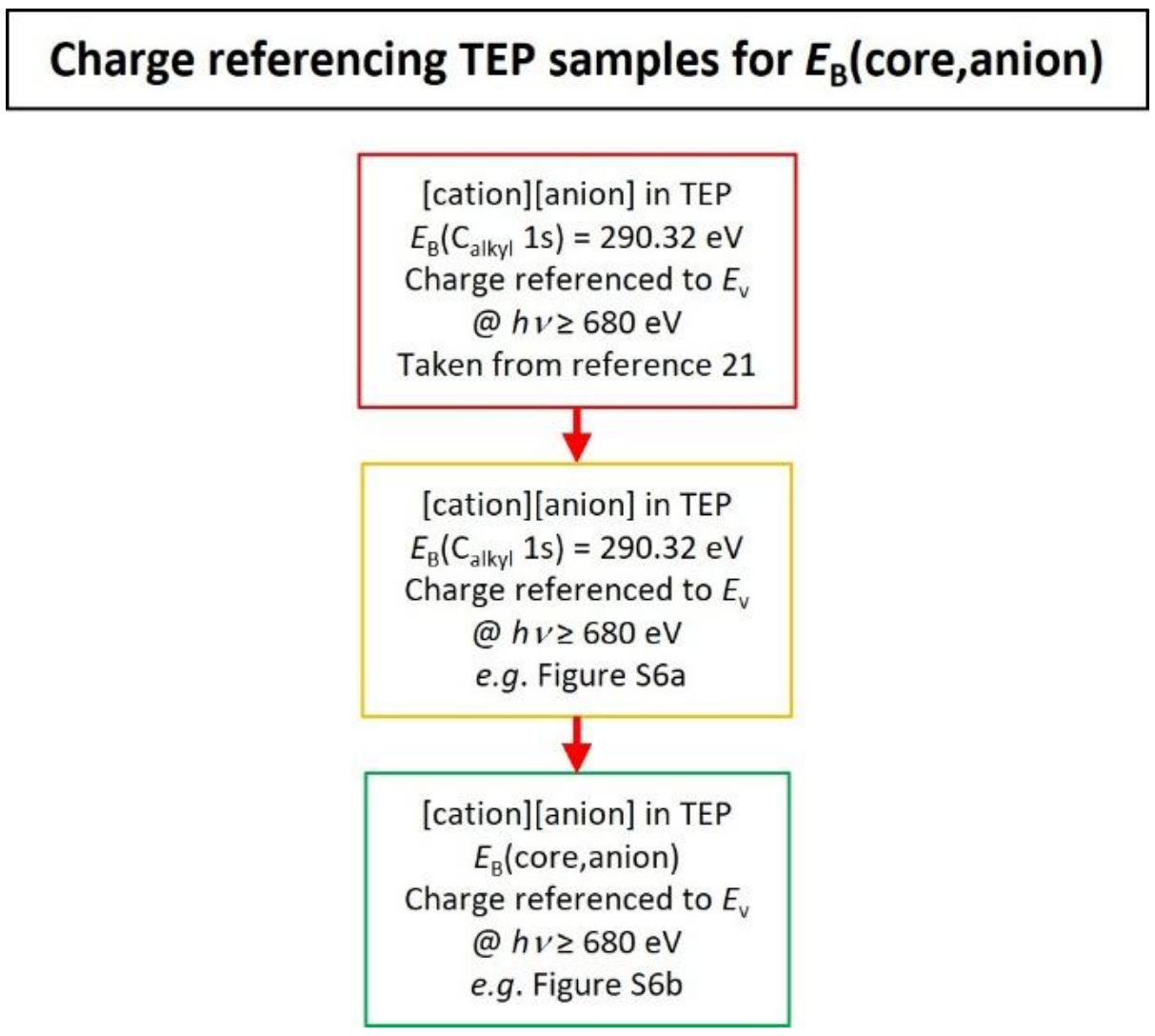

Figure S5. Flow-chart for charge referencing anions solvated in TEP-based samples.

Table S19. Charge referencing core level X-ray photoelectron spectroscopy (XPS) for TEP samples

Sample no.	Salt abbreviation	Core-level used for charge referencing	E_B(core) used to effectively charge reference to vacuum level / eV	Rationale for choosing core-level used for charge referencing
1	$[SCN][C_4C_1Im]$	C_{alkyl} 1s	290.32	Taken from reference [20]
2	$[PF_6]Li$	C_{alkyl} 1s	290.32	Taken from reference [20]
3	$[(CF_3SO_2)_2N]Na$	C_{alkyl} 1s	290.32	Taken from reference [20]
4	$[(CF_3SO_2)_2N]Li$	C_{alkyl} 1s	290.32	Taken from reference [20]
5	$[(FSO_2)_2N]Na$	C_{alkyl} 1s	290.32	Taken from reference [20]

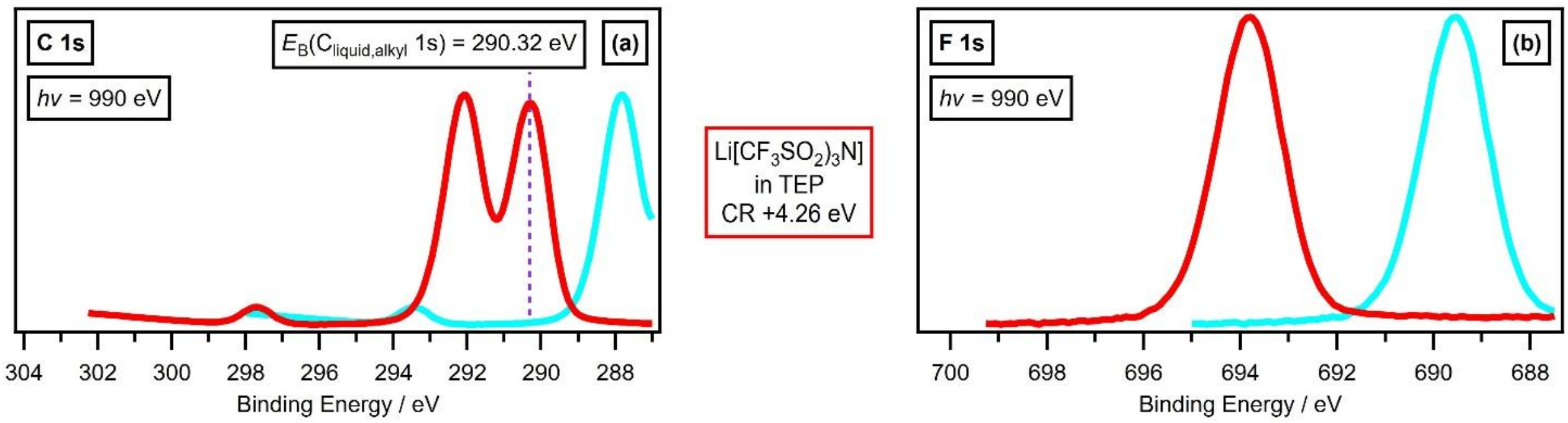

Figure S6. Example of charge referencing anions solvated in water-based samples using 0.48 M $Li[(CF_3SO_2)_2N]$ in TEP: (a) C 1s, with $E_B(C_{C=O}$ 1s) = 290.32 eV, (b) F 1s, shifted by +4.26 eV from the raw data.

8.2. Charge referencing ionic liquid XPS

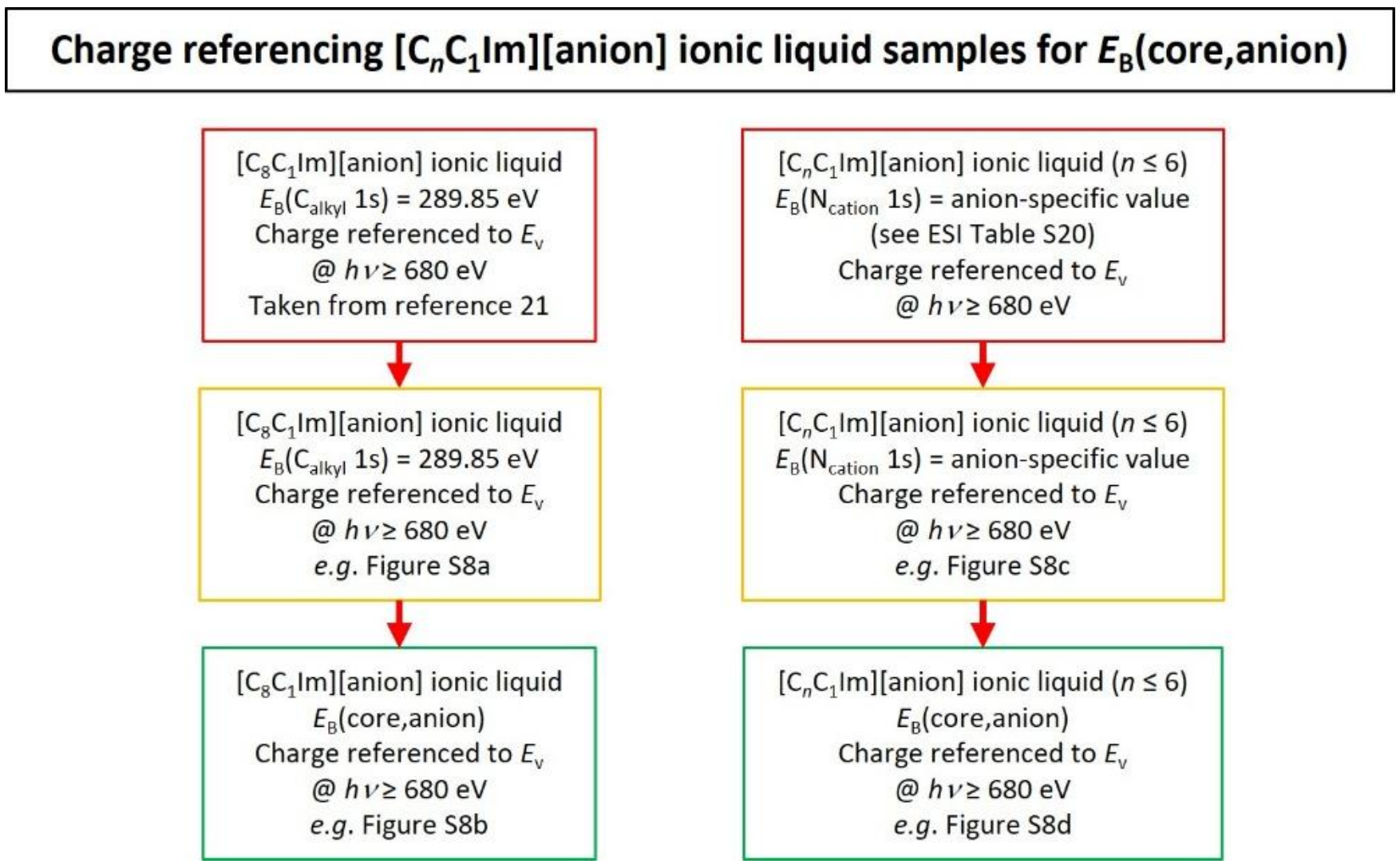

Figure S7. Flow-chart for charge referencing anions solvated in ionic liquid-based samples.

Table S20. Charge referencing core level X-ray photoelectron spectroscopy (XPS) for ionic liquid samples

Ionic liquid no.	Abbreviation	Core-level used for charge referencing	E_B(core) used to effectively charge reference to Fermi level / eV	E_B(core) used to effectively charge reference to vacuum level / eV	Rationale for choosing core-level used for charge referencing
1	$[C_8C_1Im][NO_3]$	C_{alkyl} 1s	285.00	289.85	Long alkyl chain
2	$[C_4C_1Im][CH_3CO_2]$	N_{cation} 1s	401.69	406.54	N_{cation} 1s for $[C_8C_1Im][CH_3CO_2]$ [21]
3	$[C_2C_1Im][CH_3SO_3]$	N_{cation} 1s	401.90	406.75	Average N_{cation} 1s for $[C_8C_1Im][A]$ [22]
4	$[C_8C_1Im][HOSO_3]$	C_{alkyl} 1s	285.00	289.85	Long alkyl chain
5	$[C_4C_1Im][CH_3OSO_3]$	N_{cation} 1s	401.71	406.56	N_{cation} 1s for $[C_4C_1Im][C_8H_{17}OSO_3]$
6	$[C_4C_1Im][C_8H_{17}OSO_3]$	C_{alkyl} 1s	285.00	289.85	Long alkyl chain
7	$[C_4C_1Im][(CH_3O)_2PO_2]$	N_{cation} 1s	401.90	406.75	Average N_{cation} 1s for $[C_8C_1Im][A]$ [22]
8	$[C_8C_1Im][CF_3CO_2]$	C_{alkyl} 1s	285.00	289.85	Long alkyl chain
9	$[C_8C_1Im][CF_3SO_3]$	C_{alkyl} 1s	285.00	289.85	Long alkyl chain
10	$[C_8C_1Im][(CF_3SO_2)_2N]$	C_{alkyl} 1s	285.00	289.85	Long alkyl chain
11	$[C_8C_1Im][(FSO_2)_2N]$	C_{alkyl} 1s	285.00	289.85	Long alkyl chain
12	$[C_4C_1Im][(C_2F_5SO_2)_2N]$	C_{CF2} 1s	291.07	295.92	C_{CF2} 1s for $[C_8C_1Im][(C_2F_5SO_2)_2N]$ [8]
13	$[C_8C_1Im][C(CN)_3]$	C_{alkyl} 1s	285.00	289.85	Long alkyl chain
14	$[C_6C_1Im][B(CN)_4]$	C_{alkyl} 1s	285.00	289.85	Long alkyl chain
15	$[C_4C_1Im][N(CN)_2]$	N_{cation} 1s	402.00	406.85	N_{cation} 1s for $[C_8C_1Im][N(CN)_2]$ [22]
16	$[C_8C_1Im][SCN]$	C_{alkyl} 1s	285.00	289.85	Long alkyl chain
17	$[C_8C_1Im][BF_4]$	C_{alkyl} 1s	285.00	289.85	Long alkyl chain
18	$[C_8C_1Im][PF_6]$	C_{alkyl} 1s	285.00	289.85	Long alkyl chain
19	$[C_8C_1Im][SbF_6]$	C_{alkyl} 1s	285.00	289.85	Long alkyl chain
20	$[C_2C_1Im][(C_2F_5)_3PF_3]$	N_{cation} 1s	402.27	407.12	N_{cation} 1s for $[C_8C_1Im][(C_2F_5)_3PF_3]$ [11]
21	$[C_8C_1Im]Cl$	C_{alkyl} 1s	285.00	289.85	Long alkyl chain
22	$[C_8C_1Im]_2[ZnCl_4]$	C_{alkyl} 1s	285.00	289.85	Long alkyl chain
23	$[C_8C_1Im]_2[FeCl_4]$	C_{alkyl} 1s	285.00	289.85	Long alkyl chain
24	$[C_8C_1Im]_2[CoCl_4]$	C_{alkyl} 1s	285.00	289.85	Long alkyl chain
25	$[C_8C_1Im]_2[NiCl_4]$	C_{alkyl} 1s	285.00	289.85	Long alkyl chain
26	$[C_8C_1Im][FeCl_4]$	C_{alkyl} 1s	285.00	289.85	Long alkyl chain
27	$[C_8C_1Im][InCl_4]$	C_{alkyl} 1s	285.00	289.85	Long alkyl chain
28	$[C_8C_1Im][SnCl_3]$	C_{alkyl} 1s	285.00	289.85	Long alkyl chain
29	$[C_8C_1Im]_{11}[LiCl_3]Cl[(CF_3SO_2)_2N]_8$	C_{alkyl} 1s	285.00	289.85	Long alkyl chain
30	$[C_8C_1Im]_2[Bi_2Cl_8]$	C_{alkyl} 1s	285.00	289.85	Long alkyl chain
31	$[C_8C_1Im]_2[Zn_2Cl_6]$	C_{alkyl} 1s	285.00	289.85	Long alkyl chain
32	$[C_8C_1Im]_2[Zn_3Cl_8]$	C_{alkyl} 1s	285.00	289.85	Long alkyl chain
33	$[C_8C_1Im]_2[Zn_4Cl_{10}]$	C_{alkyl} 1s	285.00	289.85	Long alkyl chain
34	$[C_8C_1Im]Br$	C_{alkyl} 1s	285.00	289.85	Long alkyl chain
35	$[C_8C_1Im]_2[ZnBr_4]$	C_{alkyl} 1s	285.00	289.85	Long alkyl chain
36	$[C_8C_1Im]_2[CoBr_4]$	C_{alkyl} 1s	285.00	289.85	Long alkyl chain
37	$[C_8C_1Im][InBr_4]$	C_{alkyl} 1s	285.00	289.85	Long alkyl chain
38	$[C_8C_1Im][SnBr_3]$	C_{alkyl} 1s	285.00	289.85	Long alkyl chain
39	$[C_8C_1Im]_2[Zn_2Br_6]$	C_{alkyl} 1s	285.00	289.85	Long alkyl chain
40	$[C_8C_1Im]_2[Zn_3Br_8]$	C_{alkyl} 1s	285.00	289.85	Long alkyl chain
41	$[C_8C_1Im]_2[Zn_4Br_{10}]$	C_{alkyl} 1s	285.00	289.85	Long alkyl chain
42	$[C_6C_1Im]I$	N_{cation} 1s	401.86	406.71	N_{cation} 1s for $[C_8C_1Im]I$ [8]
43	$[C_8C_1Im][I_3]$	C_{alkyl} 1s	285.00	289.85	Long alkyl chain
44	$[C_4C_1Im]_2[Co(NCS)_4]$	N_{cation} 1s	402.00	406.85	0.1 eV larger than $E_B(N_{cation}$ 1s) for $[C_8C_1Im][SCN]$

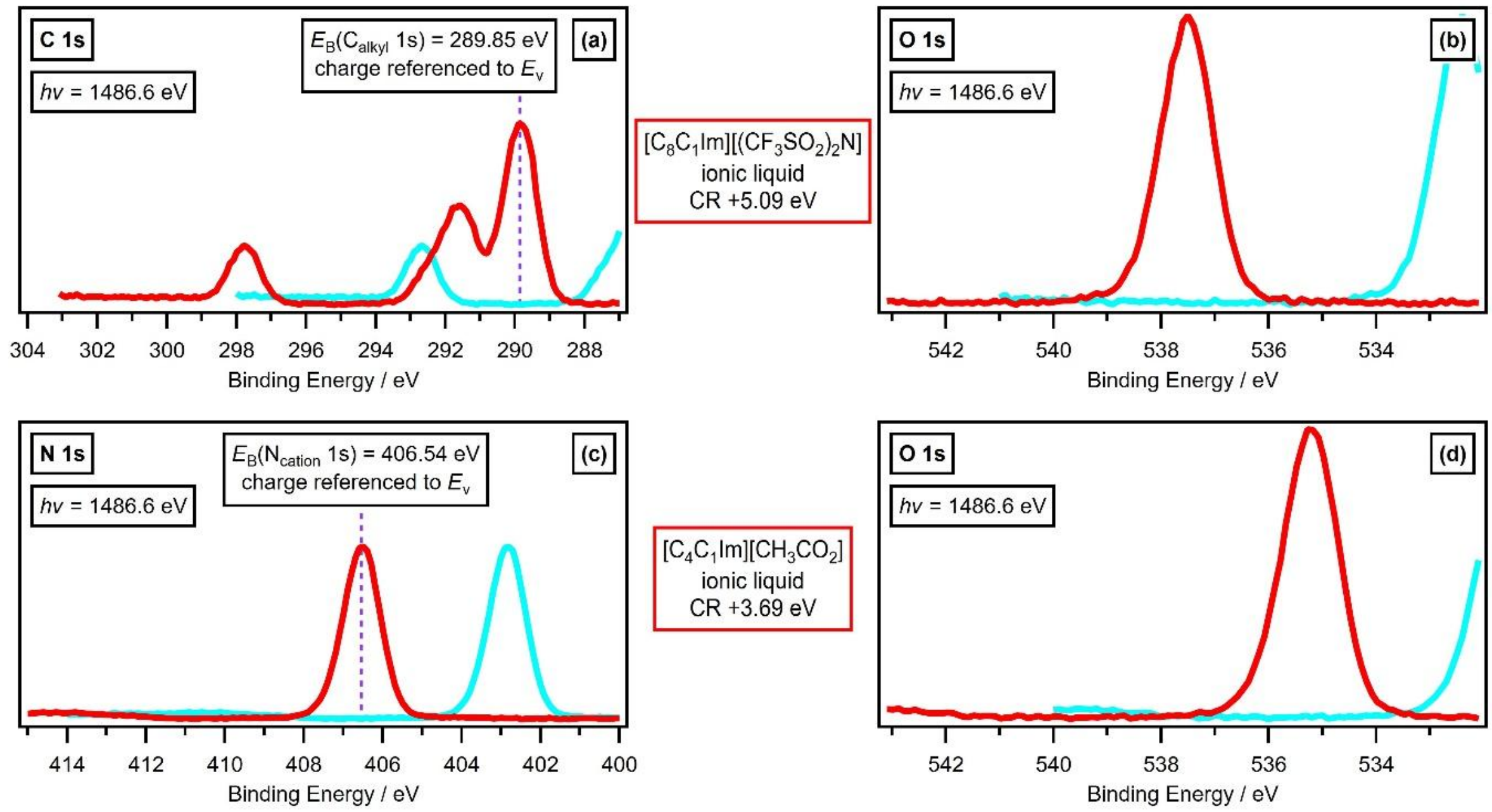

Figure S8. Example of charge referencing anions solvated in ionic liquid-based samples. (a) and (b) Using E_B(C_{alkyl} 1s). [C_8C_1Im][$(CF_3SO_2)_2N$] ionic liquid, shifted by +5.09 eV from the raw data: (a) C 1s, with E_B(C_{alkyl} 1s) = 289.85 eV (see Table S20 for rationalization of this choice), (b) O 1s. (c) and (d) Using E_B(N_{cation} 1s). [C_4C_1Im][(CH_3CO_2] ionic liquid, shifted by +3.69 eV from the raw data: (c) N 1s, with E_B(N_{cation} 1s) = 406.54 eV (see Table S20 for rationalization of this choice), (d) O 1s.

8.3. Area normalization

Quantitative area normalization is achieved by dividing by the area of the fitted component and then multiplying by the nominal number of atoms in the anion.

9. Results. XPS: demonstrating purity using XPS for new ionic liquid samples

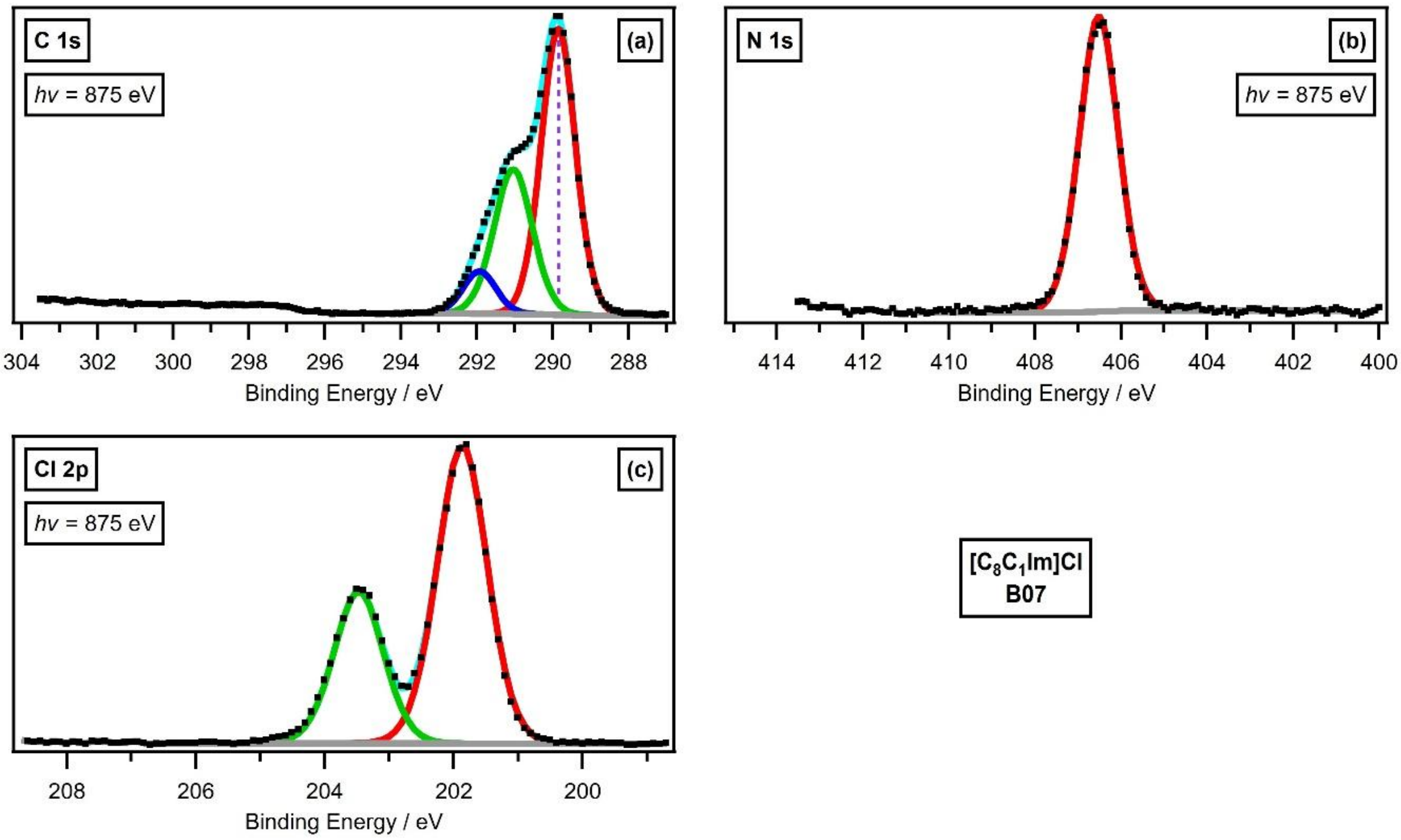

Figure S9. Core XP spectra for $[C_8C_1Im]Cl$ recorded on synchrotron–based XPS apparatus (B07-B-XPS) at $h\nu$ = 875 eV. All XP spectra were charge referenced using the method outlined in ESI Section 8.2.

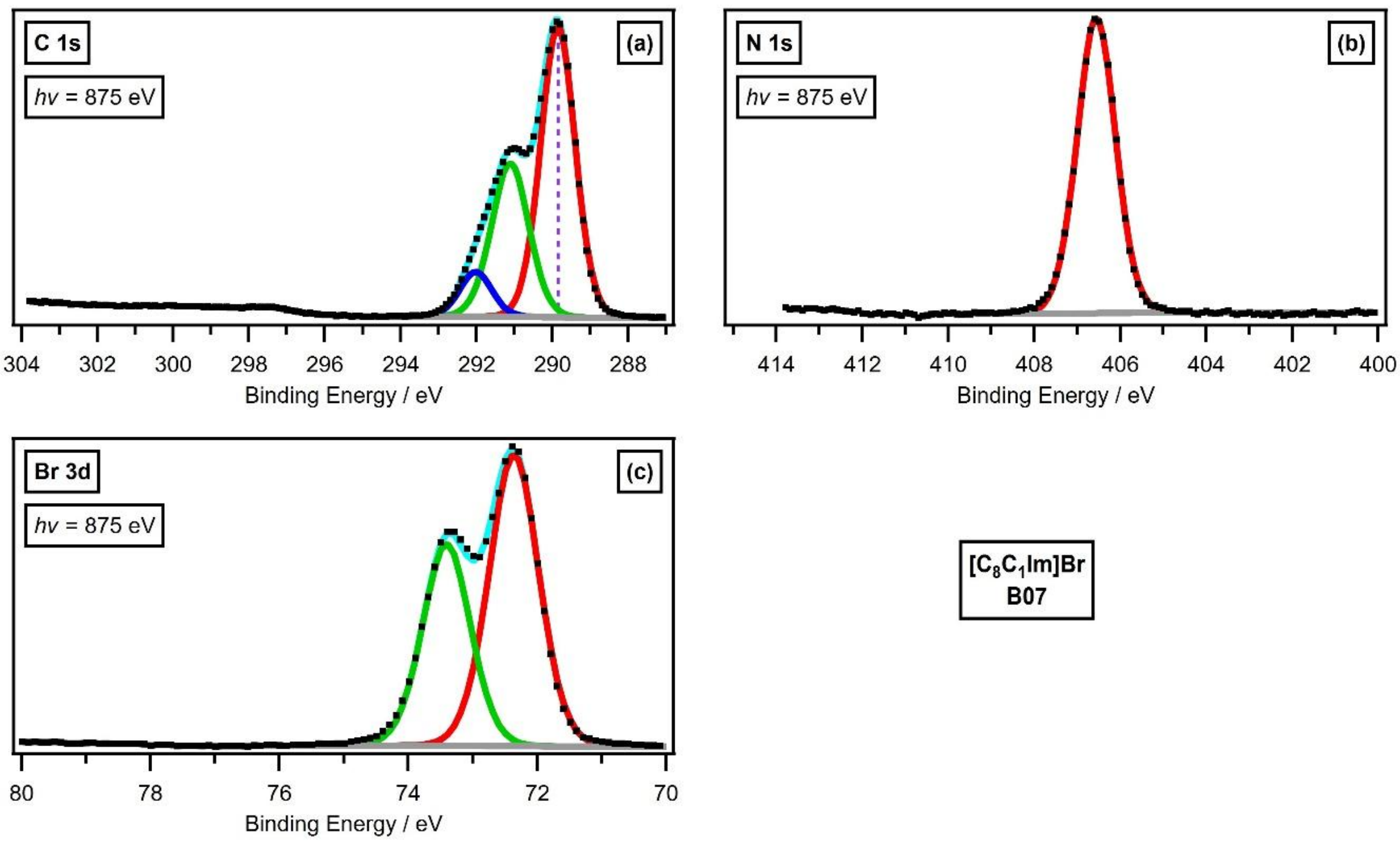

Figure S10. Core XP spectra for $[C_8C_1Im]Br$ recorded on synchrotron–based XPS apparatus (B07-B-XPS) at $h\nu$ = 875 eV. All XP spectra were charge referenced using the method outlined in ESI Section 8.2.

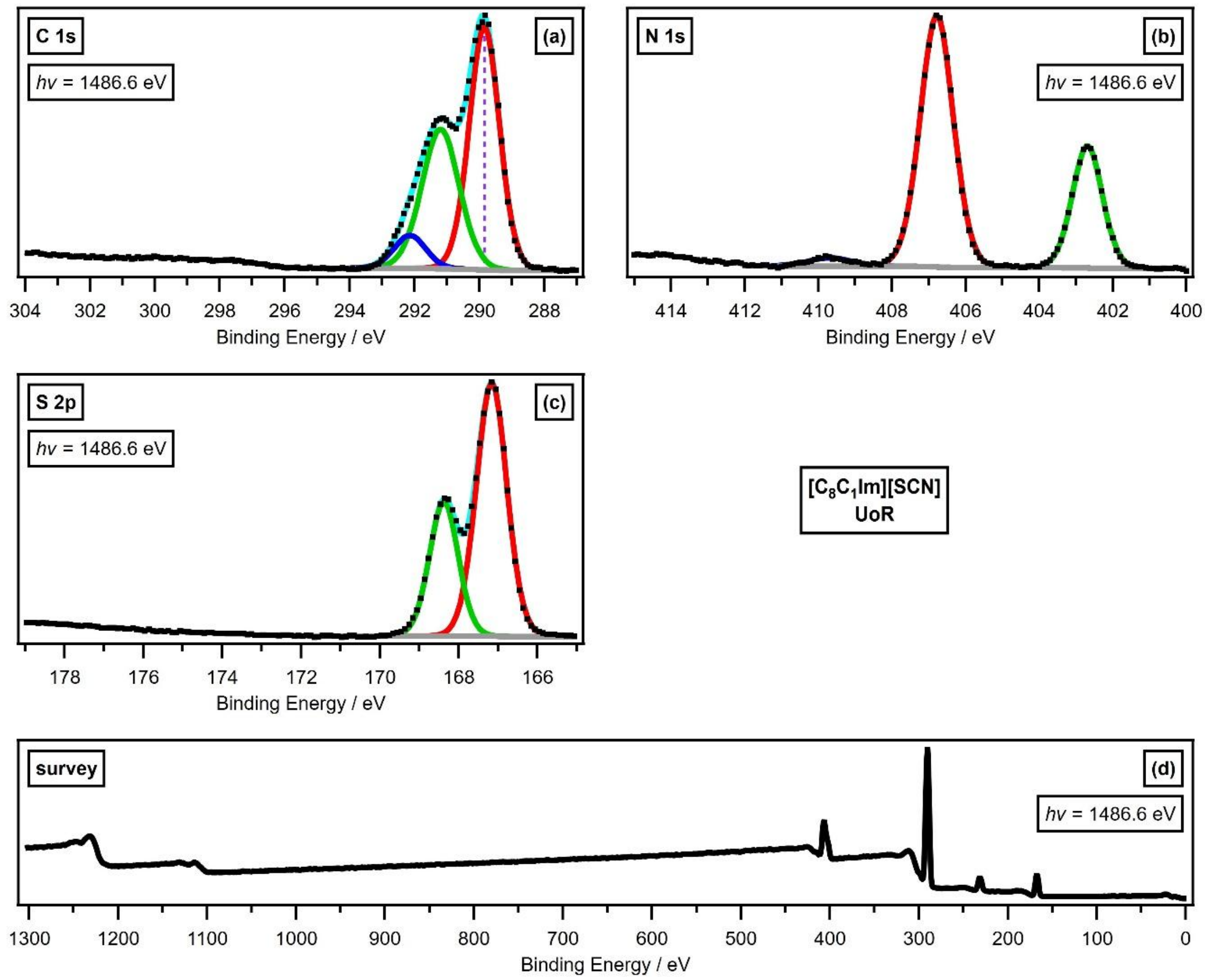

Figure S11. (a-c) Core and (d) survey XP spectra for [C_8C_1Im][SCN] recorded on laboratory–based XPS apparatus at $h\nu$ = 1486.6 eV. All XP spectra were charge referenced using the method outlined in ESI Section 8.2.

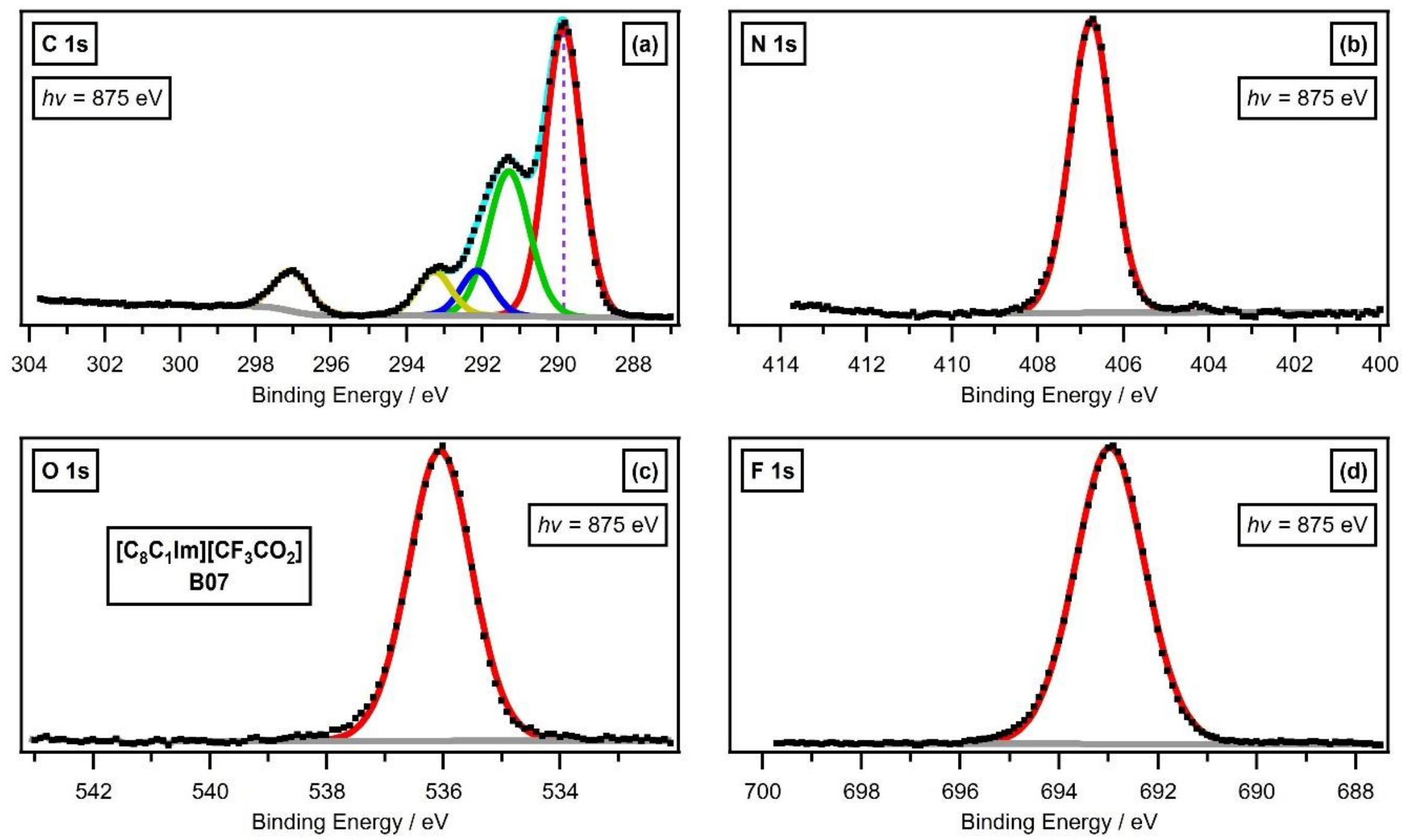

Figure S12. Core XP spectra for [C_8C_1Im][CF_3CO_2] recorded on synchrotron–based XPS apparatus (B07-B-XPS) at $h\nu$ = 875 eV. All XP spectra were charge referenced using the method outlined in ESI Section 8.2.

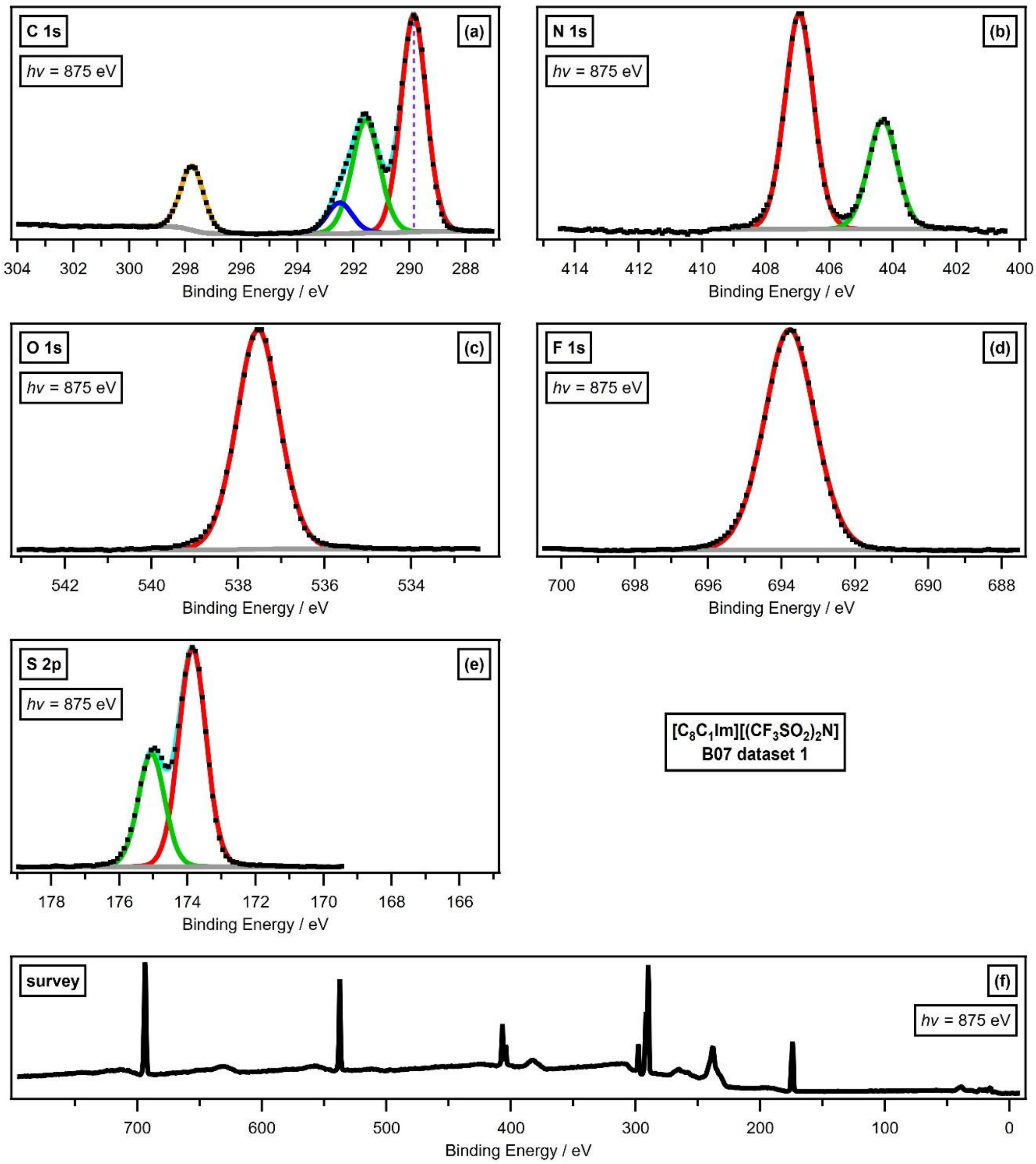

Figure S13. (a-e) Core and (f) survey XP spectra for $[C_8C_1Im][(CF_3SO_2)_2N]$ recorded on synchrotron–based XPS apparatus (B07-B-XPS) at $h\nu$ = 875 eV. All XP spectra were charge referenced using the method outlined in ESI Section 8.2.

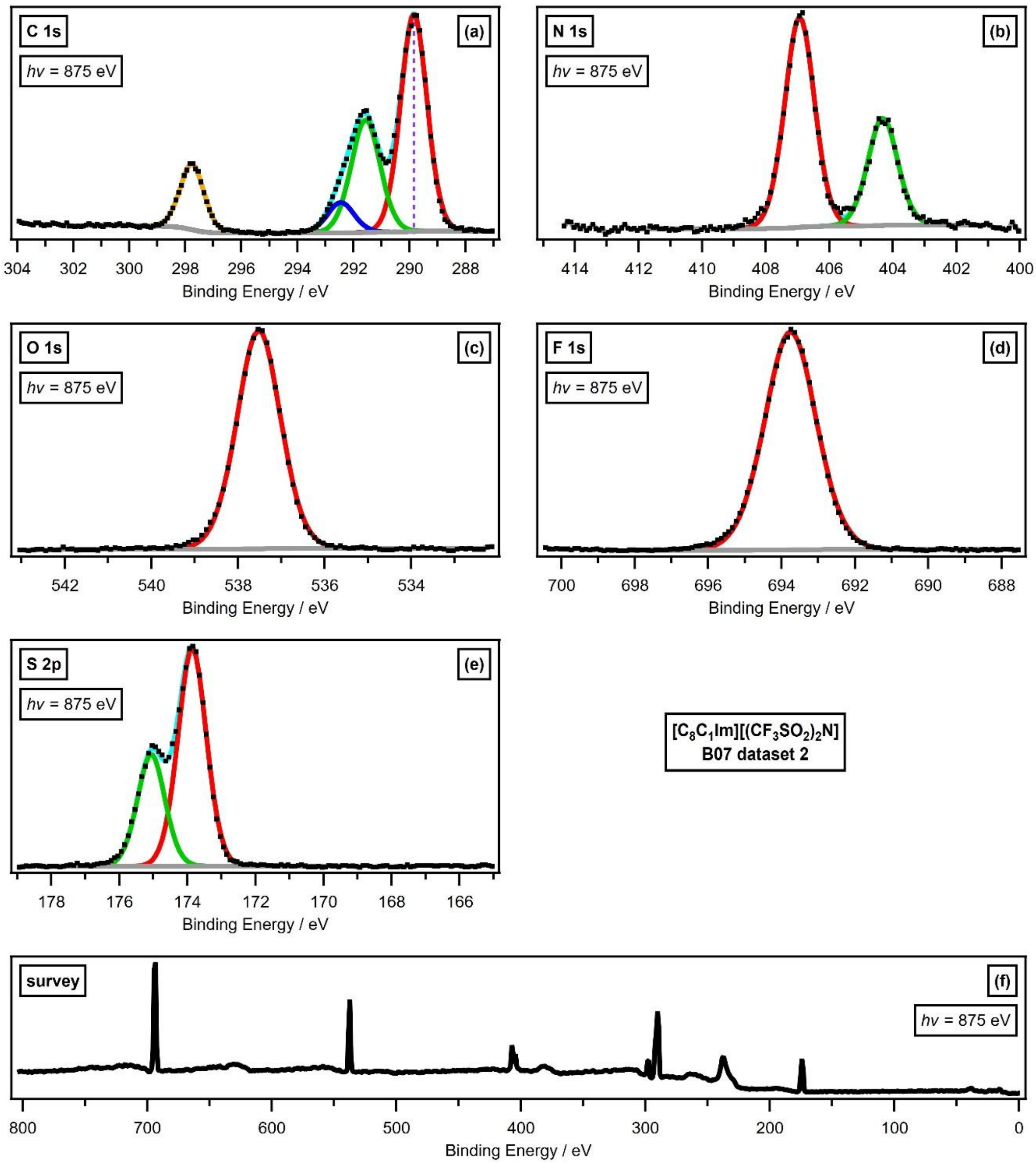

Figure S14. (a-e) Core and (f) survey XP spectra for $[C_8C_1Im][(CF_3SO_2)_2N]$ recorded on synchrotron–based XPS apparatus (B07-B-XPS) at $h\nu$ = 875 eV. All XP spectra were charge referenced using the method outlined in ESI Section 8.2.

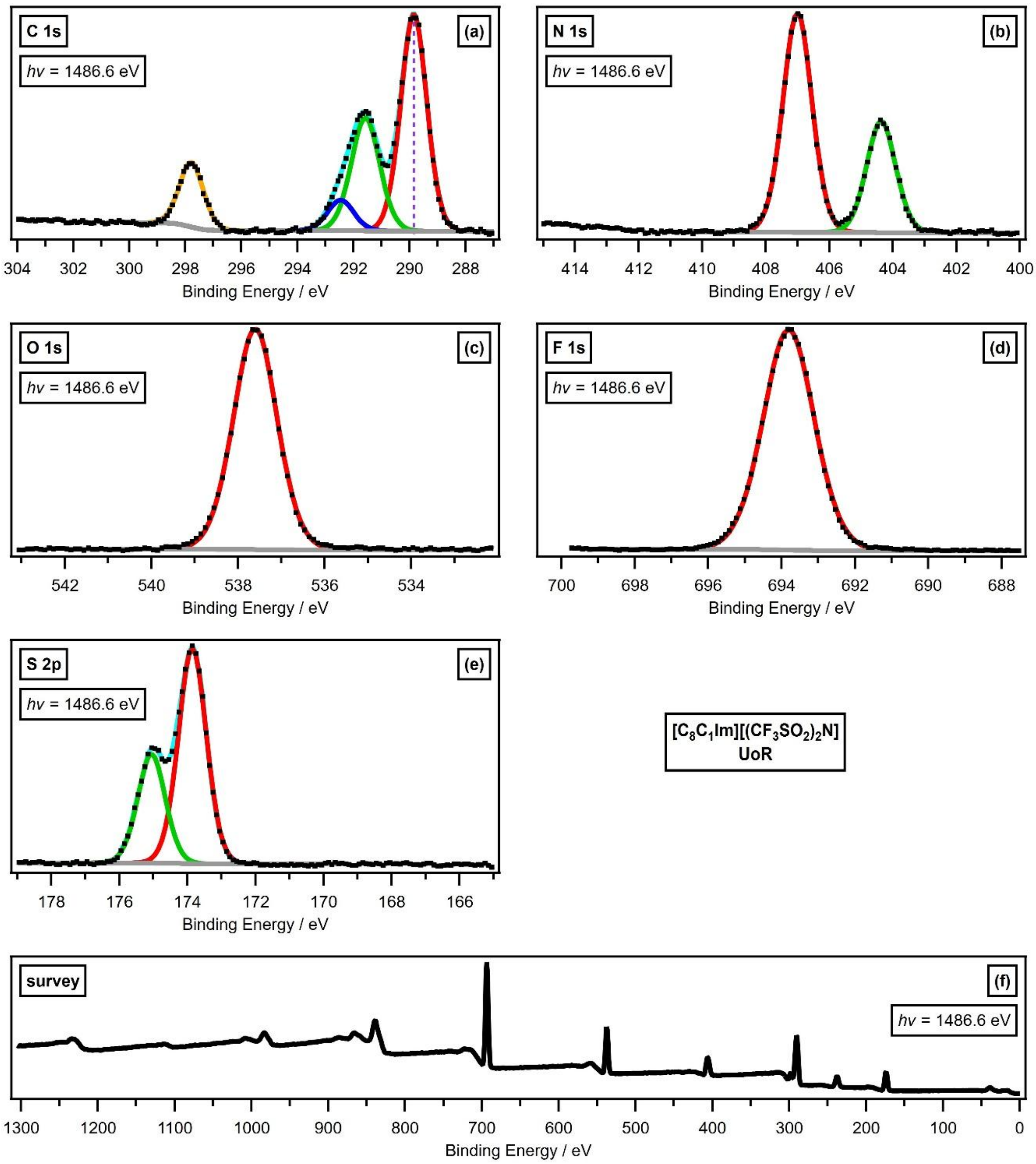

Figure S15. (a-e) Core and (f) survey XP spectra for $[C_8C_1Im][(CF_3SO_2)_2N]$ recorded on laboratory–based XPS apparatus at $h\nu$ = 1486.6 eV. All XP spectra were charge referenced using the method outlined in ESI Section 8.2.

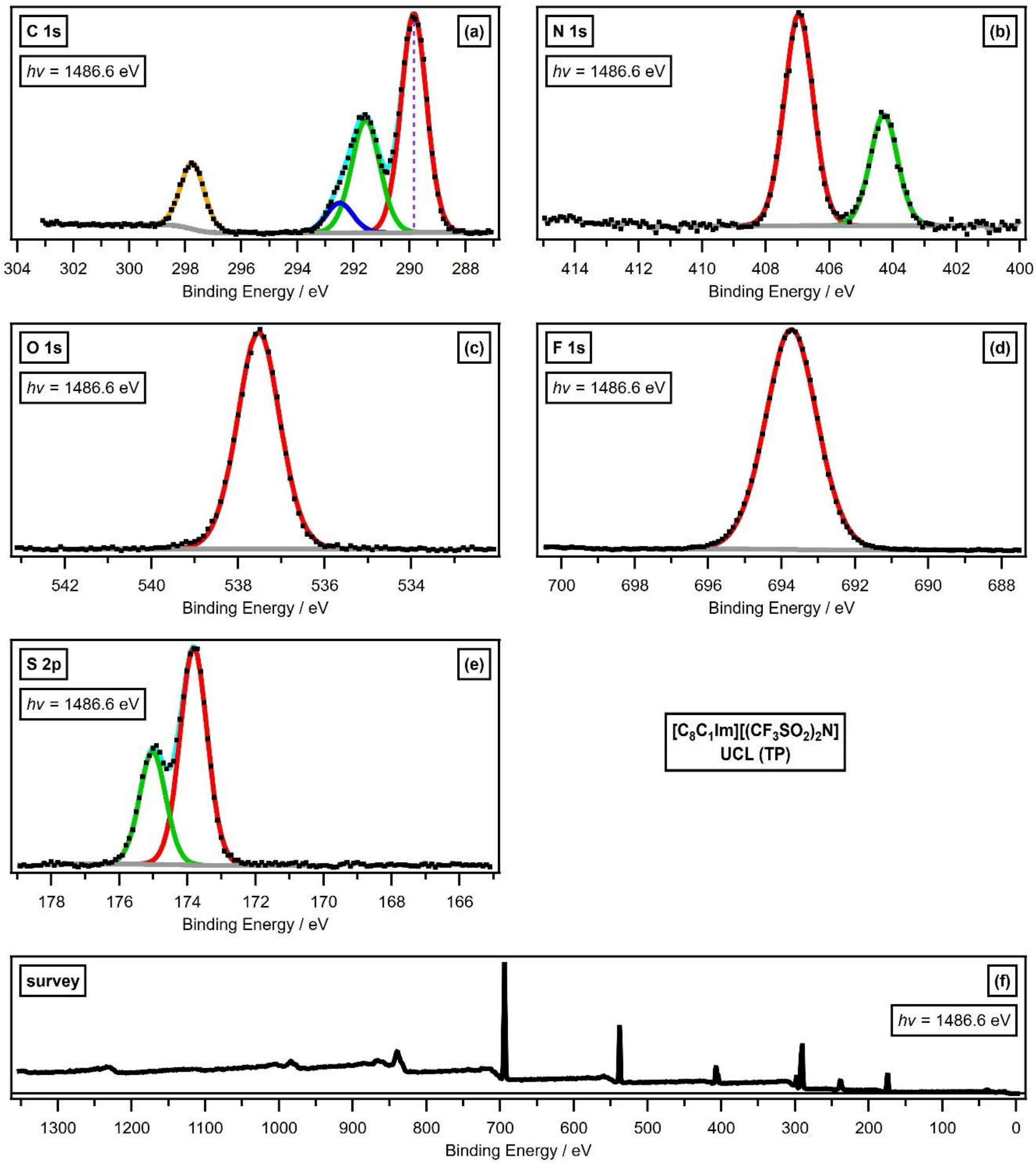

Figure S16. (a-e) Core and (f) survey XP spectra for $[C_8C_1Im][(CF_3SO_2)_2N]$ recorded on laboratory–based XPS apparatus at $h\nu$ = 1486.6 eV. All XP spectra were charge referenced using the method outlined in ESI Section 8.2.

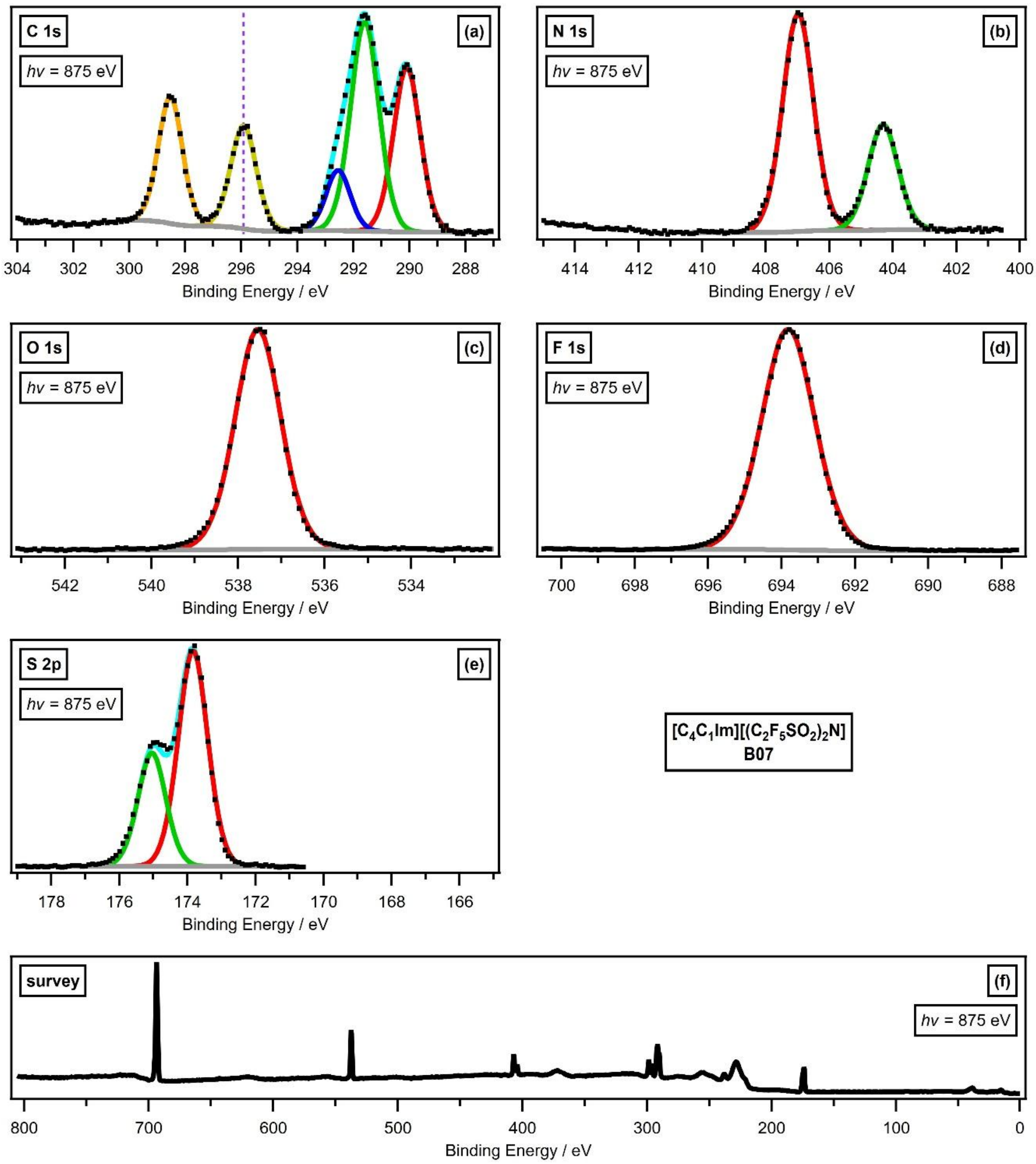

Figure S17. Core XP spectra for $[C_4C_1Im][(C_2F_5SO_2)_2N]$ recorded on synchrotron–based XPS apparatus (B07-B-XPS) at $h\nu$ = 875 eV. All XP spectra were charge referenced using the method outlined in ESI Section 8.2.

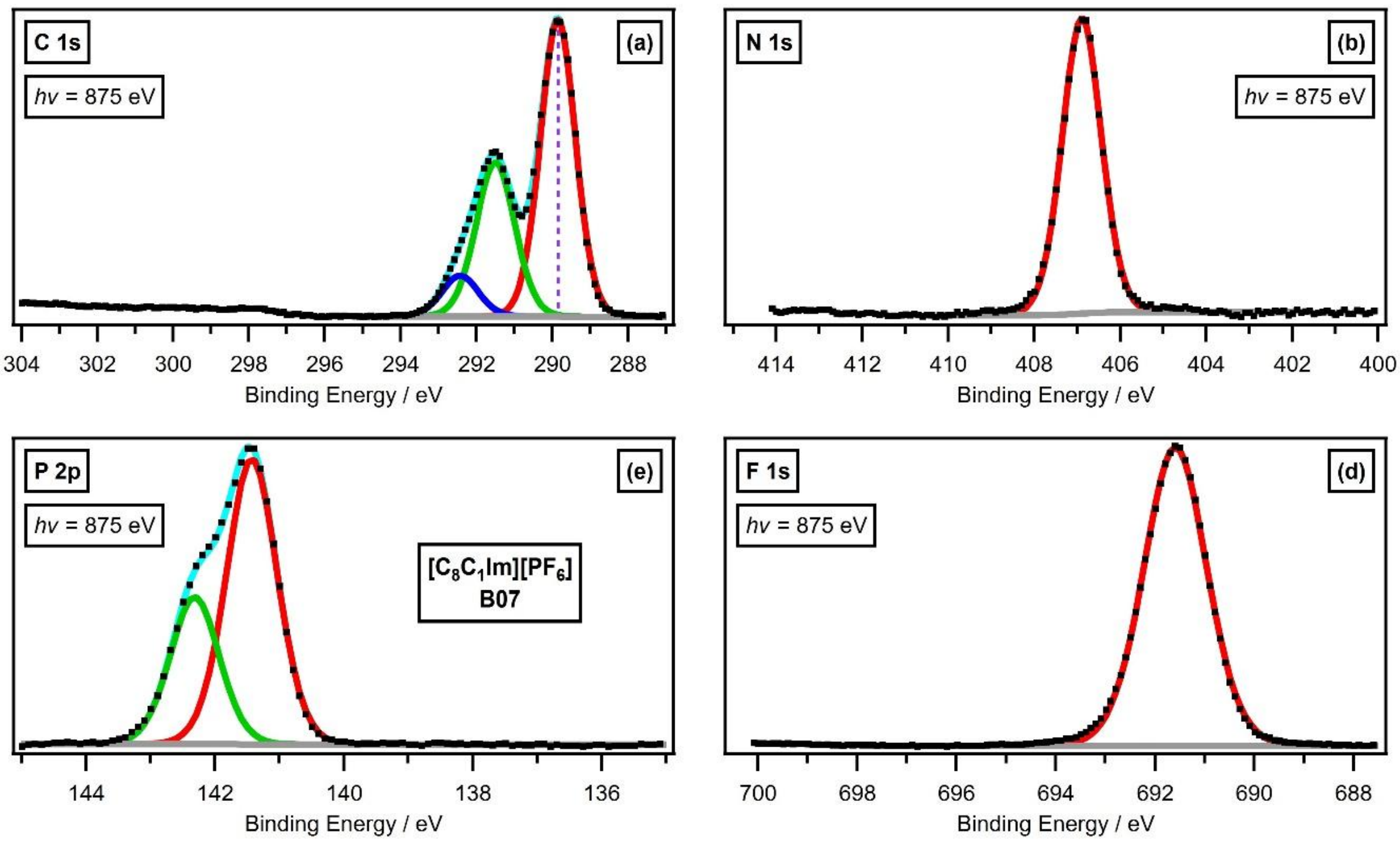

Figure S18. Core XP spectra for $[C_8C_1Im][(PF_6]$ recorded on synchrotron–based XPS apparatus (B07-B-XPS) at $h\nu = 875$ eV. All XP spectra were charge referenced using the method outlined in ESI Section 8.2.

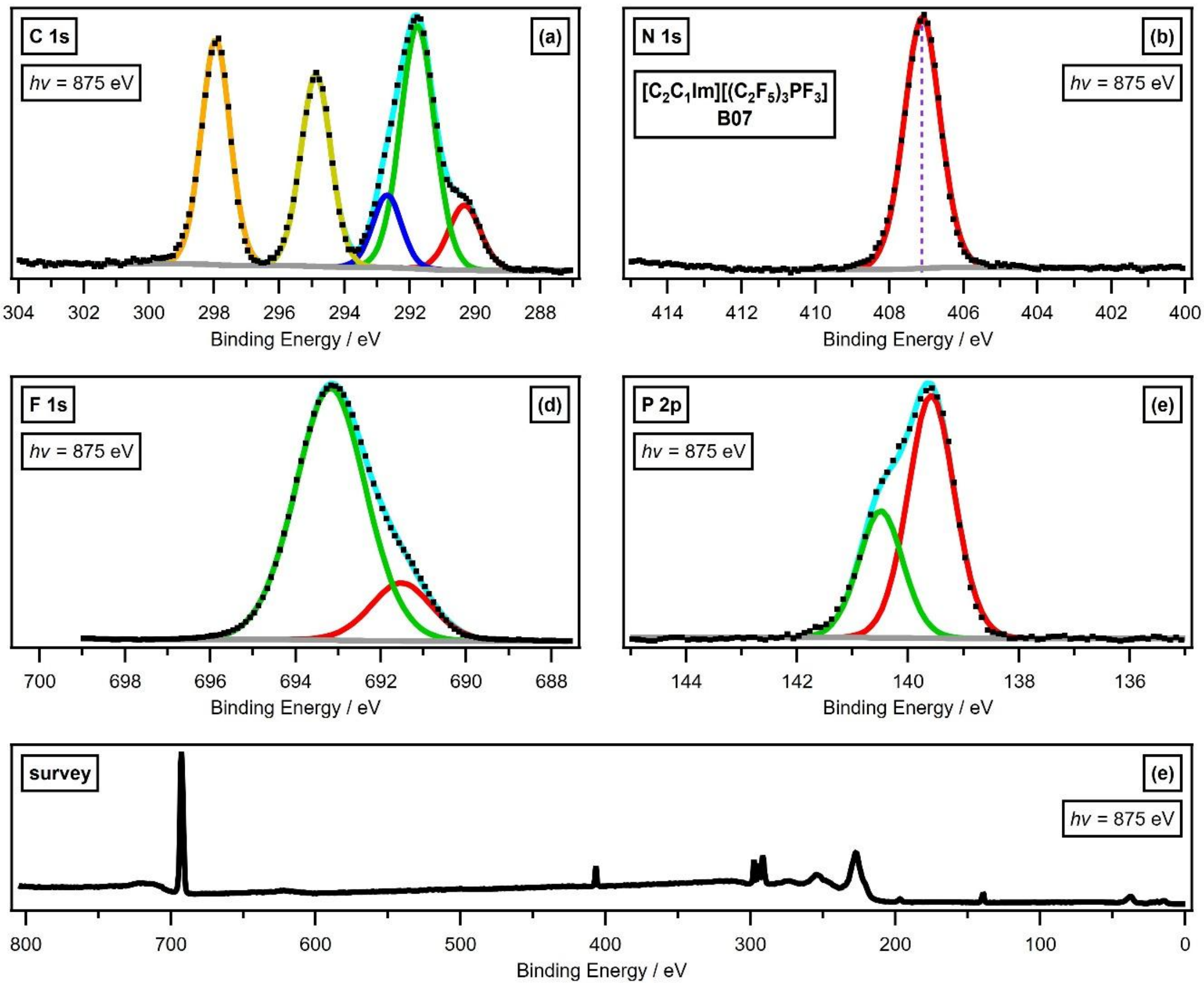

Figure S19. (a-d) Core and (e) survey XP spectra for $[C_2C_1Im][(C_2F_5)_3PF_3]$ recorded on synchrotron–based XPS apparatus (B07-B-XPS) at $h\nu = 875$ eV. All XP spectra were charge referenced using the method outlined in ESI Section 8.2.

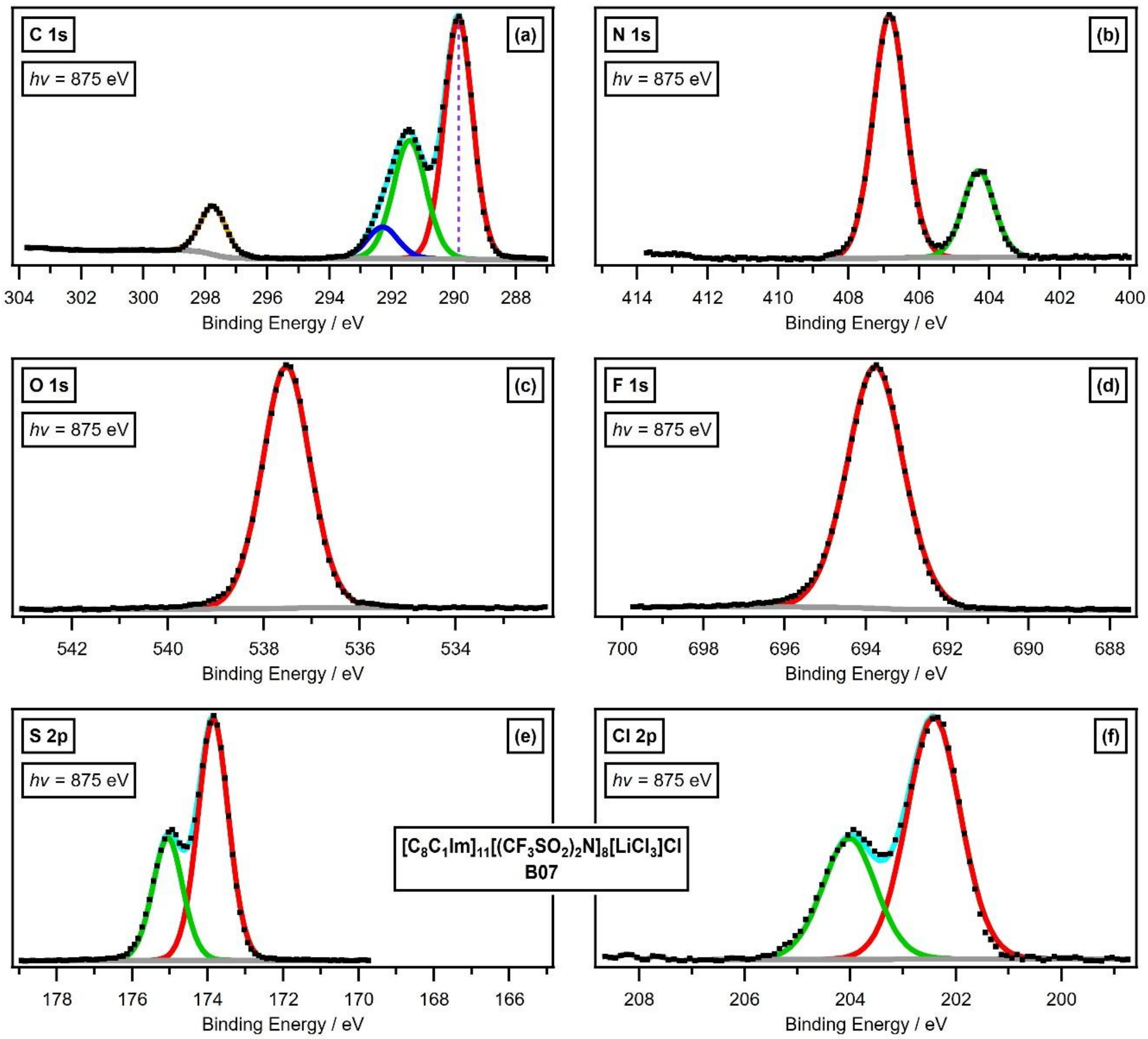

Figure S20. Core XP spectra for $[C_8C_1Im]_{11}[(CF_3SO_2)_2N]_8[LiCl_3]Cl$ recorded on synchrotron–based XPS apparatus (B07-B-XPS) at $h\nu$ = 875 eV. All XP spectra were charge referenced using the method outlined in ESI Section 8.2.

The measured experimental and nominal stoichiometries (Table S21) match well for all three ionic liquids newly investigated here using lab XPS. The relatively small differences are likely due to differences in relative sensitivity factors (RSF) values; the RSF values were not tuned specially to the XPS apparatus used here (in references [23, 24] RSF values were tuned specially to the XPS apparatus used in those studies). All these matches, along with the high-quality XP spectra given in ESI Figure S9 to ESI Figure S13, demonstrates the high purity of the ionic liquid samples newly presented here. The ionic liquid XPS measurements were made under ultrahigh vacuum (UHV) conditions. These UHV conditions mean that residual molecular solvents will have vaporized prior to XPS measurements, giving ultrapure samples from a molecular solvent contamination perspective. Furthermore, the element-specific nature of XPS means that a number of ionic impurities, *e.g.* Na^+, can be observed if present. Therefore, we can high very high confidence in the purity of our ionic liquids for the XPS measurements.

Table S21. Measured experimental and nominal (in brackets) stoichiometries for the new ionic liquids studied on lab XPS apparatus in this work, recorded at $h\nu$ = 1486.6 eV. RSF = relative sensitivity factors,[24] for C 1s, N 1s, O 1s, F 1s, S $2p_{3/2}$

		F 1s	O 1s	N 1s	C 1s	S $2p_{3/2}$
Abbreviation	RSF	1.000	0.580	0.350	0.205	0.267
$[C_8C_1Im][SCN]$ (UoR)	Measured (nominal)	N/A	N/A	2.7 (3)	13.3 (13)	1.0 (1)
$[C_8C_1Im][(CF_3SO_2)_2N]$ (UoR)	Measured (nominal)	5.3 (6)	3.8 (4)	3.0 (3)	14.6 (14)	2.2 (2)
$[C_8C_1Im][(CF_3SO_2)_2N]$ (UCL TP)	Measured (nominal)	Pass energy = 50 eV	3.7 (4)	2.8 (3)	14.5 (14)	2.1 (2)

10. Results. XPS: demonstrating purity using XPS for new salt in water samples

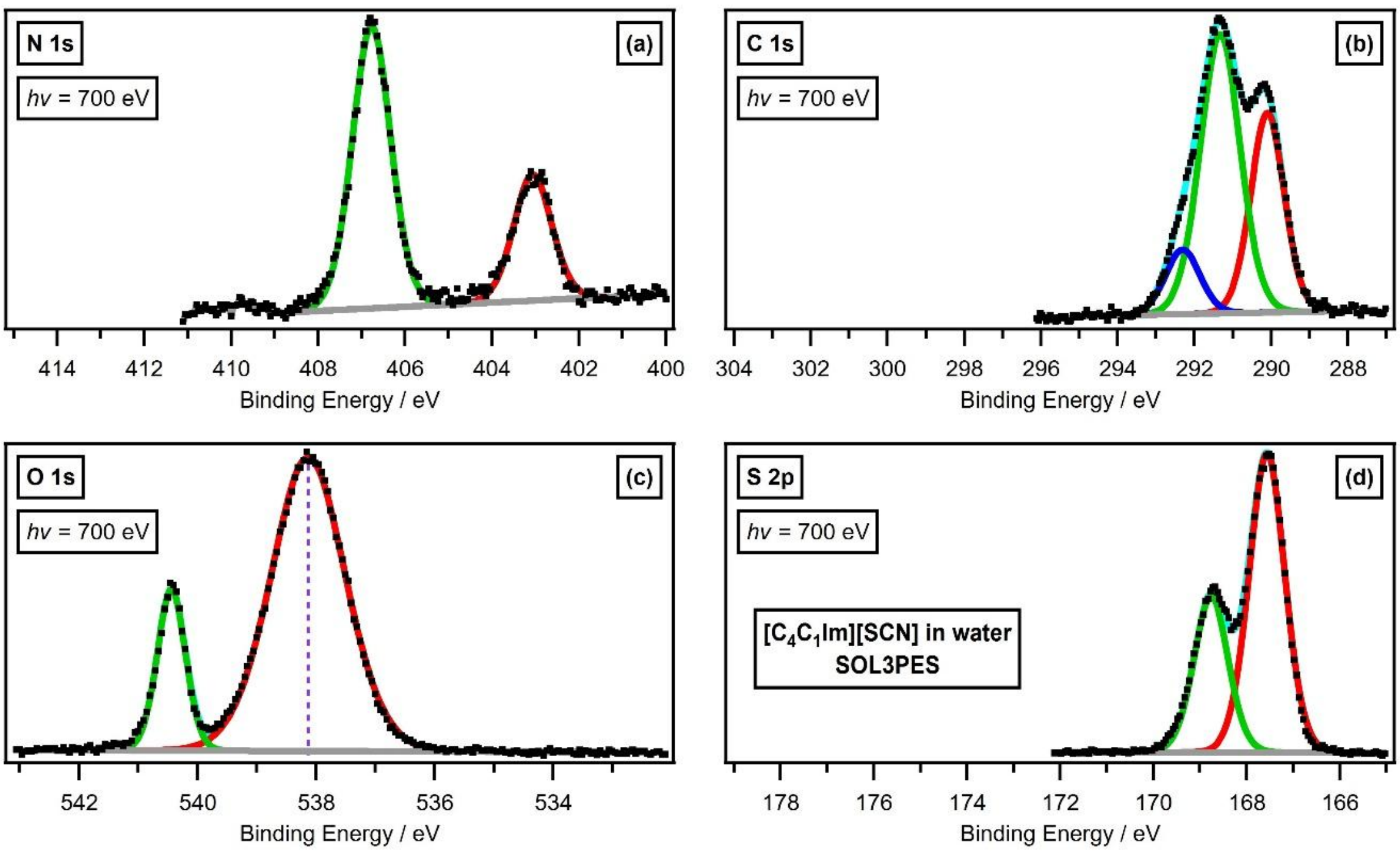

Figure S21. Core XP spectra for 0.50 M $[C_4C_1Im][SCN]$ in water recorded on synchrotron–based XPS apparatus (SOL3PES) at $h\nu$ = 700 eV. All XP spectra were charge referenced using the method outlined in ESI Section 8.1.

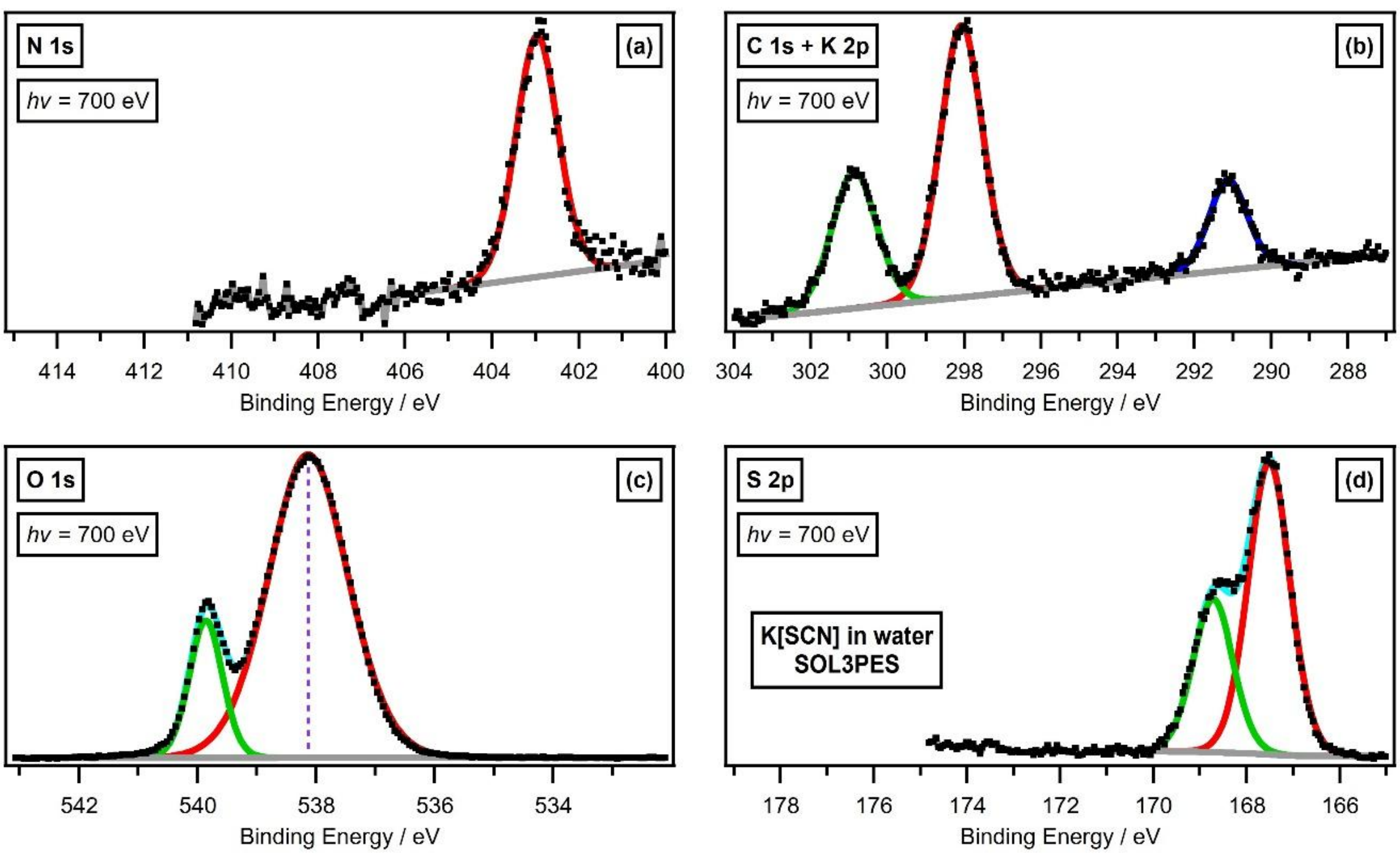

Figure S22. Core XP spectra for 0.50 M K[SCN] in water recorded on synchrotron–based XPS apparatus (SOL3PES) at $h\nu$ = 700 eV. All XP spectra were charge referenced using the method outlined in ESI Section 8.1.

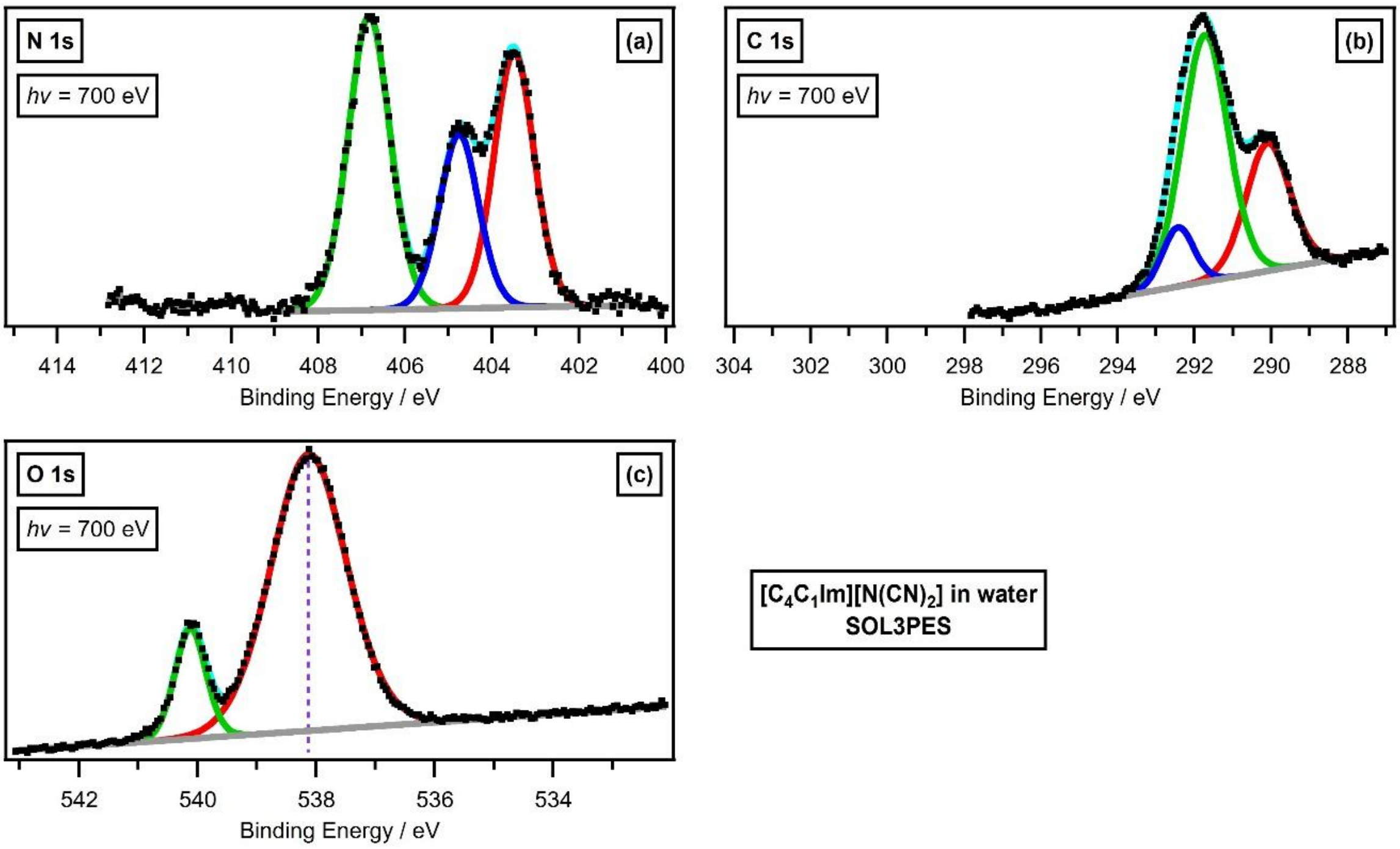

Figure S23. Core XP spectra for 0.50 M $[C_4C_1Im][N(CN)_2]$ in water recorded on synchrotron–based XPS apparatus (SOL3PES) at $h\nu$ = 700 eV. All XP spectra were charge referenced using the method outlined in ESI Section 8.1.

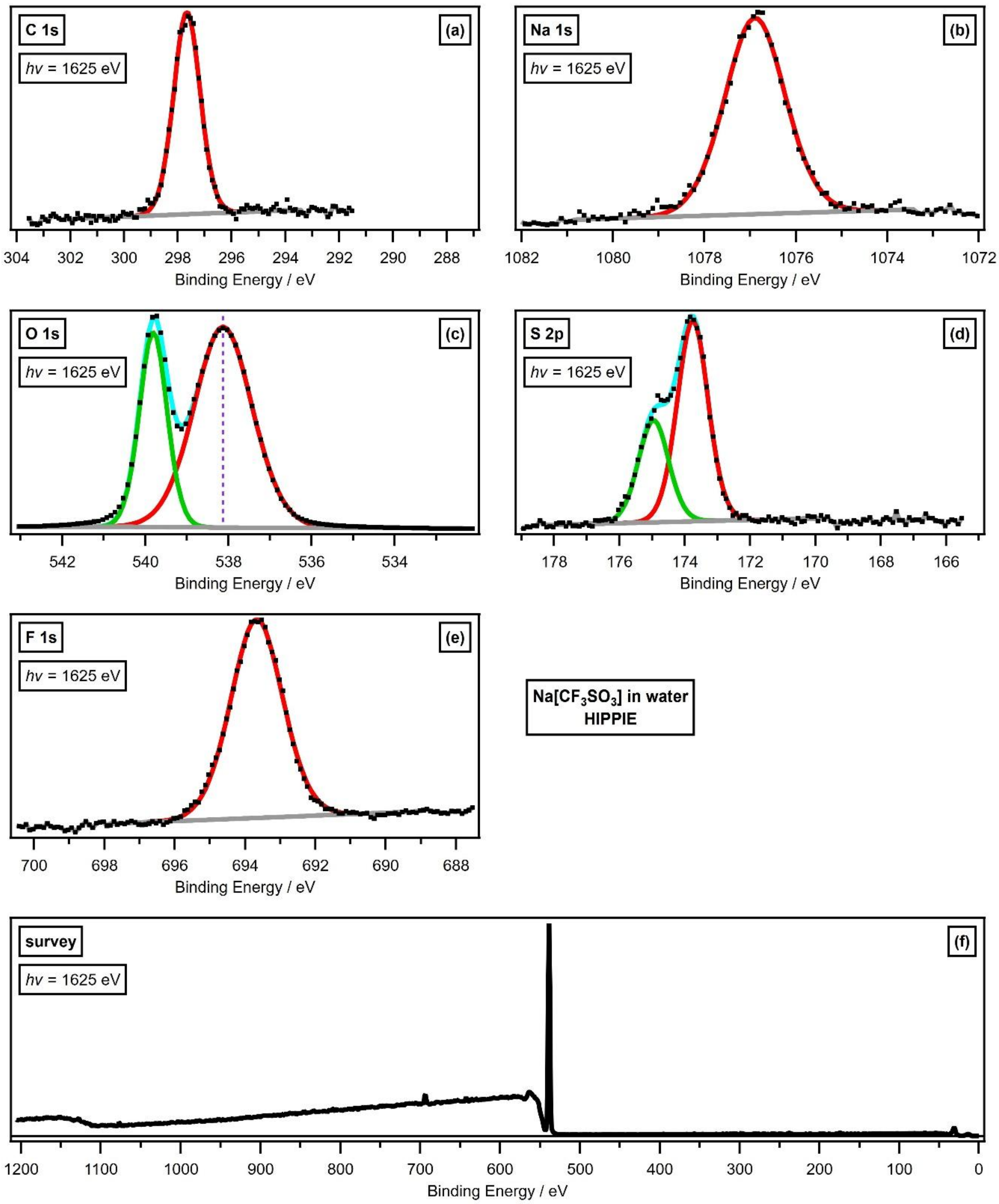

Figure S24. Core XP spectra for 0.40 M Na[CF_3SO_3] in water recorded on synchrotron–based XPS apparatus (HIPPIE) at *hν* = 1625 eV. All XP spectra were charge referenced using the method outlined in ESI Section 8.1.

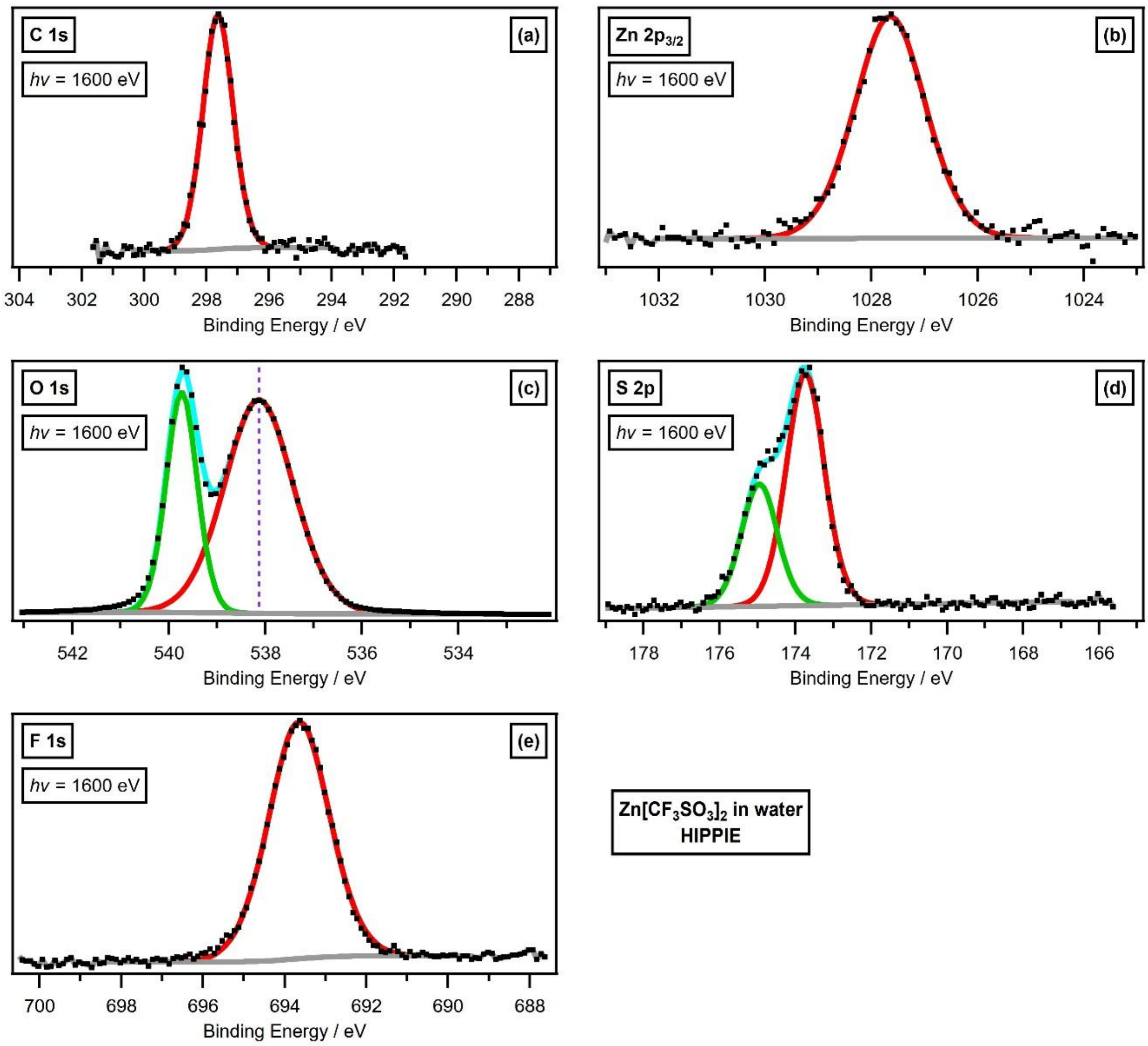

Figure S25. Core XP spectra for 0.40 M $Zn[CF_3SO_3]_2$ in water recorded on synchrotron–based XPS apparatus (HIPPIE) at $h\nu$ = 1600 eV. All XP spectra were charge referenced using the method outlined in ESI Section 8.1.

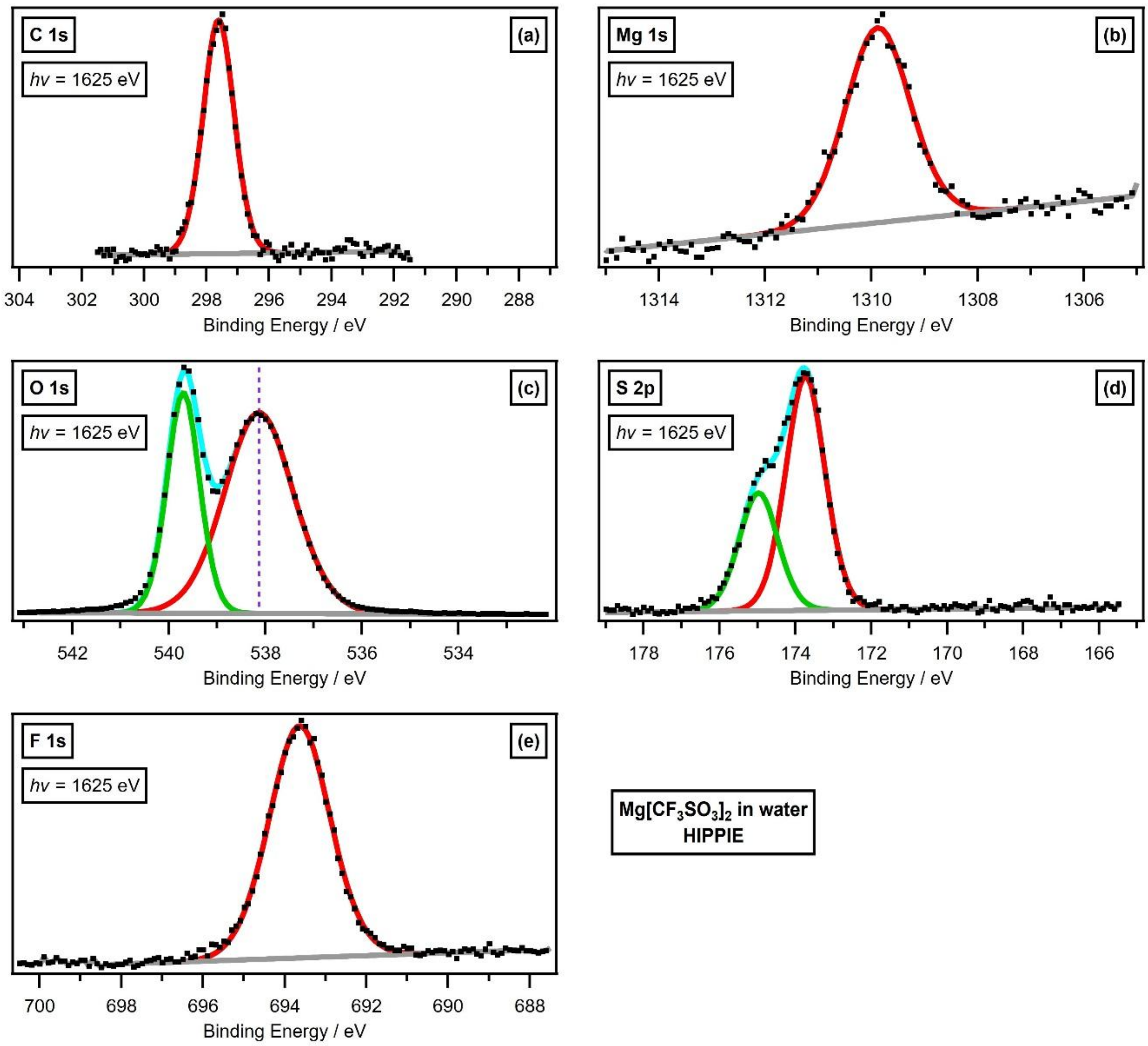

Figure S26. Core XP spectra for 0.40 M $Mg[CF_3SO_3]_2$ in water recorded on synchrotron–based XPS apparatus (HIPPIE) at $h\nu$ = 1625 eV. All XP spectra were charge referenced using the method outlined in ESI Section 8.1.

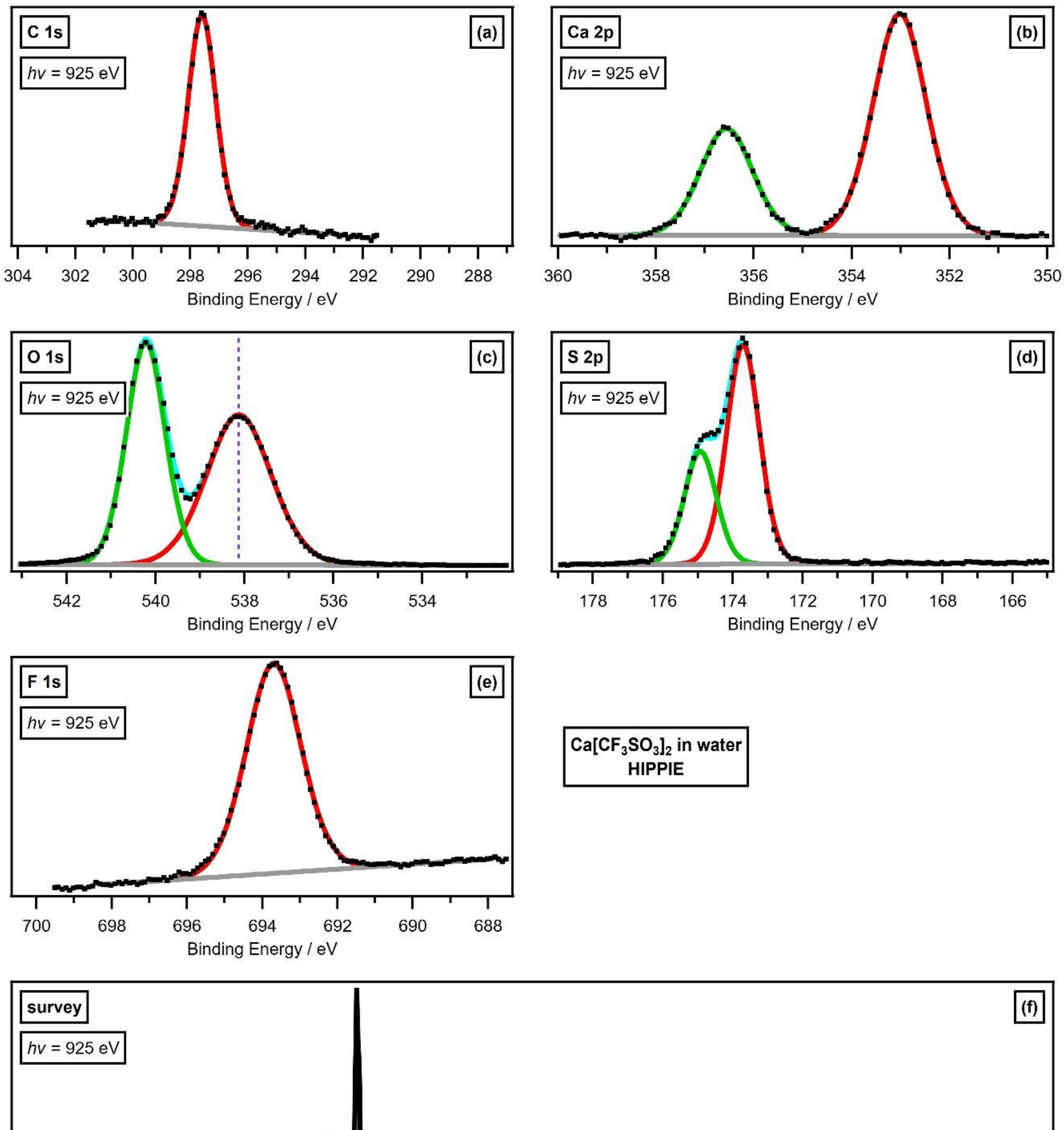

Figure S27. (a-e) Core and (f) survey XP spectra for 0.40 M $Ca[CF_3SO_3]_2$ in water recorded on synchrotron–based XPS apparatus (HIPPIE) at $h\nu$ = 925 eV. All XP spectra were charge referenced using the method outlined in ESI Section 8.1.

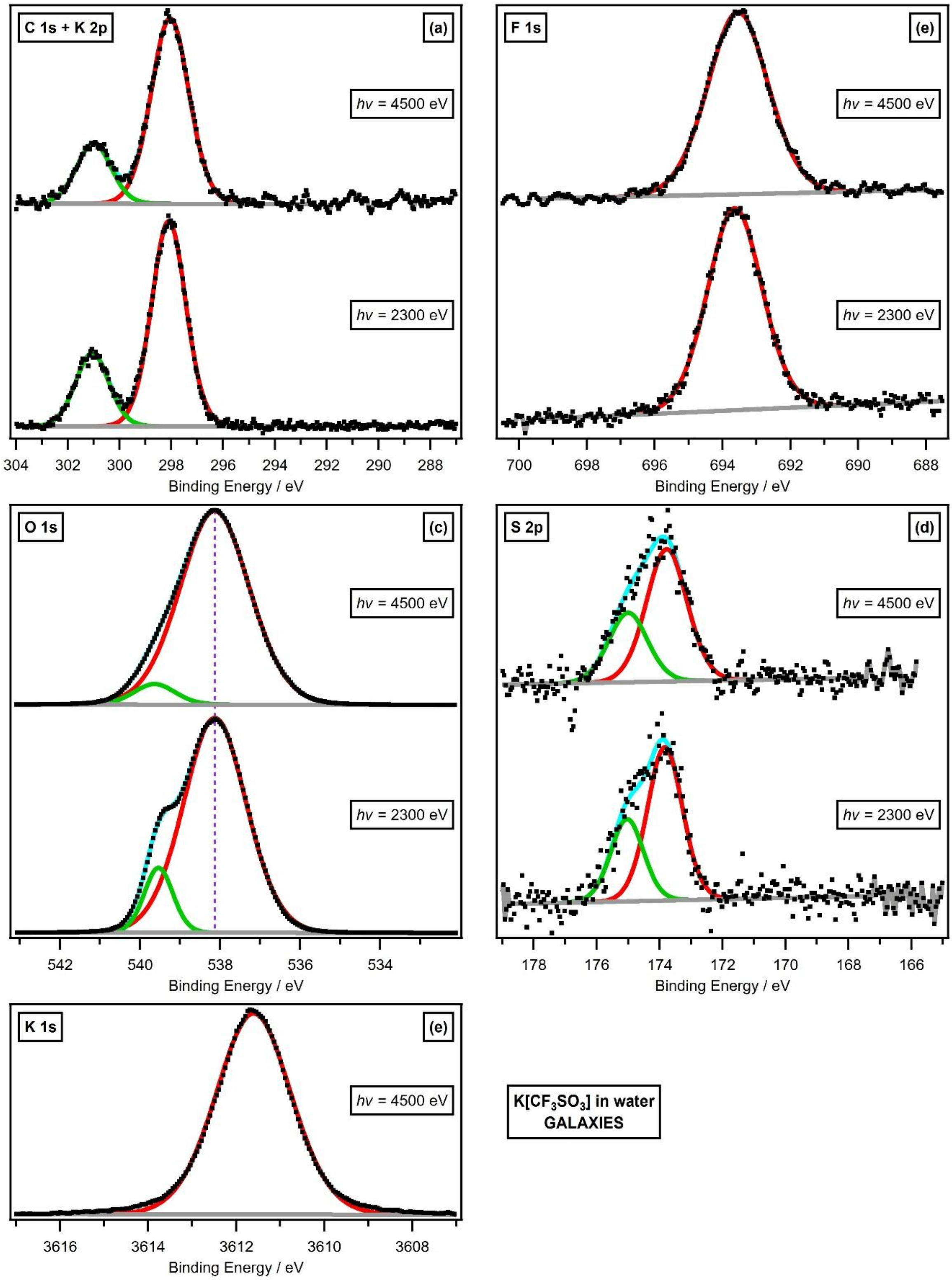

Figure S28. Core XP spectra for 0.15 M K[CF_3SO_3] in water recorded on synchrotron–based XPS apparatus (GALAXIES) at $h\nu$ = 2300 eV and 4500 eV. All XP spectra were charge referenced using the method outlined in ESI Section 8.1.

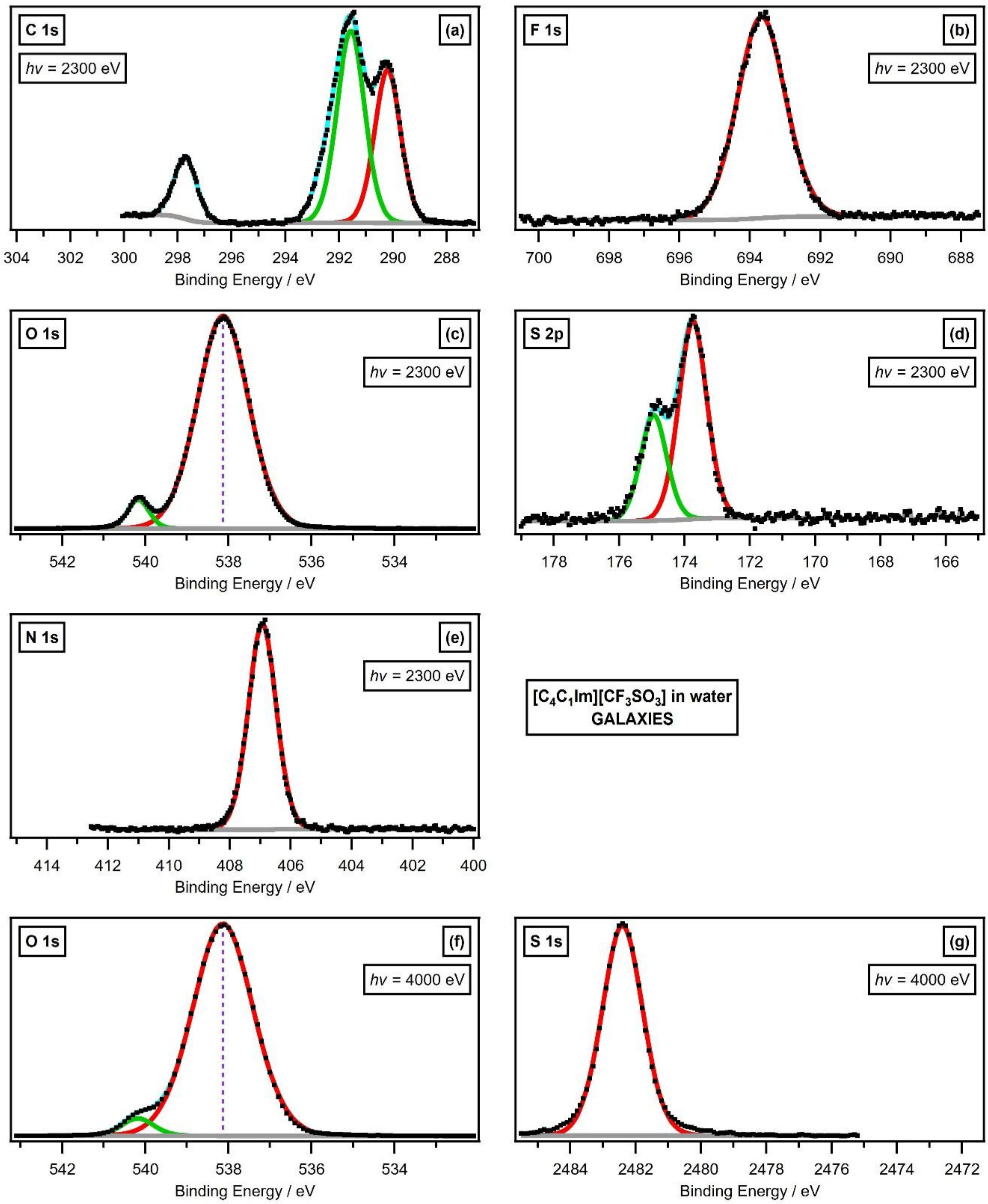

Figure S29. Core XP spectra for 0.50 M $[C_4C_1Im][CF_3SO_3]$ in water recorded on synchrotron–based XPS apparatus (GALAXIES) at $h\nu$ = 2300 eV and 4000 eV. All XP spectra were charge referenced using the method outlined in ESI Section 8.1.

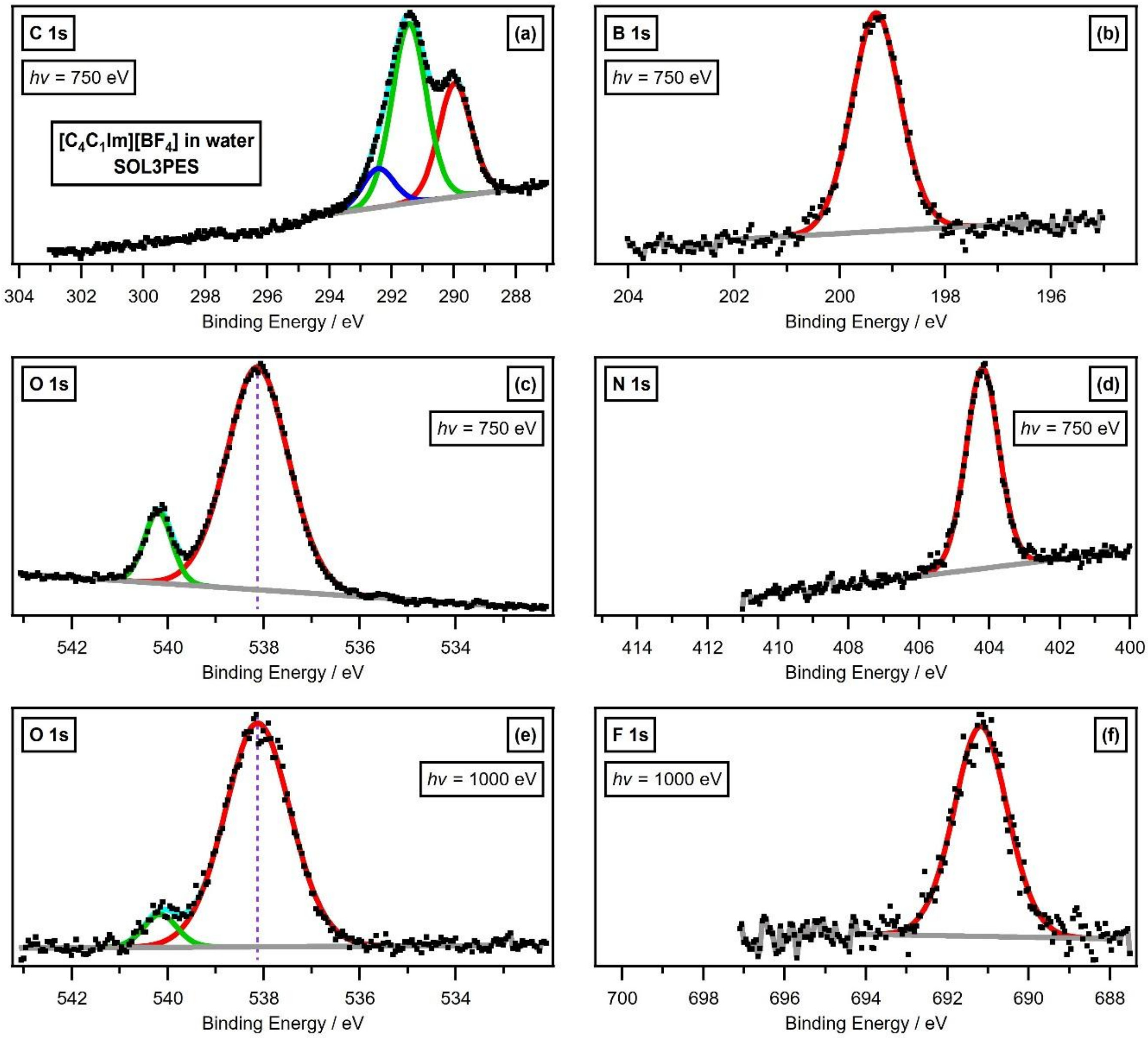

Figure S30. Core XP spectra for 0.50 M [C_4C_1Im][BF_4] in water recorded on synchrotron–based XPS apparatus (SOL3PES) at *hν* = 750 eV and 1000 eV. All XP spectra were charge referenced using the method outlined in ESI Section 8.1.

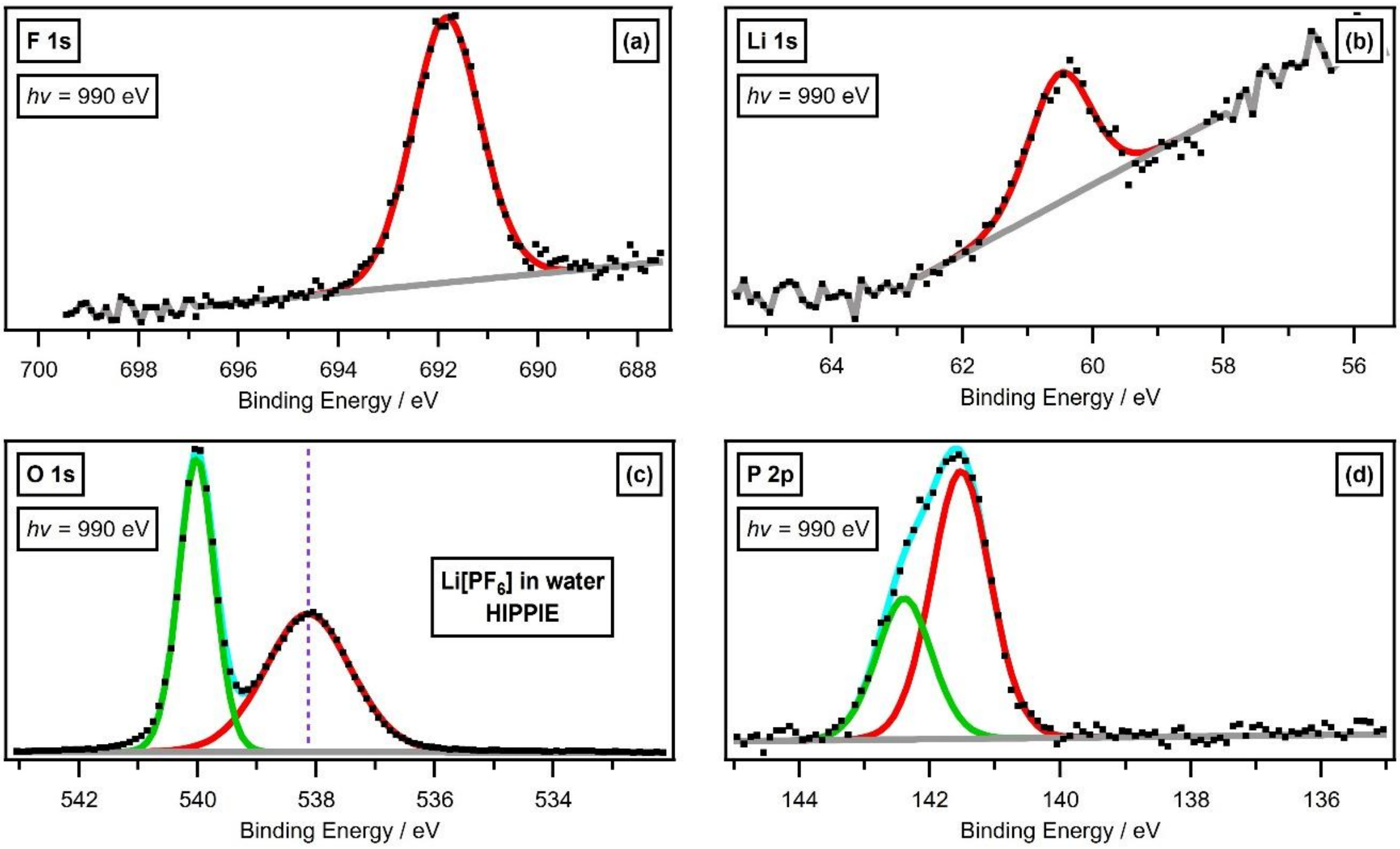

Figure S31. Core XP spectra for 0.50 M Li[PF_6] in water recorded on synchrotron–based XPS apparatus (HIPPIE) at $h\nu$ = 990 eV. All XP spectra were charge referenced using the method outlined in ESI Section 8.1.

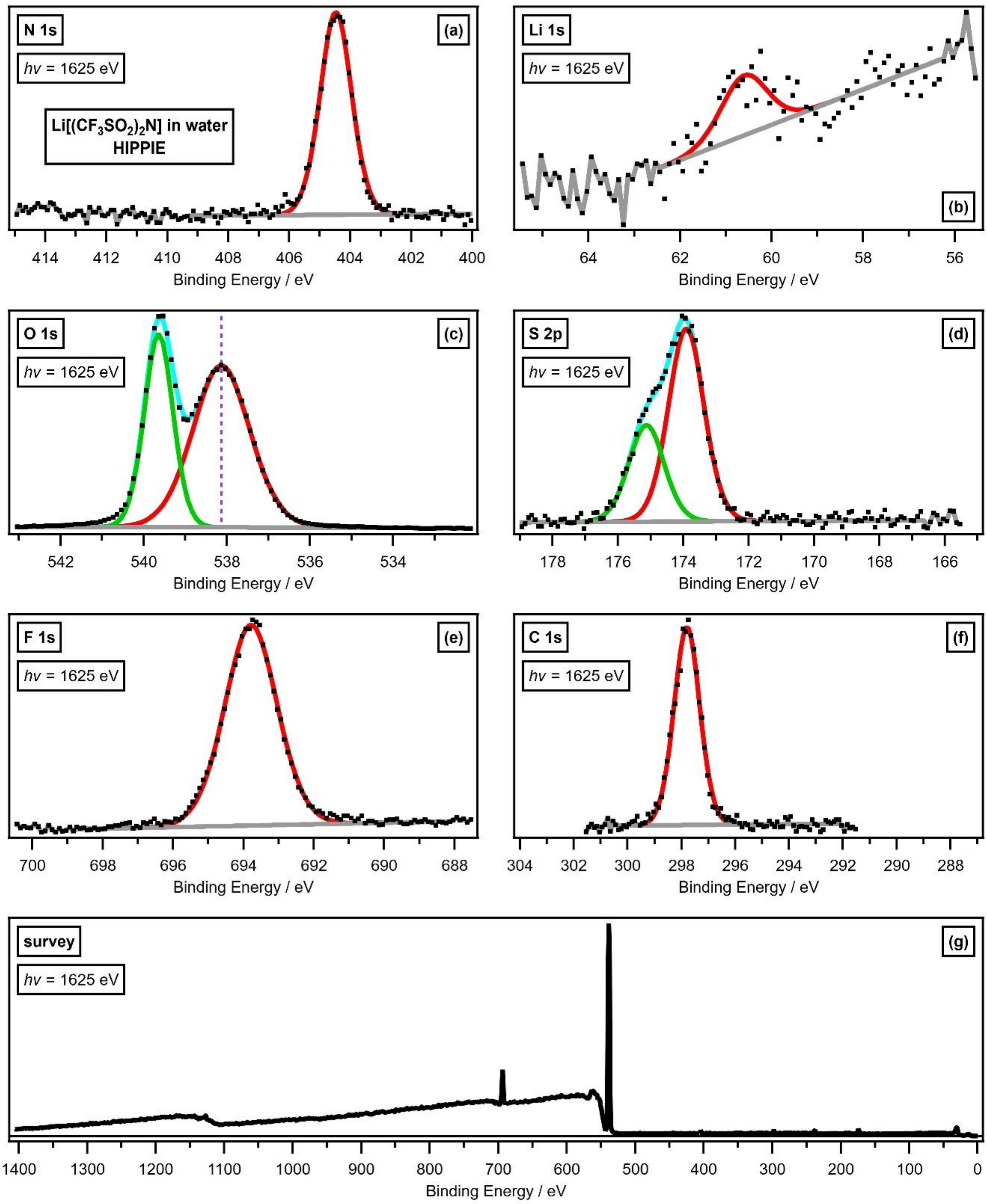

Figure S32. (a-f) Core and (g) survey XP spectra for 0.50 M Li[(CF_3SO_2)$_2$N] in water recorded on synchrotron–based XPS apparatus (HIPPIE) at $h\nu$ = 1625 eV. All XP spectra were charge referenced using the method outlined in ESI Section 8.1.

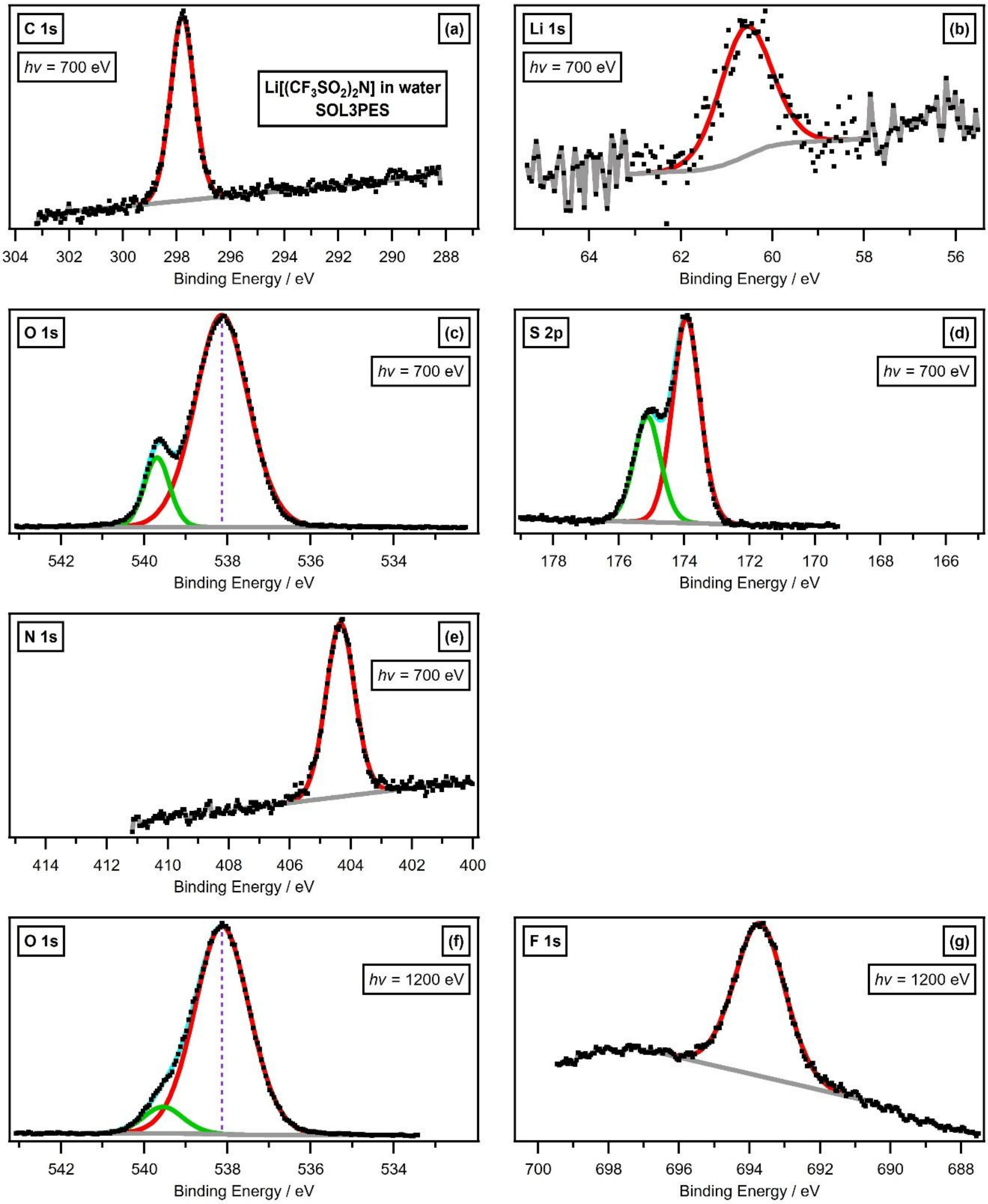

Figure S33. Core XP spectra for 0.50 M Li[($CF_3SO_2)_2$N] in water recorded on synchrotron–based XPS apparatus (SOL3PES) at $h\nu$ = 700 and 1200 eV. All XP spectra were charge referenced using the method outlined in ESI Section 8.1.

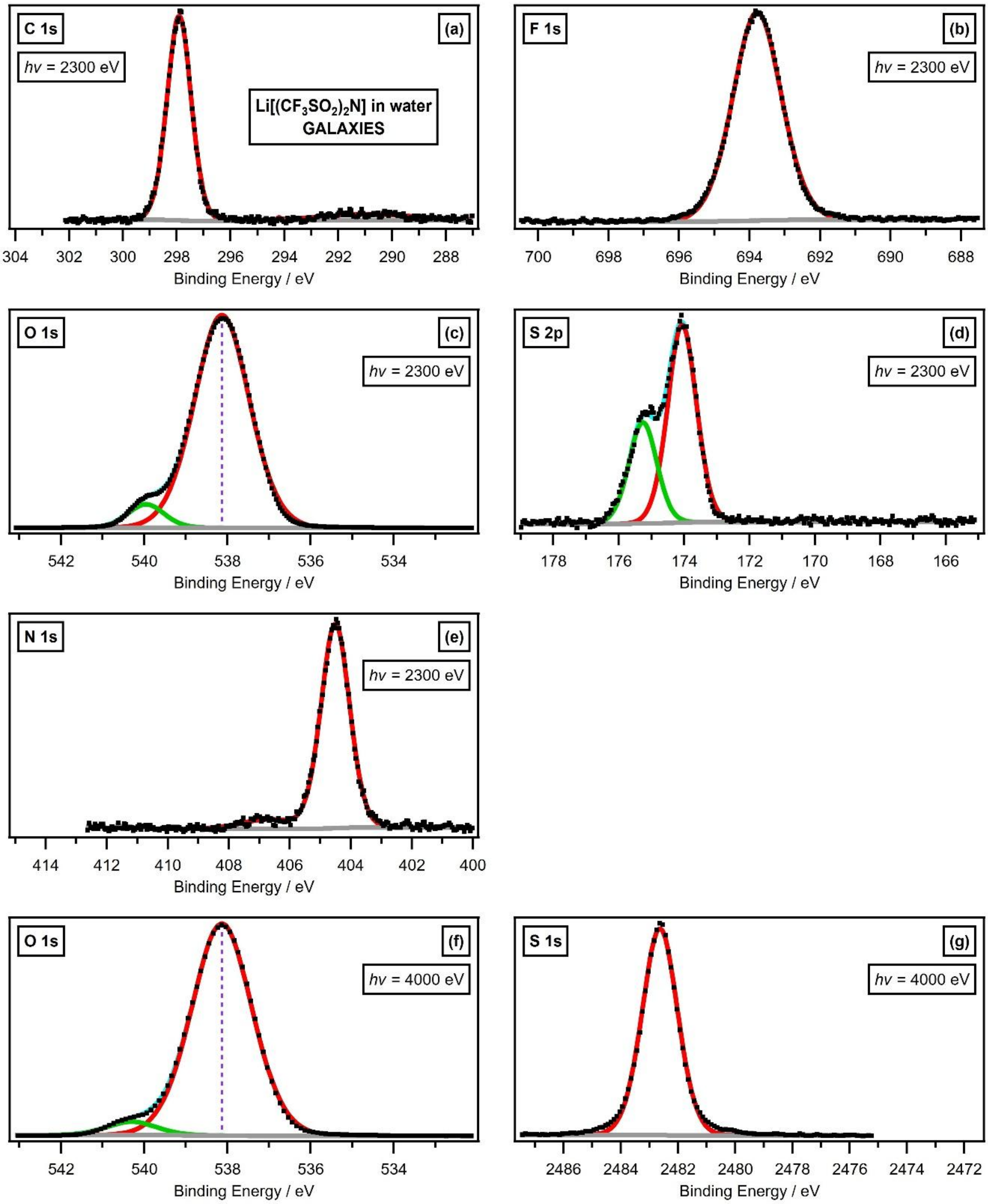

Figure S34. (a-f) Core and (g) survey XP spectra for 0.14 M Li[$(CF_3SO_2)_2N$] in water recorded on synchrotron–based XPS apparatus (GALAXIES) at $h\nu$ = 2300 and 4000 eV. All XP spectra were charge referenced using the method outlined in ESI Section 8.1.

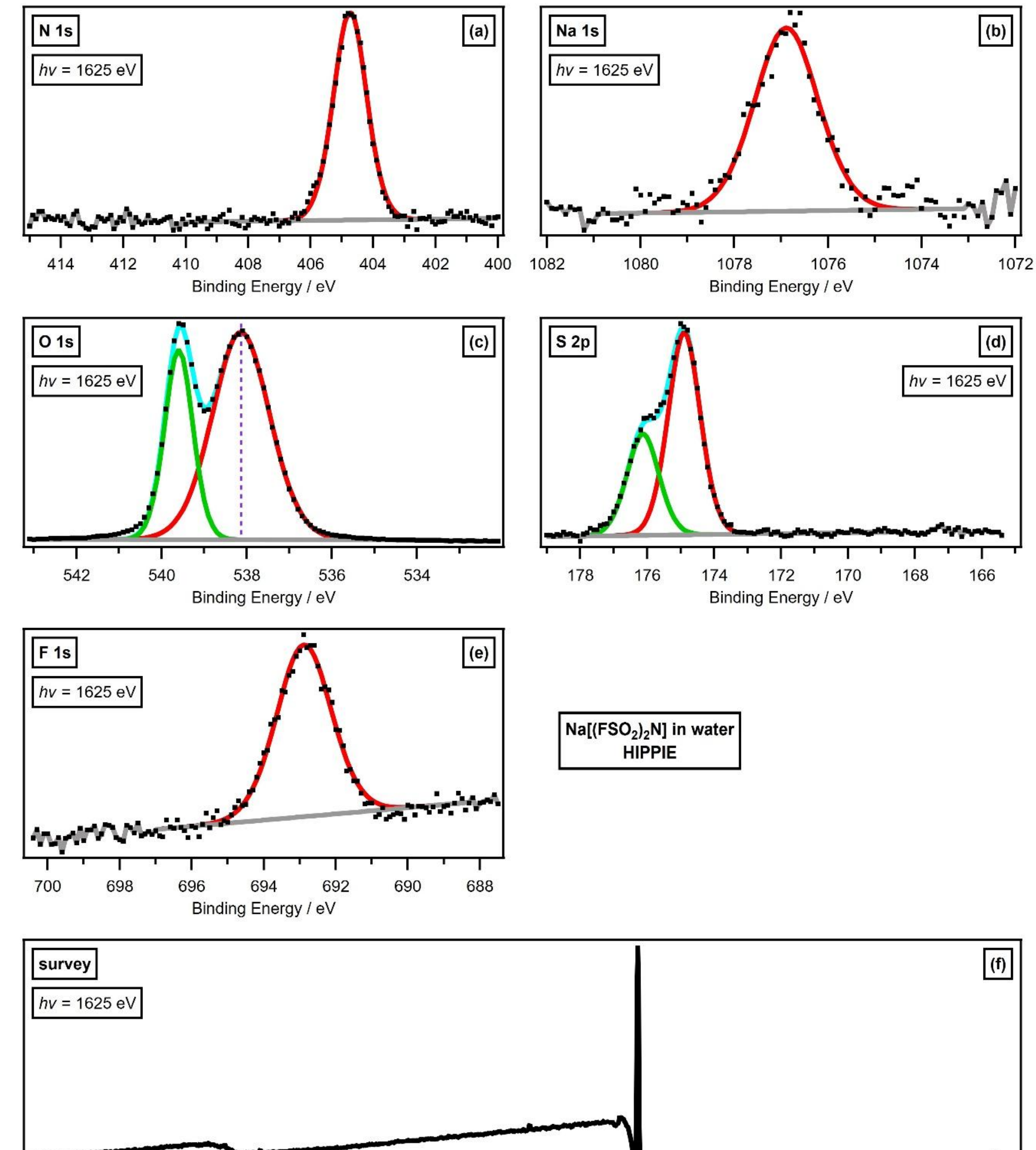

Figure S35. (a-e) Core and (f) survey XP spectra for 0.40 M $Na[(FSO_2)_2N]$ in water recorded on synchrotron–based XPS apparatus (HIPPIE) at $h\nu$ = 1625 eV. All XP spectra were charge referenced using the method outlined in ESI Section 8.1.

11. Results. XPS: demonstrating purity using XPS for new salt in PC samples

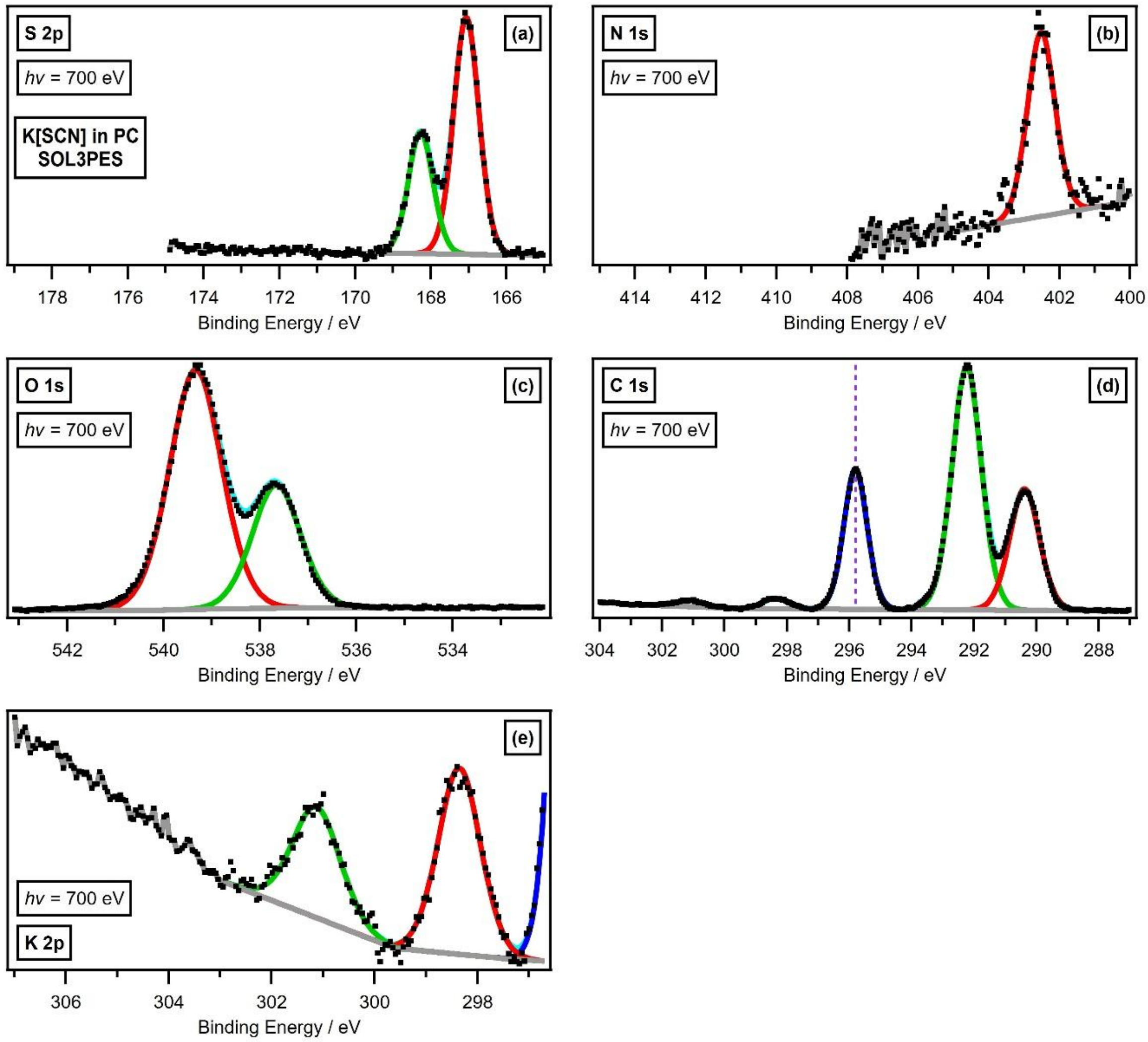

Figure S36. Core XP spectra for 0.50 M K[SCN] in PC recorded on synchrotron–based XPS apparatus (SOL3PES) at *hν* = 700 eV. All XP spectra were charge referenced using the method outlined in ESI Section 8.1.

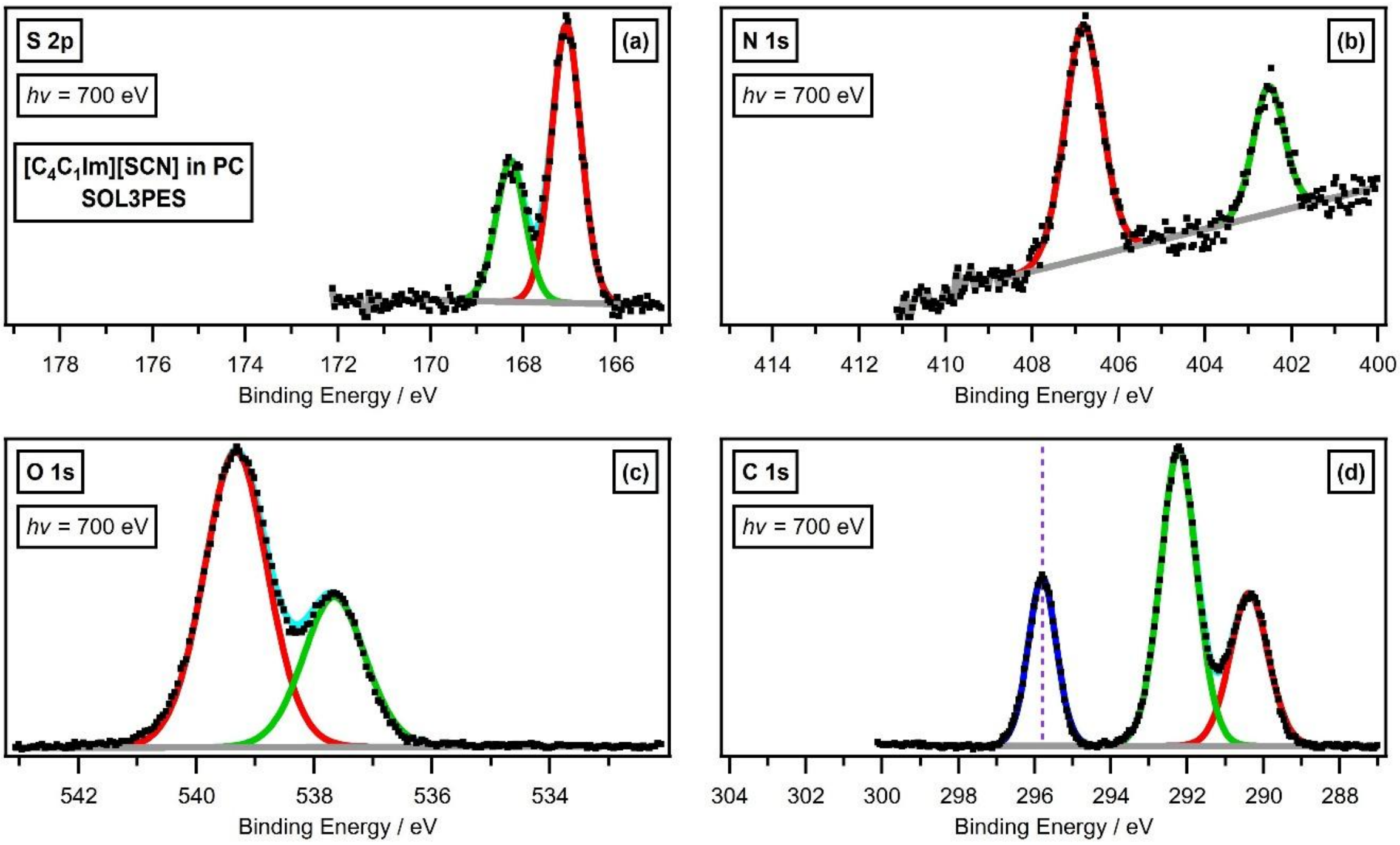

Figure S37. Core XP spectra for 0.50 M $[C_4C_1Im][SCN]$ in PC recorded on synchrotron–based XPS apparatus (SOL3PES) at $h\nu$ = 700 eV. All XP spectra were charge referenced using the method outlined in ESI Section 8.1.

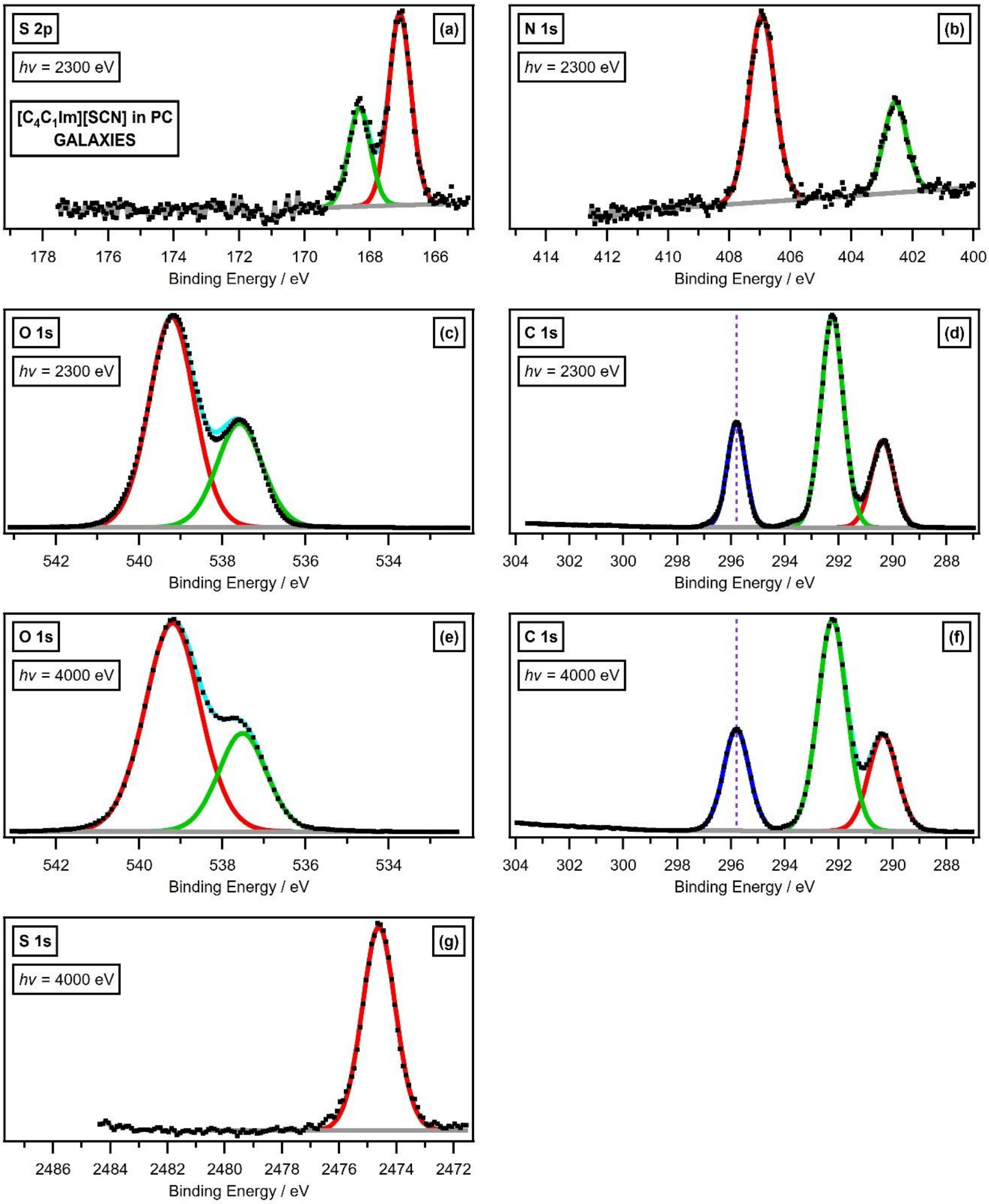

Figure S38. Core XP spectra for 0.14 M [C_4C_1Im][SCN] in PC recorded on synchrotron–based XPS apparatus (GALAXIES) at *hν* = 2300 eV and 4000 eV. All XP spectra were charge referenced using the method outlined in ESI Section 8.1.

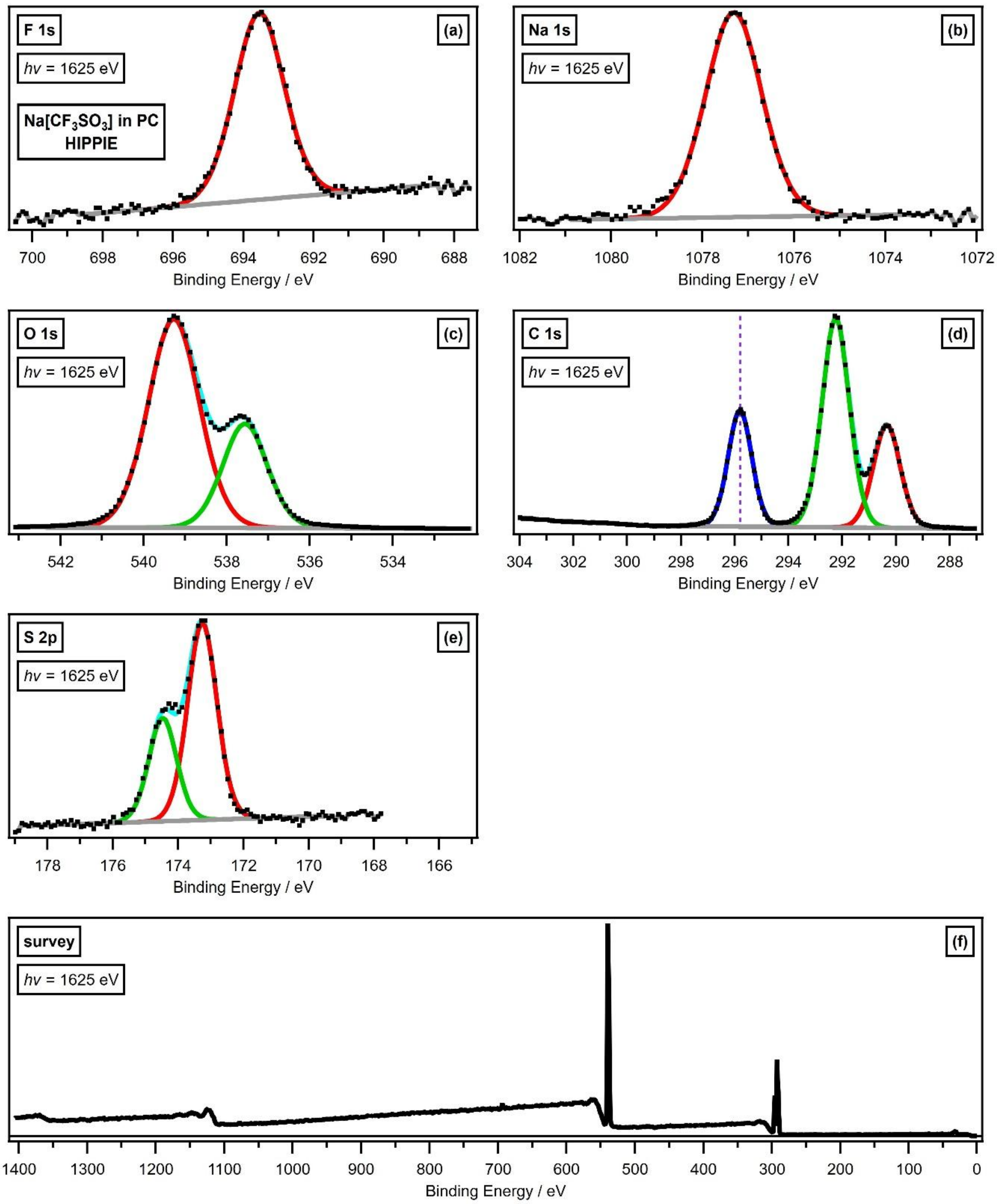

Figure S39. (a-e) Core and (f) survey XP spectra for 0.15 M Na[CF_3SO_3] in PC recorded on synchrotron–based XPS apparatus (HIPPIE) at $h\nu$ = 1625 eV. All XP spectra were charge referenced using the method outlined in ESI Section 8.1.

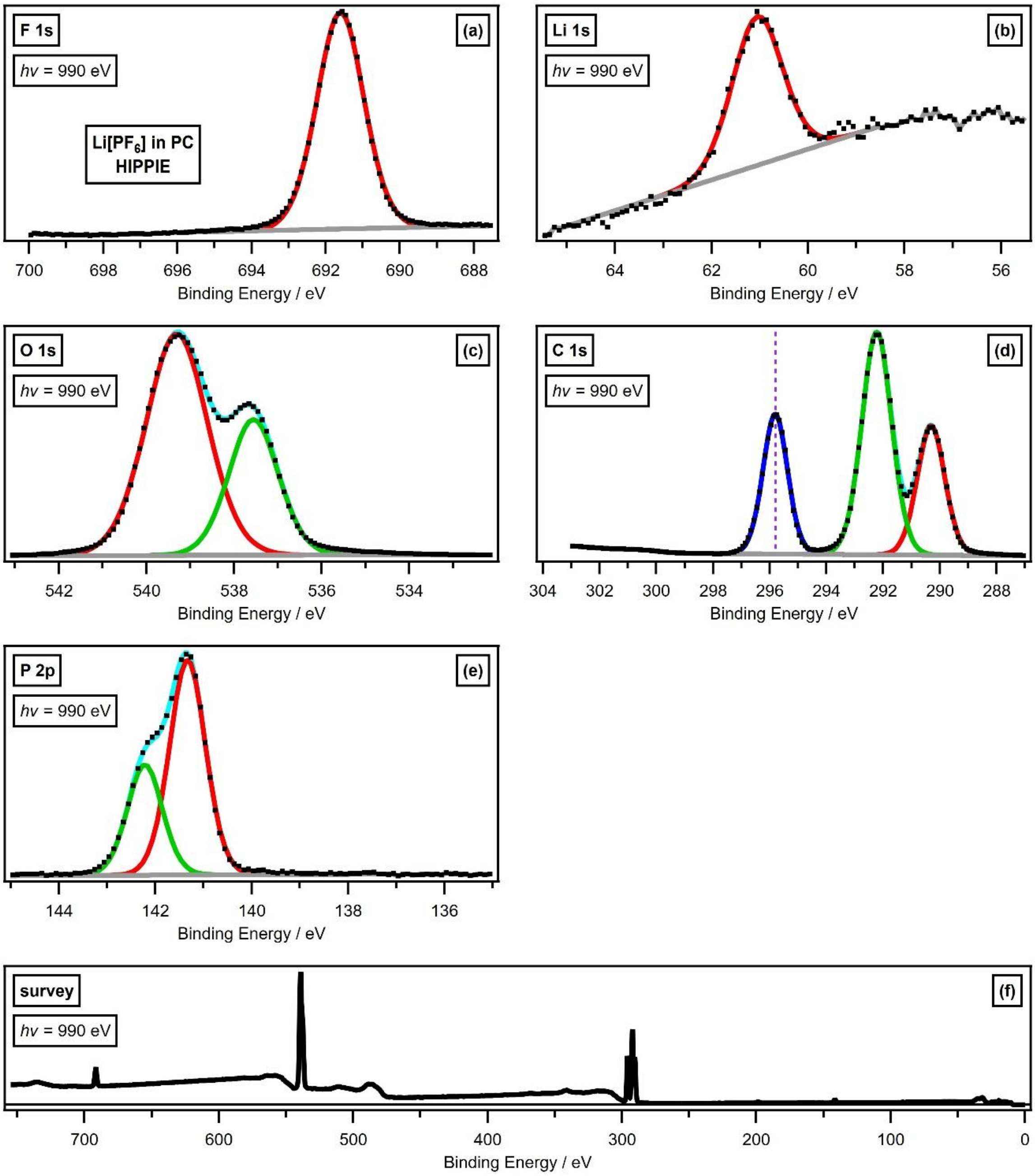

Figure S40. (a-e) Core and (f) survey XP spectra for 0.50 M Li[PF_6] in PC recorded on synchrotron–based XPS apparatus (HIPPIE) at $h\nu$ = 990 eV. All XP spectra were charge referenced using the method outlined in ESI Section 8.1.

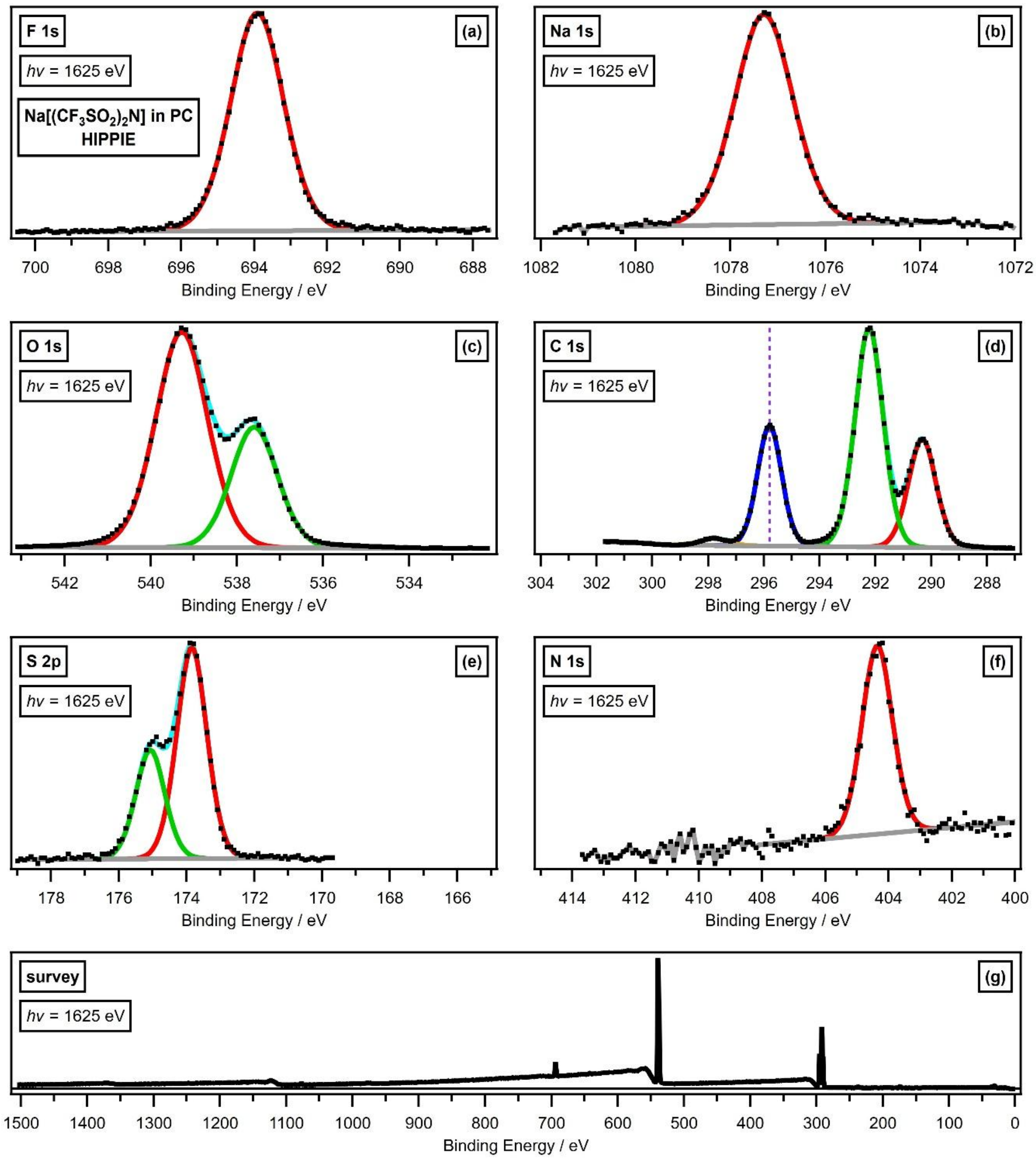

Figure S41. (a-f) Core and (g) survey XP spectra for 0.30 M $Na[(CF_3SO_2)_2N]$ in PC recorded on synchrotron–based XPS apparatus (HIPPIE) at $h\nu$ = 1625 eV. All XP spectra were charge referenced using the method outlined in ESI Section 8.1.

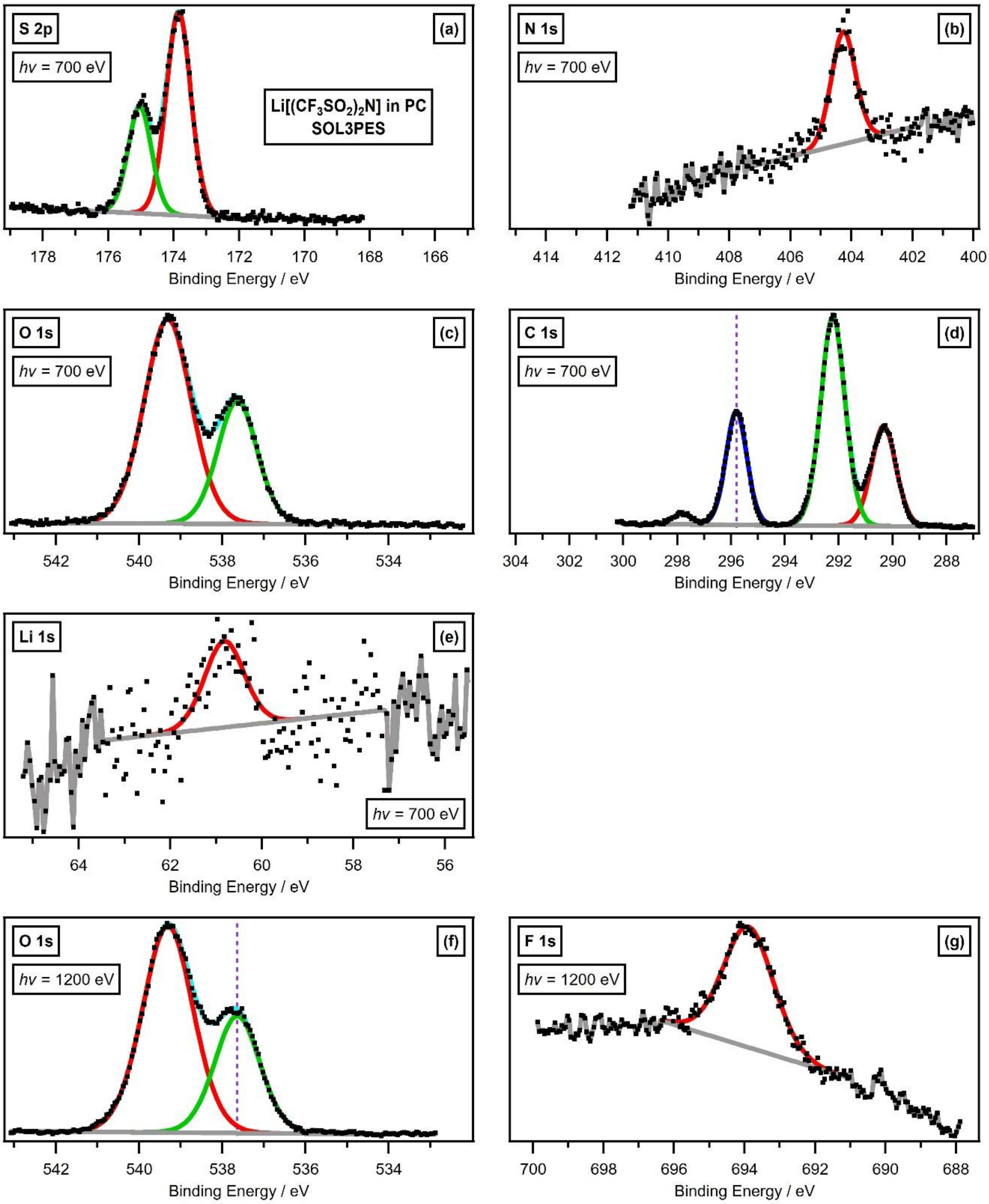

Figure S42. Core XP spectra for 0.46 M $Li[(CF_3SO_2)_2N]$ in PC recorded on synchrotron–based XPS apparatus (SOL3PES) at $h\nu$ = 700 eV and 1200 eV. All XP spectra were charge referenced using the method outlined in ESI Section 8.1.

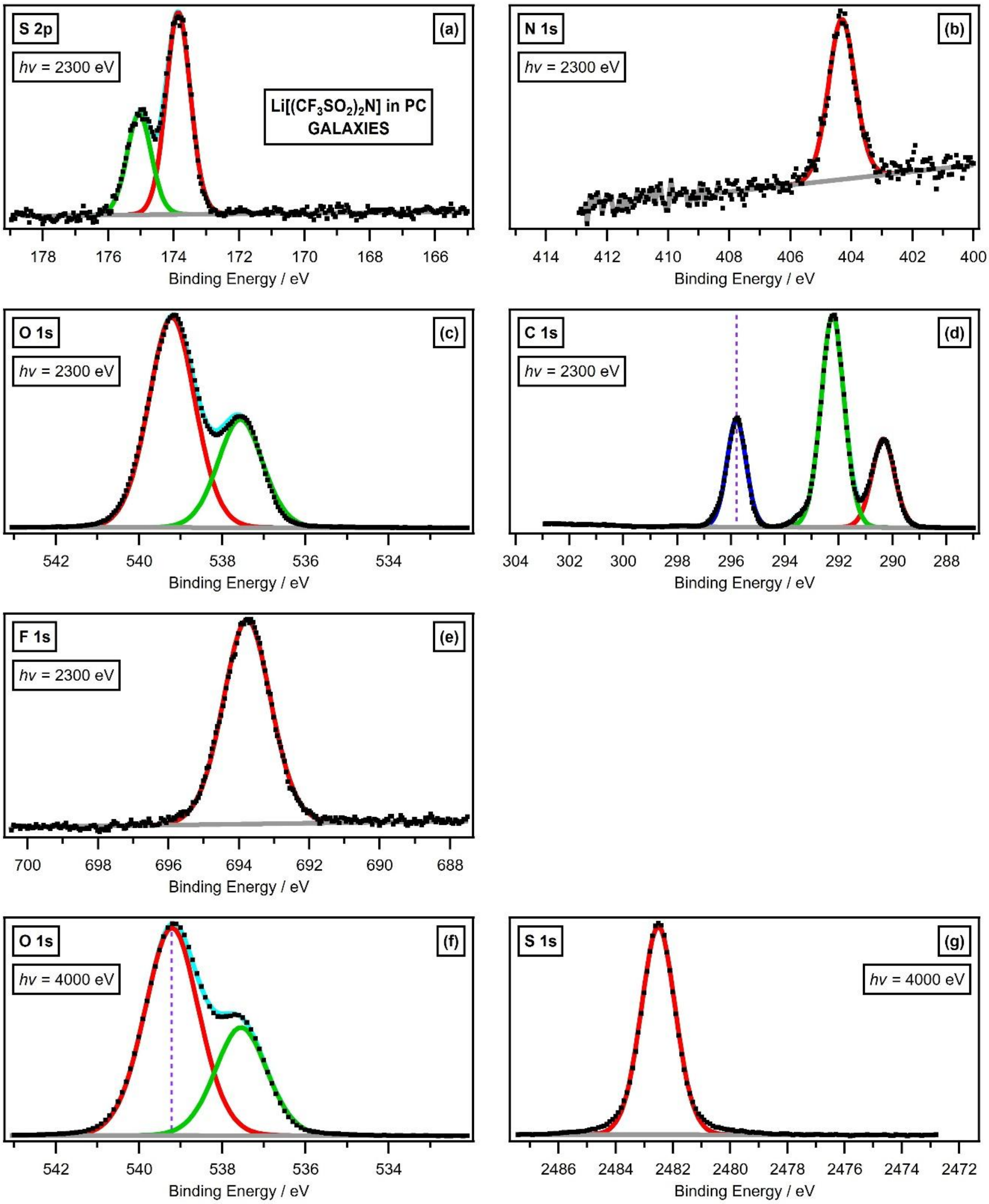

Figure S43. Core XP spectra for 0.14 M Li[$(CF_3SO_2)_2N$] in PC recorded on synchrotron–based XPS apparatus (GALAXIES) at $h\nu$ = 2300 eV and 4000 eV. All XP spectra were charge referenced using the method outlined in ESI Section 8.1.

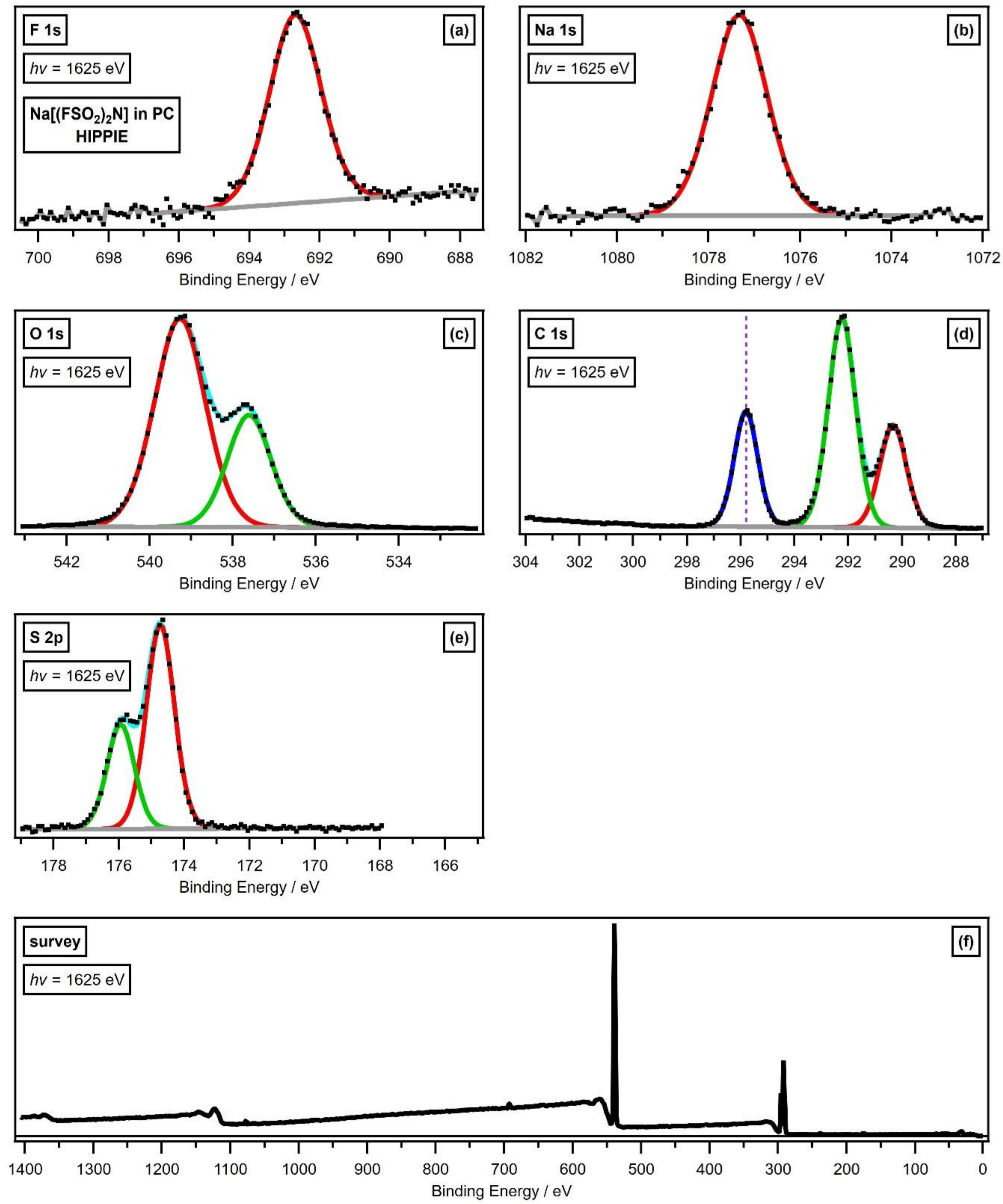

Figure S44. (a-e) Core and (f) survey XP spectra for 0.30 M $Na[(FSO_2)_2N]$ in PC recorded on synchrotron–based XPS apparatus (HIPPIE) at $h\nu$ = 1625 eV. All XP spectra were charge referenced using the method outlined in ESI Section 8.1.

12. Results. XPS: demonstrating purity using XPS for new salt in TEP samples

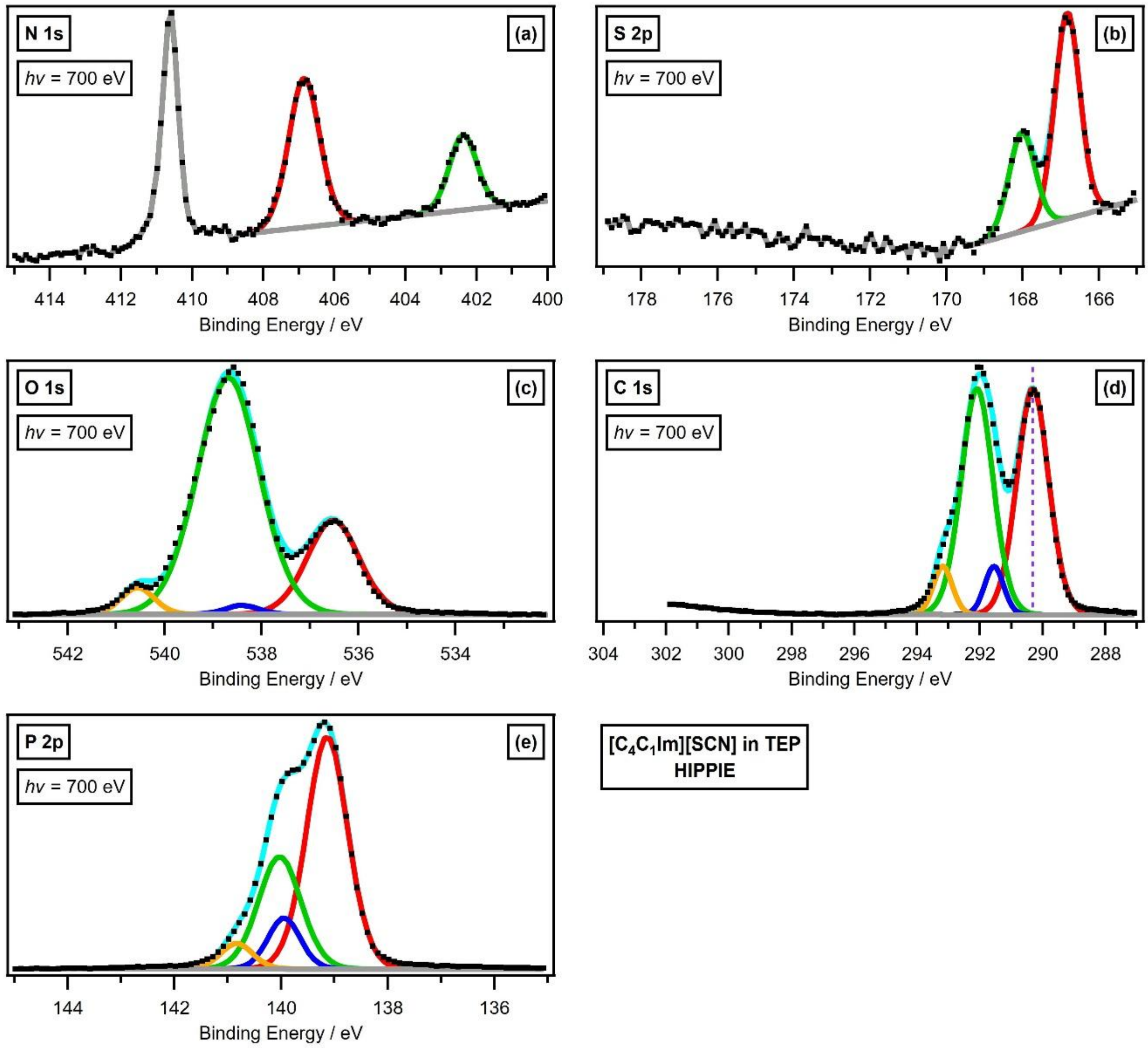

Figure S45. Core XP spectra for 0.18 M [C_4C_1Im][SCN] in TEP recorded on synchrotron–based XPS apparatus (HIPPIE) at $h\nu$ = 700 eV. All XP spectra were charge referenced using the method outlined in ESI Section 8.1. The N 1s XP spectrum has a small gas phase N_2 peak at E_B ~410.5 eV.

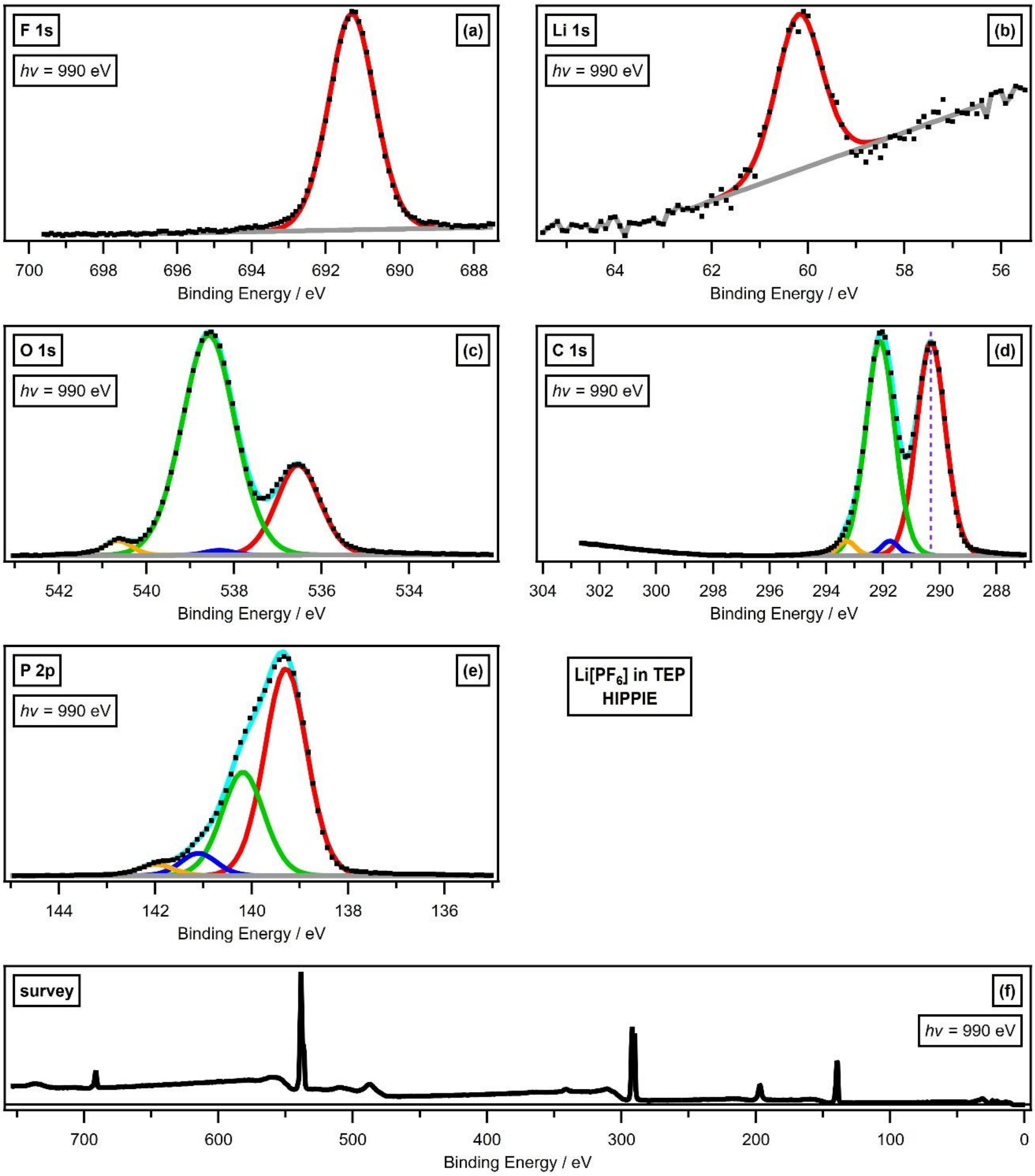

Figure S46. (a-e) Core and (f) survey XP spectra for 0.50 M Li[PF_6] in TEP recorded on synchrotron–based XPS apparatus (HIPPIE) at $h\nu$ = 990 eV. All XP spectra were charge referenced using the method outlined in ESI Section 8.1.

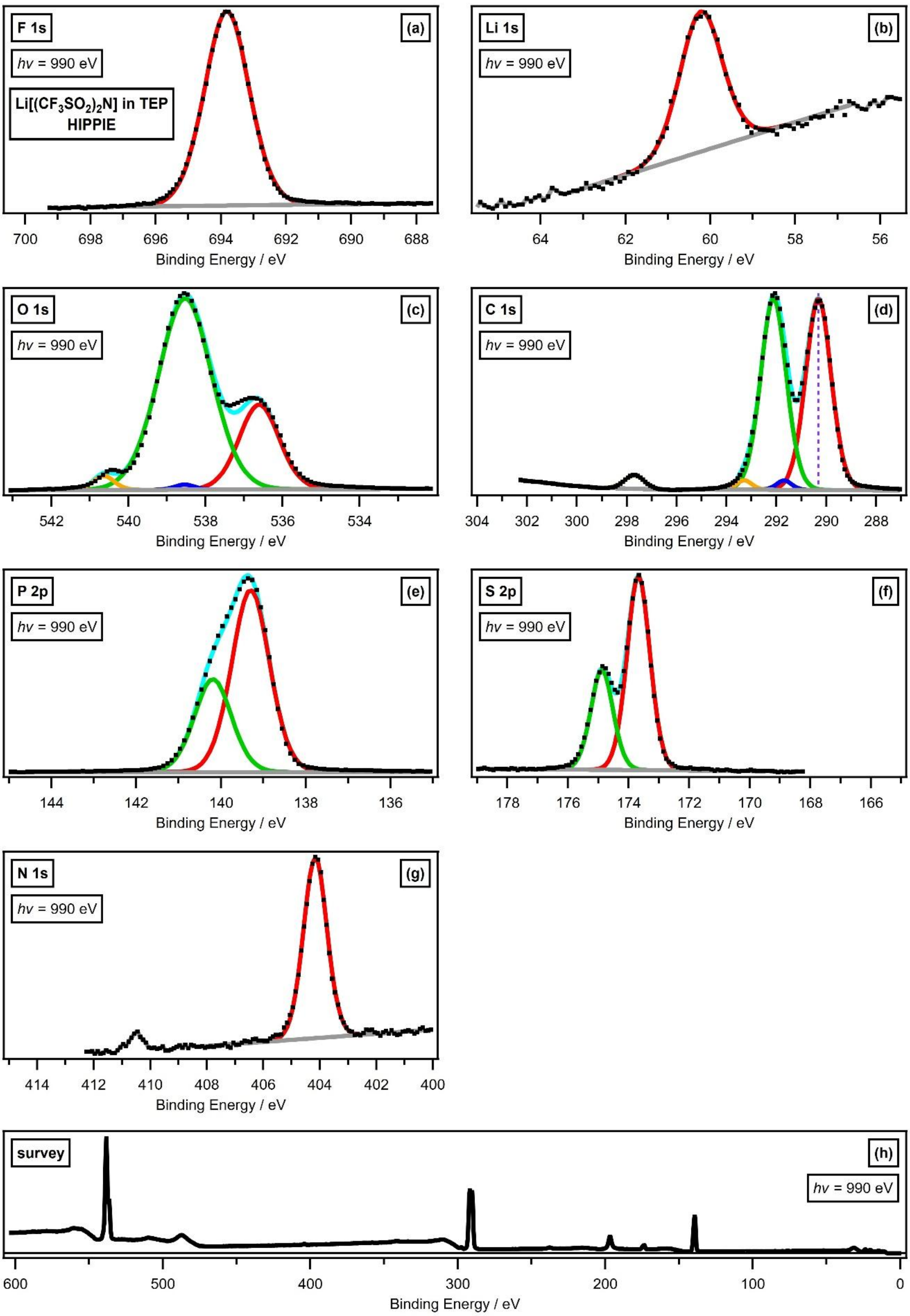

Figure S47. (a-g) Core and (h) survey XP spectra for 0.48 M Li[$(CF_3SO_2)_2N$] in TEP recorded on synchrotron–based XPS apparatus (HIPPIE) at $h\nu$ = 990 eV. All XP spectra were charge referenced using the method outlined in ESI Section 8.1. The N 1s XP spectrum has a small gas phase N_2 peak at E_B ~410.5 eV.

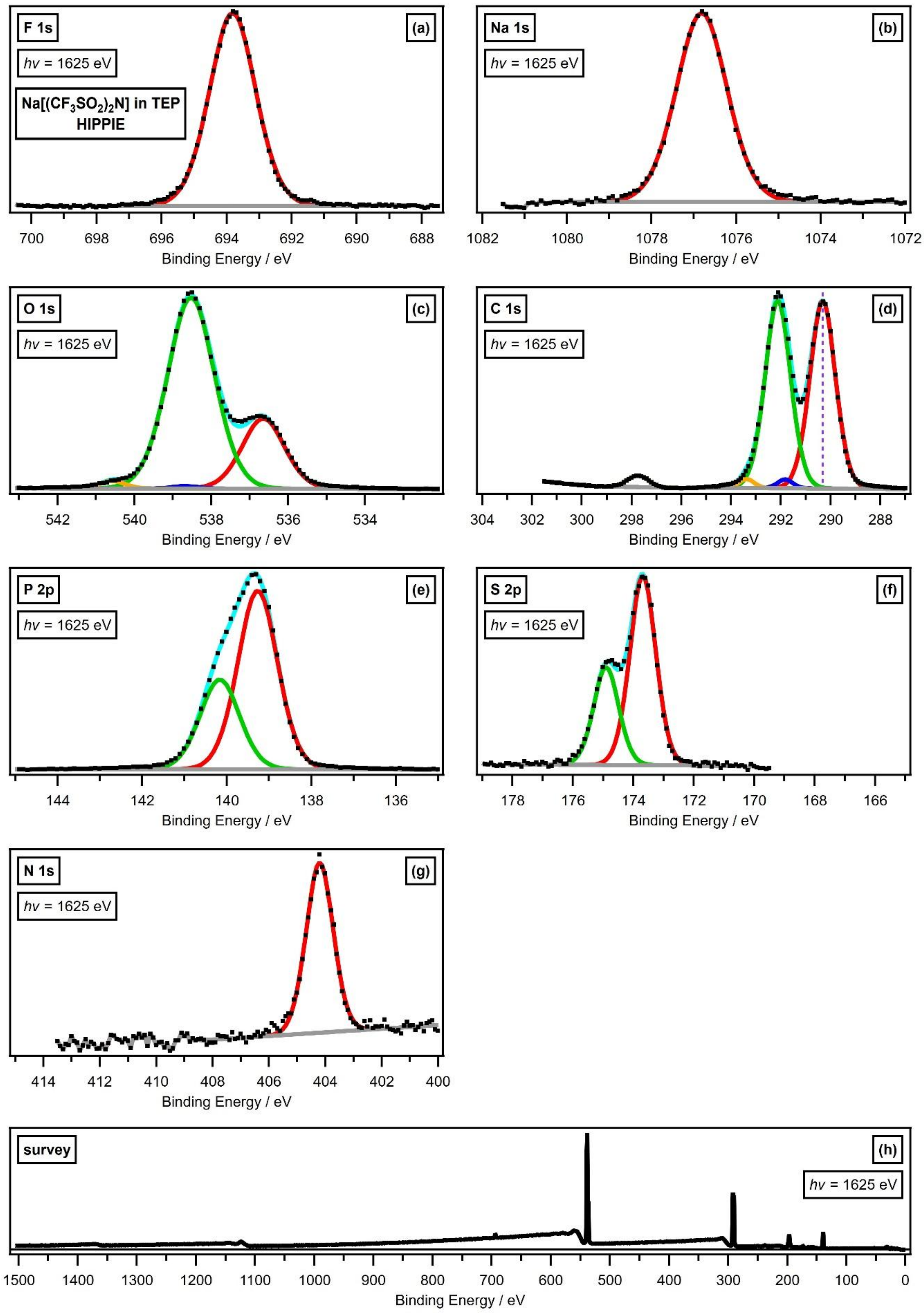

Figure S48. (a-g) Core and (h) survey XP spectra for 0.50 M $Na[(CF_3SO_2)_2N]$ in TEP recorded on synchrotron–based XPS apparatus (HIPPIE) at $h\nu$ = 990 eV. All XP spectra were charge referenced using the method outlined in ESI Section 8.1.

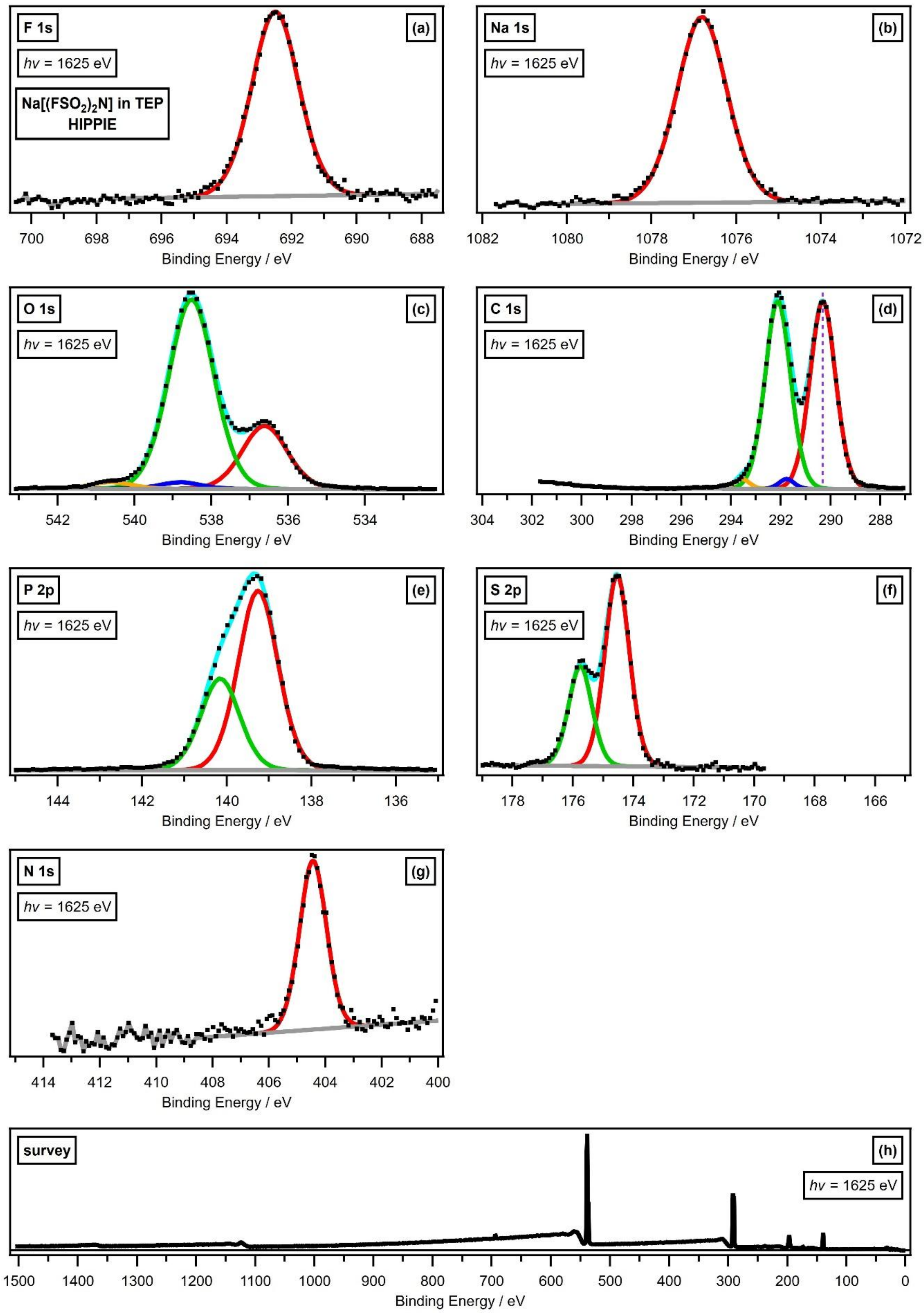

Figure S49. (a-g) Core and (h) survey XP spectra for 0.50 M $Na[(FSO_2)_2N]$ in TEP recorded on synchrotron–based XPS apparatus (HIPPIE) at $h\nu$ = 990 eV. All XP spectra were charge referenced using the method outlined in ESI Section 8.1.

13. Results. Anion element-specific donor numbers: experimental uncertainty

13.1. Large experimental uncertainty in literature anion descriptors

An important part of producing a new experimental descriptor, in our opinion at least, is demonstrating reproducibility of the descriptor measurement and the experimental uncertainty.

Some experimental anion descriptors suffer from a lack of reproducibility/relatively large experimental uncertainty by different research groups, *e.g.* for $[CH_3CO_2]^-$ (with a $[C_4C_1Im]^+$ countercation) β using the same heterocyclic dye molecule probes (so not a probe-dependent effect) gave 1.05, 1.18 and 1.20.[2, 25, 26] it is unclear whether this problem is due to sample impurities or spectroscopic difficulties.

XPS has a huge problem of data reproducibility, as E_B for the same sample can give very different E_B, *e.g.* solid $[NH_4][NO_3]$ gives an E_B(N_{anion} 1s) range of 1.8 eV for four literature values;[27-30] the source (or sources) of these differences is unclear.

For ionic liquids, E_B experimental uncertainties are quoted as ±0.15 eV [8, 24, 31] or ±0.10 eV,[21, 22, 32] but that usually relates to one group measuring on one XPS apparatus; it is unclear if E_B reproducibility holds across multiple XPS apparatus, particularly under different measurements conditions (*e.g.* different source $h\nu$, different analyser E_B calibration). For anions in molecular solvents, there is insufficient E_B data to judge reproducibility; producing comparable E_B is a significant challenge for XPS in water and other molecular liquids.[33]

13.2. Small experimental uncertainty in our anion descriptors

Matching E_B(core,anion) for the same sample can be produced once charge referencing has been carried out, within a relatively small experimental uncertainty; less than ±0.10 eV in most cases (ESI Figure S50 to S53). This small experimental uncertainty is achieved when using different XPS apparatus, including fixed $h\nu$ sources versus variable $h\nu$ synchrotron sources ($h\nu$ ranging from soft X-rays through to tender X-rays and towards hard X-rays), and also for both ionic liquid and molecular solvent samples.

We use ±0.10 eV as the experimental uncertainty here, as an upper limit of what we observe as explained in the previous paragraph. There are larger experimental uncertainties for: (i) the oligomeric chlorozincate anions with ±0.15 eV given the challenges fitting these Cl 2p XP spectra;[15] (ii) $[SbF_6]^-$ and $[FeCl_4]^-$ with ±0.15 eV as E_B(Cl_{anion} $2p_{3/2}$) for these two anions are reported to only one decimal place in references [13] and [16] respectively; (iii) $[LiCl_3]^{2-}$ with ±0.20 eV given the free Cl^- also present in solution; (iv) $[Co(NCS)_4]^{2-}$ with ±0.15 eV as E_B(S_{anion} $2p_{3/2}$) for this anion is reported to only one decimal place in reference [18].

We have measured the ionic liquid $[C_8C_1Im][(CF_3SO_2)_2N]$ six times on five different XPS apparatus (two datasets already published,[9, 12] ESI Figure S50). E_B(core,anion) match very well, with standard errors of less than 0.05 eV for all core-levels measured (ESI Table S22 to Table S27). Two datasets of E_B(core,anion) for $[C_8C_1Im][(CF_3SO_2)_2N]$ are available in the literature,[8, 22] and our data here matches to that published data, giving further confidence in E_B(core,anion) reproducibility and experimental uncertainty of less than ±0.10 eV.

We have measured $Li[(CF_3SO_2)_2N]$ diluted in water on three different synchrotron liquid-jet XPS apparatus (ESI Figure S51) and again E_B(core,anion) match very well, with standard errors of 0.05 eV or less for all core-levels measured (ESI Table S31).

We have measured $[C_4C_1Im][SCN]$ diluted in PC on two different synchrotron liquid-jet XPS apparatus (ESI Figure S52) and again E_B(core,anion) match very well, with standard errors of 0.05 eV or less for all core-levels measured (ESI Table S32).

We have measured $Li[(CF_3SO_2)_2N]$ diluted in PC on two different synchrotron liquid-jet XPS apparatus (ESI Figure S53) and again E_B(core,anion) match very well, with standard errors of 0.05 eV or less for all core-levels measured (ESI Table S33).

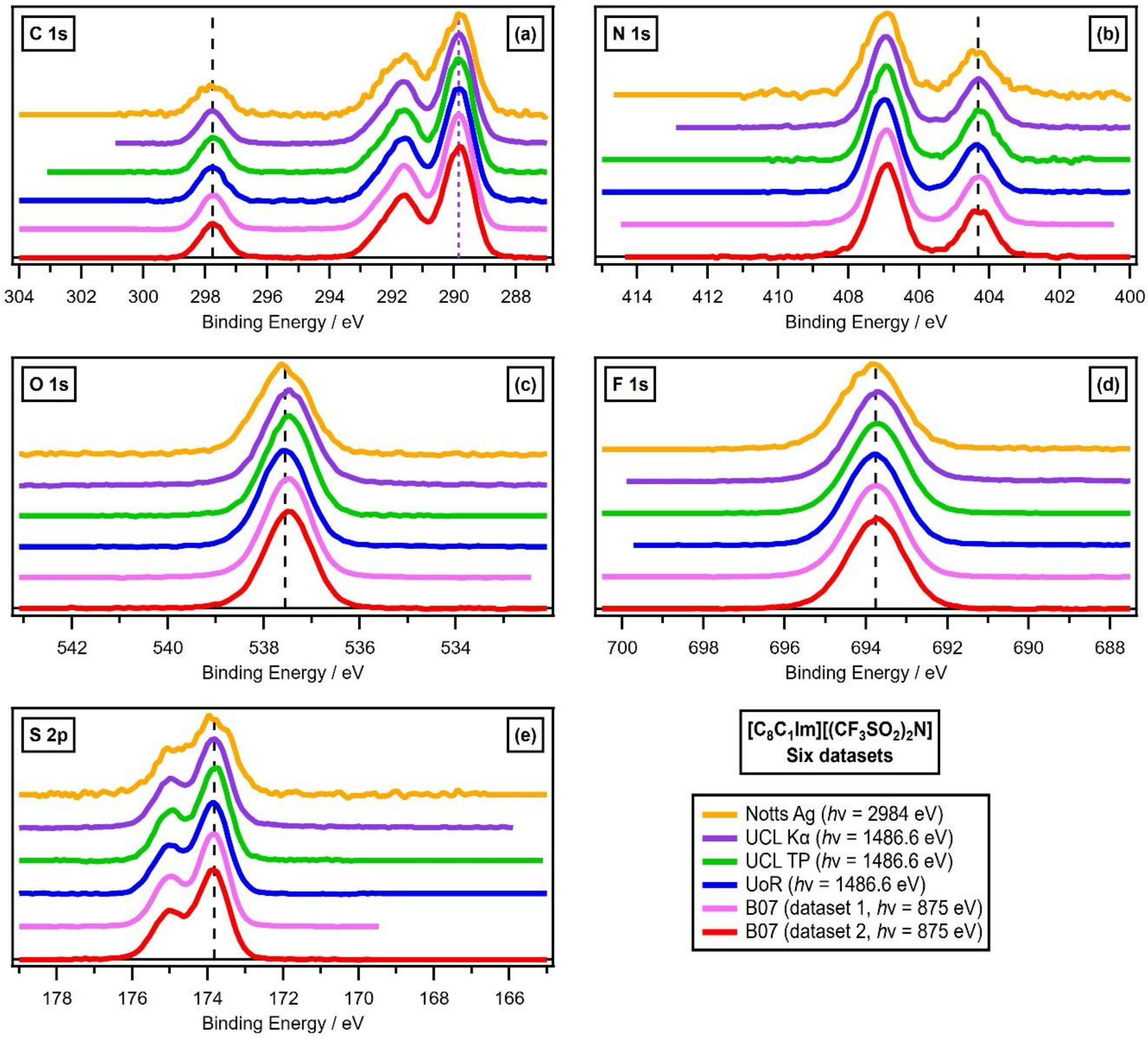

Figure S50. Core XP spectra for $[C_8C_1Im][(CF_3SO_2)_2N]$ recorded on laboratory- or synchrotron–based XPS apparatus at $h\nu$ ranging from 875 eV to 2984 eV. Spectra are offset vertically for clarity. All XP spectra were charge referenced and area normalized using the method outlined in ESI Section 8. The Nottingham Ag source and UCL Kα datasets have already been published.[9, 12]

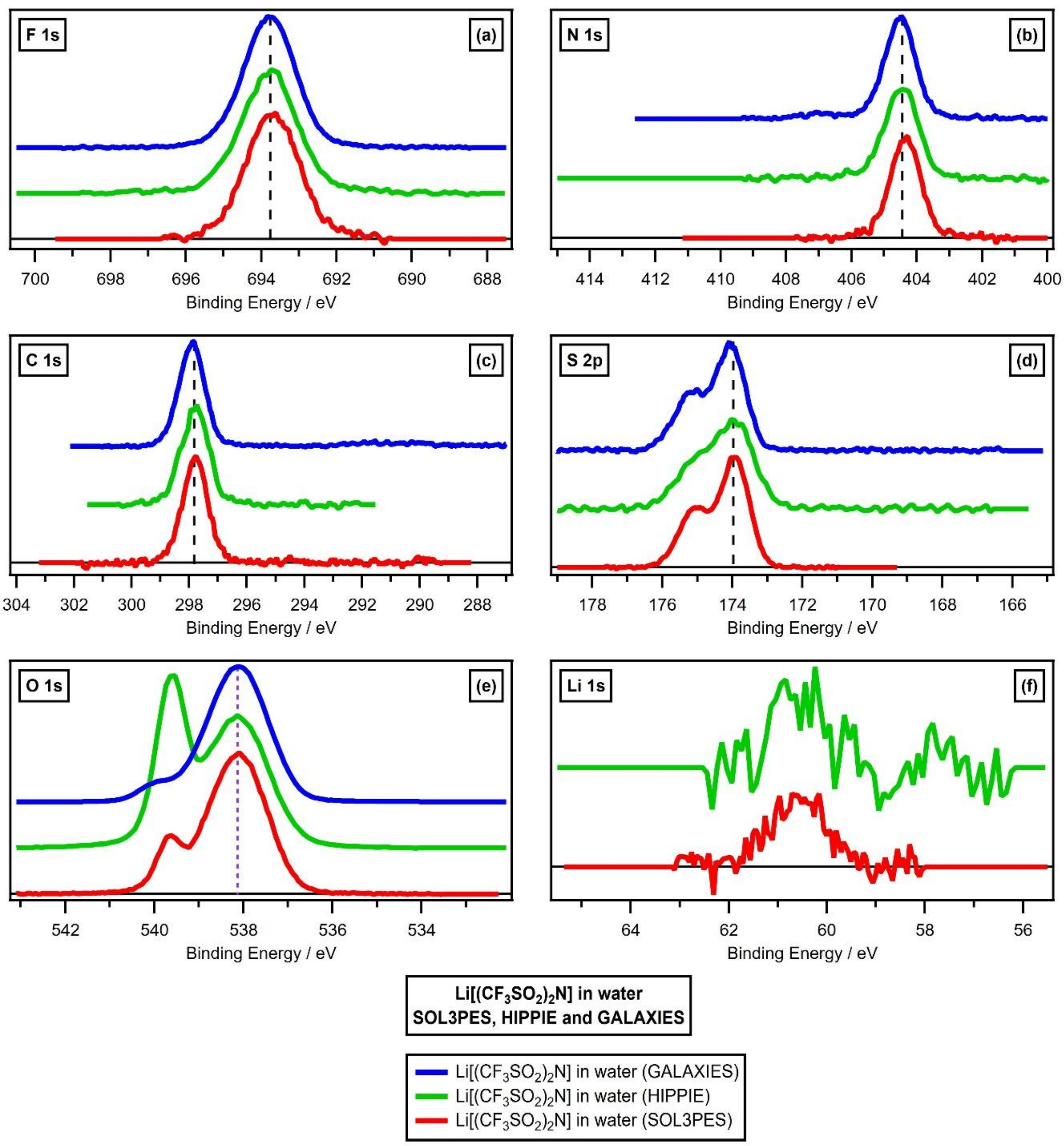

Figure S51. Core XP spectra for Li[$(CF_3SO_2)_2N$] in water recorded on synchrotron–based XPS apparatus at various $h\nu$. Spectra are offset vertically for clarity. All XP spectra were charge referenced using the method outlined in ESI Section 8.

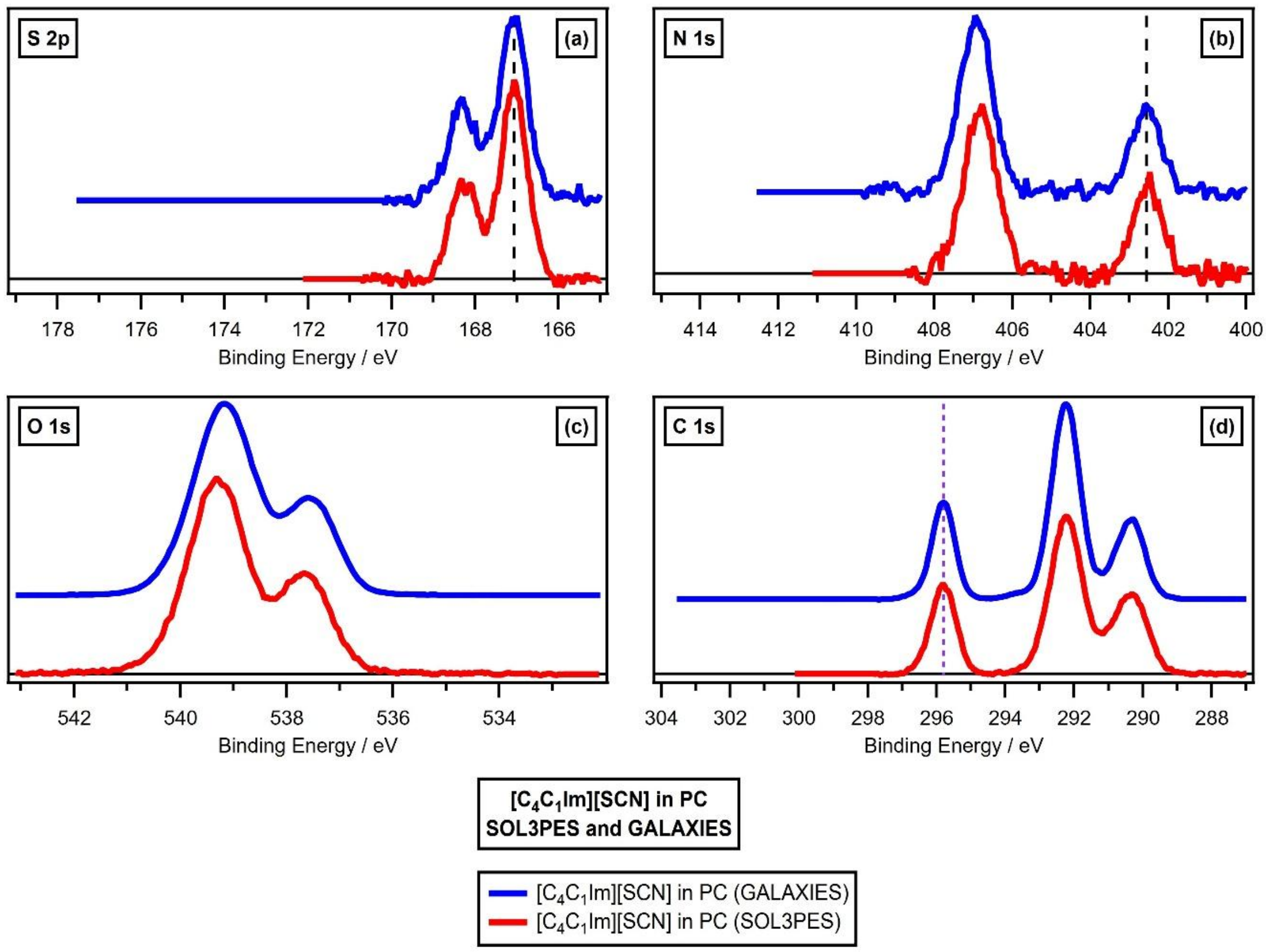

Figure S52. Core XP spectra for $[C_4C_1Im][SCN]$ in PC recorded on synchrotron–based XPS apparatus at various *h ν*. Spectra are offset vertically for clarity. All XP spectra were charge referenced using the method outlined in ESI Section 8.

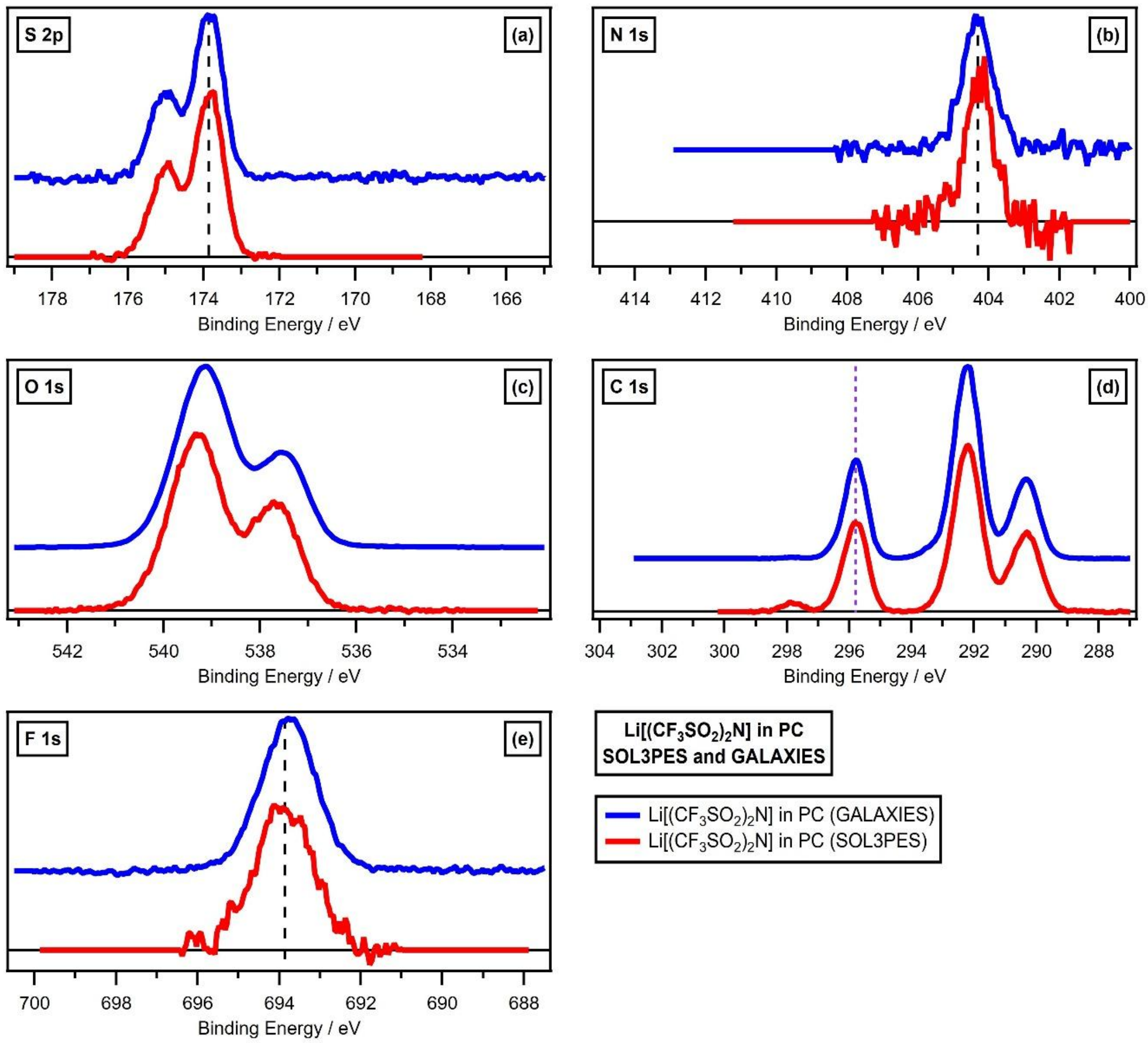

Figure S53. Core XP spectra for Li[$(CF_3SO_2)_2N$] in PC recorded on synchrotron–based XPS apparatus at various *h ν*. Spectra are offset vertically for clarity. All XP spectra were charge referenced using the method outlined in ESI Section 8.

13.3. Possible influence of contaminants on anion descriptors

For ionic liquids, contaminants are very unlikely to influence E_B(core,anion) strongly. The impact of 10% of Cl^- (a possible contaminant from synthesis) in [C_8C_1Im][$(CF_3SO_2)_2N$] on E_B(core,anion) for [$(CF_3SO_2)_2N$]$^-$ is well within experimental uncertainty.[21] Much more likely to cause problems are contaminant contributions overlapping with anion contributions to make measuring E_B(core,anion) problematic, *e.g*. Si-O grease contaminant that has been observed in ionic liquid drop XPS.[31, 34]

For salts in molecular solvents, contaminants could be a problem, given the anion concentrations used here (0.3 M to 1.0 M) compared to ionic liquids (2 M to 5 M). But all our XPS data, being element specific, means we can rule out problems with contaminants, as every time we recorded a survey spectrum, we did not observe any contaminants.

13.4. Small calculated uncertainty in our anion descriptors

We use ±0.20 eV as the calculated uncertainty here, based on data in reference [9].

13.5. Possible influence of radiation damage on anion descriptors

Radiation damage will not be a problem for LJ-XPS, given the flowing samples, so sample is constantly refreshed.

Radiation damage is a potentially a big problem for ionic liquids, particularly at synchrotrons where flux can be larger than lab X-ray sources. We have an established methods to overcome radiation damage in neat ionic liquids, employing rastering.[11, 35] As noted in ESI Section 10 to ESI Section 13, we check all core-level spectra very carefully for damage contributions, and any damage contributions in the spectra reported here are minimal.

14. Results. XP spectra used to obtain $DN_{E\text{-}XPS}$

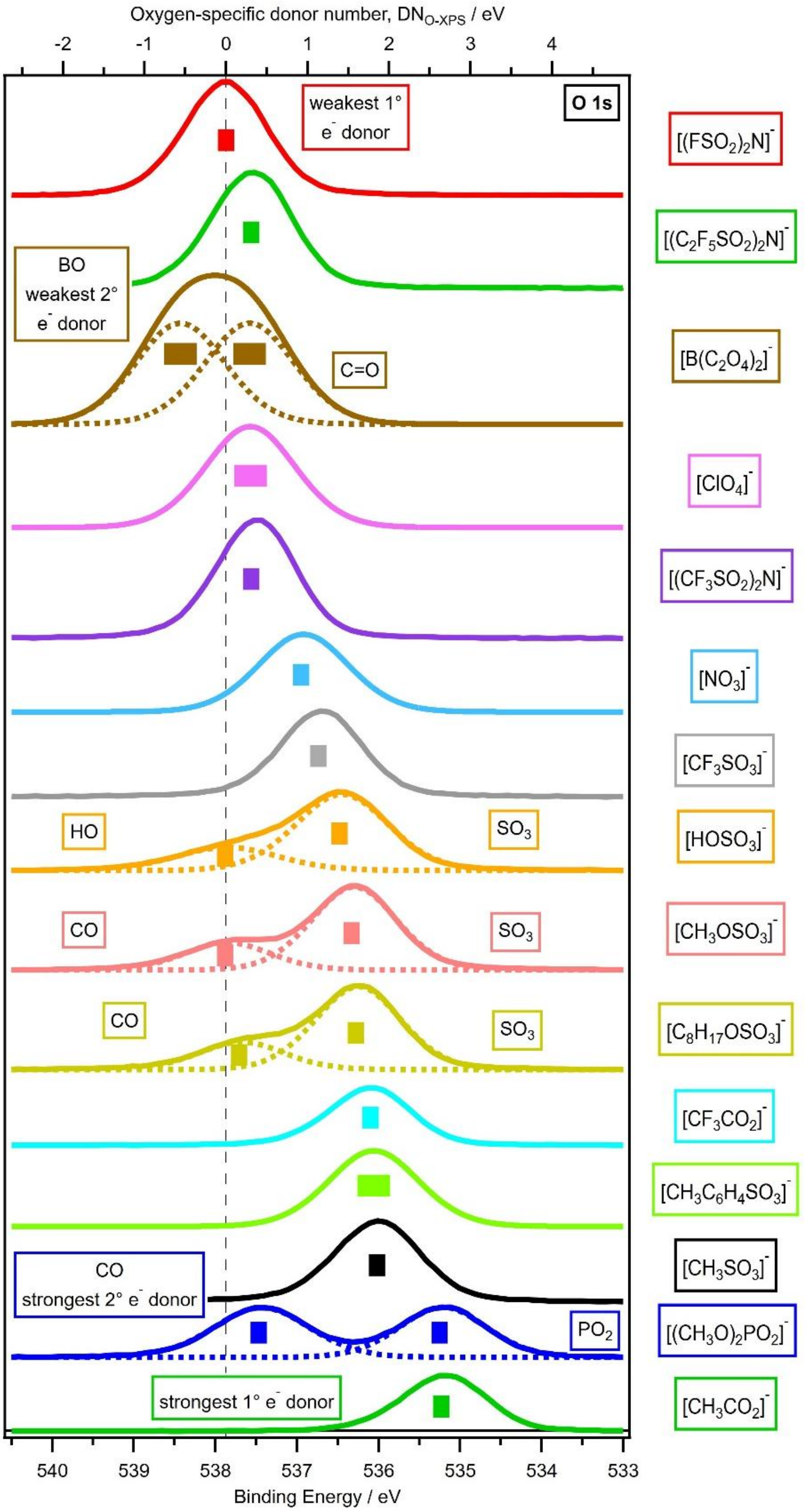

Figure S54. O 1s XP spectra and values of E_B(O_{anion} 1s) and $DN_{O\text{-}XPS}$ (bottom and top axes respectively) of oxygen-containing anions in ionic liquid: $[C_8C_1Im][(FSO_2)_2N]$, $[C_4C_1Im][(C_2F_5SO_2)_2N]$, $[B(C_2O_4)_2]^-$ (calculated, with the BO and C=O contributions plotted separately), $[ClO_4]^-$ (calculated), $[C_8C_1Im][(CF_3SO_2)_2N]$, $[C_8C_1Im][NO_3]$, $[C_8C_1Im][CF_3SO_3]$, $[C_8C_1Im][HOSO_3]$ (total, and also HO and SO_3 fitted components plotted separately), $[C_4C_1Im][CH_3OSO_3]$ (total, and also CO and SO_3 fitted components plotted separately), $[C_4C_1Im][C_8H_{17}OSO_3]$ (total, and also CO and SO_3 fitted components plotted separately), $[C_8C_1Im][CF_3CO_2]$, $[C_2C_1Im][CH_3C_6H_4SO_3]$ (calculated), $[C_2C_1Im][CH_3SO_3]$, $[C_4C_1Im][(CH_3O)_2PO_2]$ (total, and also CO and PO_2 fitted components plotted separately), $[C_4C_1Im][CH_3CO_2]$. Traces and E_B values are vertically offset for clarity. Area normalization is to the number of atoms in each anion, and explained further in ESI Section 8.3. Charge referencing given in ESI Section 8.2. For E_B(O_{anion} 1s) (and therefore $DN_{O\text{-}XPS}$), the experimental uncertainty is ±0.10 eV (ESI Section 13.2) and the calculated uncertainty is ±0.20 eV (ESI Section 13.4); these uncertainty values are represented by the width of each block.

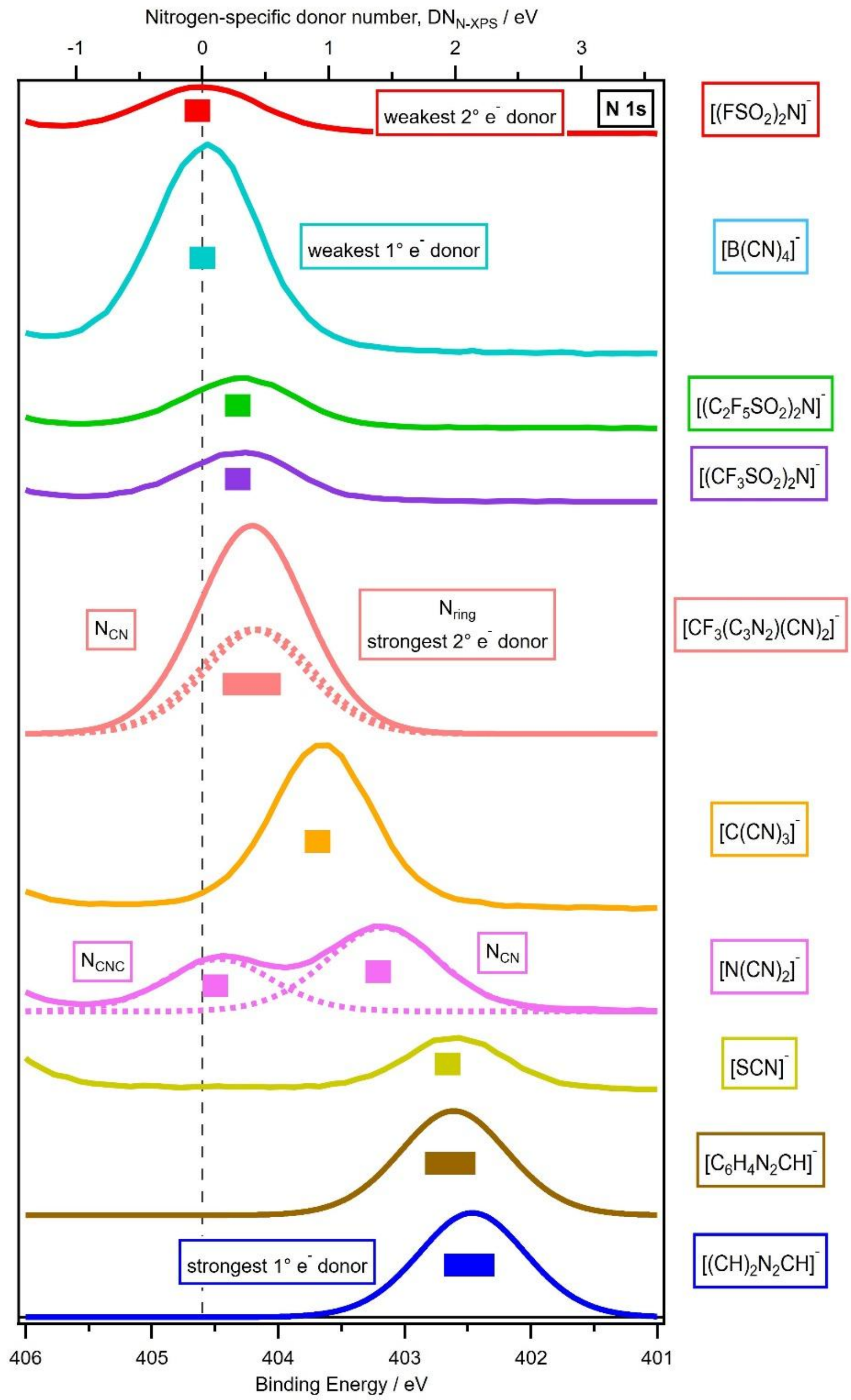

Figure S55. N 1s XP spectra and values of $E_B(N_{anion}\ 1s)$ and $DN_{N\text{-}XPS}$ (bottom and top axes respectively) of nitrogen-containing anions in ionic liquid: $[C_8C_1Im][(FSO_2)_2N]$, $[C_6C_1Im][B(CN)_4]$, $[C_4C_1Im][(C_2F_5SO_2)_2N]$, $[C_8C_1Im][(CF_3SO_2)_2N]$, $[CF_3(C_3N_2)(CN)_2]^-$ (calculated, with the N_{CN} and N_{ring} contributions plotted separately), $[C_8C_1Im][C(CN)_3]$, $[C_4C_1Im][N(CN)_2]$ (total, and also N_{CNC} and N_{CN} fitted components plotted separately), $[C_8C_1Im][SCN]$, $[C_6H_4N_2CH]^-$ (calculated), $[(CH)_2N_2CH]^-$ (calculated). Only the anion fitted components are included; the cation fitted components are not included for clarity. Note that $[C_8C_1Im][NO_3]$ is not included as the nitrogen on $[NO_3]^-$ is very unlikely to be a donor nitrogen atom. Traces and E_B values are vertically offset for clarity. Area normalization is to the number of atoms in each anion, and explained further in ESI Section 8.3. Charge referencing given in ESI Section 8.2. For $E_B(N_{anion}\ 1s)$ (and therefore $DN_{N\text{-}XPS}$), the experimental uncertainty is ±0.10 eV (ESI Section 13.2) and the calculated uncertainty is ±0.20 eV (ESI Section 13.4); these uncertainty values are represented by the width of each block.

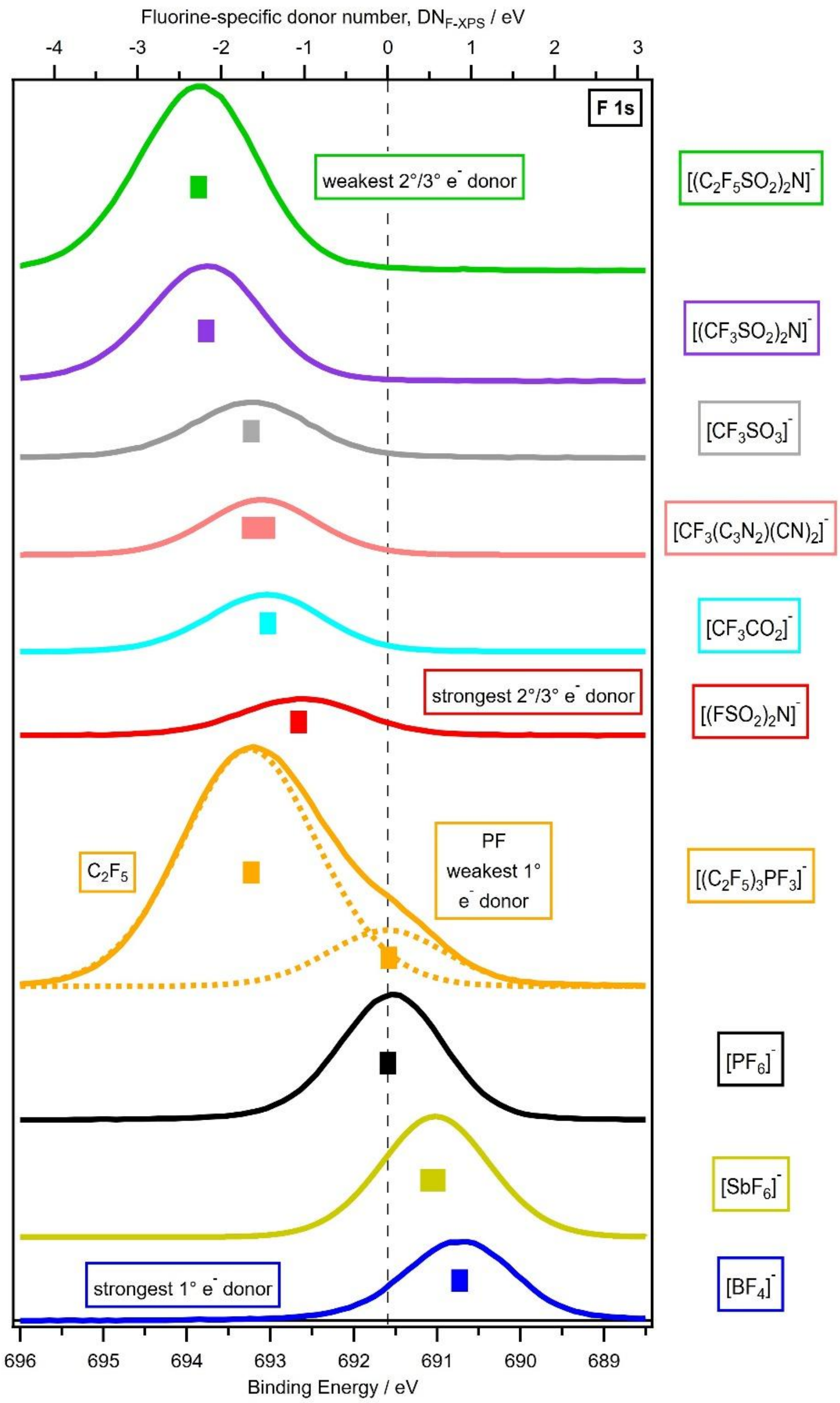

Figure S56. F 1s XP spectra and values of E_B(F$_{anion}$ 1s) and DN$_{F-XPS}$ (bottom and top axes respectively) of fluorine-containing anions in ionic liquid: [C$_4$C$_1$Im][(C$_2$F$_5$SO$_2$)$_2$N], [C$_8$C$_1$Im][(CF$_3$SO$_2$)$_2$N], [C$_8$C$_1$Im][CF$_3$SO$_3$], [CF$_3$(C$_3$N$_2$)(CN)$_2$]$^-$ (calculated), [C$_8$C$_1$Im][CF$_3$CO$_2$], [C$_8$C$_1$Im][(FSO$_2$)$_2$N], [C$_2$C$_1$Im][(C$_2$F$_5$)$_3$PF$_3$], [C$_8$C$_1$Im][PF$_6$], [C$_8$C$_1$Im][SbF$_6$], [C$_8$C$_1$Im][BF$_4$]. Traces and E_B values are vertically offset for clarity. Area normalization is to the number of atoms in each anion, and explained further in ESI Section 8.3. Charge referencing given in ESI Section 8.2. For E_B(F$_{anion}$ 1s) (and therefore DN$_{F-XPS}$), the experimental uncertainty is ±0.10 eV apart from [SbF$_6$]$^-$ (±0.15 eV) (ESI Section 13.2) and the calculated uncertainty is ±0.20 eV (ESI Section 13.4); these uncertainty values are represented by the width of each block.

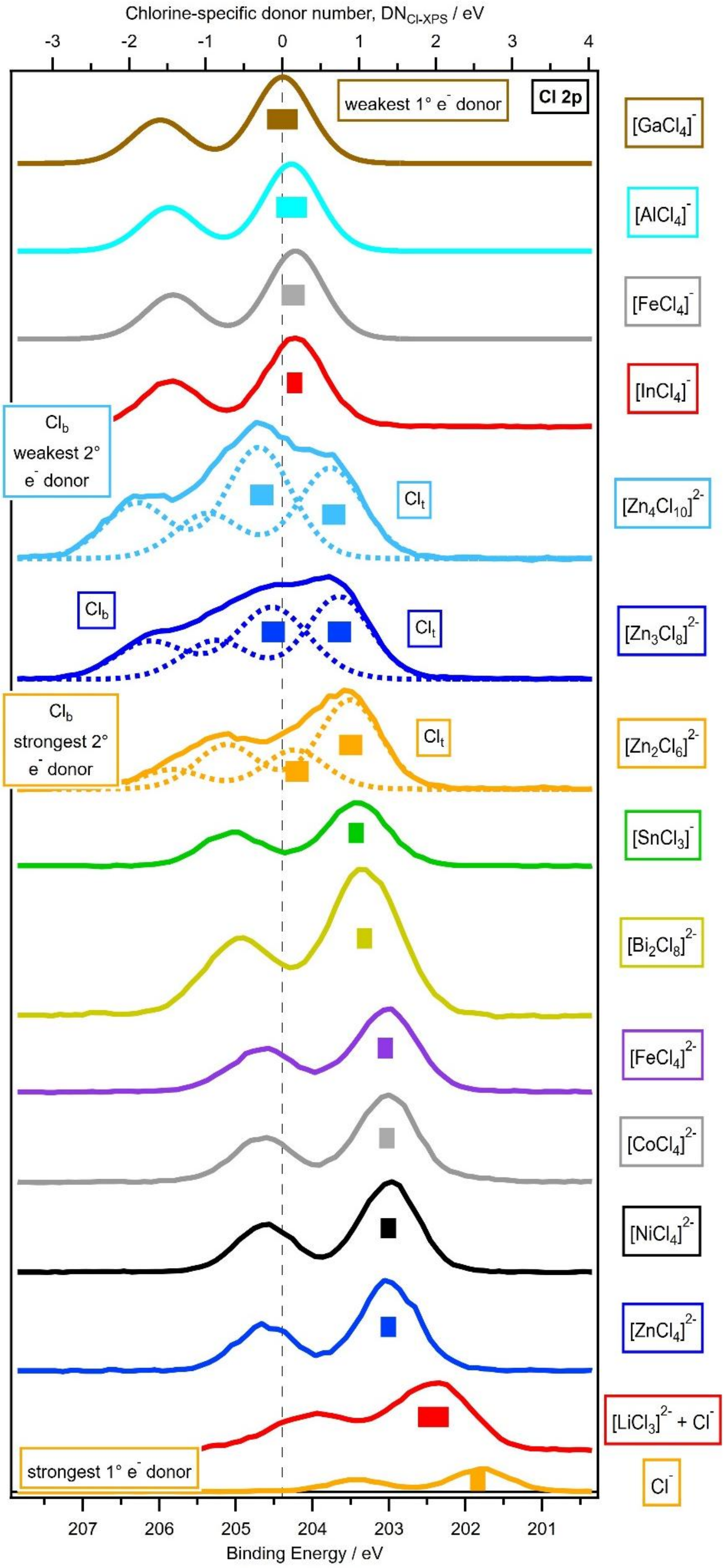

Figure S57. Cl 2p XP spectra and values of E_B(Cl$_{anion}$ 2p$_{3/2}$) and DN$_{Cl-XPS}$ (bottom and top axes respectively) of chlorine-containing anions in ionic liquid: $[GaCl_4]^-$ (calculated), $AlCl_4]^-$ (calculated), $[C_8C_1Im][FeCl_4]$ (produced from E_B(Cl$_{anion}$ 2p$_{3/2}$) given in reference [16]), $[C_8C_1Im][InCl_4]$, $[C_8C_1Im]_2[Zn_4Cl_{10}]$, $[C_8C_1Im]_2[Zn_3Cl_8]$, $[C_8C_1Im]_2[Zn_2Cl_6]$, $[C_8C_1Im][SnCl_3]$, $[C_8C_1Im]_2[Bi_2Cl_8]$, $[C_8C_1Im]_2[FeCl_4]$, $[C_8C_1Im]_2[CoCl_4]$, $[C_8C_1Im]_2[NiCl_4]$, $[C_8C_1Im]_2[ZnCl_4]$, $[C_8C_1Im]_{11}[LiCl_3]Cl[(CF_3SO_2)_2N]_8$, $[C_8C_1Im]Cl$. Traces and E_B values are vertically offset for clarity. Area normalization is to the number of atoms in each anion, and explained further in ESI Section 8.3. Charge referencing given in ESI Section 8.2. For E_B(Cl$_{anion}$ 2p$_{3/2}$) (and therefore DN$_{Cl-XPS}$), the experimental uncertainty is ±0.10 eV apart from $[LiCl_3]^{2-}$ (±0.20 eV), $[FeCl_4]^-$ (±0.15 eV), oligomeric chlorozincate anions (±0.15 eV) (ESI Section 13.2) and the calculated uncertainty is ±0.20 eV (ESI Section 13.4); these uncertainty values are represented by the width of each block.

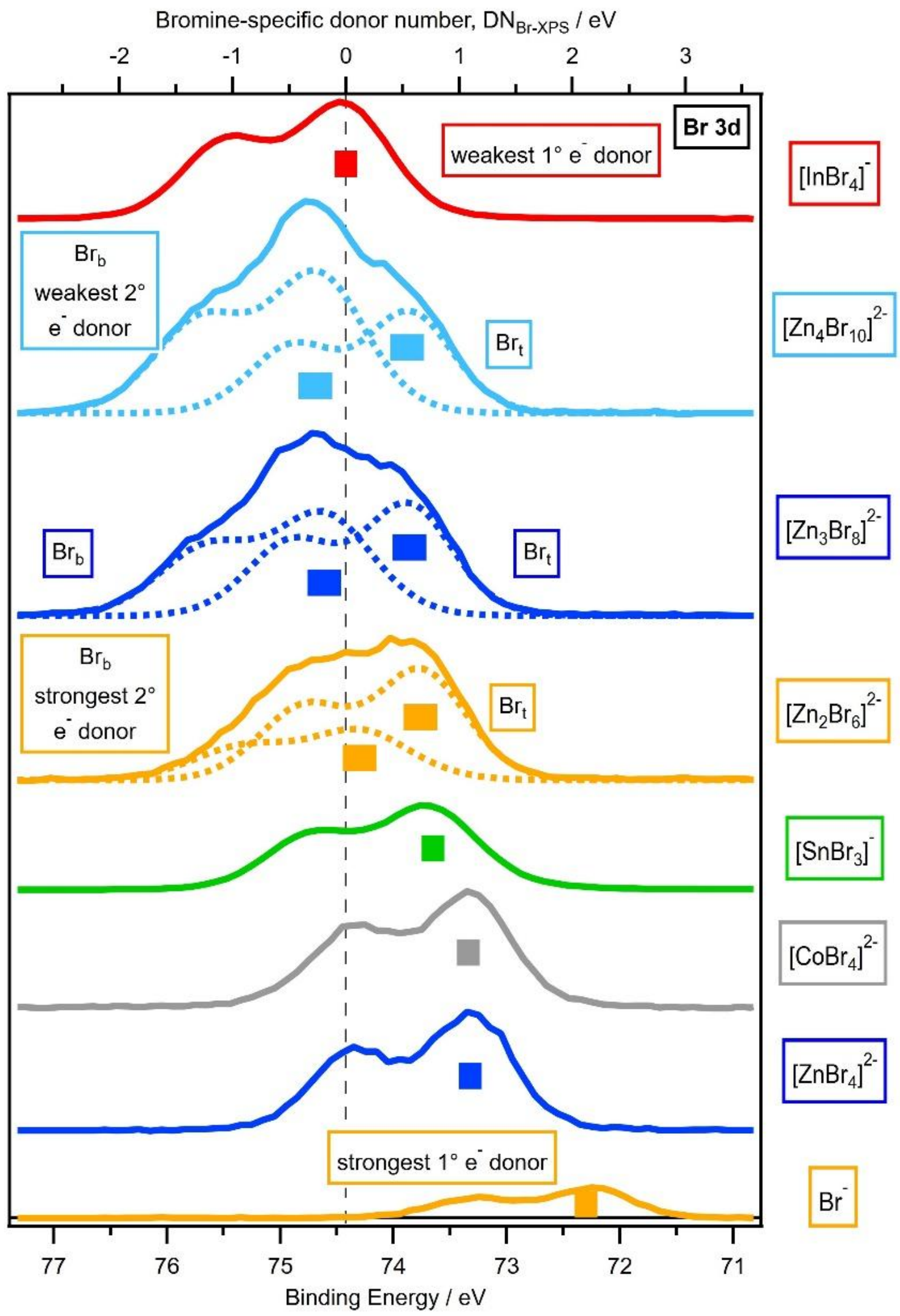

Figure S58. Br 3d XP spectra and values of E_B(Br_{anion} $3d_{5/2}$) and $DN_{Br\text{-}XPS}$ (bottom and top axes respectively) of bromine-containing anions in ionic liquid: $[C_8C_1Im][InBr_4]$, $[C_8C_1Im]_2[Zn_4Br_{10}]$, $[C_8C_1Im]_2[Zn_3Br_8]$, $[C_8C_1Im]_2[Zn_2Br_6]$, $[C_8C_1Im][SnBr_3]$, $[C_8C_1Im]_2[CoBr_4]$, $[C_8C_1Im]_2[ZnBr_4]$, $[C_8C_1Im]Br$. Traces and E_B values are vertically offset for clarity. Area normalization is to the number of atoms in each anion, and explained further in ESI Section 8.3. Charge referencing given in ESI Section 8.2. For E_B(Br_{anion} $3d_{5/2}$) (and therefore $DN_{Br\text{-}XPS}$), the experimental uncertainty is ±0.10 eV apart from oligomeric bromozincate anions (±0.15 eV) (ESI Section 13.2); these uncertainty values are represented by the width of each block.

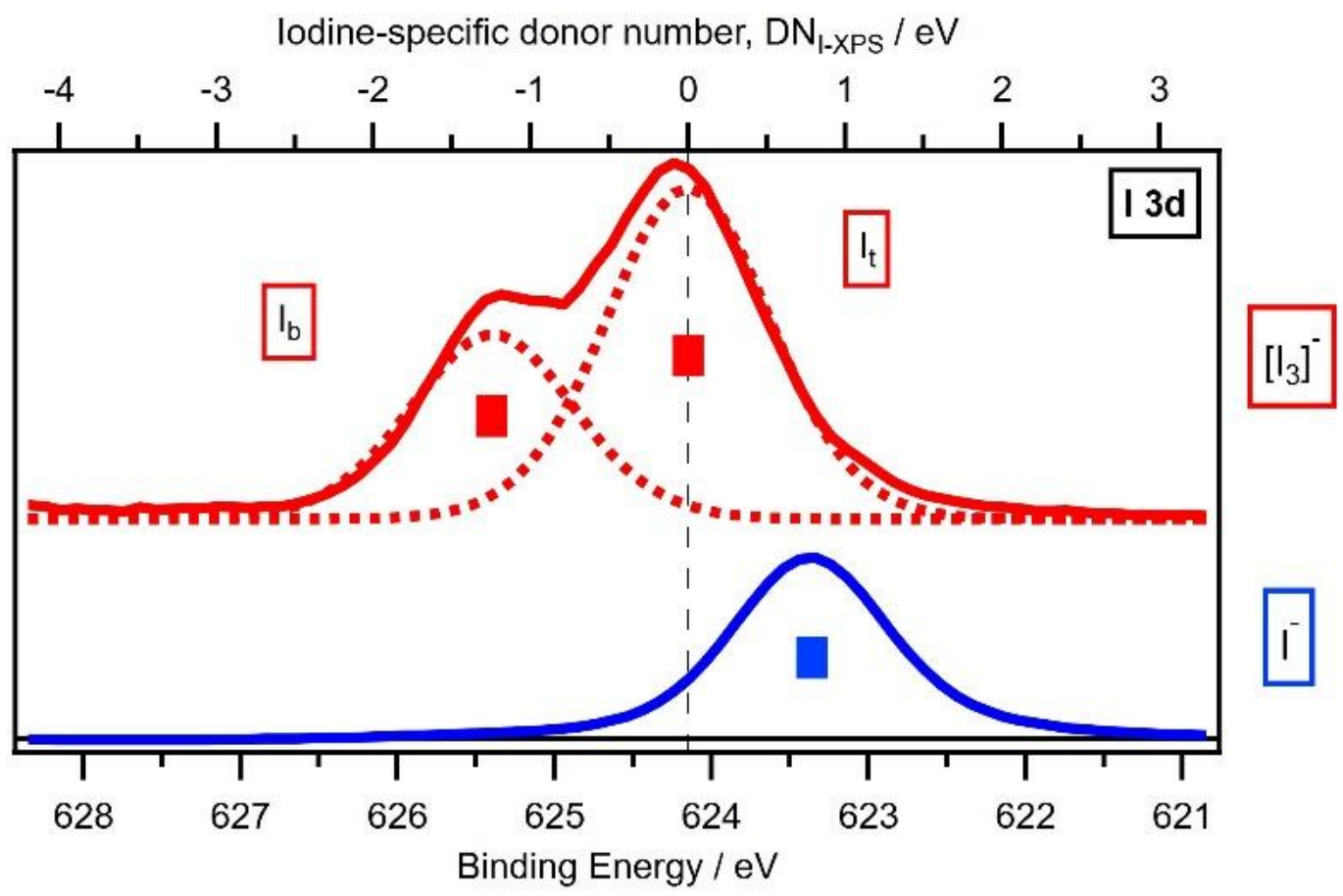

Figure S59. I 3d XP spectra and values of E_B(I_{anion} $3d_{5/2}$) and $DN_{I\text{-}XPS}$ (bottom and top axes respectively) of iodine-containing anions in ionic liquid: $[C_8C_1Im][I_3]$, $[C_6C_1Im]I$. Traces and E_B values are vertically offset for clarity. Area normalization is to the number of atoms in each anion, and explained further in ESI Section 8.3. Charge referencing given in ESI Section 8.2. For E_B(I_{anion} $3d_{5/2}$) (and therefore $DN_{I\text{-}XPS}$), the experimental uncertainty is ±0.10 eV (ESI Section 13.2); these uncertainty values are represented by the width of each block.

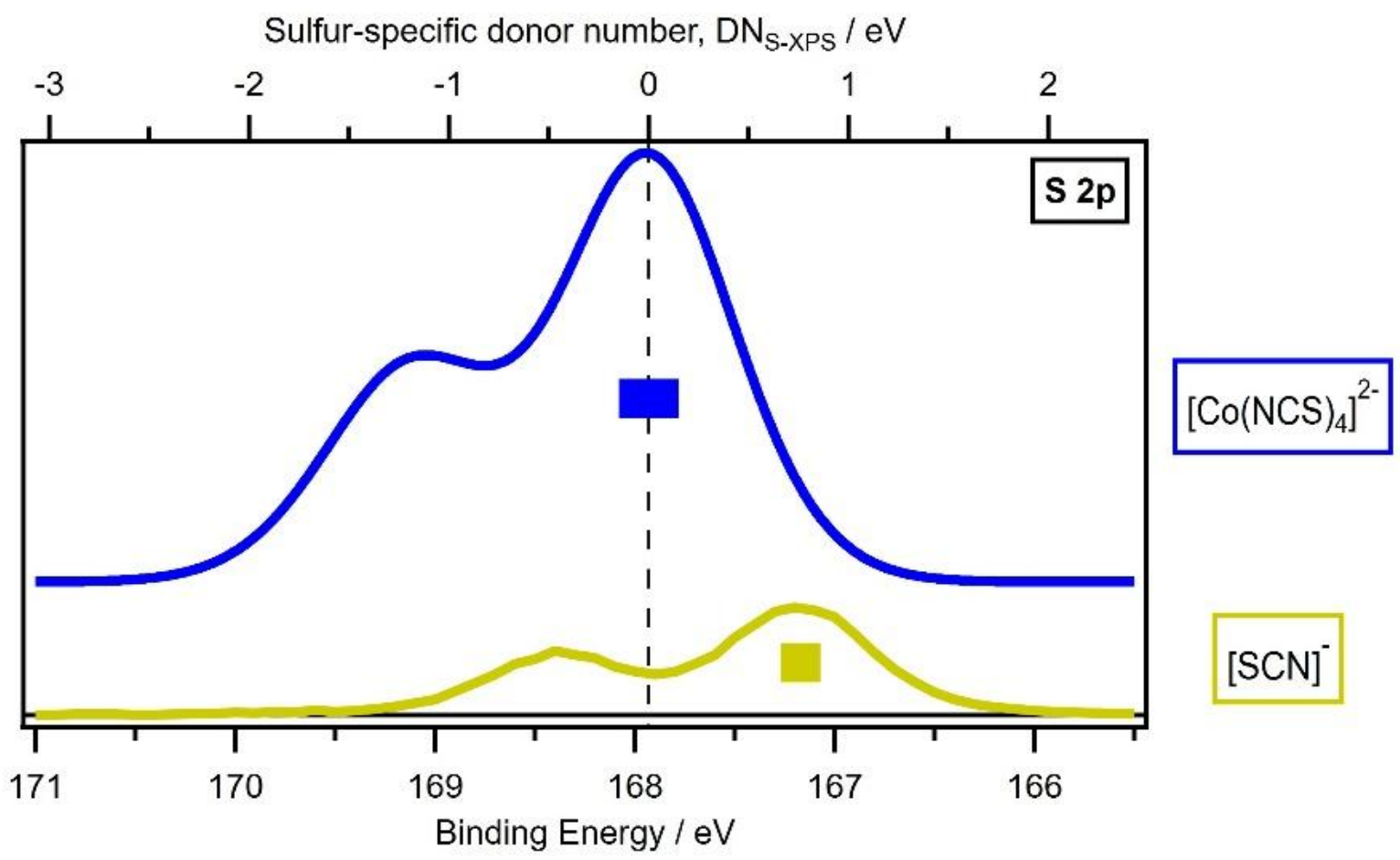

Figure S60. S 2p XP spectra and values of E_B(S_{anion} $2p_{3/2}$) and $DN_{S\text{-}XPS}$ (bottom and top axes respectively) of sulfur-containing anions in ionic liquid: $[C_8C_1Im]_2[Co(NCS)_4]$, $[C_8C_1Im][SCN]$. Traces and E_B values are vertically offset for clarity. Area normalization is to the number of atoms in each anion, and explained further in ESI Section 8.3. Charge referencing given in ESI Section 8.2. For E_B(S_{anion} $2p_{3/2}$) (and therefore $DN_{S\text{-}XPS}$), the experimental uncertainty is ±0.10 eV for $[SCN]^-$ and ±0.15 eV for $[Co(NCS)_4]^{2-}$ (ESI Section 13.2); these uncertainty values are represented by the width of each block.

15. Results. E_B(core,anion) for ionic liquids with more than one sample measured

Table S22. E_B(core,anion) for the ionic liquid $[C_8C_1Im][CF_3CO_2]$

Apparatus	$E_B(F_{anion}$ 1s) / eV	$E_B(O_{anion}$ 1s) / eV
UoR	693.09	536.14
B07	692.97	536.06
Average	693.03	536.10
Standard deviation	0.08	0.06
Standard error	0.06	0.04

Table S23. E_B(core,anion) for the ionic liquid $[C_8C_1Im][(CF_3SO_2)_2N]$

Apparatus	$E_B(F_{anion}$ 1s) / eV	$E_B(O_{anion}$ 1s) / eV	$E_B(N_{anion}$ 1s) / eV
UoR	693.81	537.60	404.36
UCL (Kα)	693.71	537.51	404.30
B07 dataset 2	693.76	537.52	404.32
UCL (TP)	693.73	537.52	404.28
B07 dataset 1	693.78	537.54	404.32
Notts (Ag source)	693.86	537.63	404.35
Average	693.77	537.56	404.32
Standard deviation	0.05	0.05	0.03
Standard error	0.02	0.02	0.01

Table S24. E_B(core,anion) for the ionic liquid $[C_4C_1Im][(C_2F_5SO_2)_2N]$

Apparatus	$E_B(F_{anion}$ 1s) / eV	$E_B(O_{anion}$ 1s) / eV	$E_B(N_{anion}$ 1s) / eV
UoR	693.90	537.59	404.34
B07	693.82	537.54	404.31
Average	693.86	537.56	404.32
Standard deviation	0.06	0.03	0.02
Standard error	0.04	0.02	0.02

Table S25. E_B(core,anion) for the ionic liquid $[C_8C_1Im][SCN]$

Apparatus	$E_B(N_{anion}$ 1s) / eV	$E_B(S_{anion}$ $2p_{3/2}$) / eV
Imperial (Kα)	402.64	167.18
UoR	402.68	167.16
Average	402.66	167.17
Standard deviation	0.03	0.01
Standard error	0.02	0.01

Table S26. E_B(core,anion) for the ionic liquid $[C_2C_1Im][(C_2F_5)_3PF_3]$

Apparatus	$E_B(F_{anion,PF}$ 1s) / eV	$E_B(F_{anion,CF}$ 1s) / eV
B07	691.53	693.18
UoR	691.64	693.28
Average	691.58	693.23
Standard deviation	0.08	0.07
Standard error	0.06	0.05

Table S27. E_B(core,anion) for the ionic liquid $[C_8C_1Im]Cl$

Apparatus	$E_B(Cl_{anion}$ $2p_{3/2}$) / eV
UCL (TP)	201.81
B07	201.86
Average	201.84
Standard deviation	0.04
Standard error	0.03

Table S28. E_B(core,anion) for the ionic liquid $[C_8C_1Im]Br$

Apparatus	$E_B(Br_{anion}\ 3d_{3/2})$ / eV
UCL (TP)	72.25
B07	72.36
Average	72.30
Standard deviation	0.07
Standard error	0.05

16. Results. E_B(core,anion) for water with more than one sample measured

Table S29. E_B(core,anion) for [cation][SCN] in water

Countercation	Apparatus	$E_B(N_{anion}$ 1s) / eV
$[C_4C_1Im]^+$	SOL3PES	403.07
K^+	SOL3PES	402.96
	Average	403.02
	Standard deviation	0.07
	Standard error	0.05

Table S30. E_B(core,anion) for [cation][CF_3SO_3] in water

Countercation	Apparatus	$E_B(F_{anion}$ 1s) / eV
Na^+	HIPPIE	693.67
Zn^{2+}	HIPPIE	693.65
Mg^{2+}	HIPPIE	693.63
Ca^{2+}	HIPPIE	693.70
K^+	GALAXIES ($h\nu$ = 2300 eV)	693.64
K^+	GALAXIES ($h\nu$ = 4000 eV)	693.54
$[C_4C_1Im]^+$	GALAXIES	693.67
	Average	693.64
	Standard deviation	0.05
	Standard error	0.02

Table S31. E_B(core,anion) for Li[$(CF_3SO_2)_2N$] in water

Countercation	Apparatus	$E_B(F_{anion}$ 1s) / eV	$E_B(N_{anion}$ 1s) / eV
Li^+	HIPPIE	693.78	404.47
Li^+	SOL3PES	693.70	404.35
Li^+	GALAXIES	693.79	404.51
	Average	693.76	404.44
	Standard deviation	0.05	0.09
	Standard error	0.03	0.05

17. Results. E_B(core,anion) for PC with more than one sample measured

Table S32. E_B(core,anion) for [cation][SCN] in PC

Countercation	Apparatus	$E_B(N_{anion}$ 1s) / eV
K^+	SOL3PES	402.53
$[C_4C_1Im]^+$	GALAXIES	402.56
$[C_4C_1Im]^+$	SOL3PES	402.52
	Average	402.54
	Standard deviation	0.02
	Standard error	0.01

Table S33. E_B(core,anion) for [cation][$(CF_3SO_2)_2N$] in PC

Countercation	Apparatus	$E_B(F_{anion}$ 1s) / eV	$E_B(N_{anion}$ 1s) / eV
Na^+	HIPPIE	693.92	404.36
Li^+	SOL3PES	693.89	404.26
Li^+	GALAXIES	693.78	404.32
	Average	693.86	404.31
	Standard deviation	0.08	0.05
	Standard error	0.04	0.03

18. Results. E_B(core,anion) for TEP with more than one sample measured

Table S34. E_B(core,anion) for [cation][$(CF_3SO_2)_2N$] in TEP

Countercation	Apparatus	$E_B(F_{anion}$ 1s) / eV	$E_B(N_{anion}$ 1s) / eV
Li^+	HIPPIE	693.82	404.16
Na^+	HIPPIE	693.81	404.20
	Average	693.82	404.18
	Standard deviation	0.00	0.03
	Standard error	0.00	0.02

19. Results. Calculated E_B(core,anion) using ionic liquid SMD parameters

$DN_{O\text{-}XPS}$ = 520.90 – uncorrected calculated $E_B(O_{anion}\ 1s)$ Equation S2
$DN_{N\text{-}XPS}$ = 389.99 – uncorrected calculated $E_B(N_{anion}\ 1s)$ Equation S3
$DN_{F\text{-}XPS}$ = 670.90 – uncorrected calculated $E_B(F_{anion}\ 1s)$ Equation S4
$DN_{Cl\text{-}XPS}$ = 194.36 – uncorrected calculated $E_B(Cl_{anion}\ 2p_{3/2})$ Equation S5

Table S35. Calculated $E_B(O_{anion}\ 1s)$ using ionic liquid SMD parameters

Anion	Moiety	Calculated $-E_B(O_{anion}\ 1s)$ / eV	Average calculated $E_B(O_{anion}\ 1s)$ / eV	Average calculated $E_B(O_{anion}\ 1s)$ after E_B correction of +16.97 eV applied (to allow direct comparison to experimental $E_B(O_{anion}\ 1s)$) / eV
$[B(C_2O_4)_2]^-$	B-O	521.46<br>521.46<br>521.46<br>521.46	521.46	538.43
$[B(C_2O_4)_2]^-$	C=O	520.61<br>520.61<br>520.61<br>520.61	520.61	537.58
$[ClO_4]^-$	Cl-O	520.60<br>520.60<br>520.60<br>520.60	520.60	537.57
$[CH_3C_6H_4SO_3]^-$	S-O	519.10<br>519.09<br>519.07	519.09	536.06

Table S36. Calculated $E_B(N_{anion}\ 1s)$ using ionic liquid SMD parameters

Anion	Moiety	Calculated $-E_B(N_{anion}\ 1s)$ / eV	Average calculated $E_B(N_{anion}\ 1s)$ / eV	Average calculated $E_B(N_{anion}\ 1s)$ after E_B correction of +14.61 eV applied (to allow direct comparison to experimental $E_B(N_{anion}\ 1s)$) / eV
$[(CH)_2N_2CH]^-$	N_{ring}	387.88<br>387.88	387.88	402.49
$[C_6H_4N_2CH]^-$	N_{ring}	388.03<br>388.03	388.03	402.64
$[CF_3(C_3N_2)(CN)_2]^-$	N_{ring}	389.57<br>389.56	389.57	404.18
$[CF_3(C_3N_2)(CN)_2]^-$	N_{CN}	389.63<br>389.62	389.63	404.24

Table S37. Calculated $E_B(F_{anion}\ 1s)$ using ionic liquid SMD parameters

Anion	Moiety	Calculated $-E_B(F_{anion}\ 1s)$ / eV	Average calculated $E_B(F_{anion}\ 1s)$ / eV	Average calculated $E_B(F_{anion}\ 1s)$ after E_B correction of +20.69 eV applied (to allow direct comparison to experimental $E_B(F_{anion}\ 1s)$) / eV
$[CF_3(C_3N_2)(CN)_2]^-$	N_{CN}	672.50<br>672.48<br>672.36	672.45	693.14

Table S38. Calculated E_B(Cl_{anion} $2p_{3/2}$) using ionic liquid SMD parameters

Anion	Moiety	Calculated -E_B(Cl_{anion} $2p_{3/2}$) / eV	Average calculated E_B(Cl_{anion} $2p_{3/2}$) / eV	Average calculated E_B(Cl_{anion} $2p_{3/2}$) after E_B correction of +10.03 eV applied (to allow direct comparison to experimental E_B(Cl_{anion} $2p_{3/2}$)) / eV
$[AlCl_4]^-$	AlCl	194.32<br>194.32<br>194.31<br>194.31<br>194.21<br>194.21<br>194.21<br>194.21<br>194.21<br>194.21<br>194.21<br>194.21	194.24	204.27
$[GaCl_4]^-$	GaCl	194.45<br>194.45<br>194.45<br>194.45<br>194.31<br>194.31<br>194.31<br>194.31<br>194.31<br>194.31<br>194.31<br>194.31	194.36	204.39

20. Results. Calculated E_B(core,anion) using water SMD parameters

Table S39. Experimental and calculated E_B(N_{anion} 1s) for anions in water

Anion	Moiety	Calculated $-E_B$(N_{anion} 1s) / eV	Average calculated E_B(N_{anion} 1s) / eV	Experimental E_B(N_{anion} 1s) / eV	ΔE_B(N_{anion} 1s) / eV
$[(CF_3SO_2)_2N]^-$	S-N-S	390.38	390.38	404.44	14.06
$[SCN]^-$	C≡N	388.42	388.42	403.02	14.60
$[N(CN)_2]^-$	C-N-C	389.91	389.91	404.76	14.85
$[N(CN)_2]^-$	C≡N	388.76<br>388.76	388.76	403.50	14.74
$[(FSO_2)_2N]^-$	S-N-S	390.74	390.74	404.73	13.99
				average	14.45

Table S40. Experimental and calculated E_B(F_{anion} 1s) for anions in water

Anion	Moiety	Calculated $-E_B$(F_{anion} 1s) / eV	Average calculated E_B(F_{anion} 1s) / eV	Experimental E_B(F_{anion} 1s) / eV	ΔE_B(F_{anion} 1s) / eV
$[PF_6]^-$	PF	671.28<br>671.28<br>671.28<br>671.28<br>671.28<br>671.28	671.28	691.83	20.55
$[BF_4]^-$	BF	670.31<br>670.31<br>670.31<br>670.31	670.31	691.18	20.87
$[(CF_3SO_2)_2N]^-$	CF_3	673.45<br>673.45<br>673.44<br>673.44<br>673.43<br>673.43	673.44	693.76	20.32
$[CF_3SO_3]^-$	CF_3	673.14<br>673.14<br>673.14	673.14	693.64	20.50
$[(FSO_2)_2N]^-$	F	672.45<br>672.45	672.45	692.88	20.43
$[CF_3CO_2]^-$	CF_3	672.61<br>672.60<br>672.60	672.60		
				average	20.53

Table S41. Calculated E_B(O_{anoin} 1s) for anions in water. Estimated E_B correction of +16.81 eV taken from Table S51

Anion	Moiety	Calculated $-E_B$(O_{anion} 1s) / eV	Average calculated E_B(O_{anion} 1s) / eV	E_B(O_{anion} 1s) with estimated E_B correction of +16.81 eV included / eV
$[(CF_3SO_2)_2N]^-$	SO	521.09<br>521.09<br>521.05<br>521.05	521.07	537.88
$[CF_3SO_3]^-$	SO	520.39<br>520.39<br>520.39	520.39	537.20
$[(FSO_2)_2N]^-$	SO	521.43<br>521.43<br>521.41<br>521.41	521.42	538.23
$[CH_3CO_2]^-$	CO_2	519.00<br>518.97	518.98	535.79
$[CF_3CO_2]^-$	CO_2	519.72<br>519.68	519.70	536.51

21. Results. Calculated E_B(core,anion) using PC SMD parameters

Table S42. Experimental and calculated $E_B(N_{anion}$ 1s) for anions in PC

Anion	Moiety	Calculated -$E_B(N_{anion}$ 1s) / eV	Average calculated $E_B(N_{anion}$ 1s) / eV	Experimental $E_B(N_{anion}$ 1s) / eV	$\Delta E_B(N_{anion}$ 1s) / eV
$[(CF_3SO_2)_2N]^-$	S-N-S	389.99	389.99	404.31	14.32
$[SCN]^-$	C≡N	388.40	388.40	402.54	14.14
$[(FSO_2)_2N]^-$	S-N-S	390.41	390.41	404.61	14.20
				average	14.22

Table S43. Experimental and calculated $E_B(F_{anion}$ 1s) for anions in PC

Anion	Moiety	Calculated -$E_B(F_{anion}$ 1s) / eV	Average calculated $E_B(F_{anion}$ 1s) / eV	Experimental $E_B(F_{anion}$ 1s) / eV	$\Delta E_B(F_{anion}$ 1s) / eV
$[PF_6]^-$	PF	671.27<br>671.27<br>671.27<br>671.27<br>671.27<br>671.27	671.27	691.60	20.33
$[(CF_3SO_2)_2N]^-$	CF_3	673.24<br>673.24<br>673.23<br>673.23<br>673.23<br>673.23	673.23	693.86	20.63
$[CF_3SO_3]^-$	CF_3	672.84<br>672.84<br>672.84	672.84	693.54	20.70
$[(FSO_2)_2N]^-$	F	672.05<br>672.05	672.05	692.69	20.64
				average	20.58

Table S44. Calculated $E_B(O_{anoin}$ 1s) for anions in PC

Anion	Moiety	Calculated -$E_B(O_{anion}$ 1s) / eV	Average calculated $E_B(O_{anion}$ 1s) / eV
$[(CF_3SO_2)_2N]^-$	SO	520.66<br>520.66<br>520.61<br>520.61	520.63
$[CF_3SO_3]^-$	SO	519.72<br>519.72<br>519.72	519.72
$[(FSO_2)_2N]^-$	SO	521.06<br>521.06<br>521.02<br>521.02	521.04

22. Results. Calculated E_B(core,anion) using TEP SMD parameters

Table S45. Experimental and calculated $E_B(N_{anion}$ 1s) for anions in TEP

Anion	Moiety	Calculated $-E_B(N_{anion}$ 1s) / eV	Average calculated $E_B(N_{anion}$ 1s) / eV	Experimental $E_B(N_{anion}$ 1s) / eV	$\Delta E_B(N_{anion}$ 1s) / eV
$[(CF_3SO_2)_2N]^-$	S-N-S	389.76	389.76	404.18	14.42
$[SCN]^-$	C≡N	388.09	388.09	402.38	14.29
$[(FSO_2)_2N]^-$	S-N-S	390.17	390.17	404.44	14.27
				average	14.33

Table S46. Experimental and calculated $E_B(F_{anion}$ 1s) for anions in TEP

Anion	Moiety	Calculated $-E_B(F_{anion}$ 1s) / eV	Average calculated $E_B(F_{anion}$ 1s) / eV	Experimental $E_B(F_{anion}$ 1s) / eV	$\Delta E_B(F_{anion}$ 1s) / eV
$[PF_6]^-$	PF	670.98<br>670.98<br>670.98<br>670.98<br>670.98<br>670.98	670.98	691.33	20.35
$[(CF_3SO_2)_2N]^-$	CF_3	673.04<br>673.04<br>673.04<br>673.04<br>673.03<br>673.03	673.04	693.82	20.78
$[CF_3SO_3]^-$	CF_3	672.60<br>672.60<br>672.60	672.60		
$[(FSO_2)_2N]^-$	F	671.81<br>671.81	671.81	692.51	20.70
				average	20.61

Table S47. Calculated $E_B(O_{anoin}$ 1s).

Anion	Moiety	Calculated $-E_B(O_{anion}$ 1s) / eV	Average calculated $E_B(O_{anion}$ 1s) / eV
$[(CF_3SO_2)_2N]^-$	SO	520.44<br>520.44<br>520.39<br>520.39	520.41
$[CF_3SO_3]^-$	SO	519.46<br>519.46<br>519.46	519.46
$[(FSO_2)_2N]^-$	SO	520.82<br>520.82<br>520.78<br>520.78	520.80

23. Results. E_B(core,anion) correction for lone-anion molecular solvent SMD calculations

Table S48. Average E_B(correction) for each core-level for lone-anion water SMD calculations

Core-level	Average E_B(correction) from lone-anion-SMD effectively charge referenced to vacuum level / eV	Standard error of the sample mean / eV
F 1s	20.53	0.09
N 1s	14.45	0.18

Table S49. Average E_B(correction) for each core-level for lone-anion PC SMD calculations

Core-level	Average E_B(correction) from lone-anion-SMD effectively charge referenced to vacuum level / eV	Standard error of the sample mean / eV
F 1s	20.58	0.08
N 1s	14.22	0.05

Table S50. Average E_B(correction) for each core-level for lone-anion TEP SMD calculations

Core-level	Average E_B(correction) from lone-anion-SMD effectively charge referenced to vacuum level / eV	Standard error of the sample mean / eV
F 1s	20.61	0.13
N 1s	14.33	0.05

Table S51. Average E_B(correction) for each core-level for lone-anion SMD calculations. Values for O 1s and Cl $2p_{3/2}$ are estimated

Core-level	Average E_B(correction) from lone-anion ionic liquid SMD effectively charge referenced to vacuum level / eV	Average E_B(correction) from lone-anion water SMD effectively charge referenced to vacuum level / eV	Average E_B(correction) from lone-anion PC SMD effectively charge referenced to vacuum level / eV	Average E_B(correction) from lone-anion TEP SMD effectively charge referenced to vacuum level / eV
F 1s	20.69	20.53	20.58	20.61
O 1s	16.97	*16.81*		
N 1s	14.61	14.45	14.22	14.33
Cl $2p_{3/2}$	10.03	*9.87*		

24. Results. E_B(core,anion) for ionic liquids used to obtain $DN_{E\text{-}XPS}$

Table S52. Experimental $E_B(Br_{anion}\ 3d_{5/2})$ and bromine-specific donor numbers ($DN_{Br\text{-}XPS}$) for ionic liquid samples. Experimental uncertainties are ±0.10 eV for experimental $E_B(Br_{anion}\ 3d_{5/2})$ (apart from the oligomeric bromozincate anions with ±0.15 eV) (ESI Section 14.2)

Anion no.	Anion	Moiety	1°/2°/3°	$E_B(Br_{anion}\ 3d_{5/2})$ / eV	$DN_{Br\text{-}XPS}$ / eV
42	Br^-	Br	Strongest 1°	72.30 ±0.10	2.12 ±0.10
43	$[ZnBr_4]^{2-}$	Br	1°	73.32 ±0.10	1.10 ±0.10
44	$[CoBr_4]^{2-}$	Br	1°	73.34 ±0.10	1.08 ±0.10
46	$[SnBr_3]^-$	Br	1°	73.65 ±0.10	0.77 ±0.10
47	$[Zn_2Br_6]^{2-}$	$Br_{terminal}$	1°	73.76 ±0.15	0.66 ±0.15
48	$[Zn_3Br_8]^{2-}$	$Br_{terminal}$	1°	73.86 ±0.15	0.56 ±0.15
49	$[Zn_4Br_{10}]^{2-}$	$Br_{terminal}$	1°	73.88 ±0.15	0.54 ±0.15
45	$[InBr_4]^-$	Br	Weakest 1°	74.42 ±0.10	0.00 ±0.10
47	$[Zn_2Br_6]^{2-}$	Br_{bridge}	Strongest 2°	74.30 ±0.15	0.12 ±0.15
48	$[Zn_3Br_8]^{2-}$	Br_{bridge}	2°	74.61 ±0.15	-0.19 ±0.15
49	$[Zn_4Br_{10}]^{2-}$	Br_{bridge}	Weakest 2°	74.69 ±0.15	-0.27 ±0.15

Table S53. Experimental $E_B(I_{anion}\ 3d_{5/2})$ and iodine-specific donor numbers ($DN_{I\text{-}XPS}$) for ionic liquid samples. Experimental uncertainties are ±0.10 eV for experimental $E_B(I_{anion}\ 3d_{5/2})$ (ESI Section 14.2)

Anion no.	Anion	Moiety	1°/2°/3°	$E_B(I_{anion}\ 3d_{5/2})$ / eV	$DN_{I\text{-}XPS}$ / eV
50	I^-	I	Strongest 1°	623.36 ±0.10	0.79 ±0.10
51	$[I_3]^-$	$I_{terminal}$	Weakest 1°	624.15 ±0.10	0.00 ±0.10
51	$[I_3]^-$	I_{bridge}	2°	625.40 ±0.10	-1.25 ±0.10

Table S54. Experimental $E_B(S_{anion}\ 2p_{3/2})$ and sulfur-specific donor numbers ($DN_{S\text{-}XPS}$) for ionic liquid samples. Experimental uncertainties are ±0.10 eV for experimental $E_B(S_{anion}\ 2p_{5/2})$ (ESI Section 14.2)

Anion no.	Anion	Moiety	1°/2°/3°	$E_B(S_{anion}\ 2p_{3/2})$ / eV	$DN_{S\text{-}XPS}$ / eV
52	$[Co(NCS)_4]^{2-}$	S	1°	167.93 ±0.15	0.00 ±0.15
21	$[SCN]^-$	S	2°	167.17 ±0.10	0.76 ±0.10

25. Results. E_B(core,anion) for water

Table S55. Experimental E_B(N_{anion} 1s) for water samples

Anion no.	Anion	Moiety	1°/2°/3°	E_B(N_{anion} 1s) / eV
21	$[SCN]^-$	N	1°	403.02
20	$[N(CN)_2]^-$	$N_{terminal}$	1°	403.50
13	$[(CF_3SO_2)_2N]^-$	N	2°	404.44
14	$[(FSO_2)_2N]^-$	N	2°	404.73
20	$[N(CN)_2]^-$	N_{bridge}	2°	404.76

Table S56. Experimental and calculated E_B(F_{anion} 1s) for water samples (calculated E_B(F_{anion} 1s) in italics, E_B correction of +20.53 eV taken from Table S51)

Anion no.	Anion	Moiety	1°/2°/3°	E_B(F_{anion} 1s) / eV
23	$[BF_4]^-$	F	1°	691.18
24	$[PF_6]^-$	F	1°	691.83
14	$[(FSO_2)_2N]^-$	F	2°/3°	692.88
11	*$[CF_3CO_2]^-$*	*F*	*2°*	*693.13*
12	$[CF_3SO_3]^-$	F	2°	693.64
13	$[(CF_3SO_2)_2N]^-$	F	2°/3°	693.76

Table S57. Calculated E_B(O_{anion} 1s) for water samples, estimated E_B correction of +16.81 eV taken from Table S51

Anion no.	Anion	Moiety	1°/2°/3°	E_B(O_{anion} 1s) / eV
2	*$[CH_3CO_2]^-$*	*O*	*1°*	*535.79*
11	*$[CF_3CO_2]^-$*	*O*	*1°*	*536.51*
12	*$[CF_3SO_3]^-$*	*O*	*1°*	*537.20*
13	*$[(CF_3SO_2)_2N]^-$*	*O*	*1°*	*537.88*
14	*$[(FSO_2)_2N]^-$*	*O*	*1°*	*538.23*

26. Results. E_B(core,anion) for PC

Table S58. Experimental $E_B(N_{anion}$ 1s) for PC samples

Anion no.	Anion	Moiety	1°/2°/3°	$E_B(N_{anion}$ 1s) / eV
21	$[SCN]^-$	N	1°	402.54
13	$[(CF_3SO_2)_2N]^-$	N	2°/3°	404.31
14	$[(FSO_2)_2N]^-$	N	2°/3°	404.61

Table S59. Experimental $E_B(F_{anion}$ 1s) for PC samples

Anion no.	Anion	Moiety	1°/2°/3°	$E_B(F_{anion}$ 1s) / eV
24	$[PF_6]^-$	F	1°	691.60
14	$[(FSO_2)_2N]^-$	F	2°/3°	692.69
12	$[CF_3SO_3]^-$	F	2°	693.54
13	$[(CF_3SO_2)_2N]^-$	F	2°/3°	693.86

27. Results. E_B(core,anion) for TEP

Table S60. Experimental $E_B(N_{anion}\ 1s)$ for TEP samples

Anion no.	Anion	Moiety	1°/2°/3°	$E_B(N_{anion}\ 1s)$ / eV
21	$[SCN]^-$	N	1°	402.38
13	$[(CF_3SO_2)_2N]^-$	N	2°/3°	404.18
14	$[(FSO_2)_2N]^-$	N	2°/3°	404.44

Table S61. Experimental $E_B(F_{anion}\ 1s)$ for TEP samples

Anion no.	Anion	Moiety	1°/2°/3°	$E_B(F_{anion}\ 1s)$ / eV
24	$[PF_6]^-$	F	1°	691.33
14	$[(FSO_2)_2N]^-$	F	2°/3°	692.51
13	$[(CF_3SO_2)_2N]^-$	F	2°/3°	693.82

28. Results. Effect of countercations on DN_{E-XPS}

28.1. Water

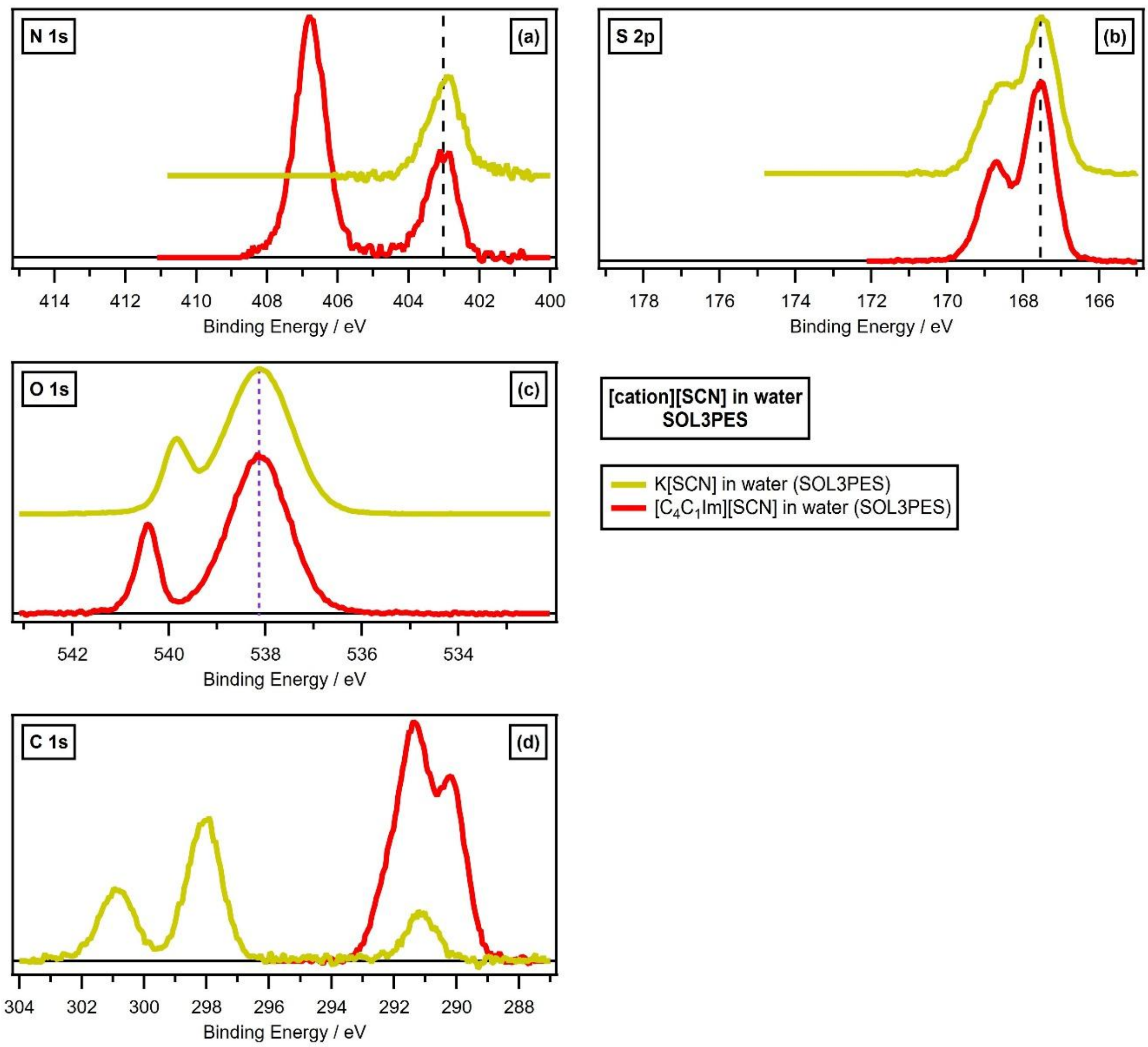

Figure S61. [cation][SCN] in water was studied with two different countercations (K^+, $[C_4C_1Im]^+$).

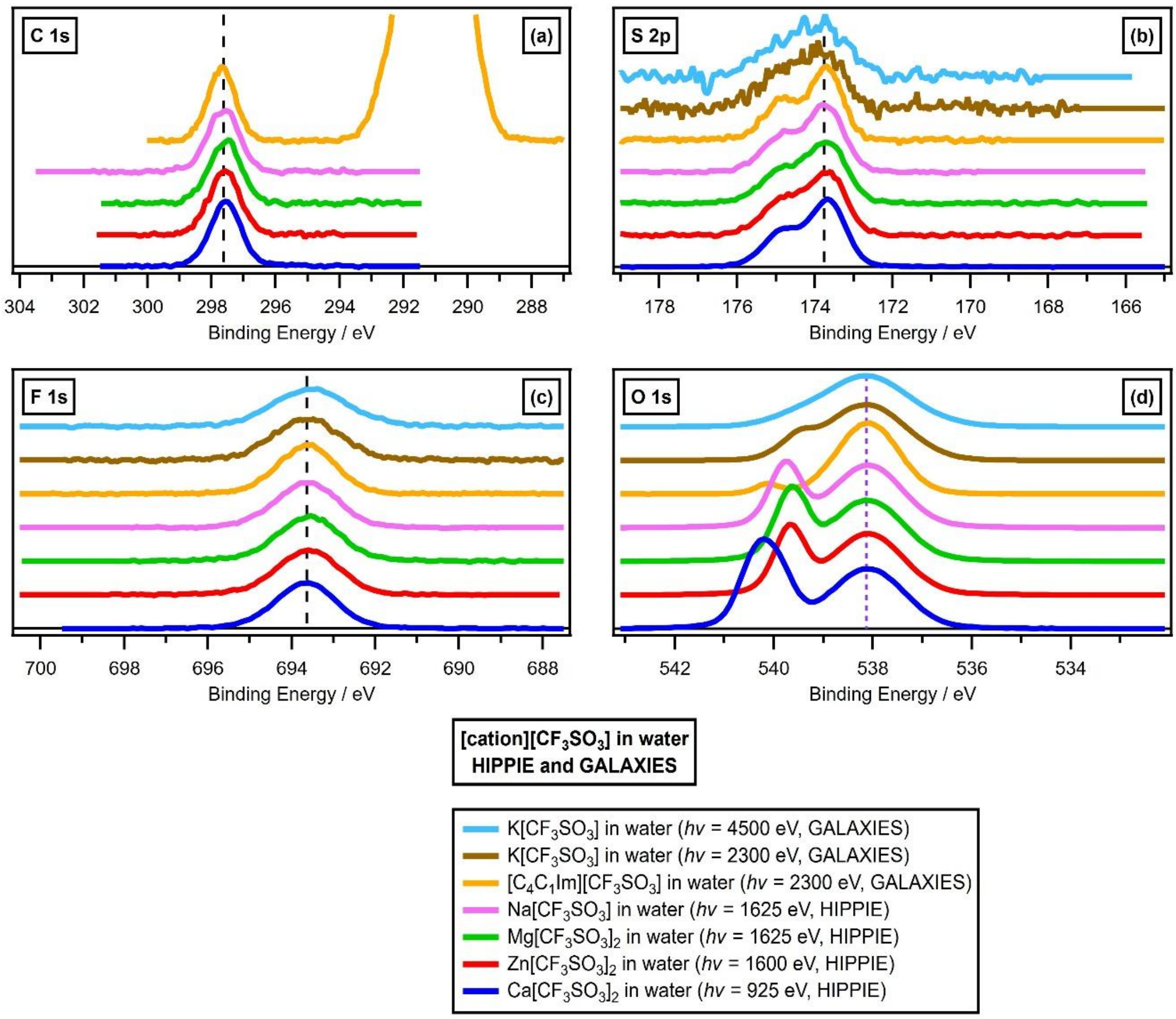

Figure S62. [cation][CF_3SO_3] in water was studied with six different countercations (Na^+, K^+, Mg^{2+}, Ca^{2+}, Zn^{2+}, [C_4C_1Im]$^+$).

28.2. PC

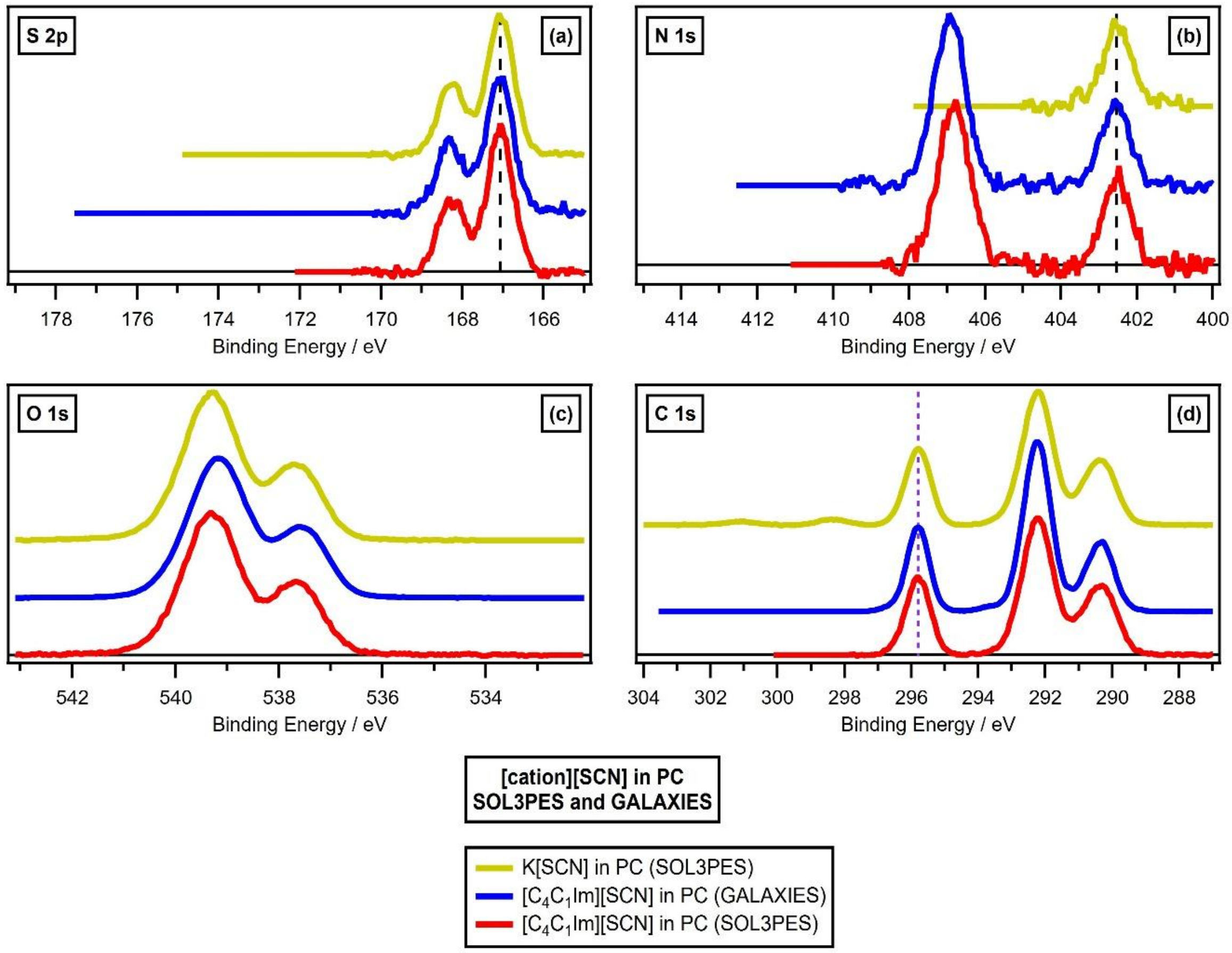

Figure S63. [cation][SCN] in PC with two different countercations (K^+, $[C_4C_1Im]^+$).

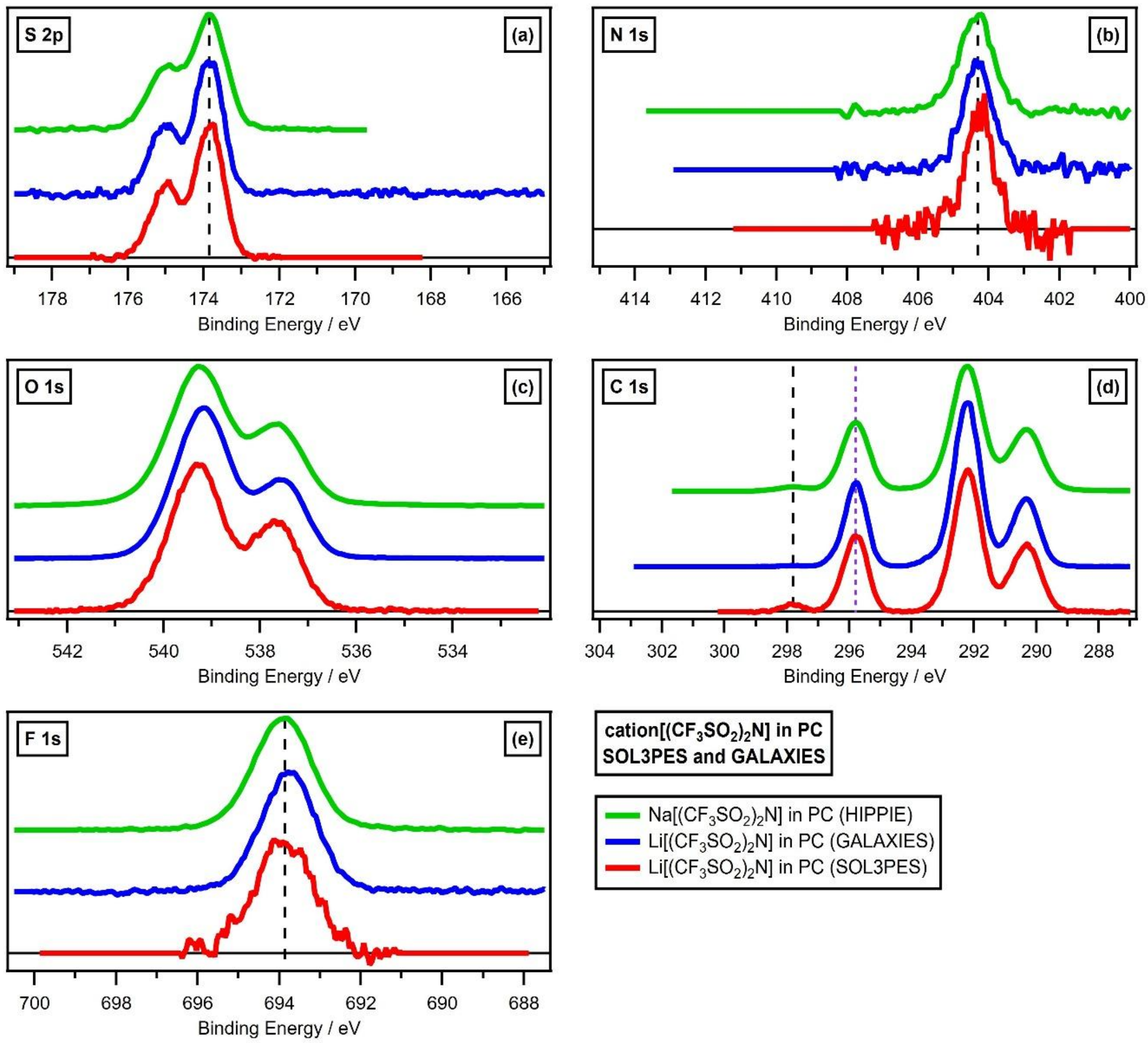

Figure S64. [cation][$(CF_3SO_2)_2N$] in PC with two different countercations (Li^+, Na^+).

28.3. TEP

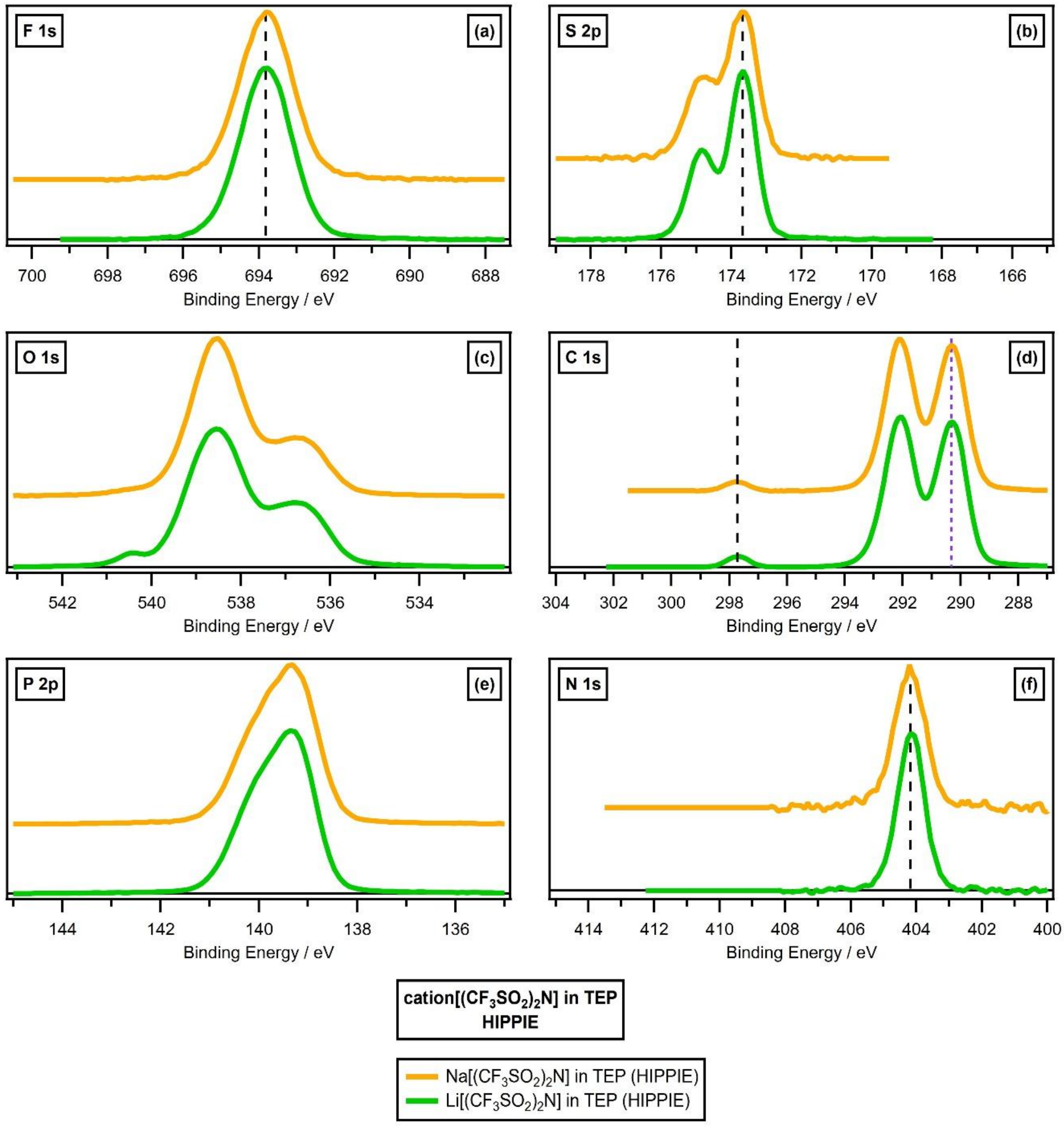

Figure S65. [cation][$(CF_3SO_2)_2N$] in TEP, with two different countercations (Li^+, Na^+).

29. Results. Experimental and calculated XP spectra for anions

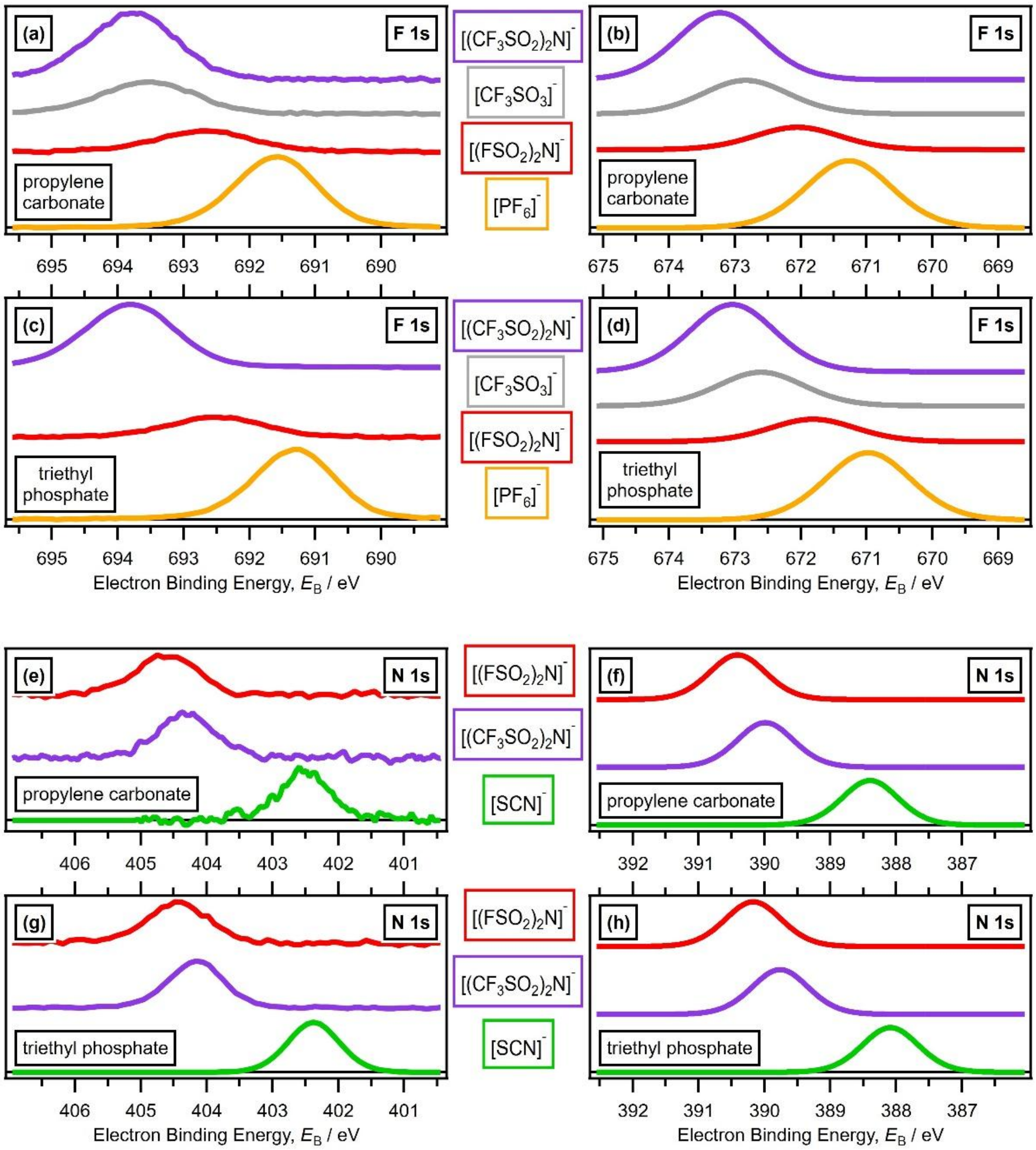

Figure S66. Experimental and calculated XPS spectra for anions in PC or TEP. F 1s XP spectra for different anions: (a) experimental in PC (same samples as in Figure 4c); (b) calculated in PC lone-anion-SMD); (c) experimental in TEP (same samples as in Figure 4d); (d) calculated in TEP lone-anion-SMD). N 1s XP spectra for different anions: (e) experimental in PC (same samples as in Figure 5c); (f) calculated in PC lone-anion-SMD); (g) experimental in TEP (same samples as in Figure 5d); (h) calculated in TEP lone-anion-SMD). Traces are vertically offset for clarity. Experimental XPS area normalization is to the number of atoms in each anion, and explained further in ESI Section 8.3. Calculated XP spectra are produced as explained in ESI Section 7. Charge referencing for experimental XP spectra: propylene carbonate E_B($C_{carbonyl}$ 1s) = 295.80 eV; triethyl phosphate E_B(C_{alkyl} 1s) = 290.32 eV (see ESI Section 8.1 for more details).

30. Results. Linear correlations of E_B(core,anion) for PC and TEP

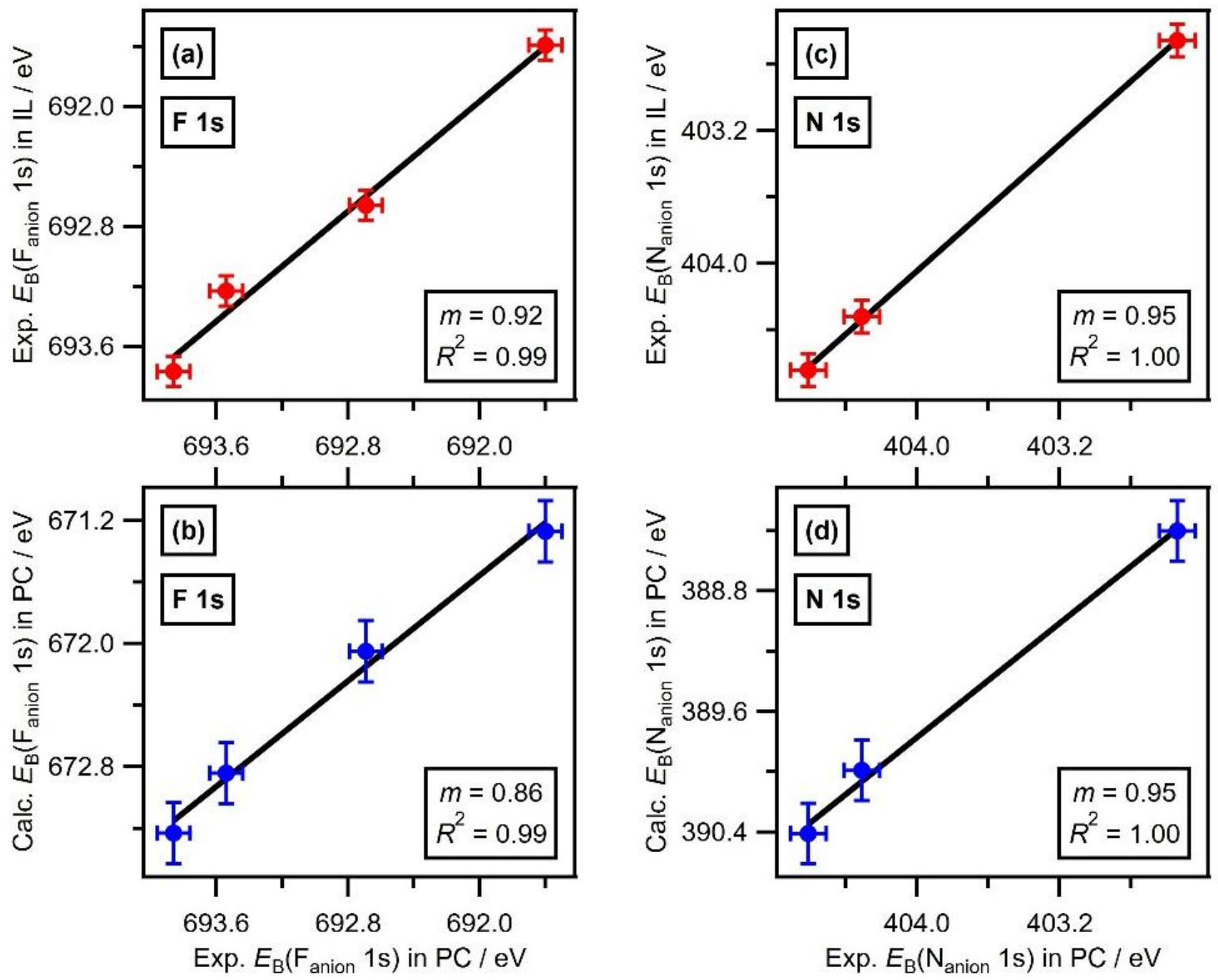

Figure S67. Linear correlations related to anions in PC. (a) Experimental $E_B(F_{anion}\ 1s)$ for ionic liquid versus experimental $E_B(F_{anion}\ 1s)$ for PC. (b) Calculated $E_B(F_{anion}\ 1s)$ (using lone-anion in PC SMD) versus experimental $E_B(F_{anion}\ 1s)$ in PC. (c) Experimental $E_B(N_{anion}\ 1s)$ for ionic liquid versus experimental $E_B(N_{anion}\ 1s)$ for PC. (d) Calculated $E_B(N_{anion}\ 1s)$ (using lone-anion in PC SMD) versus experimental $E_B(N_{anion}\ 1s)$ in PC. The experimental uncertainty in E_B(core,anion) is ±0.10 eV (unless stated otherwise in ESI Section 14). The experimental uncertainty in E_B(core,anion) is ±0.20 eV. Charge referencing: propylene carbonate $E_B(C_{carbonyl}\ 1s)$ = 295.80 eV; ionic liquid $E_B(C_{alkyl}\ 1s)$ = 289.85 eV (see ESI Section 8.1 for more details).

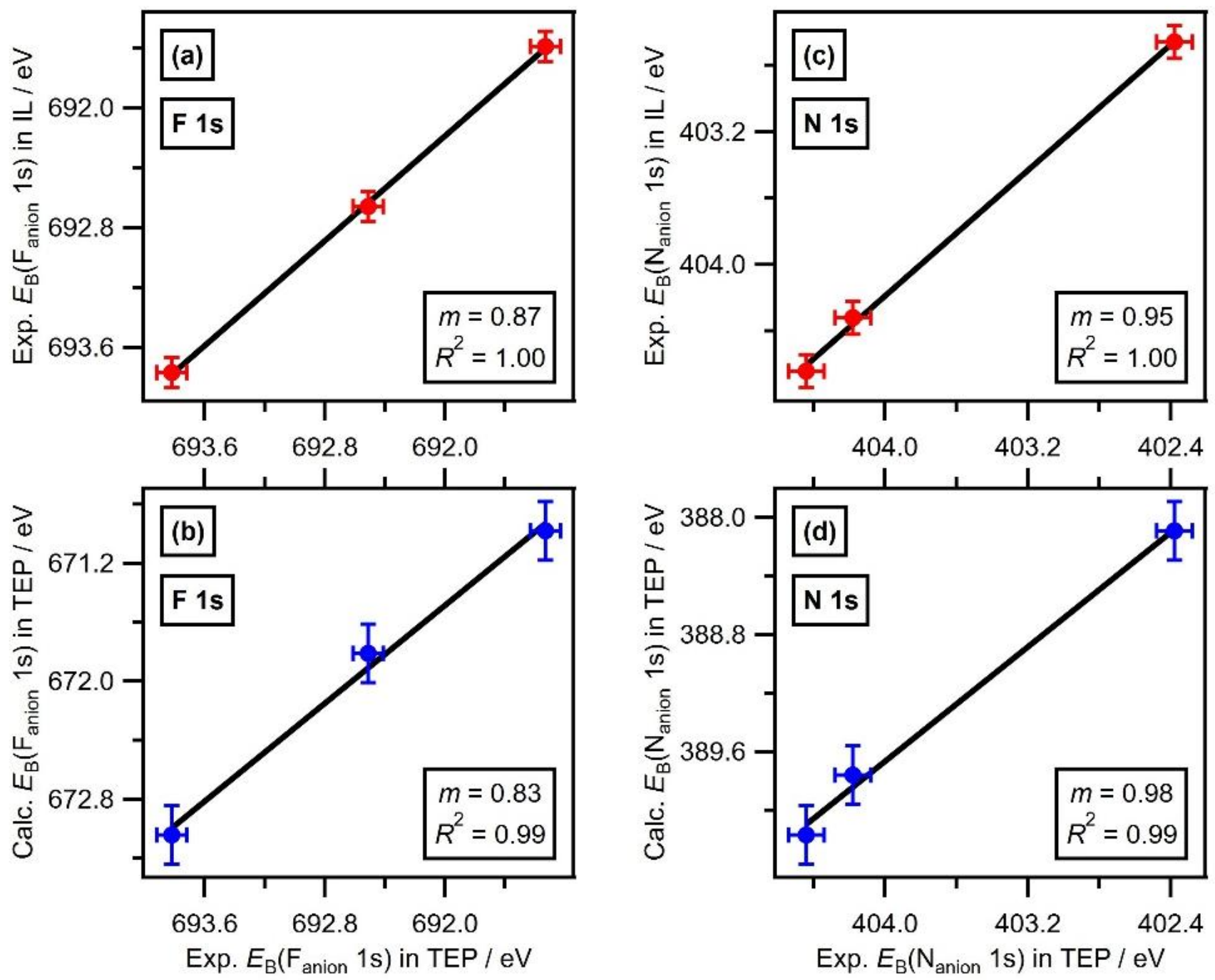

Figure S68. Linear correlations related to anions in TEP. (a) Experimental $E_B(F_{anion}\ 1s)$ for ionic liquid versus experimental $E_B(F_{anion}\ 1s)$ for TEP. (b) Calculated $E_B(F_{anion}\ 1s)$ (using lone-anion in TEP SMD) versus experimental $E_B(F_{anion}\ 1s)$ in TEP. (c) Experimental $E_B(N_{anion}\ 1s)$ for ionic liquid versus experimental $E_B(N_{anion}\ 1s)$ for TEP. (d) Calculated $E_B(N_{anion}\ 1s)$ (using lone-anion in TEP SMD) versus experimental $E_B(N_{anion}\ 1s)$ in TEP. The experimental uncertainty in E_B(core,anion) is ±0.10 eV (unless stated otherwise in ESI Section 14). The experimental uncertainty in E_B(core,anion) is ±0.20 eV. Charge referencing: triethyl phosphate $E_B(C_{alkyl}\ 1s)$ = 290.32 eV; ionic liquid $E_B(C_{alkyl}\ 1s)$ = 289.85 eV (see ESI Section 8.1 for more details).

Table S62. Linear fitting data from Figure 6a, 6b, 6c, 6d, Figure S67 and Figure S68. Coefficient values ± one standard deviation for *y* = *a* + *bx*

Type of data	Figure	***y* = *a* + *bx* data**
Experimental $E_B(F_{anion}$ 1s) for ionic liquid versus experimental $E_B(F_{anion}$ 1s) for water	6a	a = -51.755 ± 54.5<br>b = 1.0743 ± 0.0787
Calculated $E_B(F_{anion}$ 1s) (using lone-anion in water SMD) versus experimental $E_B(F_{anion}$ 1s) in water	6b	a = -129.52 ± 37.4<br>b = 1.1573 ± 0.0541
Experimental $E_B(N_{anion}$ 1s) for ionic liquid versus experimental $E_B(N_{anion}$ 1s) for water	6c	a = -46.366 ± 21.3<br>b = 1.1142 ± 0.0528
Calculated $E_B(N_{anion}$ 1s) (using lone-anion in water SMD) versus experimental $E_B(N_{anion}$ 1s) in water	6d	a = -95.217 ± 108<br>b = 1.1999 ± 0.268
Experimental $E_B(F_{anion}$ 1s) for ionic liquid versus experimental $E_B(F_{anion}$ 1s) for PC	S67a	a = 57.708 ± 52.7<br>b = 0.91656 ± 0.0761
Calculated $E_B(F_{anion}$ 1s) (using lone-anion in PC SMD) versus experimental $E_B(F_{anion}$ 1s) in PC	S67b	a = 78.423 ± 40<br>b = 0.85713 ± 0.0577
Experimental $E_B(N_{anion}$ 1s) for ionic liquid versus experimental $E_B(N_{anion}$ 1s) for PC	S67c	a = 20.223 ± 6.37<br>b = 0.95005 ± 0.0158
Calculated $E_B(N_{anion}$ 1s) (using lone-anion in PC SMD) versus experimental $E_B(N_{anion}$ 1s) in PC	S67d	a = 7.6604 ± 24.8<br>b = 0.94582 ± 0.0615
Experimental $E_B(F_{anion}$ 1s) for ionic liquid versus experimental $E_B(F_{anion}$ 1s) for TEP	S68a	a = 86.698 ± 11.8<br>b = 0.87499 ± 0.0171
Calculated $E_B(F_{anion}$ 1s) (using lone-anion in TEP SMD) versus experimental $E_B(F_{anion}$ 1s) in TEP	S68b	a = 97.573 ± 46.9<br>b = 0.82935 ± 0.0677
Experimental $E_B(N_{anion}$ 1s) for ionic liquid versus experimental $E_B(N_{anion}$ 1s) for TEP	S68c	a = 21.654 ± 13.4<br>b = 0.94687 ± 0.0331
Calculated $E_B(N_{anion}$ 1s) (using lone-anion in TEP SMD) versus experimental $E_B(N_{anion}$ 1s) in TEP	S68d	a = -6.1049 ± 28.1<br>b = 0.97963 ± 0.0697

31. Results. Bulk versus surface contributions to E_B(core,anion)

The surface sensitivity of XPS has no impact on E_B(core,anion), within experimental uncertainty. Anions at the liquid-gas surface will be solvated (at least slightly) differently to the anions solvated in the liquid bulk; most likely the anions will be more weakly solvated at the surface than in the bulk liquid. XPS is a surface-sensitive technique and not surface-specific, *i.e*. the surface will contribute towards the measured E_B(core,anion) values, but is not the only influence. Under standard conditions used for fixed-$h\nu$ XPS apparatus (*i.e*. $h\nu$ = 1486.6 eV), the bulk ionic liquid is probed, with minimal influence from the surface to E_B(core,anion).[24, 31] For significantly larger $h\nu$ on variable-$h\nu$ XPS apparatus (*e.g*. $h\nu$ 2300 eV for Li[($CF_3SO_2)_2$N] in water and $h\nu$ 2984 eV for [C_8C_1Im][($CF_3SO_2)_2$N]), clearly E_B(core,anion) will be from the bulk liquid given the large electron kinetic energies involved. E_B(core,anion) from those larger $h\nu$ measurements match those at much smaller $h\nu$ (*i.e*. smaller $h\nu$, which gives smaller electron kinetic energies and therefore more surface sensitive), *e.g*. $h\nu$ = 2984 eV and $h\nu$ = 875 eV for [C_8C_1Im][($CF_3SO_2)_2$N]); $h\nu$ = 2300 eV and $h\nu$ = 700 eV for Li[($CF_3SO_2)_2$N] in water (ESI Figure S50, ESI Figure S51, ESI Table S23, ESI Table S31).

32. Results. Comparisons of $DN_{E\text{-}XPS}$ to literature DN/β

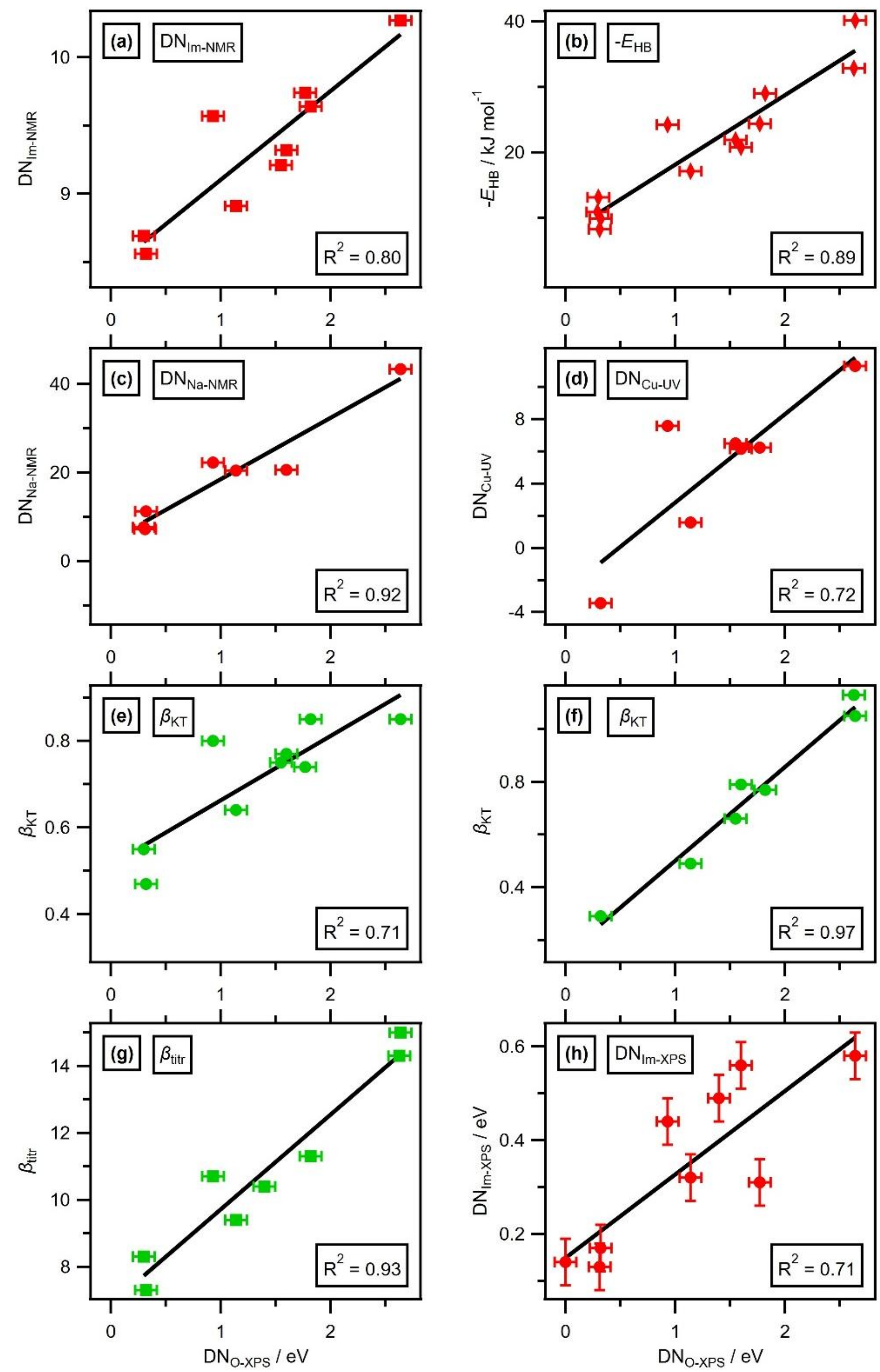

Figure S69. Measures of anion Lewis basicity/donor ability/hydrogen-bond acceptor ability plotted against oxygen-specific donor number, $DN_{O\text{-}XPS}$, of $[C_nC_1Im][A]$ neat ionic liquids: (a) Donor number measured from the chemical shifts δ(H) of the C^2-H proton by ^{1}H NMR spectroscopy of $[C_4C_1Im][A]$ in the molecular solvent CD_2Cl_2, $DN_{Im\text{-}NMR}$;[36, 37] (b) hydrogen-bond basicity calculated using ion pairs in COSMO-RS (COnductor-like Screening MOdel for Real Solvents), $-E_{HB}$;[5] (c) anion donor numbers measured from the chemical shift $\delta(Na^+)$ by ^{23}Na NMR spectroscopy of $Na[ClO_4]$ dissolved in $[C_4C_1Im][A]$ neat ionic liquids, $DN_{Na\text{-}NMR}$;[4] (d) anion donor numbers measured from the peak shift of the copper complex [Cu(acetylacetonate)(tetramethylethylenediamine)]$[ClO_4]$ by UV-vis spectroscopy in $[C_4C_1Im][A]$ neat ionic liquids, $DN_{Cu\text{-}UV}$;[36] (e) Kamlet-Taft hydrogen-bond acceptor numbers using UV-vis spectroscopy comparisons of two different neutral dye molecules in $[C_4C_1Im][A]$ neat ionic liquids, β_{KT};[1] (f) Kamlet-Taft hydrogen-bond acceptor numbers using UV-vis spectroscopy comparisons of two different neutral dye molecules in $[C_4C_1Im][A]$ neat ionic liquids, β_{KT};[2] (g) hydrogen-bond acceptor values for anions measured by titration using UV-vis spectroscopy of three different neutral dye molecules in two different organic solvents (acetonitrile and $CHCl_3$), β_{titr};[38] (h) anion donor number measured from the shifts of $E_B(O_{anion}\ 1s)$ by core-level XPS of $[C_8C_1Im][A]$ neat ionic liquids, $DN_{Im\text{-}XPS}$ [11]. Red = cation probes. Green = neutral probes. Circles = measured in neat ionic liquids, the same as $E_B(O_{anion}\ 1s)$. Squares = measured diluted in molecular solvent. Diamonds = calculations.

For our probe-independent primary $DN_{N\text{-}XPS}$, we have measurements for four anions (Table 3, Figure S55). A limitation for comparisons of our local $DN_{N\text{-}XPS}$ to other donor scales is that the literature datasets often do not contain these four anions (only four of the literature scales contain all four anions) with one scale containing none of these anions.

For our probe-independent anion primary $DN_{F\text{-}XPS}$, we have measurements for four anions (Table 4, Figure S56). There are two limitations for comparisons of our local $DN_{F\text{-}XPS}$ to other donor scales: firstly, the literature datasets often do not contain these four anions (only four of the literature scales contain all four anions) with one scale containing only one of these anions; secondly, the range of $E_B(F_{anion}\ 1s)$ for anion primary $DN_{F\text{-}XPS}$ is small at 0.9 eV.

Table S63. Data used for correlations of literature DN/β versus oxygen-specific donor number, $DN_{O\text{-}XPS}$, in Figure 6e, 6f and Figure S69

Anion no.	Anion	$DN_{O\text{-}XPS}$ (here)	$DN_{Im\text{-}NMR}$ (ref. [36, 37])	$-E_{HB}$ (ref. [5])	$DN_{Na\text{-}NMR}$ (ref. [4])	$DN_{Cu\text{-}UV}$ (ref. [36])	β_{KT} (ref. [1])	β_{KT} (ref. [2])	β_{titr} (ref. [38])	$DN_{Im\text{-}XPS}$ (ref. [11]) [a]
		**Figure 6e, 6f, S69**	**Figure S69a**	**Figure S69b**	**Figure 6e, S69c**	**Figure S69d**	**Figure S69e**	**Figure 6f, S69f**	**Figure S68g**	**Figure S69h**
2	$[CH_3CO_2]^-$	2.64	10.27	40.17	43.3	11.32	0.85	1.05	15.0	0.58
9	$(CH_3O)_2PO_2]^-$	2.63		32.85				1.13	14.3	
3	$[CH_3SO_3]^-$	1.82	9.64	29.03			0.85	0.77	11.3	
11	$[CF_3CO_2]^-$	1.77	9.74	24.38		6.23	0.74			0.31
8	$[C_8H_{17}OSO_3]^-$	1.60	9.32	20.76	20.6	6.16	0.77	0.79		0.56
7	$[CH_3OSO_3]^-$	1.55	9.21	21.88		6.49	0.75	0.66		
6	$[HOSO_3]^-$	1.40							10.4	0.49
12	$[CF_3SO_3]^-$	1.14	8.91	17.11	20.4	1.57	0.64	0.49	9.4	0.32
1	$[NO_3]^-$	0.93	9.57	24.21	22.2	7.58	0.80		10.7	0.44
13	$[(CF_3SO_2)_2N]^-$	0.32	8.56	9.86	11.2	-3.44	0.47	0.29	7.3	0.17
5	$[ClO_4]^-$	0.30	8.69	13.11	7.6		0.55		8.3	
10	$[B(C_2O_4)_2]^-$	0.29		10.88						
15	$[(C_2F_5SO_2)_2N]^-$	0.31		8.27	7.2					0.13
14	$[(FSO_2)_2N]^-$	0.00								0.14

[a] $E_B(N_{cation}\ 1s)$ from reference [11] are converted here to $DN_{Im\text{-}XPS}$ values using the equation $DN_{Im\text{-}XPS} = 402.27 - E_B(N_{cation}\ 1s)$ (because the largest $E_B(N_{cation}\ 1s)$ value is 402.27 eV in reference [11])

Table S64. Linear fitting data from Figure 6e, 6f and Figure S69 for literature DN/β versus oxygen-specific donor number, $DN_{O\text{-}XPS}$. Coefficient values ± one standard deviation for $y = a + bx$

Type of data	Figure	$y = a + bx$ data
donor number measured from the chemical shifts δ(H) of the C^2-H proton by ^1H NMR spectroscopy of $[C_4C_1Im][A]$ in the molecular solvent CD_2Cl_2, $DN_{Im\text{-}NMR}$.[36, 37]	S69a	a = 8.4515 ± 0.186<br>b = 0.65008 ± 0.122
hydrogen-bond basicity calculated using ion pairs in COSMO-RS (COnductor-like Screening MOdel for Real Solvents), $-E_{HB}$.[5]	S69b	a = 7.496 ± 1.84<br>b = 10.625 ± 1.21
anion donor numbers measured from the chemical shift $\delta(Na^+)$ by ^{23}Na NMR spectroscopy of $Na[ClO_4]$ dissolved in $[C_4C_1Im][A]$ neat ionic liquids, $DN_{Na\text{-}NMR}$.[4]	6e and S69c	a = 4.6083 ± 2.37<br>b = 13.846 ± 1.81
anion donor numbers measured from the peak shift of the copper complex [Cu(acetylacetonate)(tetramethylethylenediamine)][ClO_4] by UV-vis spectroscopy in $[C_4C_1Im][A]$ neat ionic liquids, $DN_{Cu\text{-}UV}$.[36]	S69d	a = -2.681 ± 2.44<br>b = 5.4951 ± 1.55
Kamlet-Taft hydrogen-bond acceptor numbers using UV-vis spectroscopy comparisons of two different neutral dye molecules in $[C_4C_1Im][A]$ neat ionic liquids, β_{KT}.[1]	S69e	a = 0.51444 ± 0.0547<br>b = 0.14831 ± 0.0361
Kamlet-Taft hydrogen-bond acceptor numbers using UV-vis spectroscopy comparisons of two different neutral dye molecules in $[C_4C_1Im][A]$ neat ionic liquids, β_{KT}.[2]	6f and S69f	a = 0.1454 ± 0.0516<br>b = 0.35574 ± 0.0281
hydrogen-bond acceptor values for anions measured by titration using UV-vis spectroscopy of three different neutral dye molecules in two different organic solvents (MeCN and $CHCl_3$), β_{titr}.[38]	S69g	a = 6.8847 ± 0.525<br>b = 2.8285 ± 0.32
anion donor numbers measured from the binding energy shift $E_B(N_{cation}\ 1s)$ by core-level XPS of $[C_8C_1Im][A]$ neat ionic liquids, $DN_{Im\text{-}XPS}$.[11]	S69h	a = 0.14865 ± 0.0592<br>b = 0.17826 ± 0.0431

33. References

1. S. Spange, R. Lungwitz and A. Schade. Correlation of molecular structure and polarity of ionic liquids. *J. Mol. Liq.* **2014**, *192*, 137-143. DOI: 10.1016/j.molliq.2013.06.016.
2. M. A. Ab Rani, A. Brandt, L. Crowhurst, A. Dolan, N. H. Hassan, J. P. Hallett, P. A. Hunt, M. Lui, H. Niedermeyer, J. M. Perez-Arlandis, M. Schrems, T. Q. To, T. Welton and R. Wilding. Understanding the polarity of ionic liquids. *Phys. Chem. Chem. Phys.* **2011**, *13* (37), 16831-16840. DOI: 10.1039/c1cp21262a.
3. C. Laurence, S. Mansour, D. Vuluga and J. Legros. Measurement of the hydrogen bond acceptance of ionic liquids and green solvents by the F-19 solvatomagnetic comparison method. *Green Chem.* **2021**, *23* (4), 1816-1822. DOI: 10.1039/d0gc04104a.
4. M. Schmeisser, P. Illner, R. Puchta, A. Zahl and R. van Eldik. Gutmann Donor and Acceptor Numbers for Ionic Liquids. *Chem.-Eur. J.* **2012**, *18* (35), 10969-10982. DOI: 10.1002/chem.201200584.
5. A. F. M. Cláudio, L. Swift, J. P. Hallett, T. Welton, J. A. P. Coutinho and M. G. Freire. Extended scale for the hydrogen-bond basicity of ionic liquids. *Phys. Chem. Chem. Phys.* **2014**, *16* (14), 6593-6601. DOI: 10.1039/c3cp55285c.
6. M. Chen, J. Zhang, X. Ji, J. Fu and G. Feng. Progress on predicting the electrochemical stability window of electrolytes. *Curr. Opin. Electrochem.* **2022**, *34*, 9. DOI: 10.1016/j.coelec.2022.101030.
7. O. Borodin, G. A. Giffin, A. Moretti, J. B. Haskins, J. W. Lawson, W. A. Henderson and S. Passerini. Insights into the Structure and Transport of the Lithium, Sodium, Magnesium, and Zinc Bis(trifluoromethansulfonyl)imide Salts in Ionic Liquids. *J. Phys. Chem. C* **2018**, *122* (35), 20108-20121. DOI: 10.1021/acs.jpcc.8b05573.
8. T. Cremer, C. Kolbeck, K. R. J. Lovelock, N. Paape, R. Wölfel, P. S. Schulz, P. Wasserscheid, H. Weber, J. Thar, B. Kirchner, F. Maier and H. P. Steinrück. Towards a Molecular Understanding of Cation-Anion Interactions-Probing the Electronic Structure of Imidazolium Ionic Liquids by NMR Spectroscopy, X-ray Photoelectron Spectroscopy and Theoretical Calculations. *Chem.-Eur. J.* **2010**, *16* (30), 9018-9033. DOI: 10.1002/chem.201001032.
9. F. K. Towers Tompkins, L. G. Parker, R. M. Fogarty, J. M. Seymour, R. Rowe, R. G. Palgrave, R. P. Matthews, R. A. Bennett, P. A. Hunt and K. R. J. Lovelock. Efficient prediction of the local electronic structure of ionic liquids from low-cost calculations. *Phys. Chem. Chem. Phys.* **2025**, *27* (17), 8803-8812. DOI: 10.1039/d5cp00892a.
10. R. M. Fogarty, R. G. Palgrave, R. A. Bourne, K. Handrup, I. J. Villar-Garcia, D. J. Payne, P. A. Hunt and K. R. J. Lovelock. Electron spectroscopy of ionic liquids: experimental identification of atomic orbital contributions to valence electronic structure. *Phys. Chem. Chem. Phys.* **2019**, *21* (35), 18893-18910. DOI: 10.1039/c9cp02200g.
11. E. Gousseva, F. K. Towers Tompkins, J. M. Seymour, L. G. Parker, C. J. Clarke, R. G. Palgrave, R. A. Bennett, R. Grau-Crespo and K. R. J. Lovelock. Anion-Dependent Strength Scale of Interactions in Ionic Liquids from X-ray Photoelectron Spectroscopy, Ab Initio Molecular Dynamics, and Density Functional Theory. *J. Phys. Chem. B* **2024**, *128* (20), 5030-5043. DOI: 10.1021/acs.jpcb.4c00362.
12. R. M. Fogarty, R. Rowe, R. P. Matthews, M. T. Clough, C. R. Ashworth, A. Brandt, P. J. Corbett, R. G. Palgrave, E. F. Smith, R. A. Bourne, T. W. Chamberlain, P. B. J. Thompson, P. A. Hunt and K. R. J. Lovelock. Atomic charges of sulfur in ionic liquids: experiments and calculations. *Faraday Discuss.* **2018**, *206*, 183-201. DOI: 10.1039/c7fd00155j.
13. L. S. Longo, E. F. Smith and P. Licence. Study of the Stability of 1-Alkyl-3-methylimidazolium Hexafluoroantimonate(V) Based Ionic Liquids Using X-ray Photoelectron Spectroscopy. *ACS Sustain. Chem. Eng.* **2016**, *4* (11), 5953-5962. DOI: 10.1021/acssuschemeng.6b00919.
14. J. M. Seymour, E. Gousseva, A. I. Large, C. J. Clarke, P. Licence, R. M. Fogarty, D. A. Duncan, P. Ferrer, F. Venturini, R. A. Bennett, R. G. Palgrave and K. R. J. Lovelock. Experimental measurement and prediction of ionic liquid ionisation energies. *Phys. Chem. Chem. Phys.* **2021**, *23* (37), 20957-20973. DOI: 10.1039/d1cp02441h.
15. J. M. Seymour, E. Gousseva, F. K. Towers Tompkins, L. G. Parker, N. O. Alblewi, C. J. Clarke, S. Hayama, R. G. Palgrave, R. A. Bennett, R. P. Matthews and K. R. J. Lovelock. Unravelling the complex speciation of halozincate ionic liquids using X-ray spectroscopies and calculations. *Faraday Discuss.* **2024**, *253* (0), 251-272. DOI: 10.1039/d4fd00029c.
16. A. W. Taylor, S. Men, C. J. Clarke and P. Licence. Acidity and basicity of halometallate-based ionic liquids from X-ray photoelectron spectroscopy. *RSC Adv.* **2013**, *3* (24), 9436-9445. DOI: 10.1039/c3ra40260f.
17. J. M. Seymour, E. Gousseva, L. G. Parker, F. K. T. Tompkins, R. M. Fogarty, L. Frankemoelle, R. Rowe, C. J. Clarke, D. A. Duncan, R. G. Palgrave, R. A. Bennett, P. A. Hunt and K. R. J. Lovelock. d10s2 Post-Transition Metal Anions: Identifying and Analyzing Their Dual-Mode Lewis Basicity. *J. Phys. Chem. Lett.* **2025**, *16* (11), 2831-2836. DOI: 10.1021/acs.jpclett.4c03649.
18. B. May, M. Honle, B. Heller, F. Greco, R. Bhuin, H. P. Steinrück and F. Maier. Surface-Induced Changes in the Thermochromic Transformation of an Ionic Liquid Cobalt Thiocyanate Complex. *J. Phys. Chem. Lett.* **2017**, *8* (6), 1137-1141. DOI: 10.1021/acs.jpclett.7b00142.
19. R. M. Fogarty, R. P. Matthews, P. A. Hunt and K. R. J. Lovelock. Accurate prediction of ionic liquid density-of-states from low-cost calculations. *Phys. Chem. Chem. Phys.* **2025**, *27* (17), 9068-9075. DOI: 10.1039/d5cp00214a.
20. F. K. Towers Tompkins, L. G. Parker, N. O. Alblewi, J. M. Seymour, E. Gousseva, C. D. Smith, R. A. Bennett, D. C. Grinter, W. Quevedo, R. Seidel, G. Öhrwall, R. Temperton, R. P. Matthews and K. R. J. Lovelock. Quantifying Lithium-Ligand Interactions using Lithium X-ray Photoelectron Spectroscopy. **2026**, in preparation.
21. I. J. Villar-Garcia, K. R. J. Lovelock, S. Men and P. Licence. Tuning the electronic environment of cations and anions using ionic liquid mixtures. *Chem. Sci.* **2014**, *5* (6), 2573-2579. DOI: 10.1039/c4sc00106k.

22. I. J. Villar-Garcia, E. F. Smith, A. W. Taylor, F. L. Qiu, K. R. J. Lovelock, R. G. Jones and P. Licence. Charging of ionic liquid surfaces under X-ray irradiation: the measurement of absolute binding energies by XPS. *Phys. Chem. Chem. Phys.* **2011**, *13* (7), 2797-2808. DOI: 10.1039/c0cp01587c.
23. C. Kolbeck, M. Killian, F. Maier, N. Paape, P. Wasserscheid and H. P. Steinrück. Surface characterization of functionalized imidazolium-based ionic liquids. *Langmuir* **2008**, *24* (17), 9500-9507. DOI: 10.1021/la801261h.
24. C. Kolbeck, T. Cremer, K. R. J. Lovelock, N. Paape, P. S. Schulz, P. Wasserscheid, F. Maier and H. P. Steinrück. Influence of Different Anions on the Surface Composition of Ionic Liquids Studied Using ARXPS. *J. Phys. Chem. B* **2009**, *113* (25), 8682-8688. DOI: 10.1021/jp902978r.
25. T. Doherty, M. Mora-Pale, S. Foley, R. Linhardt and J. Dordick. Ionic liquid solvent properties as predictors of lignocellulose pretreatment efficacy. *Green Chem.* **2010**, *12* (11), 1967-1975. DOI: 10.1039/c0gc00206b.
26. Y. Wu, T. Sasaki, K. Kazushi, T. Seo and K. Sakurai. Interactions between spiropyrans and room-temperature ionic liquids: Photochromism and solvatochromism. *J. Phys. Chem. B* **2008**, *112* (25), 7530-7536. DOI: 10.1021/jp800957c.
27. K. Burger, F. Tschimarov and H. Ebel. XPS-ESCA APPLIED TO QUICK-FROZEN SOLUTIONS .1. STUDY OF NITROGEN-COMPOUNDS IN AQUEOUS-SOLUTIONS. *J. Electron. Spectrosc. Relat. Phenom.* **1977**, *10* (4), 461-465. DOI: 10.1016/0368-2048(77)85042-1.
28. S. ADURU, S. CONTARINI and J. RABALAIS. ELECTRON-STIMULATED, X-RAY-STIMULATED, AND ION-STIMULATED DECOMPOSITION OF NITRATE SALTS. *J. Phys. Chem.* **1986**, *90* (8), 1683-1688. DOI: 10.1021/j100399a045.
29. B. Folkesson. ESCA STUDIES ON CHARGE-DISTRIBUTION IN SOME DINITROGEN COMPLEXES OF RHENIUM, IRIDIUM, RUTHENIUM, AND OSMIUM. *Acta Chem. Scand.* **1973**, *27* (1), 287-302. DOI: 10.3891/acta.chem.scand.27-0287.
30. W. E. Swartz and R. A. Alfonso. N(1s) PHOTOELECTRON-SPECTRA OF TRANSITION-METAL BIGUANIDE COMPLEXES. *J. Electron. Spectrosc. Relat. Phenom.* **1974**, *4* (4), 351-354. DOI: 10.1016/0368-2048(74)80066-6.
31. K. R. J. Lovelock, C. Kolbeck, T. Cremer, N. Paape, P. S. Schulz, P. Wasserscheid, F. Maier and H. P. Steinrück. Influence of Different Substituents on the Surface Composition of Ionic Liquids Studied Using ARXPS. *J. Phys. Chem. B* **2009**, *113* (9), 2854-2864. DOI: 10.1021/jp810637d.
32. R. K. Blundell and P. Licence. Quaternary ammonium and phosphonium based ionic liquids: a comparison of common anions. *Phys. Chem. Chem. Phys.* **2014**, *16* (29), 15278-15288. DOI: 10.1039/c4cp01901f.
33. R. Signorell and B. Winter. Photoionization of the aqueous phase: clusters, droplets and liquid jets. *Phys. Chem. Chem. Phys.* **2022**, *24* (22), 13438-13460. DOI: 10.1039/d2cp00164k.
34. K. R. J. Lovelock, E. F. Smith, A. Deyko, I. J. Villar-Garcia, P. Licence and R. G. Jones. Water adsorption on a liquid surface. *Chem. Commun.* **2007**, DOI: 10.1039/B711680B (46), 4866-4868. DOI: 10.1039/B711680B.
35. F. K. Towers Tompkins, L. G. Parker, R. M. Fogarty, J. M. Seymour, E. Gousseva, D. C. Grinter, R. G. Palgrave, C. D. Smith, R. A. Bennett, R. P. Matthews and K. R. J. Lovelock. Controlling and predicting alkyl-onium electronic structure. *Chem. Commun.* **2024**, *60* (77), 10756-10759. DOI: 10.1039/d4cc03388d.
36. M. Holzweber, R. Lungwitz, D. Doerfler, S. Spange, M. Koel, H. Hutter and W. Linert. Mutual Lewis Acid-Base Interactions of Cations and Anions in Ionic Liquids. *Chem.-Eur. J.* **2013**, *19* (1), 288-293. DOI: 10.1002/chem.201201978.
37. R. Lungwitz and S. Spange. A hydrogen bond accepting (HBA) scale for anions, including room temperature ionic liquids. *New J. Chem.* **2008**, *32* (3), 392-394. DOI: 10.1039/b714629a.
38. S. J. Pike, J. J. Hutchinson and C. A. Hunter. H-Bond Acceptor Parameters for Anions. *J. Am. Chem. Soc.* **2017**, *139* (19), 6700-6706. DOI: 10.1021/jacs.7b02008.
